# Rotating stars in relativity

**DOI:** 10.1007/s41114-017-0008-x

**Published:** 2017-11-29

**Authors:** Vasileios Paschalidis, Nikolaos Stergioulas

**Affiliations:** 1 Theoretical Astrophysics Program, Departments of Astronomy and Physics, University of Arizona, Tucson, AZ 85721 USA; 2 Department of Physics, Princeton University, Princeton, NJ 08544 USA; 3 Department of Physics, Aristotle University of Thessaloniki, 54124 Thessaloniki, Greece

**Keywords:** Relativistic stars, Rotation, Stability, Oscillations, Magnetic fields, Numerical relativity

## Abstract

Rotating relativistic stars have been studied extensively in recent years, both theoretically and observationally, because of the information they might yield about the equation of state of matter at extremely high densities and because they are considered to be promising sources of gravitational waves. The latest theoretical understanding of rotating stars in relativity is reviewed in this updated article. The sections on equilibrium properties and on nonaxisymmetric oscillations and instabilities in *f*-modes and *r*-modes have been updated. Several new sections have been added on equilibria in modified theories of gravity, approximate universal relationships, the one-arm spiral instability, on analytic solutions for the exterior spacetime, rotating stars in LMXBs, rotating strange stars, and on rotating stars in numerical relativity including both hydrodynamic and magnetohydrodynamic studies of these objects.

## Introduction

Rotating relativistic stars are of fundamental interest in physics. Their bulk properties constrain the proposed equations of state for densities greater than the nuclear saturation density. Accreted matter in their gravitational fields undergoes high-frequency oscillations that could become a sensitive probe for general relativistic effects. Temporal changes in the rotational period of millisecond pulsars can also reveal a wealth of information about important physical processes inside the stars or of cosmological relevance. In addition, rotational instabilities can result in the generation of copious amounts of gravitational waves the detection of which would initiate a new field of observational asteroseismology of relativistic stars. The latter is of particular importance because with the first direct detections of gravitational waves by the LIGO and VIRGO collaborations (Abbott et al. 2016a, b) the era of gravitational wave astronomy has arrived.

There exist several independent numerical codes for obtaining accurate models of rotating neutron stars in full general relativity, including two that are publicly available. The uncertainty in the high-density equation of state still allows numerically constructed maximum mass models to differ by more than 50% in mass, radius and angular velocity, and by a larger factor in the moment of inertia. Given these uncertainties, an absolute upper limit on the rotation of relativistic stars can be obtained by imposing causality as the only requirement on the equation of state. It then follows that gravitationally bound stars cannot rotate faster than 0.41 ms.

In rotating stars, nonaxisymmetric oscillations have been studied in various approximations (the Newtonian limit and the post-Newtonian approximation, the slow rotation limit, the Cowling approximation, the spatial conformal flatness approximation) as an eigenvalue problem. Normal modes in full general relativity have been obtained through numerical simulations only. Time evolutions of the linearized equations have improved our understanding of the spectrum of axial and hybrid modes in relativistic stars.

Nonaxisymmetric instabilities in rotating stars can be driven by the emission of gravitational waves [Chandrasekhar–Friedman–Schutz (CFS) instability] or by viscosity. Relativity strengthens the former, but weakens the latter. Nascent neutron stars can be subject to the $l=2$ bar mode CFS instability, which would turn them into a strong gravitational wave source. Axial fluid modes in rotating stars (*r*-modes) have received considerable attention since the discovery that they are generically unstable to the emission of gravitational waves. The *r*-mode instability could slow down newly-born relativistic stars and limit their spin during accretion-induced spin-up, which would explain the absence of millisecond pulsars with rotational periods $<\,\sim$ 1.5 ms. Gravitational waves from the *r*-mode instability could become detectable if the amplitude of *r*-modes is sufficiently large, however, nonlinear effects seem to set a small saturation amplitude on long timescales. Nevertheless, if the signal persists for a long time, even a small amplitude could become detectable. Highly differentially rotating neutron stars are also subject to the development of a one-arm ($m=1$) instability, as well as to the development of a dynamical bar-mode ($m=2$) instability which both act as emitters of potentially detectable gravitational waves.

Recent advances in numerical relativity have enabled the long-term dynamical evolution of rotating stars. Several interesting phenomena, such as dynamical instabilities, pulsation modes, and neutron star and black hole formation in rotating collapse have now been studied in full general relativity. The latest studies include realistic equations of state and also magnetic fields.

The aim of this article is to present a summary of theoretical and numerical methods that are used to describe the equilibrium properties of rotating relativistic stars, their oscillations and dynamical evolution. The focus is on the most recently available publications in the field, in order to rapidly communicate new methods and results. At the end of some sections, the reader is directed to papers that could not be presented in detail here, or to other review articles. As new developments in the field occur, updated versions of this article will appear. Another review on rotating relativistic stars has appeared by Gourgoulhon (2010), while monographs appeared by Meinel et al. (2008) and Friedman and Stergioulas (2013). In several sections, our *Living Review* article updates and extends previous versions (Stergioulas 1998, 2003) using also abridged discussions of topics from Friedman and Stergioulas (2013).

*Notation and conventions* Throughout the article, gravitational units, where $G = c = 1$ (these units are also referred to as geometrized), will be adopted in writing the equations governing stellar structure and dynamics, while numerical properties of stellar models will be listed in cgs units, unless otherwise noted. We use the conventions of Misner et al. (1973) for the signature of the spacetime metric $(-+++)$ and for signs of the curvature tensor and its contractions. Spacetime indices will be denoted by Greek letters, $\alpha$, $\beta , \ldots$, while Latin $a, b, \ldots$ characters will be reserved to denote spatial indices. (Readers familiar with abstract indices can regard indices early in the alphabet as abstract, while indices $\mu , \nu , \lambda$ and *i*, *j*, *k* will be concrete, taking values $\mu = 0,1,2,3$, $i=1,2,3$.) Components of a vector $u^\alpha$ in an orthonormal frame, $\{ \mathbf{e}_{\hat{0}}, \ldots , \mathbf{e}_{\hat{3}}\}$, will be written as $\{u^{\hat{0}}, \ldots , u^{\hat{3}}\}$. Parentheses enclosing a set of indices indicate symmetrization, while square brackets indicate anti-symmetrization.

Numbers that rely on physical constants are based on the values $c = 2.9979 \times 10^{10}\,\mathrm {cm\ s^{-1}}$, $G = 6.670\times 10^{-8}\,\mathrm {g^{-1}\ cm^{3}\ s^{-2}}$, $\hbar = 1.0545\times 10^{-27}\,\mathrm {g\ cm^{2}\ s^{-1}}$, baryon mass $m_B = 1.659\times 10^{-24}\,\mathrm {g}$, and $M_\odot = 1.989\times 10^{33}\,\mathrm {g} = 1.477\,\mathrm {km}$.

## The equilibrium structure of rotating relativistic stars

### Assumptions

A relativistic star can have a complicated structure (such as a solid crust, magnetic field, possible superfluid interior, possible quark core, etc.). Depending on which phase in the lifetime of the star one wants to study, a number of physical effects can be ignored, so that the description becomes significantly simplified. In the following, we will take the case of a *cold, uniformly rotating relativistic star* as a reference case and mention additional assumptions for other cases, where necessary.

The matter can be modeled as a perfect fluid because observations of pulsar glitches are consistent with departures from a perfect fluid equilibrium (due to the presence of a solid crust) of order $10^{-5}$ (see Friedman and Ipser 1992). The temperature of a cold neutron star has a negligible affect on its bulk properties and can be assumed to be 0 K, because its thermal energy ($\ll 1 \,\mathrm {MeV} \sim 10^{10}\,\mathrm {K}$) is much smaller than Fermi energies of the interior ($>\,60 \,\mathrm {MeV}$). One can then use a *one-parameter*, *barotropic* equation of state (EOS) to describe the matter:

1 $$\begin{aligned} \varepsilon = \varepsilon (P), \end{aligned}$$

where $\varepsilon$ is the energy density and *P* is the pressure. At birth, a neutron star is expected to be rotating differentially, but as the neutron star cools, several mechanisms can act to enforce uniform rotation. Kinematical shear viscosity is acting against differential rotation on a timescale that has been estimated to be (Flowers and Itoh 1976, 1979; Cutler and Lindblom 1987)

2 $$\begin{aligned} \tau \sim 18 \left( \frac{\rho }{10^{15}\,\mathrm {g\ cm}^{-3}}\right) ^{-5/4} \left( \frac{T}{10^9\,\mathrm {K}}\right) ^2 \left( \frac{R}{10^6\,\mathrm {cm}}\right) \,\mathrm {yr}, \end{aligned}$$

where $\rho$, *T* and *R* are the central density, temperature, and radius of the star. It has also been suggested that convective and turbulent motions may enforce uniform rotation on a timescale of the order of days (Hegyi 1977). Shapiro (2000) suggested that magnetic braking of differential rotation by Alfvén waves could be the most effective damping mechanism, acting on short timescales, possibly of the order of minutes.

Within a short time after its formation, the temperature of a neutron star becomes less than $10^{10}\,\mathrm {K}$ (due to neutrino emission). When the temperature drops further, below roughly $10^{9} \,\mathrm {K}$, its outer core is expected to become superfluid (see Mendell 1998 and references therein). Rotation causes superfluid neutrons to form an array of quantized vortices, with an intervortex spacing of

3 $$\begin{aligned} d_{\mathrm {n}} \sim 3.4 \times 10^{-3} \varOmega _2^{-1/2} \,\mathrm {cm}, \end{aligned}$$

where $\varOmega _2$ is the angular velocity of the star in $10^2 \,\mathrm {s}^{-1}$. On scales much larger than the intervortex spacing, e.g., of the order of centimeters or meters, the fluid motions can be averaged and the rotation can be considered to be uniform (Sonin 1987). With such an assumption, the error in computing the metric is of order

4 $$\begin{aligned} \left( \frac{1 \,\mathrm {cm}}{R} \right) ^2 \sim 10^{-12}, \end{aligned}$$

assuming $R\sim 10 \,\mathrm {km}$ to be a typical neutron star radius.

The above arguments show that the bulk properties of a cold, isolated rotating relativistic star can be modeled accurately by a uniformly rotating, one-parameter perfect fluid. Effects of differential rotation and of finite temperature need only be considered during the first year (or less) after the formation of a relativistic star. Furthermore, magnetic fields, while important for high-energy phenomena in the magnetosphere and for the damping of differential rotation and oscillations, do not alter the structure of the star, unless one assumes magnetic field strengths significantly higher than typical observed values.

### Geometry of spacetime

In general relativity, the spacetime geometry of a rotating star in equilibrium can be described by a stationary and axisymmetric metric $g_{\alpha \beta }$ of the form

5 $$\begin{aligned} ds^2 = -e^{2 \nu } \, dt^2 + e^{2 \psi } (d \phi - \omega \, dt)^2 + e^{2 \mu } (dr^2+r^2 d \theta ^2), \end{aligned}$$

where $\nu$, $\psi$, $\omega$ and $\mu$ are four metric functions that depend on the coordinates *r* and $\theta$ only (see, e.g., Bardeen and Wagoner 1971). For a discussion and historical overview of other coordinate choices for axisymmetric rotating spacetimes see Gourgoulhon (2010), Friedman and Stergioulas (2013). In the exterior vacuum, it is possible to reduce the number of metric functions to three, but as long as one is interested in describing the whole spacetime (including the source-region of nonzero pressure), four different metric functions are required. It is convenient to write $e^\psi$ in the form

6 $$\begin{aligned} e^\psi =r \sin \theta B e^{-\nu }, \end{aligned}$$

where *B* is again a function of *r* and $\theta$ only (Bardeen 1973).

One arrives at the above form of the metric assuming that

1. The spacetime is *stationary* and *axisymmetric*: There exist an asymptotically timelike symmetry vector $t^\alpha$ and a rotational symmetry vector $\phi ^\alpha$.
  The spacetime is said to be *strictly* stationary if $t^\alpha$ is everywhere timelike. (Some rapidly rotating stellar models, as well as black-hole spacetimes, have *ergospheres*, regions in which $t^\alpha$ is spacelike.)
2. The Killing vectors commute,
  7 $$\begin{aligned}{}[t, \phi ]=0, \end{aligned}$$
  and there is an isometry of the spacetime that simultaneously reverses the direction of $t^\alpha$ and $\phi ^\alpha$,
  8 $$\begin{aligned} t^\alpha \rightarrow -t^\alpha ,\quad \phi ^\alpha \rightarrow - \phi ^\alpha . \end{aligned}$$
3. The spacetime is asymptotically flat, i.e., $t_at^a=-1$, $\phi _a\phi ^a=+\infty$ and $t_a\phi ^a=0$ at spatial infinity
4. The spacetime is circular (there are no meridional currents in the fluid).

If the spacetime is strictly stationary, one does not need (7) as a separate assumption: A theorem by Carter (1970) shows that $[t, \phi ]=0$. The Frobenius theorem now implies the existence of scalars *t* and $\phi$ (Kundt and Trümper 1966; Carter 1969) for which there exists a family of 2-surfaces orthogonal to $t^\alpha$ and $\phi ^\alpha$, the surfaces of constant *t* and $\phi$; and it is natural to choose as coordinates $x^0=t$ and $x^3=\phi$. In the absence of meridional currents, the 2-surfaces orthogonal to $t^\alpha$ and $\phi ^\alpha$ can be described by the remaining two coordinates $x^1$ and $x^2$ (Carter 1970). Requiring that these are Lie derived by $t^\alpha$ and $\phi ^\alpha$, we have

9 $$\begin{aligned} t^\alpha= & {} {\varvec{\partial }}_t, \end{aligned}$$

10 $$\begin{aligned} \phi ^\alpha= & {} {\varvec{\partial }}_\phi . \end{aligned}$$

With coordinates chosen in this way, the metric components are independent of *t* and $\phi$.

Because time reversal inverts the direction of rotation, the fluid is not invariant under $t\rightarrow -t$ alone, implying that $t^\alpha$ and $\phi ^\alpha$ are not orthogonal to each other. The lack of orthogonality is measured by the metric function $\omega$ that describes the *dragging of inertial frames*.

In a fluid with meridional convective currents one loses both time-reversal invariance and invariance under the simultaneous inversion $t\rightarrow -t, \phi \rightarrow -\phi$, because the inversion changes the direction of the circulation. In this case, the spacetime metric will have additional off-diagonal components (Gourgoulhon and Bonazzola 1993; Birkl et al. 2011).

A common choice for $x^1$ and $x^2$ are *quasi-isotropic coordinates*, for which $g_{r\theta }=0$ and $g_{\theta \theta }=r^2 g_{rr}$ (in spherical polar coordinates), or $g_{\varpi z}=0$ and $g_{zz}=r^2 g_{\varpi \varpi }$ (in cylindrical coordinates). In the nonrotating limit, the metric (5) reduces to the metric of a nonrotating relativistic star in *isotropic coordinates* (see Weinberg 1972 for the definition of these coordinates). In the slow rotation formalism by Hartle (1967), a different form of the metric is used, requiring $g_{\theta \theta }=g_{\phi \phi }/ \sin ^2 \theta$, which corresponds to the choice of Schwarzschild coordinates in the vacuum region.

The three metric functions $\nu$, $\psi$ and $\omega$ can be written as invariant combinations of the two Killing vectors $t^\alpha$ and $\phi ^\alpha$, through the relations

11 $$\begin{aligned} t_\alpha t^\alpha= & {} g_{tt}= -e^{2\nu } + \omega ^2 e^{2\psi }, \end{aligned}$$

12 $$\begin{aligned} \phi _\alpha \phi ^\alpha= & {} g_{\phi \phi }= e^{2\psi }, \end{aligned}$$

13 $$\begin{aligned} t_\alpha \phi ^\alpha= & {} g_{t\phi }= -\omega e^{2\psi }, \end{aligned}$$

The corresponding components of the contravariant metric are

14 $$\begin{aligned} g^{tt}= & {} \nabla _\alpha t \nabla ^\alpha t = -e^{-2\nu }, \end{aligned}$$

15 $$\begin{aligned} g^{\phi \phi }= & {} \nabla _\alpha \phi \nabla ^\alpha \phi = e^{-2\psi }-\omega ^2 e^{-2\nu }, \end{aligned}$$

16 $$\begin{aligned} g^{t\phi }= & {} \nabla _\alpha t \nabla ^\alpha \phi = -\omega e^{-2\nu }. \end{aligned}$$

The fourth metric function $\mu$ determines the conformal factor $e^{2\mu }$ that characterizes the geometry of the orthogonal 2-surfaces.

There are two main effects that distinguish a rotating relativistic star from its nonrotating counterpart: The shape of the star is flattened by centrifugal forces (an effect that first appears at second order in the rotation rate), and the local inertial frames are dragged by the rotation of the source of the gravitational field. While the former effect is also present in the Newtonian limit, the latter is a purely relativistic effect.

The study of the dragging of inertial frames in the spacetime of a rotating star is assisted by the introduction of the local Zero-Angular-Momentum-Observers (ZAMO) (Bardeen 1970, 1973). These are observers whose worldlines are normal to the $t=\,\mathrm {const.}$ hypersurfaces (also called *Eulerian* or *normal* observers in the 3+1 formalism Arnowitt et al. 2008). Then, the metric function $\omega$ is the angular velocity $d\phi /dt$ of the local ZAMO with respect to an observer at rest at infinity. Also, $e^{-\nu }$ is the time dilation factor between the proper time of the local ZAMO and coordinate time *t* (proper time at infinity) along a radial coordinate line. The metric function $\psi$ has a geometrical meaning: $e^\psi$ is the *proper circumferential radius* of a circle around the axis of symmetry.

In rapidly rotating models, an *ergosphere* can appear, where $g_{tt}>0$ (as long as we are using the Killing coordinates described above). In this region, the rotational frame-dragging is strong enough to prohibit counter-rotating time-like or null geodesics to exist, and particles can have negative energy with respect to a stationary observer at infinity. Radiation fields (scalar, electromagnetic, or gravitational) can become unstable in the ergosphere (Friedman 1978), but the associated growth time is comparable to the age of the universe (Comins and Schutz 1978).

The lowest-order asymptotic behaviour of the metric functions $\nu$ and $\omega$ is

17 $$\begin{aligned} \nu\sim & {} -{M \over r}, \end{aligned}$$

18 $$\begin{aligned} \omega\sim & {} {2J \over r^3}, \end{aligned}$$

where *M* and *J* are the total gravitational mass and angular momentum (see Sect. 2.5 for definitions). The asymptotic expansion of the dragging potential $\omega$ shows that it decays rapidly far from the star, so that its effect will be significant mainly in the vicinity of the star.

### The rotating fluid

When sources of non-isotropic stresses (such as a magnetic field or a solid state of parts of the star), viscous stresses, and heat transport are neglected in constructing an equilibrium model of a relativistic star, then the matter can be modeled as a perfect fluid, described by the stress-energy tensor

19 $$\begin{aligned} T^{\alpha \beta } = (\varepsilon +P)u^\alpha u^\beta + P g^{\alpha \beta }, \end{aligned}$$

where $u^\alpha$ is the fluid’s 4-velocity. In terms of the two Killing vectors $t^\alpha$ and $\phi ^\alpha$, the 4-velocity can be written as

20 $$\begin{aligned} u^\alpha = \frac{e^{-\nu }}{\sqrt{1-v^2}} (t^\alpha + \varOmega \phi ^\alpha ), \end{aligned}$$

where *v* is the 3-velocity of the fluid with respect to a local ZAMO, given by

21 $$\begin{aligned} v = (\varOmega -\omega )e^{\psi -\nu }, \end{aligned}$$

and $\varOmega \equiv u^\phi / u^t=d\phi / dt$ is the angular velocity of the fluid in the *coordinate frame*, which is equivalent to the *angular velocity of the fluid as seen by an observer at rest at infinity*. Stationary configurations can be differentially rotating, while uniform rotation ($\varOmega = \,\mathrm {const.}$) is a special case (see Sect. 2.5).

The covariant components of the 4-velocity take the form

22 $$\begin{aligned} u_t= & {} -\frac{e^\nu }{\sqrt{1-v^2}}(1+e^{\psi -\nu }\omega v ), \qquad u_{\phi } = \frac{e^\psi v}{\sqrt{1-v^2}}. \end{aligned}$$

Notice that the components of the 4-velocity are proportional to the *Lorentz factor* $W:=(1-v^2)^{-1/2}$.

### Equations of structure

Having specified an equation of state of the form $\varepsilon = \varepsilon (P)$, the structure of the star is determined by solving four components of Einstein’s gravitational field equation

23 $$\begin{aligned} R_{\alpha \beta } = 8 \pi \left( T_{\alpha \beta }- \frac{1}{2} g_{\alpha \beta }T \right) , \end{aligned}$$

(where $R_{\alpha \beta }$ is the Ricci tensor and $T=T_\alpha {}^\alpha$) and the equation of hydrostationary equilibrium. Setting $\zeta = \mu + \nu$, one common choice (Butterworth and Ipser 1976) for the components of the gravitational field equation are the three equations of elliptic type

24 $$\begin{aligned} \varvec{\nabla }\cdot (B \varvec{\nabla }\nu )= & {} \frac{1}{2} r^2\sin ^2\theta B^3e^{-4\nu } \varvec{\nabla }\omega \cdot \varvec{\nabla }\omega \nonumber \\&+\,4 \pi B e^{2\zeta -2\nu } \left[ \frac{(\varepsilon +P)(1+v^2)}{1-v^2} +2P \right] , \end{aligned}$$

25 $$\begin{aligned} \varvec{\nabla }\cdot (r^2\sin ^2\theta B^3e^{-4\nu }\varvec{\nabla }\omega )= & {} -16 \pi r \sin \theta B^2e^{2\zeta -4 \nu } \frac{(\varepsilon +P)v}{1-v^2}, \end{aligned}$$

26 $$\begin{aligned} \varvec{\nabla }\cdot (r \sin \theta \varvec{\nabla }B)= & {} 16 \pi r \sin \theta Be^{2\zeta -2\nu }P, \end{aligned}$$

supplemented by a first order differential equation for $\zeta$

27 $$\begin{aligned} \frac{1}{\varpi }\zeta ,_\varpi + \frac{1}{B}(B,_\varpi \zeta ,_\varpi -B,_z\zeta ,_z)= & {} \frac{1}{2\varpi ^2B}(\varpi ^2B,_\varpi ),_\varpi -\frac{1}{2B}B,_{zz}+(\nu ,_\varpi )^2 \nonumber \\&-(\nu ,_z)^2 -\frac{1}{4}\varpi ^2B^2e^{-4\nu }\left[ (\omega ,_\varpi )^2-(\omega ,_z)^2\right] . \nonumber \\&\end{aligned}$$

Above, $\varvec{\nabla }$ is the 3-dimensional derivative operator in a flat 3-space with spherical polar coordinates *r*, $\theta$, $\phi$. The remaining nonzero components of the gravitational field equation yield two more elliptic equations and one first order partial differential equation, which are consistent with the above set of four equations.

The equation of motion (*Euler equation*) follows from the projection of the conservation of the stress-energy tensor orthogonal to the 4-velocity $(\delta ^\gamma {}_\beta +u^\gamma u_\beta )\nabla _\alpha T^{\alpha \beta }=0$

28 $$\begin{aligned} \frac{\nabla _\alpha p}{(\epsilon +p)}= & {} -u^\beta \nabla _\beta u_\alpha \nonumber \\= & {} \nabla _\alpha \ln u^t -u^tu_\phi \nabla _\alpha \varOmega . \end{aligned}$$

In the $r-\theta$ subspace, one can find the following equivalent forms

29 $$\begin{aligned} \frac{\nabla p}{(\epsilon +p)}= & {} -\frac{1}{1-v^2} \left( \nabla \nu -v^2\nabla \psi +e^{\psi -\nu } v \nabla \omega \right) , \end{aligned}$$

30 $$\begin{aligned}= & {} \nabla \ln u^t - u^t u_\phi \nabla \varOmega , \end{aligned}$$

31 $$\begin{aligned}= & {} \nabla \ln u^t - \frac{l}{1-\varOmega l} \nabla \varOmega , \end{aligned}$$

32 $$\begin{aligned}= & {} -\nabla \ln (-u_t) + \frac{\varOmega }{1-\varOmega l} \nabla l, \end{aligned}$$

33 $$\begin{aligned}= & {} -\nabla \nu + \frac{1}{1-v^2} \left( v \nabla v - \frac{v^2\nabla \varOmega }{\varOmega -\omega } \right) , \end{aligned}$$

where $l:=-u_\phi /u_t$ is conserved along fluid trajectories (since $h u_t$ and $h u_\phi$ are conserved, so is their ratio and *l* is the *angular momentum per unit energy*).

For barotropes, one can arrive at a first integral of the equations of motion in the following way. Since $\epsilon =\epsilon (p)$, one can define a function

34 $$\begin{aligned} H(p) := \int _0^p \frac{dp'}{\epsilon (p')+p'}, \end{aligned}$$

so that (28) becomes

35 $$\begin{aligned} \nabla (H-\ln u^t) = -F \nabla \varOmega , \end{aligned}$$

where we have set $F:=u^t u_\phi =l/(1-l\varOmega )$. For *homentropic stars* (stars with a homogeneous entropy distribution) one obtains $H=\ln h$ (where *h* is the *specific enthalpy*) and the equation of hydrostationary equilibrium takes the form

36 $$\begin{aligned} \nabla \left( \ln \frac{h}{u^t} \right) = -F \nabla \varOmega . \end{aligned}$$

Taking the curl of (35) one finds that either

37 $$\begin{aligned} \varOmega =\mathrm{constant}, \end{aligned}$$

(*uniform rotation*), or

38 $$\begin{aligned} F=F(\varOmega ), \end{aligned}$$

in the case of *differential rotation*. In the latter case, (35) becomes

39 $$\begin{aligned} H-\ln u^t+\int ^\varOmega _{\varOmega _\mathrm{pole}} F(\varOmega ')d\varOmega ' =\nu |_\mathrm{pole}, \end{aligned}$$

where the lower limit, $\varOmega _0$ is chosen as the value of $\varOmega$ at the pole, where *H* and *v* vanish. The above *global* first integral of the hydrostationary equilibrium equations is useful in constructing numerical models of rotating stars.

For a uniformly rotating star, (39) can be written as

40 $$\begin{aligned} H-\ln u^t = \nu |_\mathrm{pole}, \end{aligned}$$

which, in the case of a homentropic star, becomes

41 $$\begin{aligned} \frac{h}{u^t} = \mathcal{E}, \end{aligned}$$

with $\mathcal{E}=\left. e^\nu \right. |_\mathrm{pole}$ constant over the star ($\mathcal E$ has the meaning of the *injection energy* (Friedman and Stergioulas 2013), the increase in a star’s mass when a unit mass of baryons is injected at a point in the star).

In the Newtonian limit $\displaystyle e^\psi = \varpi + O(\lambda ^2), \quad e^\nu = 1+O(\lambda ^2)$, so to Newtonian order we have

42 $$\begin{aligned} u^t u_\phi = v\varpi = \varpi ^2\varOmega , \end{aligned}$$

and the functional dependence of $\varOmega$ implied by Eq. (38) becomes the familiar requirement that, for a barotropic equation of state, $\varOmega$ be stratified on cylinders,

43 $$\begin{aligned} \varOmega = \varOmega (\varpi ), \end{aligned}$$

where $\varpi =r\sin (\theta )$. The Newtonian limit of the integral of motion (39) is

44 $$\begin{aligned} h_\mathrm{Newtonian} -\frac{1}{2} v^2 + \varPhi = \text{ constant }, \end{aligned}$$

where, in the Newtonian limit, $h_\mathrm{Newtonian}=h-1$ differs from the relativistic definition by the rest mass per unit rest mass.

### Rotation law and equilibrium quantities

A special case of rotation law is *uniform rotation* (uniform angular velocity in the coordinate frame), which minimizes the total mass–energy of a configuration for a given baryon number and total angular momentum (Boyer and Lindquist 1966; Hartle and Sharp 1967). In this case, the term involving $F(\varOmega )$ in (39) vanishes.

More generally, a simple, *one-parameter* choice of a differential-rotation law is

45 $$\begin{aligned} F(\varOmega )= A^2(\varOmega _{\mathrm {c}}-\varOmega ) = \frac{(\varOmega -\omega )r^2\sin ^2\theta ~e^{2(\beta -\nu )}}{1-(\varOmega -\omega )^2r^2\sin ^2\theta ~e^{2(\beta -\nu )}}, \end{aligned}$$

where *A* is a constant (Komatsu et al. 1989a, b). When $A \rightarrow \infty$, the above rotation law reduces to the uniform rotation case. In the Newtonian limit and when $A \rightarrow 0$, the rotation law becomes a so-called *j*-constant rotation law (with specific angular momentum *j*, angular momentum per unit mass, being constant in space), which satisfies the Rayleigh criterion for local dynamical stability against axisymmetric disturbances (*j* should not decrease outwards, $dj/d\varOmega <0$). The same criterion is also satisfied in the relativistic case, but with $j\rightarrow \tilde{j}=u_\phi (\varepsilon +P)/\rho$ (Komatsu et al. 1989b), where $\rho$ is the fluid rest-mass density. It should be noted that differentially rotating stars may also be subject to a shear instability that tends to suppress differential rotation (Zahn 1993).

The above rotation law is a simple choice that has proven to be computationally convenient. A new, *3-parameter* generalization of the above rotation law was recently proposed in Galeazzi et al. (2012) and is defined by

46 $$\begin{aligned} F(\varOmega )= \frac{\frac{R_0^2}{\varOmega _c^\alpha }\varOmega (\varOmega ^\alpha -\varOmega _c^\alpha )}{1-\frac{R_0^2}{\varOmega _c^\alpha }\varOmega ^2(\varOmega ^\alpha -\varOmega _c^\alpha )} \end{aligned}$$

where $\alpha ,\ R_0$ and $\varOmega _c$ are constants. The specific angular momentum corresponding to this law is

47 $$\begin{aligned} l= \frac{R_0^2}{\varOmega _c^\alpha }\varOmega (\varOmega ^\alpha -\varOmega _c^\alpha ) . \end{aligned}$$

The Newtonian limit for this law yields an angular frequency of

48 $$\begin{aligned} \varOmega = \varOmega _c\left[ 1+\left( \frac{\varpi }{R_0}\right) ^2\right] ^{\frac{1}{\alpha }}, \end{aligned}$$

thus, for $\varpi \ll R_0$, $\varOmega \sim \varOmega _c$, whereas for $\varpi \gg R_0$, $\varOmega _c\sim \varOmega (\varpi /R_0)^{2/\alpha }$. A more recent *4-parameter* family of rotation laws was proposed in (Mach and Malec 2015) mainly for accretion tori (it has not yet been applied to models of rotating neutron stars). It remains to be seen how well the above laws can match the angular velocity profiles of proto-neutron stars and remnants of binary neutron star mergers formed in numerical simulations.

**Table 1:** Equilibrium properties

Circumferential radius	$R=e^\psi$
Gravitational mass	$M=-2 \int (T_\alpha {}^\beta -{1 \over 2} \delta _\alpha ^\beta T) t^\alpha \hat{n}^\beta \, dV$
Baryon mass	$M_0 = \int \rho u_\beta \hat{n}^\beta \, dV$
Internal energy	$U = \int u u_\beta \hat{n}^\beta \, dV$
Proper mass	$M_{\mathrm {p}} = M_0 +U$
Gravitational binding energy	$W=M-M_{\mathrm {p}}-T$
Angular momentum	$J=\int T_{\alpha \beta } \phi ^\alpha \hat{n}^\beta \, dV$
Moment of inertia (uniform rotation)	$I=J / \varOmega$
Kinetic energy	$T={1 \over 2} J \varOmega$

Equilibrium quantities for rotating stars, such as gravitational mass, baryon mass, or angular momentum, for example, can be obtained as integrals over the source of the gravitational field. A list of the most important equilibrium quantities that can be computed for axisymmetric models, along with the equations that define them, is displayed in Table 1. There, $\rho$ is the *rest-mass density*, $u=\varepsilon -\rho c^2$ is the *internal energy density*, $\hat{n}^a= \nabla _at/|\nabla _bt\nabla ^bt|^{1/2}$ is the *unit normal vector* to the $t= \,\mathrm {const.}$ spacelike hypersurfaces, and $dV=\sqrt{|{}^3g|} \, d^3x$ is the proper 3-volume element (with ${}^3g$ being the determinant of the 3-metric of spacelike hypersurfaces). It should be noted that the moment of inertia cannot be computed directly as an integral quantity over the source of the gravitational field. In addition, there exists no unique generalization of the Newtonian definition of the moment of inertia in general relativity and $I=J/\varOmega$ is a common choice.

### Equations of state

#### Relativistic polytropes

Because old neutron-stars have temperatures much smaller than the Fermi energy of their constituent particles, one can ignore entropy gradients and assume a uniform specific entropy *s*. The increase in pressure and density toward the star’s center are therefore adiabatic, if one neglects the slow change in composition. That is, they are related by the first law of thermodynamics, with $ds=0$,

49 $$\begin{aligned} d\epsilon = \frac{\epsilon +p}{\rho } d\rho , \end{aligned}$$

with *p* given in terms of $\rho$ by

50 $$\begin{aligned} \frac{\rho }{p} \frac{dp}{d\rho } = \frac{\epsilon +p}{p}\frac{dp}{d \epsilon } = \varGamma _1. \end{aligned}$$

Here $\varGamma _1$ is the *adiabatic index*, the fractional change in pressure per fractional change in comoving volume, at constant entropy and composition. In an ideal degenerate Fermi gas, in the nonrelativistic and ultrarelativistic regimes, $\varGamma _1$ has the constant values 5 / 3 and 4 / 3, respectively. Except in the outer crust, neutron-star matter is far from an ideal Fermi gas, but models often assume a constant effective adiabatic index, chosen to match an average stellar compressibility. An equation of state of the form

51 $$\begin{aligned} p = K \rho ^\varGamma , \end{aligned}$$

with *K* and $\varGamma$ constants, is called *polytropic*; *K* and $\varGamma$ are the *polytropic constant* and *polytropic exponent*, respectively. The corresponding relation between $\epsilon$ and *p* follows from (49)

52 $$\begin{aligned} \epsilon = \rho + \frac{p}{\varGamma -1}. \end{aligned}$$

The polytropic exponent $\varGamma$ is commonly replaced by a *polytropic index* *N*, given by

53 $$\begin{aligned} \varGamma =1+\frac{1}{N}. \end{aligned}$$

For the above polytropic EOS, the quantity $c^{(\varGamma -2)/(\varGamma -1)} \sqrt{K^{1/(\varGamma -1)}/G}$ has units of length. In gravitational units one can thus use $K^{N/2}$ as a fundamental length scale to define dimensionless quantities. Equilibrium models are then characterized by the polytropic index *N* and their dimensionless central energy density. Equilibrium properties can be scaled to different dimensional values, using appropriate values for *K*. For $N<1.0$ ($N>1.0$) one obtains stiff (soft) models, while for $N\sim 0.5$–1.0, one obtains models whose masses and radii are roughly consistent with observed neutron-star masses and with the weak constraints on radius imposed by present observations and by candidate equations of state.

The definition (51), (52) of the relativistic polytropic EOS was introduced by Tooper (1965), to allow a polytropic exponent $\varGamma$ that coincides with the adiabatic index of a relativistic fluid with constant entropy per baryon (a homentropic fluid). A different form, $p=K\epsilon ^\varGamma$, previously also introduced by Tooper (1964), does not satisfy Eq. (49) and therefore it is not consistent with the first law of thermodynamics for a fluid with uniform entropy.

#### Hadronic equations of state

Cold matter below the nuclear saturation density, $\rho _0 = 2.7\times 10^{14}\,\mathrm {g/cm}^{3}$ (or $n_0 = 0.16\,\mathrm {fm}^{-3}$), is thought to be well understood. A derivation of a sequence of equations of state at increasing densities, beginning with the semi-empirical mass formula for nuclei, can be found in Shapiro and Teukolsky (1983) (see also Haensel et al. 2007). Another treatment, using experimental data on neutron-rich nuclei was given in Haensel and Pichon (1994). In a neutron star, matter below nuclear density forms a crust, whose outer part is a lattice of nuclei in a relativistic electron gas. At $4\times 10^{11}\,\mathrm {g/cm}^{3}$, the electron Fermi energy is high enough to induce *neutron drip*: Above this density nucleons begin leaving their nuclei to become free neutrons. The inner crust is then a two-phase equilibrium of the lattice nuclei and electrons and a gas of free neutrons. The emergence of a free-neutron phase means that the equation of state softens immediately above neutron drip: Increasing the density leads to an increase in free neutrons and to a correspondingly smaller increase in pressure. Melting of the Coulomb lattice, marking the transition from crust to a liquid core of neutrons, protons and electrons occurs between $10^{14}\,\mathrm {g/cm}^{3}$ and $\rho _0$.

A review by Heiselberg and Pandharipande (2000) describes the partly phenomenological construction of a primarily nonrelativistic many-body theory that gives the equation of state at and slightly below nuclear density. Two-nucleon interactions are matched to neutron–neutron scattering data and the experimentally determined structure of the deuteron. Parameters of the three-nucleon interaction are fixed by matching the observed energy levels of light nuclei.

Above nuclear density, however, the equation of state is still beset by substantial uncertainties. For a typical range of current candidate equations of state, values of the pressure differ by more than a factor of 5 at $2\rho _0 \sim 5\times 10^{14}$ g/cm$^3$, and by at least that much at higher densities (Haensel 2003). Although scattering experiments probe the interactions of nucleons (and quarks) at distances small compared to the radius of a nucleon, the many-body theory required to deduce the equation of state from fundamental interactions is poorly understood. Heavy ion collisions do produce collections of nucleons at supranuclear densities, but here the unknown extrapolation is from the high temperature of the experiment to the low temperature of neutron-star matter.

Observations of neutron stars provide a few additional constraints, of which, two are unambiguous and precise: The equation of state must allow a mass at least as large as $1.97\,M_{\odot }$, the largest accurately determined mass of a neutron star. (The observation by Antoniadis et al. (2013) is of a $2.01 \pm 0.04$ neutron star. There is also an observation by Demorest et al. (2010) of a $1.97 \pm 0.04\,M_{\odot }$ neutron star). The equation of state must also allow a rotational period at least as small as 1.4 ms, the period of the fastest confirmed millisecond pulsar (Hessels et al. 2006). Observations of neutron star radii are much less precise, but a large number of observations of type I X-ray bursts or transient X-ray binaries may allow for the reconstruction of the neutron star equation of state (Özel and Psaltis 2009; Özel et al. 2010; Steiner et al. 2010).

The uncertainty in the equation of state above nuclear density is dramatically seen in the array of competing alternatives for the nature of matter in neutron star cores: Cores that are dominantly neutron matter may have sharply different equations of state, depending on the presence or absence of pion or kaon condensates, of hyperons, and of droplets of strange quark matter (described below). The inner core of the most massive neutron stars may be entirely strange quark matter. Other differences in candidate equations of state arise from constructions based on relativistic and on nonrelativistic many-body theory. A classic collection of early proposed EOSs was compiled by Arnett and Bowers (1977), while reviews of many modern EOSs have been compiled by Haensel (2003) and Lattimer and Prakash (2007). Detailed descriptions and tables of several modern EOSs, especially EOSs with phase transitions, can be found in Glendenning (1997); his treatment is particularly helpful in showing how one constructs an equation of state from a relativistic field theory. The review by Heiselberg and Pandharipande (2000), in contrast, presents a more phenomenological construction of equations of state that match experimental data. Detailed theoretical derivations of equations of state are presented in the book by Haensel et al. (2007). For recent reviews on nuclear EOSs see Sagert et al. (2010), Lattimer (2012), Fischer et al. (2014), Lattimer and Prakash (2016), Oertel et al. (2017).

Candidate EOSs are supplied in the form of an energy density versus pressure table and intermediate values are interpolated. This results in some loss of accuracy because the usual interpolation methods do not preserve thermodynamic consistency. Swesty (1996) devised a cubic Hermite interpolation scheme that does preserve thermodynamical consistency and the scheme has been shown to indeed produce higher-accuracy neutron star models (Nozawa et al. 1998).

High density equations of state with pion condensation were proposed in Migdal (1971) and Sawyer and Scalapino (1972) (see also Kunihiro et al. 1993). Beyond nuclear density, the electron chemical potential could exceed the rest mass of $\pi ^-$ (139 MeV) by a margin large enough to overcome a pion-neutron repulsion and thus allow a condensate of zero-momentum pions. The critical density is thought to be $2\rho _0$ or higher, but the uncertainty is greater than a factor of 2; and a condensate with both $\pi ^0$ and $\pi ^-$ has also been suggested. Because the s-wave kaon-neutron interaction is attractive, kaon condensation may also occur, despite the higher kaon mass, a possibility suggested in Kaplan and Nelson (1986) (for discussions with differing viewpoints see Brown and Bethe 1994; Pandharipande et al. 1995; Heiselberg and Pandharipande 2000). Pion and kaon condensates lead to significant softening of the equation of state.

As initially suggested in Ambartsumyan and Saakyan (1960), when the Fermi energy of the degenerate neutrons exceeds the mass of a $\varLambda$ or $\varSigma$, weak interactions convert neutrons to these hyperons: Examples are $2n\leftrightarrow p+\varSigma ^-$, $n+p^+\rightarrow p^++\varLambda$. Reviews and further references can be found in Glendenning (1997), Balberg and Gal (1997), Prakash et al. (1997), and more recent work, spurred by the *r*-mode instability (see Sect. 4.5.3), is reported in Lindblom and Owen 2002; Haensel et al. 2002; Lackey et al. 2006. The critical density above which hyperons appear is estimated at 2 or 3 times nuclear density. Above that density, the presence of copious hyperons can significantly soften the equation of state. Because a softer core equation of state can support less mass against collapse, the larger the observed maximum mass, the less likely that neutron stars have cores with hyperons (or with pion or kaon condensates). In particular, a measured mass of $1.97\pm 0.04\,M_{\odot }$ for the pulsar PSR J1614-2230 with a white dwarf companion (Demorest et al. 2010) limits the equation of state parameter space (Read et al. 2009), ruling out several candidate equations of states with hyperons (Özel et al. 2010). Whether a hyperon core is consistent with a mass this large remains an open question (Stone et al. 2010).

A new hadron-quark hybrid equation of state was recently introduced by Benić et al. (2015) (see also Bejger et al. 2016 for potential observational signatures of these objects). The quark matter description is based on a quantum chromodynamics approach, while the hadronic matter is modeled by means of a relativistic mean-field method with an excluded volume correction at supranuclear densities to treat the finite size of the nucleons. The excluded volume correction in conjunction with the quark repulsive interactions, result in a first-order phase transition, which leads to a new family of compact stars in a mass-radius relationship plot whose masses can exceed the $2\,M_{\odot }$ limit that is set by observations. These new stars are termed “twin” stars. The twin star phenomenon was predicted a long time ago by Gerlach (1968) (see also Kampfer 1981; Schertler et al. 2000; Glendenning and Kettner 2000). Twin stars consist of a quark core with a shell made of hadrons and a first-order phase transition at their interface. Recently, rotating twin star solutions were constructed by Haensel et al. (2016).

#### Strange quark equations of state

Before a density of about $6\rho _0$ is reached, lattice QCD calculations indicate a phase transition from quarks confined to nucleons (or hyperons) to a collection of free quarks (and gluons). Heavy ion collisions at CERN and RHIC show evidence of the formation of such a quark-gluon plasma. A density for the phase transition higher than that needed for strange quarks in hyperons is similarly high enough to give a mixture of up, down and strange quarks in quark matter, and the expected strangeness per unit baryon number is $\simeq -1$. If densities become high enough for a phase transition to quark matter to occur, neutron-star cores may contain a transition region with a mixed phase of quark droplets in neutron matter (Glendenning 1997).

Bodmer (1971) and, later, Witten (1984) pointed out that experimental data do not rule out the possibility that the ground state of matter at zero pressure and large baryon number is not iron but strange quark matter. If this is the case, all “neutron stars” may be strange quark stars, a lower density version of the quark-gluon plasma, again with roughly equal numbers of up, down and strange quarks, together with electrons to give overall charge neutrality (Bodmer 1971; Farhi and Jaffe 1984). The first extensive study of strange quark star properties is due to Witten (1984) (but, see also Ipser et al. 1975; Brecher and Caporaso 1976), while hybrid stars that have a mixed-phase region of quark and hadronic matter, have also been studied extensively (see, e.g., the review by Glendenning 1997).

The strange quark matter equation of state can be represented by the following linear relation between pressure and energy density

54 $$\begin{aligned} p=a(\epsilon -\epsilon _0), \end{aligned}$$

where $\epsilon _0$ is the energy density at the surface of a bare strange star (neglecting a possible thin crust of normal matter). The MIT bag model of strange quark matter involves three parameters, the bag constant, $\mathcal{B}=\epsilon _0/4$, the mass of the strange quark, $m_s$, and the QCD coupling constant, $\alpha _c$. The constant *a* in (54) is equal to 1 / 3 if one neglects the mass of the strange quark, while it takes the value of $a=0.289$ for $m_s=250\,\mathrm {MeV}$. When measured in units of $\mathcal{B}_{60}=\mathcal{B}/(60\,\mathrm {MeV\ fm}^{-3})$, the constant $\mathcal{B}$ is restricted to be in the range

55 $$\begin{aligned} 0.9821<\mathcal{B}_{60}<1.525, \end{aligned}$$

assuming $m_s=0$. The lower limit is set by the requirement of stability of neutrons with respect to a spontaneous fusion into strangelets, while the upper limit is determined by the energy per baryon of ${}^{56}$Fe at zero pressure (930.4 MeV). For other values of $m_s$ the above limits are modified somewhat (see also Dey et al. 1998; Gondek-Rosińska et al. 2000 for other attempts to describe deconfined strange quark matter).

### Numerical schemes

All available methods for solving the system of equations describing the equilibrium of rotating relativistic stars are numerical, as no self-consistent solution for both the interior and exterior spacetime in an algebraic closed form has been found. The first numerical solutions were obtained by Wilson (1972) and by Bonazzola and Schneider (1974). In the following, we give a description of several available numerical methods and their various implementations (codes) and extensions.

#### Hartle

To order $\mathcal{O}(\varOmega ^2)$, the structure of a star changes only by quadrupole terms and the equilibrium equations become a set of ordinary differential equations. Hartle’s (1967; 1968) method computes rotating stars in this slow rotation approximation, and a review of slowly rotating models has been compiled by Datta (1988). Weber and Glendenning (1991) and Weber et al. (1991) also implement Hartle’s formalism to explore the rotational properties of four new EOSs.

Weber and Glendenning (1992) improve on Hartle’s formalism in order to obtain a more accurate estimate of the angular velocity at the mass-shedding limit, but their models still show large discrepancies compared to corresponding models computed without the slow rotation approximation (Salgado et al. 1994a). Thus, Hartle’s formalism is appropriate for typical pulsar (and most millisecond pulsar) rotational periods, but it is not the method of choice for computing models of rapidly rotating relativistic stars near the mass-shedding limit. An extension of Hartle’s scheme to 3rd order was presented by Benhar et al. (2005).

#### Butterworth and Ipser (BI)

The BI scheme (Butterworth and Ipser 1976) solves the four field equations following a Newton–Raphson-like linearization and iteration procedure. One starts with a nonrotating model and increases the angular velocity in small steps, treating a new rotating model as a linear perturbation of the previously computed rotating model. Each linearized field equation is discretized and the resulting linear system is solved. The four field equations and the hydrostationary equilibrium equation are solved separately and iteratively until convergence is achieved.

Space is truncated at a finite distance from the star and the boundary conditions there are imposed by expanding the metric potentials in powers of 1 / *r*. Angular derivatives are approximated by high-accuracy formulae and models with density discontinuities are treated specially at the surface. An equilibrium model is specified by fixing its rest mass and angular velocity.

The original BI code was used to construct uniform density models and polytropic models (Butterworth and Ipser 1976; Butterworth 1976). Friedman et al. (1986, 1989) (FIP) extend the BI code to obtain a large number of rapidly rotating models based on a variety of realistic EOSs. Lattimer et al. (1990) used a code that was also based on the BI scheme to construct rotating stars using “exotic” and schematic EOSs, including pion or kaon condensation and strange quark matter.

#### Komatsu, Eriguchi, and Hachisu (KEH)

In the KEH scheme (Komatsu et al. 1989a, b), the same set of field equations as in BI is used, but the three elliptic-type field equations are converted into integral equations using appropriate Green’s functions. The boundary conditions at large distance from the star are thus incorporated into the integral equations, but the region of integration is truncated at a finite distance from the star. The fourth field equation is an ordinary first order differential equation. The field equations and the equation of hydrostationary equilibrium are solved iteratively, fixing the maximum energy density and the ratio of the polar radius to the equatorial radius, until convergence is achieved. In Komatsu et al. (1989a, b) and Eriguchi et al. (1994), the original KEH code is used to construct uniformly and differentially rotating stars for both polytropic and realistic EOSs.

Cook, Shapiro, and Teukolsky (CST) improve on the KEH scheme by introducing a new radial variable that maps the semi-infinite region $[0,\infty )$ to the closed region [0, 1]. In this way, the region of integration is not truncated and the model converges to a higher accuracy. Details of the code are presented in Cook et al. (1992) and polytropic and realistic models are computed in Cook et al. (1994b) and Cook et al. (1994a).

Stergioulas and Friedman (SF) implement their own KEH code following the CST scheme. They improve on the accuracy of the code by a special treatment of the second order radial derivative that appears in the source term of the first order differential equation for one of the metric functions. This derivative was introducing a numerical error of 1–2% in the bulk properties of the most rapidly rotating stars computed in the original implementation of the KEH scheme. The SF code is presented in Stergioulas and Friedman (1995) and in Stergioulas (1996). It is available as a public domain code, named RNS, and can be downloaded from Stergioulas (1999).

A generalized KEH-type numerical code, suitable also for binary compact objects, was presented by Uryū and Tsokaros (2012); Uryū et al. (2012). The COCAL code has been applied to black hole models, and was recently extended to neutron star models, either in isolation (Uryū et al. 2014, 2016b) or in binaries (Tsokaros et al. 2015). The extended COCAL code allows for the generation of (quasi)equilribrium, magnetized, and rotating axisymmetric neutron star models, as well as quasiequilibrium corotational, irrotational, and spinning neutron star binaries. The code can also build models of isolated, quasiequilibrium, triaxial neutron stars (Uryū et al. 2016b, a)—a generalization of Jacobi ellipsoids in general relativity. Such configurations were recently studied dynamically in Tsokaros et al. (2017) and were found to be dynamically stable, though their secular stability still remains an open question.

#### Bonazzola et al. (BGSM)

In the BGSM scheme (Bonazzola et al. 1993), the field equations are derived in the 3$+$1 formulation. All four chosen equations that describe the gravitational field are of elliptic type. This avoids the problem with the second order radial derivative in the source term of the ODE used in BI and KEH. The equations are solved using a spectral method, i.e., all functions are expanded in terms of trigonometric functions in both the angular and radial directions and a Fast Fourier Transform (FFT) is used to obtain coefficients. Outside the star a redefined radial variable is used, which maps infinity to a finite distance.

In Salgado et al. (1994a, b), the code is used to construct a large number of models based on recent EOSs. The accuracy of the computed models is estimated using two general relativistic virial identities, valid for general asymptotically flat spacetimes (Gourgoulhon and Bonazzola 1994; Bonazzola and Gourgoulhon 1994) (see Sect. 2.7.8).

While the field equations used in the BI and KEH schemes assume a perfect fluid, isotropic stress-energy tensor, the BGSM formulation makes no assumption about the isotropy of $T_{ab}$. Thus, the BGSM code can compute stars with a magnetic field, a solid crust, or a solid interior, and it can also be used to construct rotating boson stars.

#### LORENE/rotstar

Bonazzola et al. (1998) have improved the BGSM spectral method by allowing for several domains of integration. One of the domain boundaries is chosen to coincide with the surface of the star and a regularization procedure is introduced for the divergent derivatives at the surface (that appear in the density field when stiff equations of state are used). This allows models to be computed that are nearly free of Gibbs phenomena at the surface. The same method is also suitable for constructing quasi-stationary models of binary neutron stars. The new method has been used in Gourgoulhon et al. (1999) for computing models of rapidly rotating strange stars and it has also been used in 3D computations of the onset of the viscosity-driven instability to bar-mode formation (Gondek-Rosińska and Gourgoulhon 2002).

The LORENE library is available as public domain software (Gourgoulhon et al. 2008). It has also been used to construct equilibrium models of rotating stars as initial data for a fully constraint evolution scheme in the Dirac gauge and with maximal slicing (Lin and Novak 2006).

#### Ansorg et al. (AKM)

Another multi-domain spectral scheme was introduced in Ansorg et al. (2002, 2003). The scheme can use several domains inside the star, one for each possible phase transition in the equation of state. Surface-adapted coordinates are used and approximated by a two-dimensional Chebyshev-expansion. Transition conditions are satisfied at the boundary of each domain, and the field and fluid equations are solved as a free boundary value problem by a full Newton–Raphson method, starting from an initial guess. The field-equation components are simplified by using a corotating reference frame. Applying this new method to the computation of rapidly rotating homogeneous relativistic stars, Ansorg et al. achieve near machine accuracy, when about 24 expansion coefficients are used (see Sect. 2.7.9). For configurations near the mass-shedding limit the relative error increases to about $10^{-5}$, even with 24 expansion coefficients, due to the low differentiability of the solution at the surface. The AKM code has been used in systematic studies of uniformly rotating homogeneous stars (Schöbel and Ansorg 2003) and differentially rotating polytropes (Ansorg et al. 2009). A detailed description of the numerical method and a review of the results is given in Meinel et al. (2008).

A public domain library which implements spectral methods for solving nonlinear systems of partial differential equations with a Newton–Rapshon method was presented by Grandclément (2010, 2009). The KADATH library could be used to construct equilibrium models of rotating relativistic stars in a similar manner as in Ansorg et al. (2002, 2003).

#### IWM-CFC approximation

The spatial conformal flatness condition (IWM-CFC) (Isenberg 2008; Wilson et al. 1996) is an approximation, in which the spatial part of the metric is assumed to be conformally flat. Computationally, one has to solve one equation less than in full GR, for isolated stars. The accuracy of this approximation has been tested for uniformly rotating stars by Cook et al. (1996) and it is satisfactory for many applications. Nonaxisymmetric configurations in the IWM-CFC approximation were obtained in Huang et al. (2008). The accuracy of the IWM-CFC approximation was also tested for initial data of strongly differentially rotating neutron star models (Iosif and Stergioulas 2013).

The conformal flatness approach has been extended to avoid non-uniqueness issues arising in the solution of the standard CFC equations by Cordero-Carrión et al. (2009). This method has also been termed the “extended CFC” approach (Bucciantini and Del Zanna 2011) and has been applied to the construction of general relativistic magnetodydrodynamic equilibria (Pili et al. 2014, 2017).

#### The virial identities

Equilibrium configurations in Newtonian gravity satisfy the well-known virial relation (assuming a polytropic equation of state)

56 $$\begin{aligned} 2T+3(\varGamma -1)U+W=0. \end{aligned}$$

This can be used to check the accuracy of computed numerical solutions. In general relativity, a different identity, valid for a stationary and axisymmetric spacetime, was found in Bonazzola (1973). More recently, two relativistic virial identities, valid for general asymptotically flat spacetimes, have been derived by Gourgoulhon and Bonazzola (1994); Bonazzola and Gourgoulhon (1994). The 3-dimensional virial identity (GRV3) (Gourgoulhon and Bonazzola 1994) is the extension of the Newtonian virial identity (56) to general relativity. The 2-dimensional (GRV2) (Bonazzola and Gourgoulhon 1994) virial identity is the generalization of the identity found in Bonazzola (1973) (for axisymmetric spacetimes) to general asymptotically flat spacetimes. In Bonazzola and Gourgoulhon (1994), the Newtonian limit of GRV2, in axisymmetry, is also derived. Previously, such a Newtonian identity had only been known for spherical configurations (Chandrasekhar 1939).

The two virial identities are an important tool for checking the accuracy of numerical models and have been repeatedly used by several authors (see, e.g., Bonazzola et al. 1993; Salgado et al. 1994a, b; Nozawa et al. 1998; Ansorg et al. 2002).

#### Direct comparison of numerical codes

The accuracy of the above numerical codes can be estimated, if one constructs exactly the same models with different codes and compares them directly. The first such comparison of rapidly rotating models constructed with the FIP and SF codes is presented in Stergioulas and Friedman (1995). Rapidly rotating models constructed with several EOSs agree to 0.1–1.2% in the masses and radii and to better than 2% in any other quantity that was compared (angular velocity and momentum, central values of metric functions, etc.). This is a very satisfactory agreement, considering that the BI code was using relatively few grid points, due to limitations of computing power at the time of its implementation.

**Table 2:** Detailed comparison of the accuracy of different numerical codes in computing a rapidly rotating, uniform density model

	AKM	Lorene/rotstar	SF ($260 \times 400$)	SF ($70 \times 200$)	BGSM	KEH
$\bar{p}_{\mathrm {c}}$	1.0					
$r_{\mathrm {p}}/r_{\mathrm {e}}$	0.7				$1 \times 10^{-3}$	
$\bar{\varOmega }$	1.41170848318	$9 \times 10^{-6}$	$3 \times 10^{-4}$	$3 \times 10^{-3}$	$1 \times 10^{-2}$	$1 \times 10^{-2}$
$\bar{M}$	0.135798178809	$2 \times 10^{-4}$	$2 \times 10^{-5}$	$2 \times 10^{-3}$	$9 \times 10^{-3}$	$2 \times 10^{-2}$
$\bar{M}_0$	0.186338658186	$2 \times 10^{-4}$	$2 \times 10^{-4}$	$3 \times 10^{-3}$	$1 \times 10^{-2}$	$2 \times 10^{-3}$
$\bar{R}_{\mathrm {circ}}$	0.345476187602	$5 \times 10^{-5}$	$3 \times 10^{-5}$	$5 \times 10^{-4}$	$3 \times 10^{-3}$	$1 \times 10^{-3}$
$\bar{J}$	0.0140585992949	$2 \times 10^{-5}$	$4 \times 10^{-4}$	$5 \times 10^{-4}$	$2 \times 10^{-2}$	$2 \times 10^{-2}$
$Z_{\mathrm {p}}$	1.70735395213	$1 \times 10^{-5}$	$4 \times 10^{-5}$	$1 \times 10^{-4}$	$2 \times 10^{-2}$	$6 \times 10^{-2}$
$Z_{\mathrm {eq}}^{\mathrm {f}}$	−0.162534082217	$2 \times 10^{-4}$	$2 \times 10^{-3}$	$2 \times 10^{-2}$	$4 \times 10^{-2}$	$2 \times 10^{-2}$
$Z_{\mathrm {eq}}^{\mathrm {b}}$	11.3539142587	$7 \times 10^{-6}$	$7 \times 10^{-5}$	$1 \times 10^{-3}$	$8 \times 10^{-2}$	$2 \times 10^{-1}$
$|\mathrm {GRV3}|$	$4 \times 10^{-13}$	$3 \times 10^{-6}$	$3 \times 10^{-5}$	$1 \times 10^{-3}$	$4 \times 10^{-3}$	$1 \times 10^{-1}$

The absolute value of the relative error in each quantity, compared to the AKM code, is shown for the numerical codes Lorene/rotstar, SF (at two resolutions), BGSM, and KEH (see text). The resolutions for the SF code are (angular $\times$ radial) grid points. See Nozawa et al. (1998) for the definition of the various equilibrium quantities

In Stergioulas and Friedman (1995), it is also shown that a large discrepancy between certain rapidly rotating models (constructed with the FIP and KEH codes) that was reported by Eriguchi et al. (1994), resulted from the fact that Eriguchi et al. and FIP used different versions of a tabulated EOS.

Nozawa et al. (1998) have completed an extensive direct comparison of the BGSM, SF, and the original KEH codes, using a large number of models and equations of state. More than twenty different quantities for each model are compared and the relative differences range from $10^{-3}$ to $10^{-4}$ or better, for smooth equations of state. The agreement is also excellent for soft polytropes. These checks show that all three codes are correct and successfully compute the desired models to an accuracy that depends on the number of grid points used to represent the spacetime.

If one makes the extreme assumption of uniform density, the agreement is at the level of $10^{-2}$. In the BGSM code this is due to the fact that the spectral expansion in terms of trigonometric functions cannot accurately represent functions with discontinuous first order derivatives at the surface of the star. In the KEH and SF codes, the three-point finite-difference formulae cannot accurately represent derivatives across the discontinuous surface of the star.

The accuracy of the three codes is also estimated by the use of the two virial identities. Overall, the BGSM and SF codes show a better and more consistent agreement than the KEH code with BGSM or SF. This is largely due to the fact that the KEH code does not integrate over the whole spacetime but within a finite region around the star, which introduces some error in the computed models.

A direct comparison of different codes is also presented by Ansorg et al. (2002). Their multi-domain spectral code is compared to the BGSM, KEH, and SF codes for a particular uniform density model of a rapidly rotating relativistic star. An extension of the detailed comparison in Ansorg et al. (2002), which includes results obtained by the LORENE/rotstar code in Gondek-Rosińska and Gourgoulhon (2002) and by the SF code with higher resolution than the resolution used in Nozawa et al. (1998), is shown in Table 2. The comparison confirms that the virial identity GRV3 is a good indicator of the accuracy of each code. For the particular model in Table 2, the AKM code achieves nearly double-precision accuracy, while the Lorene/rotstar code has a typical relative accuracy of $2 \times 10^{-4}$–$7\times 10^{-6}$ in various quantities. The SF code at high resolution comes close to the accuracy of the Lorene/rotstar code for this model. Lower accuracy is obtained with the SF, BGSM, and KEH codes at the resolutions used in Nozawa et al. (1998).

The AKM code converges to machine accuracy when a large number of about 24 expansion coefficients are used at a high computational cost. With significantly fewer expansion coefficients (and comparable computational cost to the SF code at high resolution) the achieved accuracy is comparable to the accuracy of the LORENE/rotstar and SF codes. Moreover, at the mass-shedding limit, the accuracy of the AKM code reduces to about 5 digits (which is still highly accurate, of course), even with 24 expansion coefficients, due to the nonanalytic behaviour of the solution at the surface. Nevertheless, the AKM method represents a great achievement, as it is the first method to converge to machine accuracy when computing rapidly rotating stars in general relativity.

*Going further*   A review of spectral methods in numerical relativity can be found in Grandclément and Novak (2009). Pseudo-Newtonian models of axisymmetric, rotating relativistic stars are treated in Kim et al. (2009), while a formulation for nonaxisymmetric, uniformly rotating equilibrium configurations in the second post-Newtonian approximation is presented in Asada and Shibata (1996). Slowly-rotating models of white dwarfs in general relativity are presented in Boshkayev et al. (2013). The validity of the slow-rotation approximation is examined in Berti et al. (2005). A minimal-surface scheme was presented in Neugebauer and Herold (1992). The convergence properties iterative self-consistent-field methods when applied to stellar equilibria are investigated in Price et al. (2009).

### Analytic approximations to the exterior spacetime

The exterior metric of a rapidly rotating neutron star differs considerably from the Kerr metric. The two metrics agree only to lowest order in the rotational velocity (Hartle and Thorne 1969). At higher order, the multipole moments of the gravitational field created by a rapidly rotating compact star are different from the multipole moments of the Kerr field. There have been many attempts in the past to find analytic solutions to the Einstein equations in the stationary, axisymmetric case, that could describe a rapidly rotating neutron star.

In the vacuum region surrounding a stationary and axisymmetric star, the spacetime only depends on three metric functions (while four metric functions are needed for the interior). The most general form of the metric was given by Papapetrou (1953)

57 $$\begin{aligned} ds^2=-f(dt-\omega d\phi )^2+f^{-1}\left\{ e^{2\gamma } (d\tilde{\varpi }^2+d\tilde{z}^2)+\tilde{\varpi }^2d\phi ^2 \right\} . \end{aligned}$$

Here *f*, $\omega$ and $\gamma$ are functions of the quasi-cylindrical Weyl–Lewis–Papapetrou coordinates $(\tilde{\varpi },~\tilde{z})$. Starting from this metric, one can write the vacuum Einstein–Maxwell equations as two equations for two complex potentials $\mathcal{E}$ and $\varPhi$, following a procedure due to Ernst Ernst (1968a, b). Once the potentials are known, the metric can be reconstructed. Sibgatullin and Queen (1991) devised a powerful procedure for reducing the solution of the Ernst equations to simple integral equations. The exact solutions are generated as a series expansion, in terms of the physical multipole moments of the spacetime, by choosing the values of the Ernst potentials on the symmetry axis.

An interesting exact vacuum solution, given in a closed, algebraic form, was found by Manko et al. (2000a, b). For non-magnetized sources of zero net charge, it reduces to a 3-parameter solution, involving the gravitational mass, *M*, the specific angular momentum, $a=J/M$, and a third parameter, *b*, related to the quadrupole moment of the source. The Ernst potential $\mathcal E$ on the symmetry axis is

58 $$\begin{aligned} e(z)={(z-M-ia)(z+ib)+d-\delta -ab \over (z+M-ia)(z+ib)+d-\delta -ab}, \end{aligned}$$

where

59 $$\begin{aligned} \delta= & {} {-M^2b^2\over M^2-(a-b)^2}, \end{aligned}$$

60 $$\begin{aligned} d= & {} {1\over 4}\left[ M^2-(a-b)^2\right] . \end{aligned}$$

Since *a* and *b* are independent parameters, setting *a* equal to zero does not automatically imply a vanishing quadrupole moment. Instead, the nonrotating solution ($a=0$) has a quadrupole moment equal to

61 $$\begin{aligned} Q(a=0)=-{M \over 4} {\left( M^2+b^2 \right) ^2 \over \left( M^2-b^2 \right) }, \end{aligned}$$

and there exists no real value of the parameter *b* for which the quadrupole moment vanishes for a nonrotating star. Hence, the 3-parameter solution by Manko et al. does not reduce continuously to the Schwarzschild solution as the rotation vanishes and is not suitable for describing slowly rotating stars.

For rapidly rotating models, when the quadrupole deformation induced by rotation roughly exceeds the minimum nonvanishing oblate quadrupole deformation of the solution in the absence of rotation, the 3-parameter solution by Manko et al. is still relevant. A matching of the vacuum exterior solution to numerically-constructed interior solutions of rapidly rotating stars (by identifying three multipole moments) was presented by Berti and Stergioulas (2004). For a wide range of candidate EOSs, the critical rotation rate $\varOmega _\mathrm{crit}/\varOmega _\mathrm{K}$ above which the Manko et al. 3-parameter solution is relevant, ranges from $\sim 0.4$ to $\sim 0.7$ for sequences of models with $M=1.4\,M_{\odot }$, with the lower ratio corresponding to the stiffest EOS. For the maximum-mass sequence the ratio is $\sim 0.9$, nearly independent of the EOS. In Manko et al. (2000a), the quadrupole moment was also used for matching the exact vacuum solution to numerical interior solutions, but only along a different solution branch which is not a good approximation to rotating stars.

A more versatile exact exterior vacuum solution found by Manko et al. (1995) involves (in the case of vanishing charge and magnetic field) four parameters, which can be directly related to the four lowest-order multipole moments of a source (mass, angular momentum, quadrupole moment and current octupole moment). The advantage of the above solution is that its four parameters are introduced linearly in the first moment it appears. For this reason, one can always match the exact solution to a numerical solution by identifying the four lowest-order multipole moments. Therefore, the 4-parameter Manko et al. (1995) solution is relevant for studying rotating relativistic stars at any rotation rate. Pappas (2009) compared the two Manko et al. solutions to numerical solutions of rapidly rotating relativistic stars, finding good agreement. In Pappas et al. (2013), a more detailed comparison is shown, using a corrected expression for the numerical computation of the quadrupole moment. Manko and Ruiz (2016a) express the Manko et al. 4-parameter solution explicitly in terms of only three potentials, and compare the multipole structure of the solution with physically realistic numerical models of Berti and Stergioulas (2004).

Another exact exterior solution (that is related to the 4-parameter Manko et al. solution) was presented by Pachón et al. (2006) and was applied to relativistic precession and oscillation frequencies of test particles around rotating compact stars. Furthermore, an exact vacuum solution (constructed via Bäcklund transformations), that can be matched to numerically constructed solutions with an arbitrary number of constants, was presented by Teichmüller et al. (2011), who found very good agreement with numerical solutions even for a small number of parameters.

A very recent analytic solution for the exterior spacetime is provided by Pappas (2017). The metric is constructed by adopting the Ernst formulation, it is written as an expansion in Weyl–Papapetrou coordinates and has 3 free parameters—multipole moments of the NS. The metric compares favourably with numerically computed general relativistic neutron star spacetimes. An extension of the approximate metric to scalar–tensor theories with massless fields is also provided.

### Properties of equilibrium models

#### Bulk properties and sequences of equilibrium models

Neutron star models constructed with various realistic EOSs have considerably different bulk properties, due to the large uncertainties in the equation of state at high densities. Very compressible (soft) EOSs produce models with small maximum mass, small radius, and large rotation rate. On the other hand, less compressible (stiff) EOSs produce models with a large maximum mass, large radius, and low rotation rate. The sensitivity of the maximum mass to the compressibility of the neutron-star core is responsible for the strongest astrophysical constraint on the equation of state of cold matter above nuclear density. With the mass measurement of $1.97 \pm 0.04\,M_{\odot }$ for PSR J1614-2230 (Demorest et al. 2010) and of $2.01 \pm 0.04$ for PSR J0348+0432 (Antoniadis et al. 2013), several candidate EOSs that yielded models with maximum masses of nonrotating stars below this limit are ruled out, but the remaining range of candidate EOSs is still large, yielding compact objects with substantially different properties.

Not all properties of the maximum mass models between proposed EOSs differ considerably, at least not within groups of similar EOSs. For example, most realistic hadronic EOSs predict a maximum mass model with a ratio of rotational to gravitational energy *T* / |*W*| of $0.11 \pm 0.02$, a dimensionless angular momentum $cJ/GM^2$ of $0.64 \pm 0.06$, and an eccentricity of $0.66 \pm 0.04$ (Friedman and Ipser 1992). Hence, within the set of realistic hadronic EOSs, some properties are directly related to the stiffness of the EOS while other properties are rather insensitive to stiffness. On the other hand, if one considers strange quark EOSs, then for the maximum mass model, *T* / |*W*| can become more than 60% larger than for hadronic EOSs.

**Fig. 1:**
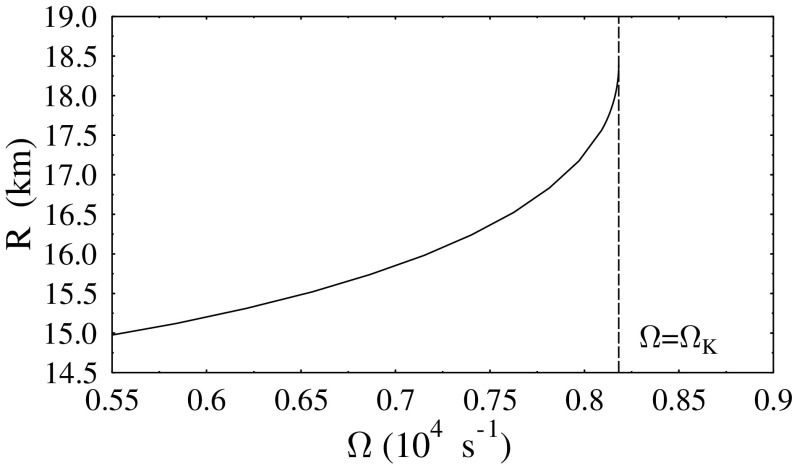
The radius *R* of a uniformly rotating star increases sharply as the Kepler (mass-shedding) limit ($\varOmega =\varOmega _K$) is approached. The particular sequence of models shown here has a constant central energy density of $\epsilon _c = 1.21 \times 10^{15}\,\mathrm {g\ cm}^{-3}$ and was constructed with EOS L. (Image reproduced with permission from Stergioulas and Friedman 1995, copyright by AAS)

**Fig. 2:**
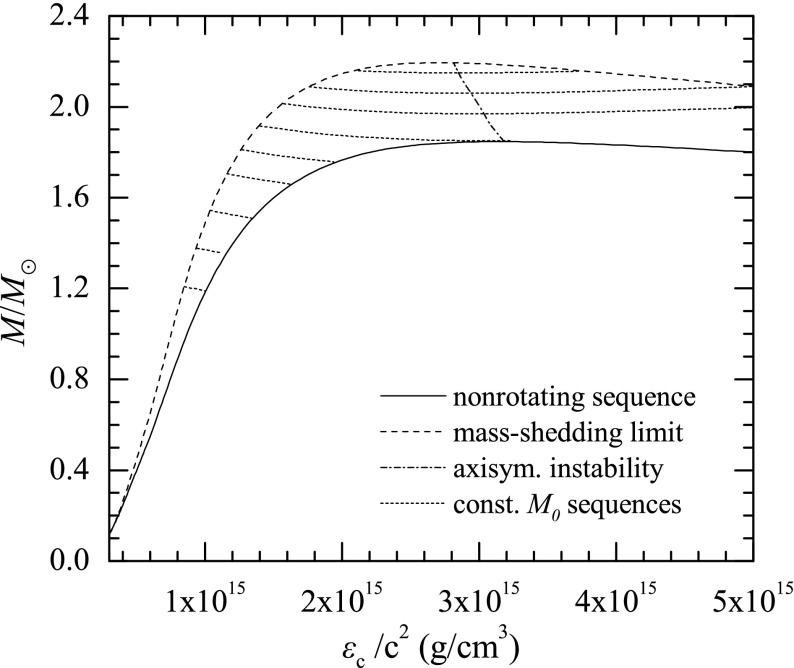
Representative sequences of rotating stars with fixed baryon mass, for EOS WFF3 (Wiringa et al. 1988). Above a rest mass of $M_0=2.17\,M_{\odot }$ only supramassive stars exist, which reach the axisymmetric instability limit when spun down. The onset of axisymmetric instability approximately coincides with the minima of the constant rest mass sequences. (Image reproduced with permission from Friedman and Stergioulas 2013, copyright by the authors)

Compared to nonrotating stars, the effect of rotation is to increase the equatorial radius of the star and also to increase the mass that can be sustained at a given central energy density. As a result, the mass of the maximum-mass rigidly rotating model is roughly 15–20% higher than the mass of the maximum mass nonrotating model (Morrison et al. 2004), for typical realistic hadronic EOSs. The corresponding increase in radius is 30–40%. Figure 1 shows an example of a sequence of uniformly rotating equilibrium models with fixed central energy density,^1^ constructed with EOS L (Pandharipande and Smith 1975; Pandharipande et al. 1976). Near the Kepler (mass-shedding) limit ($\varOmega =\varOmega _K$), the radius increases sharply. This leads to the appearance of a cusp in the equatorial plane. The effect of rotation in increasing the mass and radius becomes more pronounced in the case of strange quark EOSs (see Sect. 2.9.8).

For a given zero-temperature EOS, the uniformly rotating equilibrium models form a two-dimensional surface in the three-dimensional space of central energy density, gravitational mass, and angular momentum (Stergioulas and Friedman 1995). The surface is limited by the nonrotating models and by the models rotating at the mass-shedding (Kepler) limit. Cook et al. (1992, 1994b, 1994a) have shown that the model with maximum angular velocity does not coincide with the maximum mass model, but is generally very close to it in central density and mass. Stergioulas and Friedman (1995) showed that the maximum angular velocity and maximum baryon mass equilibrium models are also distinct. The distinction becomes significant in the case where the EOS has a large phase transition near the central density of the maximum mass model; otherwise the models of maximum mass, baryon mass, angular velocity, and angular momentum can be considered to coincide for most purposes.

In the two-dimensional parameter space of uniformly rotating models one can construct different one-dimensional sequences, depending on which quantity is held fixed. Examples are sequences of constant central energy density, constant angular momentum or constant rest mass. Figure 2 displays a representative sample of fixed rest mass sequences for EOS WFF3 (Wiringa et al. 1988) in a mass versus central energy density graph, where the sequence of nonrotating models and the sequence of models at the mass-shedding limit are also shown.^2^ The rest mass of the maximum-mass nonrotating model is $2.17\, M_{\odot }$. Below this value, all fixed rest mass sequences have a nonrotating member. Along such a sequence, the gravitational mass increases somewhat, since it also includes the rotational kinetic energy. Above $M_0=2.17\,M_{\odot }$ none of the fixed rest mass sequences have a nonrotating member. Instead, the sequences terminate at the *axisymmetric instability limit* (see Sect. 4.3.1). The onset of the instability occurs just prior to the minimum of each fixed rest mass sequence, and models to the right of the instability line are unstable.

Models with $M_0>2.17\,M_{\odot }$ have masses larger than the maximum-mass nonrotating model and are called *supramassive* (Cook et al. 1992). A millisecond pulsar spun up by accretion can become supramassive, in which case it would subsequently spin down along a sequence with approximately fixed rest mass, finally reaching the axisymmetric instability limit and collapsing to a black hole. Some relativistic stars could also be born supramassive or become so as the result of a binary merger; here, however, the star would be initially differentially rotating, and collapse would be triggered by a combination of spin-down and by viscosity (or magnetic-field braking) driving the star to uniform rotation. The *maximum mass of differentially rotating* supramassive neutron stars can be significantly larger than in the case of uniform rotation (Lyford et al. 2003) and typically 50% or more than the TOV limit (Morrison et al. 2004).

A supramassive relativistic star approaching the axisymmetric instability will actually *spin up* before collapse, even though it loses angular momentum (Cook et al. 1992, 1994b, a). This potentially observable effect is independent of the equation of state and it is more pronounced for rapidly rotating massive stars. Similarly, stars can be spun up by loss of angular momentum near the mass-shedding limit, if the equation of state is extremely stiff or extremely soft.

#### Multipole moments

The deformed shape of a rapidly rotating star creates a non-spherical distortion in the spacetime metric, and in the exterior vacuum region the metric is determined by a set of multipole moments, which arise at successively higher powers of $r^{-1}$. As in electromagnetism, the asymptotic spacetime is characterized by two sets of multipoles, mass multipoles and current multipoles, analogs of the electromagnetic charge multipoles and current multipoles.

The dependence of metric components on the choice of coordinates leads to the complication that in coordinate choices natural for a rotating star (including the quasi-isotropic coordinates) the asymptotic form of the metric includes information about the coordinates as well as about the multipole structure of the geometry. Because the metric potentials $\nu$, $\omega$ and $\psi$ are scalars constructed locally from the metric and the symmetry vectors $t^\alpha$ and $\phi ^\alpha$, as in Eqs. (11–13), their definition is in this sense coordinate-independent. But, the functional forms, $\nu (r,\theta )$, $\omega (r,\theta )$, $\psi (r,\theta )$, depend on *r* and $\theta$ and one must disentangle the physical mass and current moments from the coordinate contributions.

Up to $O(r^{-3})$, the only contributing multipoles are the monopole and quadrupole mass moments and the $l=1$ current moment. Two approaches to asymptotic multipoles of stationary systems, developed by Thorne (1980) and by Geroch (1970b) and Hansen (1974) yield identical definitions for $l\le 2$, while higher multipoles differ only in the normalization chosen. Ryan (1995) and Laarakkers and Poisson (1999) provide coordinate invariant definitions of multiple moments.

In the nonrotating limit, the quasi-isotropic metric (5) takes the isotropic form

62 $$\begin{aligned} ds^2 = -\left( \frac{1-M/2r}{1+M/2r}\right) ^2 \, dt^2 + \left( 1+\frac{M}{2r}\right) ^4(dr^2+ r^2\sin ^2\theta d\phi ^2 + r^2d\theta ^2), \qquad \end{aligned}$$

with asymptotic form

63 $$\begin{aligned} ds^2= & {} -\left[ 1-\frac{2M}{r}+2\frac{M^2}{r^2}-\frac{1}{4}\frac{M^3}{r^3}+O(r^{-5})\right] dt^2 \nonumber \\&+ \left[ 1+\frac{2M}{r}+\frac{3}{2}\frac{M^2}{r^2}+O(r^{-3})\right] (dr^2+r^2\sin ^2\theta d\phi ^2 + r^2d\theta ^2), \phantom {xxx} \end{aligned}$$

Thus, the metric potentials $\nu$, $\mu$ and $\psi$ have asymptotic behavior

64 $$\begin{aligned} \nu= & {} -\frac{M}{r} -\frac{1}{12}\frac{M^3}{r^3} +O(r^{-5}), \end{aligned}$$

65 $$\begin{aligned} \mu= & {} \frac{M}{r} - \frac{1}{4}\frac{M^2}{r^2}+\frac{1}{12}\frac{M^3}{r^3} +O(r^{-4}),\end{aligned}$$

66 $$\begin{aligned} \psi= & {} \log (r\sin \theta ) +\mu . \end{aligned}$$

For a rotating star, the asymptotic metric differs from the nonrotating form already at $O(r^{-2})$. Through $O(r^{-3})$ there are three corrections due to rotation: (i) the frame dragging potential $\displaystyle \omega \sim \frac{2J}{r^3}$; (ii) a quadrupole correction to the diagonal metric coefficients at $O(r^{-3})$ associated with the mass quadrupole moment *Q* of the rotating star; and (iii) *coordinate-dependent* monopole and quadrupole corrections to the diagonal metric coefficients (reflecting the asymptotic shape of the *r*- and $\theta$- surfaces) which can be described by a dimensionless parameter *a*.

For convenience, one can define a dimensionless qudrupole moment parameter $q := Q/M^3$. Then, Friedman and Stergioulas (2013) show that the asymptotic form of the metric is given in terms of the parameters *M*, *J*, *q* and *a* by:

67 $$\begin{aligned} \nu= & {} -\frac{M}{r} -\frac{1}{12}\frac{M^3}{r^3} +\left( a - 4aP_2 - qP_2\right) \frac{M^3}{r^3} +O(r^{-4}), \end{aligned}$$

68 $$\begin{aligned} \mu= & {} \frac{M}{r} - \frac{1}{4}\frac{M^2}{r^2}+\frac{1}{12}\frac{M^3}{r^3} -(a-4a P_2)\frac{M^2}{r^2} -(a-4a P_2 - qP_2)\frac{M^3}{r^3} \nonumber \\&+ O(r^{-4}),\end{aligned}$$

69 $$\begin{aligned} \psi= & {} \log (r\sin \theta ) +\mu + O(r^{-4}), \end{aligned}$$

70 $$\begin{aligned} \omega= & {} \frac{2J}{r^3} + O(r^{-4}), \end{aligned}$$

where $P_2$ is the Legendre polynomial $P_2(\cos \theta )$. The coefficient of $-P_2/r^3$ in the expansion of the metric potential $\nu$ is thus $Q+4aM^3$, from which the quadrupole moment *Q* can be extracted, if the parameter *a* has been determined from the coefficient of $P_2/r^2$ in the expansion of the metric potential $\mu$. Notice that sometimes the coeffient of $-P_2/r^3$ in the expansion of $\nu$ is identified with *Q* (instead of $Q+4aM^3$), which can lead to a deviation of up to about $20\%$ in the numerical values of the quadrupole moment. Pappas and Apostolatos (2012) have independently verified the correctness of the identification in Friedman and Stergioulas (2013) and also provide the correct identification of the current-octupole moment.

Laarakkers and Poisson (1999) found that along a sequence of fixed gravitational mass *M*, the quadrupole moment *Q* scales quadratically with the angular momentum, as

71 $$\begin{aligned} Q = -a_2 \frac{J^2}{Mc^2} = -a_2 \chi ^2 M^3, \end{aligned}$$

where $a_2$ is a dimensionless coefficient that depends on the equation of state, and $\chi :=J/M^2$. In Laarakkers and Poisson (1999), the coefficient $a_2$ varied between $a \sim 2$ for very soft EOSs and $a \sim 8$ for very stiff EOSs, for sequences of $M=1.4\,M_{\odot }$, but these values were computed with the erroneous identification of *Q* discussed above. Pappas and Apostolatos (2012) verify the simple form of (71) and provide corrected values for the parameter $a_2$ as well as similar relations for other multipole moments. Pappas and Apostolatos (2014) and Yagi et al. (2014) have further found that in addition to *Q*, the spin octupole $S_3$ and mass hexadecapole $M_4$ also have scaling relationships for realistic equations of state as follows

72 $$\begin{aligned} S_3= & {} -\beta _3 \chi ^3 M^4, \end{aligned}$$

73 $$\begin{aligned} M_4= & {} \gamma _4 \chi ^4 M^5, \end{aligned}$$

where $\beta _3$ and $\gamma _4$ are dimensionless constants.

#### Mass-shedding limit and the empirical formula

Mass-shedding occurs when the angular velocity of the star reaches the angular velocity of a particle in a circular Keplerian orbit at the equator, i.e., when

74 $$\begin{aligned} \varOmega = \varOmega _{\mathrm {K}}, \end{aligned}$$

where

75 $$\begin{aligned} \varOmega _{\mathrm {K}}=\frac{\omega '}{2\psi '} + e^{\nu -\psi }\left[ c^2\frac{\nu '}{\psi '}+ \left( \frac{\omega '}{2\psi '}e^{\psi -\nu }\right) ^2\right] ^{1/2} \!\!\!+\omega , \end{aligned}$$

(a prime indicates radial differentiation). In differentially rotating stars, even a small amount of differential rotation can significantly increase the angular velocity required for mass-shedding. Thus, a newly-born, hot, differentially rotating neutron star or a massive, compact object formed in a binary neutron star merger could be sustained (temporarily) in equilibrium by differential rotation, even if a uniformly rotating configuration with the same rest mass does not exist.

In the Newtonian limit, one can use the Roche model to derive the maximum angular velocity for uniformly rotating polytropic stars, finding $\varOmega _K \simeq (2/3)^{3/2} (GM/R^3)^{1/2}$ (see Shapiro and Teukolsky 1983). An identical result is obtained in the relativistic Roche model of Shapiro et al. (1983). For relativistic stars, the empirical formula (Haensel and Zdunik 1989; Friedman et al. 1989; Friedman 1990; Haensel et al. 1995)

76 $$\begin{aligned} \varOmega _K = 0.67 \sqrt{\frac{G M^{\max }_\mathrm{sph}}{(R^{\max }_\mathrm{sph})^3}}, \end{aligned}$$

gives the maximum angular velocity in terms of the mass and radius of the maximum mass *nonrotating* (spherical) model with an accuracy of 5–7%, without actually having to construct rotating models. Expressed in terms of the minimum period $P_\mathrm{min}=2\pi /\varOmega _K$, the empirical formula reads

77 $$\begin{aligned} P_{\min } \simeq 0.82 \left( \frac{M_\odot }{M_\mathrm{sph}^{\max }} \right) ^{1/2} \left( \frac{R_\mathrm{sph}^{\max }}{10\,\mathrm {km}} \right) ^{3/2}\,\mathrm {ms}. \end{aligned}$$

The empirical formula results from universal proportionality relations that exist between the mass and radius of the maximum mass rotating model and those of the maximum mass nonrotating model for the same EOS. Lasota et al. (1996) found that, for most EOSs, the numerical coefficient in the empirical formula is an almost linear function of the parameter

78 $$\begin{aligned} \chi _s = \frac{2GM^{\max }_\mathrm{sph}}{R^{\max }_\mathrm{sph} c^2}. \end{aligned}$$

The Lasota et al. empirical formula

79 $$\begin{aligned} \varOmega _K = (0.468+0.378 \chi _s) \sqrt{\frac{G M^{\max }_\mathrm{sph}}{(R^{\max }_\mathrm{sph})^3}}, \end{aligned}$$

reproduces the exact values with a relative error of only $1.5\%$. The corresponding formula for $P_{\min }$ is

80 $$\begin{aligned} P_{\min } \simeq \frac{0.187}{(\chi _s)^{3/2}(1+0.808\chi _s)} \left( \frac{M_\odot }{M_\mathrm{sph}^{\max }} \right) \mathrm{ms}. \end{aligned}$$

The above empirical relations are specifically constructed for the most rapidly rotating model for a given EOS.

Lattimer and Prakash (2004) suggest the following empirical relation

81 $$\begin{aligned} P_{\min } \simeq 0.96 \left( \frac{M_\odot }{M} \right) ^{1/2} \left( \frac{R_\mathrm{sph}}{10\,\mathrm {km}} \right) ^{3/2}\,\mathrm {ms}, \end{aligned}$$

for any neutron star model with mass *M* and radius $R_\mathrm{sph}$ of the nonrotating model with same mass, as long as its mass is not close to the maximum mass allowed by the EOS. Haensel et al. (2009) refine the above formula, giving a factor of 0.93 for hadronic EOSs and 0.87 for strange stars. A corresponding empirical relation between the radius at maximal rotation and the radius of a nonrotating configuration of same mass also exists.

Using the above relation, one can set an approximate constraint on the radius of a nonrotating star with mass *M*, given the minimum observed rotational period of pulsars.

#### Upper limits on mass and rotation: theory versus observation

*Maximum mass*: Candidate EOSs for high density matter predict vastly different maximum masses for nonrotating models. One of the stiffest proposed EOSs (EOS L) has a nonrotating maximum mass of $3.3\,M_{\odot }$. Some core-collapse simulations suggest a bi-modal mass distribution of the remnant, with peaks at about $1.3\,M_{\odot }$ and $1.7\,M_{\odot }$ (Timmes et al. 1996).

Observationally, the masses of a large number of compact objects have been determined, but, in most cases, the observational error bars are still large. A recent review of masses and spins of neutron stars as determined by observations was presented by Miller and Miller (2015). The heaviest neutron stars with the most accurately determined masses ever observed are PSR J1614-2230, with $M=1.97\pm 0.04\,M_{\odot }$ (Demorest et al. 2010) and PSR J0348+0432, with $2.01\pm 0.04$ (Antoniadis et al. 2013), and there are indications for even higher masses (see Haensel et al. 2007 for a detailed account). Masses of compact objects have been measured in different types of binary systems: double neutron star binaries, neutron star-white dwarf binaries, X-ray binaries and binaries composed of a compact object around a main sequence star. For most double neutron star binaries, masses have already been determined with good precision and are restricted to a narrow range of about $1.2-1.4\,M_{\odot }$ (Thorsett and Chakrabarty 1999). This narrow range of relatively small masses is probably associated with an upper mass limit on iron cores, which in turn is related to the stability of the core of each progenitor star. Masses determined for compact stars in X-ray binaries still have large error bars, but are consistently higher than $1.4\,M_{\odot }$, which is probably the result of mass-accretion. A similar finding seems to apply to white dwarf–neutron star binaries (see Paschalidis et al. 2009 and references therein).

*Minimum period*: When magnetic-field effects are ignored, conservation of angular momentum can yield very rapidly rotating neutron stars at birth. Simulations of the rotational core collapse of evolved rotating progenitors (Heger et al. 2000; Fryer and Heger 2000) have demonstrated that rotational core collapse could result in the creation of neutron stars with rotational periods of the order of 1 ms (and similar initial rotation periods have been estimated for neutron stars created in the accretion-induced collapse of a white dwarf, Liu and Lindblom 2001). However, magnetic fields may complicate this picture. Spruit and Phinney (1998) have presented a model in which a strong internal magnetic field couples the angular velocity between core and surface during most evolutionary phases. The core rotation decouples from the rotation of the surface only after central carbon depletion takes place. Neutron stars born in this way would have very small initial rotation rates, even smaller than the ones that have been observed in pulsars associated with supernova remnants. In this model, an additional mechanism is required to spin up the neutron star to observed periods. On the other hand, Livio and Pringle (1998) argue for a much weaker rotational coupling between core and surface by a magnetic field, allowing for the production of more rapidly rotating neutron stars than in Spruit and Phinney (1998). In Heger et al. (2004), intermediate initial rotation rates were obtained. Clearly, more detailed studies of the role of magnetic fields are needed to resolve this important question.

Independently of their initial rotation rate, compact stars in binary systems are spun up by accretion, reaching high rotation rates. In principle, accretion could drive a compact star to its mass-shedding limit. For a wide range of candidates for the neutron-star EOS, the mass-shedding limit sets a minimum period of about 0.5–0.9 ms (Friedman 1995). However, there are a number of different processes that could limit the maximum spin to lower values. In one model, the minimum rotational period of pulsars could be set by the occurrence of the *r*-mode instability in accreting neutron stars in LMXBs (Bildsten 1998; Andersson et al. 2000), during which gravitational waves carry away angular momentum. Other models are based on the standard magnetospheric model for accretion-induced spin-up (White and Zhang 1997), or on the idea that the spin-up torque is balanced by gravitational radiation produced by an accretion-induced quadrupole deformation of the deep crust (Bildsten 1998; Ushomirsky et al. 2000), by deformations induced by a very strong toroidal field Cutler (2002) or by magnetically confined “mountains” (Melatos and Payne 2005; Vigelius and Melatos 2008). With the maximum observed pulsar spin frequency at 716 Hz (Hessels et al. 2006) and a few more pulsars at somewhat lower rotation rates (Chakrabarty 2008), it is likely that one of the above mechanisms ultimately dominates over the accretion-induced spin-up, setting an upper limit that may be somewhat dependent on the final mass, the magnetic field or the spin-up history of the star. This is consistent with the absence of sub-millisecond pulsars in pulsar surveys that were in principle sensitive down to a few tenths of a millisecond (Burderi and D’Amico 1997; D’Amico 2000; Crawford et al. 2000; Edwards et al. 2001).

*EOS constraints*: One can systematize the observational constraints on the neutron-star EOS by introducing a parameterized EOS above nuclear density with a set of parameters large enough to encompass the wide range of candidate EOSs and small enough that the number of parameters is smaller than the number of relevant observations. Read et al. (2009) found that one can match a representative set of EOSs to within about 3% rms accuracy with a 4-parameter EOS based on piecewise polytropes.

Using spectral modeling to simultaneously estimate the radius and mass of a set of neutron stars in transient low-mass X-ray binaries, Özel et al. (2010) and Steiner et al. (2010) find more stringent constraints. They also adopt piecewise-polytropic parametrizations to find the more restricted region of the EOS space. Future gravitational-wave observations of inspiraling neutron-star binaries (Flanagan and Hinderer 2008; Read et al. 2009; Markakis et al. 2009, 2012; Duez et al. 2010; Bernuzzi et al. 2012; Damour et al. 2012) and of oscillating, post-merger remnants (Shibata et al. 2005; Bauswein et al. 2012; Bauswein and Janka 2012; Bauswein et al. 2014, 2016; Clark et al. 2016) may yield comparable or more accurate constraints without the model-dependence of the current electromagnetic studies.

The existence of $2.0\,M_{\odot }$ neutron stars in conjunction with nuclear physics place constraints on the neutron star EOS. For example, Hebeler et al. (2013) use microscopic calculations of neutron matter based on nuclear interactions derived from chiral effective field theory to constrain the equation of state of neutron-rich matter at sub- and supranuclear densities, arriving at a range of $9.7{-}13.9\,\mathrm {km}$ for the radius of nonrotating neutron stars, which is somewhat smaller than the range that a large sample of various proposed EOSs allow (the authors use a piecewise polytropic approach to derive the constraints). The corresponding range of compactness is 0.149–0.213.

A review of efforts to observationally constrain the EOS is given by Lattimer (2001). For recent reviews and the most up-to-date constraints on the neutron star radii and masses from electromagnetic observations see Lattimer (2012) and Özel and Freire (2016) and references therein. Future observations with missions such as NICER (Gendreau et al. 2012) and the proposed LOFT (Feroci and Stella 2012) have the potential to determine the neutron star radius with $\sim 5{-}10\%$ uncertainty, which will be useful in placing stringent (albeit model dependent) constraints on the EOS (see Psaltis et al. 2014).

#### Maximum mass set by causality

If one is interested in obtaining an upper limit on the mass, independent of the current uncertainty in the high-density part of the EOS for compact stars, one can construct a schematic EOS that satisfies only a *minimal set of physical constraints* and which yields a model of absolute maximum mass. The minimal set of constraints are

(0) *A relativistic star is described as a self-gravitating, uniformly rotating perfect fluid with a one-parameter EOS*, an assumption that is satisfied to high accuracy by cold neutron stars.
(1) *Matter at high densities satisfies the causality constraint* $c_s\equiv \sqrt{dp/d\epsilon } < 1$, where $c_\mathrm{s}$ is the sound speed. Relativistic fluids are governed by hyperbolic equations whose characteristics lie inside the light cone (consistent with the requirement of causality) only if $c_\mathrm{s}< 1$Geroch and Lindblom (1991).
(2) *The EOS is known at low densities* One assumes that the EOS describing the crust of cold relativistic stars is accurately known up to a matching energy density $\epsilon _m$.

For *nonrotating stars*, Rhoades and Ruffini (1974) showed that the EOS that satisfies the above constraints and yields the maximum mass consists of a high density region at the causal limit, $dp/d \epsilon =1$ (as stiff as possible), that matches directly to the assumed low density EOS at $\epsilon =\epsilon _m$

82 $$\begin{aligned} p(\epsilon )= & {} {\left\{ \begin{array}{ll} p_\mathrm{crust}(\epsilon ) &{}\quad \epsilon < \epsilon _m, \\ &{} \\ p_m+ \epsilon -\epsilon _m &{}\quad \epsilon > \epsilon _m, \end{array} \right. } \end{aligned}$$

where $p_m = p_\mathrm{crust}(\epsilon _m)$. For this *maximum mass EOS* and a *specific value* of the matching density, they computed a maximum mass of $3.2\,M_{\odot }$. More generally, $M_{\max }$ depends on $\epsilon _m$ as (Hartle and Sabbadini 1977; Hartle 1978)

83 $$\begin{aligned} M_{\max } = 4.8 \ \left( \frac{2 \times 10^{14}\,\mathrm {g/cm}^3}{ \epsilon _m/c^2} \right) ^{1/2}\,M_{\odot }. \end{aligned}$$

In the case of *uniformly rotating stars*, one obtains the following limit on the mass, when matching to the FPS EOS at low densities

84 $$\begin{aligned} M^\mathrm{rot}_{\max } = 6.1 \ \left( \frac{2 \times 10^{14}\,\mathrm {g/cm}^3}{ \epsilon _m/c^2} \right) ^{1/2} M_{\odot }, \end{aligned}$$

(see Friedman and Ipser 1987; Koranda et al. 1997).

#### Minimum period set by causality

A rigorous limit on the minimum period of uniformly rotating, gravitationally bound stars, allowed by causality, has been obtained in Koranda et al. (1997) (hereafter KSF), extending previous results by Glendenning (1992). The same three minimal constraints (0), (1) and (2) of Sect. 2.9.5, as in the case of the maximum mass allowed by causality, yield the minimum period. However, the *minimum period EOS* is different from the maximum mass EOS (82). KSF found that just the two constraints (0), (1) (without matching to a known low-density part) suffice to yield a simpler, *absolute minimum period EOS* and an absolute lower bound on the minimum period.

**Fig. 3:**
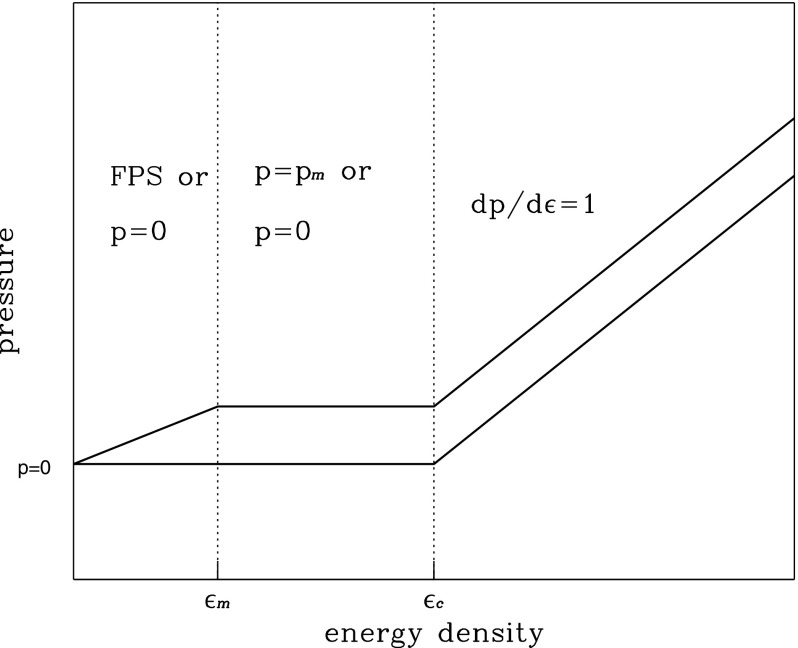
Schematic representations of the minimum-period EOSs (85) and (92). For the minimum-period EOS (85) the pressure vanishes for $\epsilon <\epsilon _C$. The minimum-period EOS (92) matches the FPS EOS to a constant pressure region at an energy density $\epsilon _m$. For $\epsilon > \epsilon _C$ both EOSs are at the causal limit with $dp/d\epsilon =1$. (Image reproduced with permission from Koranda et al. 1997, copyright by AAS)

*Absolute minimum period, without matching to low-density EOS*: Considering only assumptions (0) and (1), so that the EOS is constrained only by causality, the minimum period EOS is simply

85 $$\begin{aligned} p(\epsilon )= & {} {\left\{ \begin{array}{ll} 0 &{} \quad \epsilon \le \epsilon _C, \\ &{} \\ \epsilon -\epsilon _C &{}\quad \epsilon \ge \epsilon _C, \end{array} \right. } \end{aligned}$$

describing a star entirely at the causal limit $dp/d\epsilon =1$, with surface energy density $\epsilon _C$. This is not too surprising. A soft EOS yields stellar models with dense central cores and thus small rotational periods. Soft EOSs, however, cannot support massive stars. This suggests that the model with minimum period arises from an EOS which is *maximally stiff* ($dp/d\epsilon =1$) at high density, allowing stiff cores to support against collapse, but *maximally soft* at low density ($dp/d\epsilon =0$), allowing small radii and thus fast rotation, in agreement with (85). The minimum period EOS is depicted in Fig. 3 and yields an absolute lower bound on the period of uniformly rotating stars obeying the causality constraint, independent of any specific knowledge about the EOS for the matter composing the star. Choosing different values for $\epsilon _C$, one constructs EOSs with different $M_\mathrm{sph}^{\max }$. All properties of stars constructed with EOS (85) scale according to their dimensions in gravitational units and thus, the following relations hold between different maximally rotating stars computed from minimum-period EOSs with different $\epsilon _C$:

86 $$\begin{aligned} P_\mathrm{min}\propto & {} M_\mathrm{sph}^{\max } \propto R_\mathrm{sph}^{\max }, \end{aligned}$$

87 $$\begin{aligned} \epsilon _\mathrm{sph}^{\max }\propto & {} \frac{1}{\bigl ( M_\mathrm{sph}^{\max } \bigr )^2}, \end{aligned}$$

88 $$\begin{aligned} M_\mathrm{rot}^{\max }\propto & {} M_\mathrm{sph}^{\max }, \end{aligned}$$

89 $$\begin{aligned} R_\mathrm{rot}^{\max }\propto & {} R_\mathrm{sph}^{\max }, \end{aligned}$$

90 $$\begin{aligned} \epsilon _\mathrm{rot}^{\max }\propto & {} \epsilon _\mathrm{sph}^{\max }. \end{aligned}$$

A fit to the numerical results, yields the following relation for the absolute minimum period

91 $$\begin{aligned} \frac{P_{\min }}{\mathrm{ms}} = 0.196 \left( \frac{M_{\mathrm{sph}}^{\max }}{{ M}_\odot } \right) . \end{aligned}$$

Thus, for $M_\mathrm{sph}^{\max }=2\,M_\odot$ the absolute minimum period is 0.39 ms.

*Minimum period when low-density EOS is known*: Assuming all three constraints (0), (1) and (2) of Sect. 2.9.5 (so that the EOS matches to a known EOS at low density), the minimum-period EOS is

92 $$\begin{aligned} p(\epsilon )= & {} {\left\{ \begin{array}{ll} p_\mathrm{crust}(\epsilon ) &{} \quad \epsilon \le \epsilon _m, \\ &{} \\ p_m &{}\quad \epsilon _m\le \epsilon \le \epsilon _C, \\ &{} \\ p_m + \epsilon -\epsilon _C &{}\quad \epsilon \ge \epsilon _C. \end{array} \right. } \end{aligned}$$

**Fig. 4:**
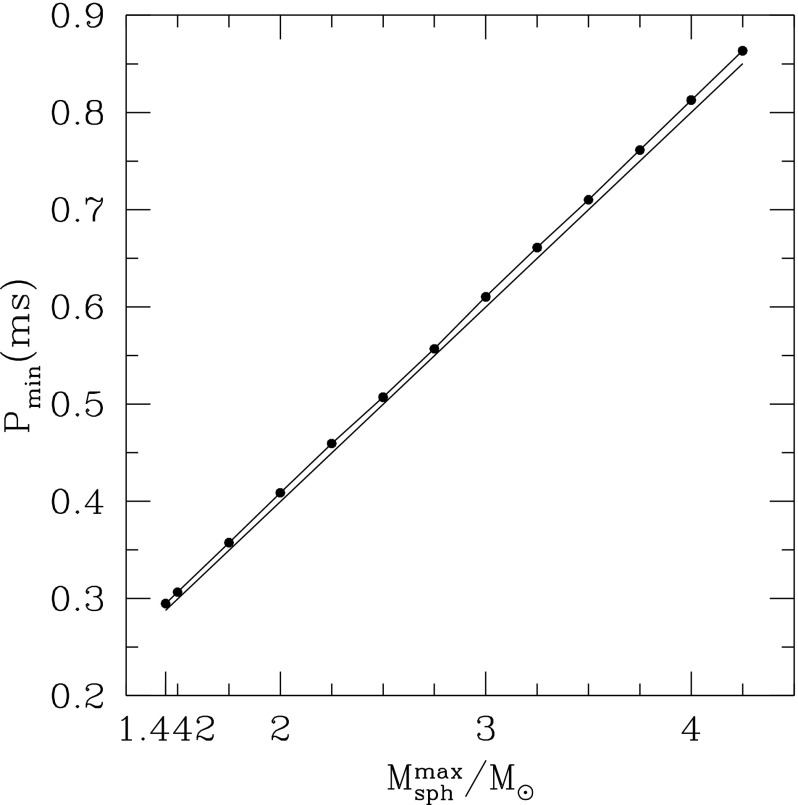
Minimum period $P_{\min }$ allowed by causality for uniformly rotating, relativistic stars as a function of the mass $M_\mathrm{sph}^{\max }$ of the maximum mass nonrotating model. Lower curve: constructed using the absolute minimum-period EOS (85), which does not match at low densities to a known EOS. Upper curve: constructed using the minimum-period EOS (92), which matches at low densities to the FPS EOS. Due to the causality constraint, the region below the curves is inaccessible to stars. (Image reproduced with permission from Koranda et al. 1997, copyright by AAS)

Between $\epsilon _m$ and $\epsilon _C$ the minimum period EOS has a constant pressure region (a first order phase transition) and is maximally soft, while above $\epsilon _{C}$ the EOS is maximally stiff, see Fig. 3. For a matching number density of $n_m=0.1\>\,\mathrm {fm}^{-3}$ to the FPS EOS, the minimum period allowed by causality is shown as a function of $M^{\max }_\mathrm{sph}$ in Fig. 4. A quite accurate linear fit of the numerical results is

93 $$\begin{aligned} \frac{P_{\min }}{ ms} = 0.295 + 0.203 \ \left( \frac{M_\mathrm{sph}^{\max }}{{ M}_\odot }-1.442\right) . \end{aligned}$$

Thus, if $M_\mathrm{sph}^{\max }=2\,M_{\odot }$, the minimum period is $P_\mathrm{min}=0.41\,\mathrm {ms}$. This result is rather insensitive to $n_m$, for $n_m <0.2\,\mathrm {fm}^{-3}$, but starts to depend significantly on $n_m$ for larger matching densities.

Comparing (93)–(91) it is evident that the currently trusted part of the nuclear EOS plays a negligible role in determining the minimum period due to causality. In addition, since matching to a known low-density EOS *raises* $P_{\min }$, (91) represents an *absolute minimum period*.

#### Moment of inertia and ellipticity

The scalar moment of inertia of a neutron star, defined as the ratio $I=J/\varOmega$, has been computed for polytropes and for a wide variety of candidate equations of state (see, e.g., Stergioulas et al. 1999; Cook et al. 1994a, b; Friedman et al. 1986). For a given equation of state the maximum value of the moment of inertia typically exceeds its maximum value for a spherical star by a factor of 1.5–1.6. For spherical models, Bejger et al. (2005) obtain an empirical formula for the maximum value of *I* for a given EOS in terms of the maximum mass for that EOS and the radius of that maximum-mass configuration,

94 $$\begin{aligned} I_{\max ,\varOmega =0}\approx & {} 0.97\times 10^{45} \left( \frac{M_{\max }}{M_\odot } \right) \left( \frac{R_{M_{\max }}}{10\,\mathrm {km}}\right) ^{2\,\mathrm {g\ cm}^2}. \end{aligned}$$

Neutron-star moments of inertia can in principle be measured by observing the periastron advance of a binary pulsar (Damour and Schäfer 1988). Because the mass of each star can be found to high accuracy, this would allow a simultaneous measurement of two properties of a single neutron star (Morrison et al. 2004; Lattimer and Schutz 2005; Bejger et al. 2005; Read et al. 2009).

The departure of the shape of a rotating neutron star from axisymmetry can be expressed in terms of its ellipticity $\varepsilon$, defined in a Newtonian context by

95 $$\begin{aligned} \varepsilon := \frac{I_{xx}-I_{yy}}{I} = \sqrt{\frac{8\pi }{15}} \frac{Q_{22}}{I}, \end{aligned}$$

where $I=I_{zz}$ is the moment of inertia about the star’s rotation axis and the $m=2$ part of a neutron star’s quadrupole moment is given by

96 $$\begin{aligned} Q_{22} := \mathrm{Re}\int \rho Y_{22} r^2 \, dV, \end{aligned}$$

where $Y_{22}$ is the $l=2,m=2$ spherical harmonic.

Following Ushomirsky et al. (2000), Owen (2005) finds for the maximum value of a neutron star’s ellipticity the expression

97 $$\begin{aligned} \varepsilon _{\max }= & {} 3.3\times 10^{-7} \frac{\sigma _{\max }}{10^{-2}} \left( \frac{1.4\,M_{\odot }}{M}\right) ^{2.2} \left( \frac{R}{10\,\mathrm km}\right) ^{4.26} \nonumber \\&\times \left[ 1+0.7\left( \frac{M}{1.4\,M_{\odot }}\right) \left( \frac{10\,\mathrm km}{R}\right) \right] ^{-1}, \end{aligned}$$

where $\sigma _{\max }$ is the breaking strain of the crust, with an estimated value of order $10^{-2}$ for crusts below $10^8K$ (Chugunov and Horowitz 2010).

#### Rotating strange quark stars

Most rotational properties of strange quark stars differ considerably from the properties of rotating stars constructed with hadronic EOSs. First models of rapidly rotating strange quark stars were computed by Friedman et al. (1989) and by Lattimer et al. (1990). Nonrotating strange stars obey relations that scale with the constant $\mathcal{B}$ in the MIT bag-model of the strange quark matter EOS. In Gourgoulhon et al. (1999), scaling relations for the model with maximum rotation rate were also found. The maximum angular velocity scales as

98 $$\begin{aligned} \varOmega _{\max }=9.92 \times 10^3 \sqrt{\mathcal{B}_{60}} \ \mathrm{s}^{-1}, \end{aligned}$$

while the allowed range of $\mathcal{B}$ implies an allowed range of $0.513 \ \mathrm{ms}<P_\mathrm{min}<0.640 \ \mathrm{ms}$. The empirical formula (76) also holds for rotating strange stars with an accuracy of better than $2\%$. Rotation increases the mass and radius of the maximum mass model by 44 and 54%, correspondingly, significantly more than for hadronic EOSs.

Accreting strange stars in LMXBs will follow different evolutionary paths in a mass versus central energy density diagram than accreting hadronic stars (Zdunik et al. 2002). When (and if) strange stars reach the mass-shedding limit, the ISCO still exists (Stergioulas et al. 1999) (while it disappears for most hadronic EOSs). In Stergioulas et al. (1999) it was shown that the radius and location of the ISCO for the sequence of mass-shedding models also scales as $\mathcal{B}^{-1/2}$, while the angular velocity of particles in circular orbit at the ISCO scales as $\mathcal{B}^{1/2}$. Additional scalings with the constant *a* in the strange quark EOS (54) (that were proposed in Lattimer et al. 1990) were found to hold within an accuracy of better than $\sim 9\%$ for the mass-shedding sequence:

99 $$\begin{aligned} M \propto a^{1/2}, \qquad R \propto a^{1/4}, \qquad \varOmega \propto a^{-1/8}. \end{aligned}$$

In addition, it was found that models at the mass-shedding limit can have *T* / |*W*| as large as 0.28 for $M=1.34\,M_{\odot }$.

If strange stars have a solid normal crust, then the density at the bottom of the crust is the neutron drip density $\epsilon _\mathrm{ND}\simeq 4.1 \times 10^{11}\,\mathrm {g\ cm}^{-3}$, as neutrons are absorbed by strange quark matter. A strong electric field separates the nuclei of the crust from the quark plasma. In general, the mass of the crust that a strange star can support is very small, of the order of $10^{-5}\,M_{\odot }$. Rapid rotation increases by a few times the mass of the crust and the thickness at the equator becomes much larger than the thickness at the poles (Zdunik et al. 2001). The mass $M_\mathrm{crust}$ and thickness $t_\mathrm{crust}$ of the crust can be expanded in powers of the spin frequency $\nu _3=\nu /(10^3\,\mathrm {Hz})$ as

100 $$\begin{aligned} M_\mathrm{crust}= & {} M_\mathrm{crust,0}(1+0.24 \nu _3^2+0.16 \nu _3^8), \end{aligned}$$

101 $$\begin{aligned} t_\mathrm{crust}= & {} t_\mathrm{crust,0} (1+0.4 \nu _3^2+0.3\nu _3^6), \end{aligned}$$

where a subscript “0” denotes nonrotating values (Zdunik et al. 2001). For $\nu \le 500\,\mathrm {Hz}$, the above expansion agrees well with a quadratic expansion derived previously in Glendenning and Weber (1992). The presence of the crust reduces the maximum angular momentum and ratio of *T* / |*W*| by about 20%, compared to corresponding bare strange star models.

#### Rotating magnetized neutron stars

The presence of a magnetic field has been ignored in the models of rapidly rotating relativistic stars that were considered in the previous sections. The reason is that the inferred surface dipole magnetic field strength of pulsars ranges between $10^8$ and $2 \times 10^{13}\,\mathrm {G}$. These values of the magnetic field strength imply a magnetic field energy density that is too small compared to the energy density of the fluid, to significantly affect the structure of a neutron star. However, there exists another class of compact objects with much stronger magnetic fields than normal pulsars—*magnetars*, that could have global fields up to the order of $10^{15}\,\mathrm {G}$ (Duncan and Thompson 1992), possibly born initially with high spin (but quickly spinning down to rotational periods of a few seconds). In addition, even though moderate magnetic field strengths do not alter the bulk properties of neutron stars, they may have an effect on the damping or growth rate of various perturbations of an equilibrium star, affecting its stability. For these reasons, a fully relativistic description of magnetized neutron stars is necessary. However, for fields $<\,10^{15}\,\mathrm {G}$ a *passive* description, where one ignores the influence of the magnetic field on the equilibrium properties of the fluid and the spacetime is sufficient for most practical purposes.

The equations of electromagnetism and magnetohydrodynamics (MHD) in general relativity have been discussed in a number of works; see, e.g., Lichnerowicz (1967), Misner et al. (1973), Bekenstein and Oron (1978), Anile (1989), Gourgoulhon et al. (2011) and references therein. The electromagnetic (E/M) field is described by a vector potential $A_\alpha$, from which one constructs the antisymmetric Faraday tensor $F_{\alpha \beta }= \nabla _\alpha A_\beta - \nabla _\beta A_\alpha ,$ satisfying Maxwell’s equations

102 $$\begin{aligned} \nabla _\beta {}^*F^{\alpha \beta }= & {} 0, \end{aligned}$$

103 $$\begin{aligned} \nabla _\beta F^{\alpha \beta }= & {} 4\pi J^\alpha , \end{aligned}$$

where ${}^*F_{\alpha \beta }{}=\frac{1}{2}\epsilon _{\alpha \beta \gamma \delta }F^{\gamma \delta }$, with $\epsilon _{\alpha \beta \gamma \delta }$ the totally antisymmetric Levi-Civita tensor. In (103), $J^\alpha$ is the 4-current creating the E/M field and the Faraday tensor can be decomposed in terms of an electric 4-vector $E_\alpha =F_{\alpha \beta }u^\beta$ and a magnetic 4-vector $B_\alpha ={}^*F_{\beta \alpha }u^\beta$ which are measured by an observer comoving with the plasma and satisfy $E_\alpha u^\alpha =B_\alpha u^\alpha =0$.

The stress–energy tensor of the E/M field is

104 $$\begin{aligned} T_{\alpha \beta }^\mathrm{(em)}=\frac{1}{4\pi } \left( F_{\alpha \gamma }F_\beta {}^\gamma -\frac{1}{4}F^{\gamma \delta }F_{\gamma \delta } g_{\alpha \beta }\right) , \end{aligned}$$

and the conservation of the total stress–energy tensor leads to the Euler equation in magnetohydrodynamics

105 $$\begin{aligned} (\epsilon +p)u^\beta \nabla _\beta u^\alpha = -q^{\alpha \beta }\nabla _\beta p + q^\alpha {}_\delta F^\delta {}_\gamma J^\gamma , \end{aligned}$$

where $q_{\alpha \beta }:=g_{\alpha \beta }+u_\alpha u_\beta$. In the ideal MHD approximation, where the conductivity ($\sigma$) is assumed to be $\sigma \rightarrow \infty$, the MHD Euler equation takes the form

106 $$\begin{aligned} \left( \epsilon +p+\frac{B_\gamma B^\gamma }{4\pi } \right) u^\beta \nabla _\beta u_\alpha = - q_\alpha {}^\beta \left[ \nabla _\beta \left( p+\frac{B_\gamma B^\gamma }{8\pi } \right) - \frac{1}{4\pi }\nabla _\gamma (B_\beta B^\gamma ) \right] . \nonumber \\ \end{aligned}$$

In general, a magnetized compact star will possess a magnetic field with both poloidal and toroidal components. Then its velocity field may include *non-circular* flows that give rise to the toroidal component. In such case, the spacetime metric will include additional non-vanishing components. The general formalism describing such a spacetime has been presented by Gourgoulhon and Bonazzola (1993), but no numerical solutions of equilibrium models have been constructed, so far. Instead, one can look for special cases, where the velocity field is circular or *assume* that it is approximately so.

If the current is purely toroidal, i.e., of the form $(J_t, 0,0, J_\phi )$, then a theorem by Carter (1973) allows for equilibrium solutions with circular velocity flows and a purely poloidal magnetic field, of the form $(0,B_r, B_\theta , 0)$. In ideal MHD, a purely toroidal magnetic field, $(B_t,0,0,B_\phi )$, is also allowed, generated by a current of the form $(0, J_r,J_\theta , 0)$ (Oron 2002).

For *purely poloidal* magnetic fields, rotating stars must be uniformly rotating in order to be in a stationary equilibrium and the Euler equation becomes

107 $$\begin{aligned} \nabla (H-\ln u^t) -\frac{1}{\epsilon +p}(j^\phi -\varOmega j^t) \nabla A_\phi =0, \end{aligned}$$

where $j^\alpha$ is the conduction current (the component of $J^\alpha$ normal to the fluid 4-velocity). The hydrostationary equilibrium equation has a first integral in three different cases. These are (a) $(j^\phi -\varOmega j^t)=0$, (b) $(\epsilon +p)^{-1}(j^\phi -\varOmega j^t)=$ const., and (c) $(\epsilon +p)^{-1}(j^\phi -\varOmega j^t)=f(A_\phi )$. The first case corresponds to a vanishing Lorentz force and has been considered in Bekenstein and Oron (1979), Oron (2002) (force-free field). The second case is difficult to realize, but has been considered as an approximation in, e.g., Colaiuda et al. (2008). The third case is more general and was first considered in Bonazzola et al. (1993); Bocquet et al. (1995). After making a choice for the current and for the total charge, the system consisting of the Einstein equations, the hydrostationary equilibrium equation and Maxwell’s equations can be solved for the spacetime metric, the hydrodynamical variables and the vector-potential components $A_t$ and $A_\phi$, from which the magnetic and electric fields in various observer frames are obtained.

**Fig. 5:**
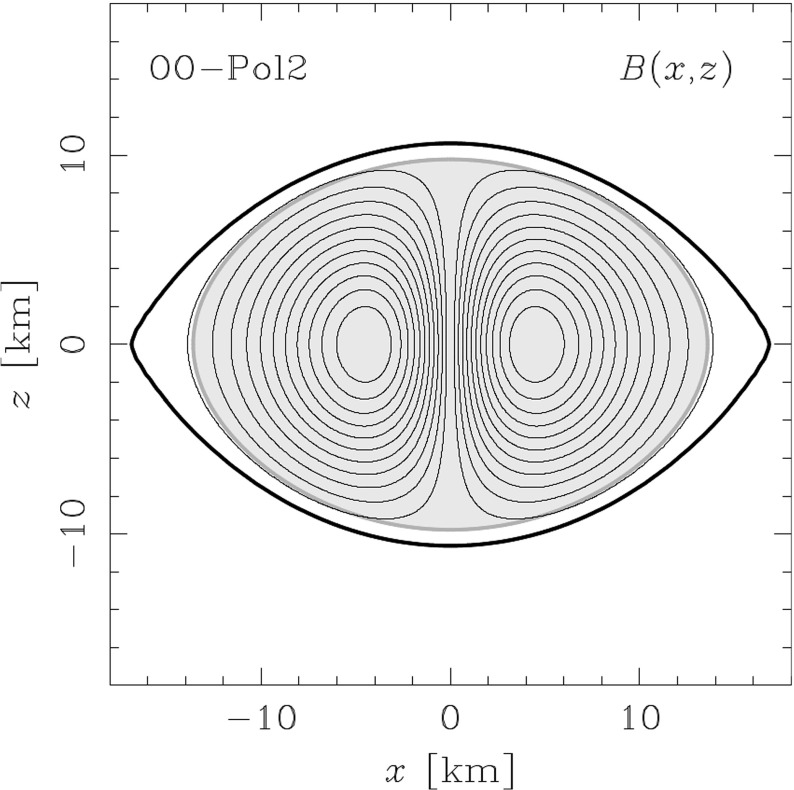
Isocontours of magnetic field strength in the meridional plane, for a rapidly rotating model with a purely toroidal magnetic field. (Image from Frieben and Rezzolla 2012, copyright by the authors)

For a *purely toroidal magnetic field*, the only non-vanishing component of the Faraday tensor is $F_{r\theta }$. Then, the ideal MHD condition does not lead to a restriction on the angular velocity of the star. For uniformly rotating stars, the Euler equation becomes (Kiuchi and Yoshida 2008; Gourgoulhon et al. 2011)

108 $$\begin{aligned} \nabla ( H -\ln u^t) + \frac{1}{4\pi (\epsilon +p)g_2} \sqrt{\frac{g_2}{g_1}} F_{r\theta } \nabla \left( \sqrt{\frac{g_2}{g_1}} F_{r\theta } \right) =0, \end{aligned}$$

where $g_1 = g_{rr} g_{\theta \theta }-(g_{r\theta })^2$, $g_2 = -g_{tt} g_{\phi \phi }+(g_{t\phi })^2$, which implies the existence of solutions for which $\sqrt{\frac{g_2}{g_1}} F_{r\theta }$ is a function of $(\epsilon +p)g_2$ (see Kiuchi and Yoshida 2008; Kiuchi et al. 2009 for representative numerical solutions). A detailed study of rapidly rotating equilibrium models with purely toroidal fields (in uniform rotation) was recently presented by Frieben and Rezzolla (2012) and Fig. 5 shows the isocontours of magnetic field strength in the meridional plane, for a representative case.

Gourgoulhon et al. (2011) find a general form of stationary axisymmetric magnetic fields, including non-circular equilibria.

Equilibria with purely toroidal or purely poloidal magnetic fields are unstable in nonrotating stars (and likely unstable in rotating stars), see, e.g., Wright (1973), Tayler (1973a), Markey and Tayler (1973), Lasky et al. (2011), Ciolfi and Rezzolla (2012) and Lasky et al. (2012). Some mixed poloidal/toroidal configurations seem more promising for stability (see, e.g., Duez et al. 2010, who infer stability of mixed equilibria in a Newtonian context from numerical evolutions).

A poloidal magnetic field in a differentially rotating star will be wound up, leading to the appearance of a toroidal component. This has several consequences, such as magnetic braking of the differential rotation, amplification of the magnetic field through dynamo action and the development of the magnetorotational instability see Sect. 2.12.

### Approximate universal relationships

Yagi and Yunes (2013b, 2013a) recently discovered a set of universal relationships that relate the moment of inertia, the tidal love number and the (spin-induced) quadrupole moment for slowly rotating neutron stars and quark stars (for another review of the *I*-Love-*Q* relations see also Yagi and Yunes 2017 where applications of these relations are also presented). The word “universal” in this context means within the framework of a particular theory of gravitation, but independent of the equation of state, provided the equation of state belongs to the class of *cold, realistic* equations of state, i.e., those that for the most part agree below the nuclear saturation density where our knowledge of nuclear physics is robust. More specifically, the universal relationships were established numerically between properly defined non-dimensional versions of the moment of inertia, the tidal Love number and the quadrupole moment. In particular, if *M* is the gravitational mass of the star, Yagi and Yunes introduced the following dimensionless quantities: $\bar{I} \equiv I/M^3$, $\bar{Q} \equiv -Q/(M^3\chi ^2)$, where $\chi \equiv J/M^2$ is the dimensionless NS spin parameter, and $\bar{\lambda }^\mathrm{(tid)}\equiv \lambda ^\mathrm{(tid)}/M^5$. Here $\lambda ^\mathrm{(tid)}$ is the tidal Love number, which determines the magnitude of the quadrupole moment tensor, $Q_{ij}$, induced on the star by an external quadrupole tidal tensor field $\mathcal {E}_{ij}$ through the relation $Q_{ij}=-\, \lambda ^\mathrm{(tid)}\mathcal {E}_{ij}$. The universal relations can be expressed through the following fitting formulae (Yagi and Yunes 2013b) (see also Lattimer and Lim 2013)

109 $$\begin{aligned} \ln y_i = a_i+b_i\ln x_i+c_i(\ln x_i)^2 + d_i(\ln x_i)^3+e_i(\ln x_i)^4, \end{aligned}$$

where $y_i$ and $x_i$ are a pair of two variables from the trio $\bar{I}$, $\bar{\lambda }^\mathrm{(tid)}$ and $\bar{Q}$, and the values of the coefficients $a_i,b_i,c_i,d_i,e_i$ are given in Table 3.

**Table 3:** Coefficients for the fitting formulae of the neutron star and quark star *I*-Love, $I{-}Q$ and Love-*Q* relations

$y_i$	$x_i$	$a_i$	$b_i$	$c_i$	$d_i$	$e_i$
$\bar{I}$	$\bar{\lambda }^\mathrm{(tid)}$	1.47	0.0817	0.0149	$2.87\times 10^{-4}$	$-\,3.64 \times 10^{-5}$
$\bar{I}$	$\bar{Q}$	1.35	0.697	$-$ 0.143	$9.94\times 10^{-2}$	$-\,1.24 \times 10^{-2}$
$\bar{Q}$	$\bar{\lambda }^\mathrm{(tid)}$	0.94	0.0936	0.0474	$-\,4.21\times 10^{-3}$	$1.23 \times 10^{-4}$

As pointed out in Yagi and Yunes (2013a) these relations could have been anticipated because in the Newtonian limit $\bar{I} \propto C^{-2}$, $\bar{Q} \propto C^{-1}$ and $\bar{\lambda }^\mathrm{(tid)} \propto C^{-5}$, indicating the existence of one-parameter relation between the trio $\bar{I}$, $\bar{\lambda }^\mathrm{(tid)}$, $\bar{Q}$. Here, *C* is the compactness of the star. The advantage of the existence of such universal relations is that in principle the measurement of one of the *I*-Love-*Q* parameters determines the other two, and one can use these relations to lift quadrupole moment and spin–spin degeneracies that arise in parameter estimation from future gravitational wave observations of compact binaries involving neutron stars (Yagi and Yunes 2013b, a). These relations could also help constrain modified theories of gravity (Yagi and Yunes 2013b, a) (but see below).

Shortly after the discovery of these relations, several works attempted to test the limits of the universality of these relations. Maselli et al. (2013) relaxed the small tidal deformation approximation assumed in Yagi and Yunes (2013b, 2013a) and derived universal relations for the different phases during a neutron star inspiral, concluding that these relations do not deviate significantly from those reported in Yagi and Yunes (2013b, 2013a). On the other hand, Haskell et al. (2014), considered neutron star quadrupole deformations that are induced by the presence of a magnetic field. They built self-consistent magnetized equilibria with the LORENE libraries and concluded that the *I*-Love-*Q* universal relations break down for slowly rotating neutron stars (spin periods $> 10$ s), and for polar magnetic field strengths $B_p > 10^{12}\,\mathrm {G}$. Doneva et al. (2014b) considered self-consistent, equilibrium models of spinning neutron stars beyond the slow-rotation approximation adopted in Yagi and Yunes (2013b, 2013a). They use the RNS code to built rapidly rotating stars, and find that with increasing rotation rate, the $\bar{I}$-$\bar{Q}$ relation departs significantly from its slow-rotation limit deviating up to 40% for neutron stars and up to 75% for quark stars. Moreover, they find that the deviation is EOS dependent and for a broad set of hadronic and strange matter EOS the spread due to rotation is comparable to the spread due to the EOS, if one considers sequences with fixed rotational frequency. For a restricted set of EOSs, that do not include models with extremely small or large radii, they were still able to find relations that are roughly EOS-independent at fixed rotational frequencies. However, Pappas and Apostolatos (2014) using the RNS code, showed that even for rapidly rotating neutron stars universality is again recovered, if instead of the $\bar{I}$-$\bar{Q}$ and angular frequency parameters, one focuses on the 3 dimensional parameter space spanned by the dimensionless spin angular momentum $\chi$, the dimensionless mass quadrupole $\bar{Q}$ and the dimensionless spin octupole moment $\beta _2\equiv -s_3/\chi ^3$, where $s_3 \equiv -S_3/M^4$, and where, again, $S_3$ is the spin octupole moment of the Hansen–Geroch moments (Geroch 1970a; Hansen 1974). Moreover, Pappas and Apostolatos (2014) show that if one considers the parameter space $(\chi ,\bar{I},\bar{Q})$, then the $I{-}Q$ EOS universality is recovered, in the sense that for each value $\chi$ there exists a unique universal $\bar{I}$-$\bar{Q}$ relation.

It should be pointed out (Yagi and Yunes 2013b, a; Pappas and Apostolatos 2014) that these “universal” relations hold not among the moments themselves, but among the rescaled, dimensionless moments, where the mass scale is factored out. Thus, the introduction of a scale will lift the apparent degeneracy among different EOSs.

Given the existence of such universal relations relating moments of neutron and quark stars, a fundamental question then arises: what is the origin of the universality? Yagi et al. (2014) performed a thorough study to answer this question and concluded that universality arises as an emergent approximate symmetry in that relativistic stars have an approximate self-similarity in their isodensity contours, which leads to the universal behavior observed in their exterior multipole moments. Work by Chan et al. (2015) has explored the origin of the *I*-Love relation through a post-Minkowskian expansion for the moment of inertia and the tidal deformability of incompressible stars.

Another way deviations from universality can take place are in protoneutron stars for which a cold, nuclear EOS in not applicable. Martinon et al. (2014) find that the *I*-Love-*Q* relations do not apply following one second after the birth of a protoneutron star, but that they are satisfied as soon as the entropy gradients are smoothed out typically within a few seconds. See also Marques et al. (2017), where a new finite temperature hyperonic equation of state is constructed and finds a similar conclusion as Martinon et al. (2014) regarding thermal effects.

Pani and Berti (2014) have extended the Hartle–Thorne formalism for slowly rotating stars to the case of scalar tensor theories of gravity and explored the validity of the *I*-Love-*Q* relations in scalar–tensor theories of gravity focusing on theories exhibiting the phenomenon of spontaneous scalarization (Damour and Esposito-Farèse 1993, 1996). Pani and Berti find that *I*-Love-*Q* relations exist in scalar-tensor gravity and interestingly also for spontaneously scalarized stars. Most remarkably, the relations in scalar–tensor theories coincide with their general relativity counterparts to within less than a few percent. This result implies that the *I*-Love-*Q* relations may not be used to distinguish between general relativity and scalar–tensor theories. We note that a similar conclusion was drawn by Sham et al. (2014) in the context of Eddington-inspired Born–Infeld gravity where the *I*-Love-*Q* relations were found to be indistinguishable than those of GR—an anticipated result (Pani and Berti 2014). More recently, Sakstein et al. (2017) have found the the *I*-*C* (*C* for compactness) in beyond Hordenski theories are clearly distinct from those in GR.

The effects of anisotropic pressure have been explored by Yagi and Yunes (2015). They find that anisotropy breaks the universality, but that the *I*-Love-*Q* relations remain approximately universal to within 10%. Finally, Yagi and Yunes (2016) considered anisotropic pressure to build slowly rotating, very high compactness stars that approach the black hole compactness limit, in order to answer the question of how the approximate *I*-Love-*Q* relations become exact in the BH limit. While the adopted methodology provides some hints into how the BH limit is approached, an interesting, and perhaps, definitive way to probe this is to consider unstable rotating neutron stars and perform dynamical simulations of neutron star collapse to black hole with full GR simulations.

In addition to the *I*-Love-*Q* relations, Pappas and Apostolatos (2014) find that for realistic equations of state there exists a universal relation between $\alpha _2$ and $\beta _3$, i.e., $\beta _3=\beta _3(\alpha _2)$, while Yagi et al. (2014) discover a similar universal relation between $\gamma _4$ and $\alpha _2$, i.e., $\gamma _4=\gamma _4(\alpha _2)$. These new approximate universal relations provide a type of “no-hair” relations among the multipole moments for neutron stars and quark stars. Motivated by these studies, Manko and Ruiz (2016b), show that there exists an infinite hierarchy of universal relations for neutron star multipole moments, assuming that neutron star exterior field can be described by four arbitrary parameters as in Manko et al. (1995).

### Rapidly rotating equilibrium configurations in modified theories of gravitation

With the arrival of “multimessenger” astronomy, gravitational wave and electromagnetic signatures of compact objects will soon offer a unique probe to test the limits of general relativity. Neutron stars are an ideal astrophysical laboratory for testing gravity in the strong field regime, because of their high compactness and because of the coupling of possible extra mediator fields with the matter.

Testing for deviations from general relativity would preferably require a generalized framework that parametrizes such deviations in an agnostic way as in the spirit of the parameterized post-Newtonian approach (Will 2014) (which systematically models post-Newtonian deviations from GR), or in the spirit of the parametrized post-Einsteinian approach (Yunes and Pretorius 2009; Cornish et al. 2011), which parametrizes a class of deviations from general relativistic waveforms within a certain regime. In the absence of such a complete parameterized framework, the existence of alternative theories of gravity are welcome not only as a means for testing for such deviations, but also for gaining a better understanding of how to develop a generalized, theory-agnostic framework of deviations from general relativity. Motivated by these ideas and by observations that can be interpreted as an accelerated expansion of the Universe (Riess et al. 1998) a number of extended theories of gravitation have been proposed as alternatives to a cosmological constant in order to explain dark energy (see e.g., Tsujikawa 2010; Paschalidis et al. 2011; Bloomfield et al. 2013; de Rham 2014; Joyce et al. 2016; Koyama 2016 for reviews and multiple aspects of such theories). The “infrared” predictions of modified gravity theories have been investigated extensively, and recently their strong-field predictions have attracted considerable attention (see e.g., Berti et al. 2015 for a review). Studies of spherically symmetric and slowly rotating neutron stars in modified gravity are reviewed in Berti et al. (2015), thus we focus here on the bulk properties of equilibrium, rapidly rotating neutron stars in modified theories of gravity.

Doneva et al. (2013b) presented a study of rapidly rotating neutron stars in scalar-tensor theories of gravity, by extending the RNS code to treat these theories in the Einstein frame, while computing physical quantities in the Jordan frame. The Jordan frame action considered in Doneva et al. (2013b) is given by

110 $$\begin{aligned} S = \frac{1}{16\pi }\int d^4 x\sqrt{-\tilde{g}}\left[ F(\varPhi )\tilde{R}-Z(\varPhi )g^{\mu \nu }\partial _\mu \varPhi \partial _\nu \varPhi - 2 U(\varPhi )\right] + S_m(\varPsi _m;\tilde{g}_{\mu \nu }),\nonumber \\ \end{aligned}$$

where $\tilde{g}_{\mu \nu }$ is the Jordan frame metric, $\tilde{R}$ the Ricci scalar accociated with $\tilde{g}_{\mu \nu }$, $\varPhi$ the scalar field, $U(\varPhi )$ the potential and $S_m$ denotes the matter action and $\varPsi _m$ denotes the matter fields. The functions $U(\varPhi )$, $F(\varPhi )$ and $Z(\varPhi )$ control the dynamics of the scalar field. However, requiring that the gravitons carry positive energy implies $Z(\varPhi ) > 0$, and non-negativity of the scalar field kinetic energy requires $2F(\varPhi )Z(\varPhi )+3(dF/d\varPhi )^2 \ge 0$. Note that the matter action does not involve $\varPhi$. Via a conformal transformation

111 $$\begin{aligned} g_{\mu \nu } = F(\varPhi ) \tilde{g}_{\mu \nu } \end{aligned}$$

and a scalar field redefinition via

112 $$\begin{aligned} \left( \frac{d\phi }{d\varPhi }\right) ^2 = \frac{3}{4}\left( \frac{d\ln F(\varPhi )}{d\varPhi }\right) ^2+ \frac{Z(\varPhi )}{2F(\varPhi )} \end{aligned}$$

and letting

113 $$\begin{aligned} \mathcal {A}(\phi )=1/\sqrt{F(\varPhi )}, 2V(\phi )=U(\varPhi )/F(\varPhi )^2, \end{aligned}$$

one recovers the Einstein frame action

114 $$\begin{aligned} S = \frac{1}{16\pi }\int d^4 x\sqrt{- g}\left[ R-2g^{\mu \nu }\partial _\mu \phi \partial _\nu \phi - 4 V(\phi )\right] + S_m(\varPsi _m;A(\phi )^2 g_{\mu \nu }) ,\qquad \end{aligned}$$

where *R* is the Ricci scalar associated with the Einstein frame metric $g_{\mu \nu }$. Variation of this action with respect to $g_{\mu \nu }$ and $\phi$ yields the equations of motion for the metric and for the scalar field, which are then cast in a convenient form and coupled to the equilibrium fluid equations that make it straightforward to extend the general relativistic equations solved by the RNS code. In their study, Doneva et al. (2013b) set $V(\phi )=0$ and consider two choices for the function $\mathcal {A}(\phi )$, namely $\ln \mathcal {A}(\phi )=k_0\phi$ and $\ln \mathcal {A}(\phi )=\beta \phi ^2/2$, while setting $\lim _{r\rightarrow \infty } \phi = 0$ and focusing on rigidly rotating equilibria. The former choice for $\mathcal {A}(\phi )$ is equivalent to Brans–Dicke theory, but the latter choice while it is indistinguishable from general relativity in the weak field regime, leads to the emergence of new phenomenology, such as a bifurcation due to non-uniqueness of solutions (Damour and Esposito-Farèse 1993, 1996). Observations currently constrain $k_0$ and $\beta$ to values $k_0 < 4\times 10^{-3}$ and $\beta \gtrsim -\,4.5$ (Will 2014; Freire et al. 2012; Antoniadis et al. 2013; Shibata et al. 2014). However, as pointed out in Popchev (2015); Ramazanoǧlu and Pretorius (2016), a massive scalar field naturally circumvents these observational bounds if the Compton wavelength of the scalar field is small compared to the binary orbital separation. The equation of state adopted in Doneva et al. (2013b) is a polytrope $P=k\rho ^{1+1/n}$, with $n=0.7463$ and $k=1186$ in units where $G=c=M_{\odot }=1$.

**Fig. 6:**
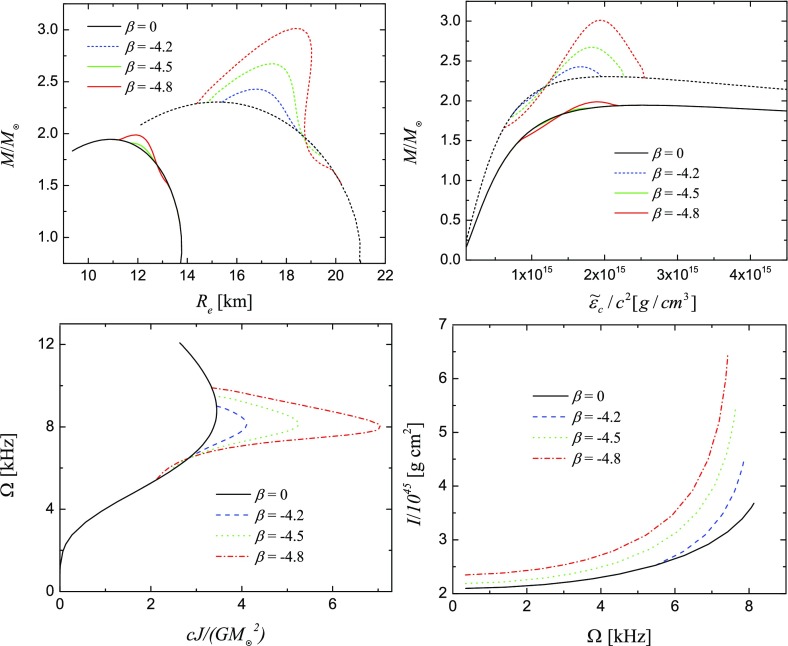
From left to right, top to bottom the plots correspond to mass versus radius, mass vs central energy density, angular frequency versus dimensionless angular momentum, and moment of inertia versus angular frequency. For the plots in the top row, solid lines correspond to nonrotating stars, while dotted lines correspond to the mass-shedding sequence. The angular frequency versus dimensionless angular momentum has only the mass-shedding sequence, while the moment of inertia plot corresponds to models for which the central energy density is fixed at $\sim \epsilon _c/c^2 = 1.5 \times 10^{15}\,\mathrm g/cm^3$. (Image reproduced with permission from Doneva et al. 2013b, copyright by APS)

For $\ln \mathcal {A}(\phi )=k_0\phi$ with the largest allowed value $k_0 = 4\times 10^{-3}$, Doneva et al. (2013b) find that even for stars rotating at the mass shedding limit, their the total mass, radius and angular momentum are practically indistinguishable from their counterparts in general relativity. However, for $\ln \mathcal {A}(\phi )=\beta \phi ^2/2$, while all general relativity solutions are also solutions of the scalar tensor theory with $\phi =0$, for certain values of $\beta$ and a certain range of neutron star densities new solutions emerge with non-trivial scalar field values that are also energetically favored (Damour and Esposito-Farèse 1993, 1996). This phenomenon is known as spontaneous scalarization and for the equation of state adopted in Doneva et al. (2013b), Harada has argued that the phenomenon occurs only for $\beta \lesssim -\,4.35$ (Harada 1998). One of the important findings in Doneva et al. (2013b) is that rapid rotation extends the range of $\beta$ values for which spontaneous scalarization can take place, and in particular that along the mass-shedding limit the bound becomes $\beta < -\,3.9$. In addition, it is found that rapid rotation changes significantly several bulk properties from their GR counterparts. Examples of such properties include the mass, radius, angular momentum, and moment of inertia as can be seen in Fig. 6. Of all bulk quantities those affected the most by the scalar field are the angular momentum and the moment of inertia of the star, which can differ up to a factor of two from their corresponding values in general relativity. It is also worth noting that the deviation of the bulk properties from their GR values, increases further if one considers smaller values of $\beta$, that are still in agreement with the observations. Based on the sensitivity of the moment of inertia (even at slow rotation rates), Doneva et al. suggested that the moment of inertia could be an astrophysical probe of theories exhibiting spontaneous scalarization.

In a subsequent paper, Doneva et al. (2014c) extended the equilibrium solutions of rapidly rotating compact stars for the spontaneous scalarization model $\ln \mathcal {A}(\phi )=\beta \phi ^2/2$ with $V(\phi )=0$, for tabulated equations of state. For the cases when scalarization occurs, they find results similar to those reported in Doneva et al. (2013b). In addition, they compute orbital and epicyclic frequencies for particles orbiting these neutron star models and find considerable differences of these frequencies between the scalar tensor theory and general relativity for the maximum-mass rotating models (but not so for models with spin frequency of $\sim 700\,\mathrm {Hz}$ or less, with the exception of very stiff equations of state).

The $I{-}Q$ relation for rapidly rotating stars in the model $\ln \mathcal {A}(\phi )=\beta \phi ^2/2$ was considered by Doneva et al. (2014a). The authors find that the $I{-}Q$ relation is nearly EOS independent for scalarized rapidly rotating stars, and that the spread of the relationship for higher rotation rates increases compared to general relativity. They also find that smaller negative values of $\beta$ lead to larger deviations from the general relativitivstic $I{-}Q$ relation, but the deviations (at most 5% for $\beta =-4.5$) are less than the anticipated accuracy of the observations. These results provide, yet, another example where the $I{-}Q$ relation may not be able to provide strong constraints on deviations from general relativity. We note that similar conclusions hold for rapidly rotating stars in Einstein–Gauss–Bonnet-dilaton gravity (Kleihaus et al. 2014).

In a recent paper, Doneva and Yazadjiev (2016) studied rapidly rotating stars for the model $\ln \mathcal {A}(\phi )=\beta \phi ^2/2$, but this time extending it to the case of a massive scalar field by adding a potential $V(\phi )=m_\phi ^2 \phi ^2/2$. In this case, In this case, the scalar field is short-range and observations practically leave the value of $\beta$ unconstrained. However, for the spontaneous scalarization of the neutron star one must have $10^{-16}\,\mathrm {eV} \lesssim m_\phi \lesssim 10^{-9}\,\mathrm {eV}$. Adopting the value $\beta = -\,6$, Doneva and Yazadjiev find that the $I{-}Q$ relation remains universal, but they deviate substantially (up to $\sim 20\%$) from those in general relativity. Thus, the $I{-}Q$ relation could be used to infer deviations from general relativity.

Another modified gravity theory that has been considered in the context of rapidly rotating stars is a particular model of *f*(*R*) gravity (Sotiriou and Faraoni 2010; de Felice and Tsujikawa 2010) with $f(R)=R+a R^2$ sometimes referred to as $R^2$ gravity. It can be shown that the Einstein frame action of this particular model of *f*(*R*) gravity can be cast in the form (114) with $\ln A(\phi )=-\phi /\sqrt{3}$, but with a non-zero potential $V(\phi )=(1-\exp (-2\phi /\sqrt{3}))^2/16a$ (Yazadjiev et al. 2015). Motivated by the results found for static and slowly rotating stars in $R^2$ gravity (Yazadjiev et al. 2014; Staykov et al. 2014), Yazadjiev et al. (2015) modified the RNS code to allow for the construction of rapidly rotating neutron star models in $R^2$ gravity. Adopting different equations of state, they find that rapid rotation enhances the discrepancy in global quantities such as mass, radius, and angular momentum between $R^2$-gravity and general relativistic stars. Also, the differences become larger as the coupling constant *a* increases. Generically, the $R^2$-gravity maximum neutron star mass is larger than the corresponding limit in general relativity. Yazadjiev et al. adopted $a/M_{\odot }^2\in [0,10^4]$, which is within the Gravity Probe B constraint $a\lesssim 5\times 10^{5}\,\mathrm {km^2}$, but much larger than the Eöt-Wash experiment constraint $a\lesssim 10^{-16}\,\mathrm {km^2}$ (Näf and Jetzer 2010). However, the bound from Gravity Probe B is still relevant because the chameleon nature of *f*(*R*) gravity can give rise to different effective values at different scales (Näf and Jetzer 2010). For the mass-shedding sequences and with $a=10^4\,M_{\odot }^2$, they find that for the equations of state considered, the maximum fractional differences between general relativity and $R^2$-gravity in maximum mass and maximum moment of inertia are 16.6 and 65.6%, respectively.

Armed with the $R^2$-gravity code, Doneva et al. (2015b) studied the universality of the $I{-}Q$ relation. They find that $R^2$ gravity exhibits an EOS-independent $I{-}Q$ relation, but that the differences with the Einstein gravity can be as large as $\sim 20\%$ for $a=10^4\,M_{\odot }^2$, similar to the deviations found in Doneva and Yazadjiev (2016) for a scalar–tensor model $\ln \mathcal {A}(\phi )=\beta \phi ^2/2$ and a massive scalar field. Thus, while it would be difficult to use the $I{-}Q$ relation in order to single out a specific extended theory of gravity, this relation could potentially be used to infer deviations from general relativity and to exclude some theories of extended gravity.

**Fig. 7:**
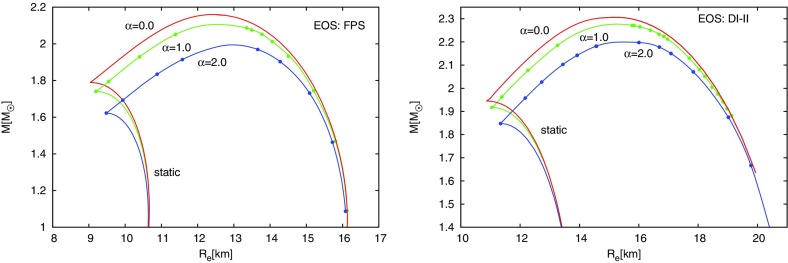
The physical regime on the mass-circumferential equatorial radius plane for neutron star solutions in EdGB theory for the FPS (left) and DI-II (right) EOSs. The left boundary in each panel designates the static sequence and the upper and right boundary the mass-shedding sequence. The values of the coupling constant $\alpha =0,1,2$ are in units of $M_{\odot }^2$. (Image reproduced with permission from Kleihaus et al. 2016, copyright by APS)

In addition to theories mentioned that can be cast in the usual form of scalar-tensor theories of gravity, the Einstein-dilaton-Gauss–Bonet (EdGB) theory is another example that has received attention in the context of rapidly rotating neutron stars. EdGB is inspired by heterotic string theory (Gross and Sloan 1987; Metsaev and Tseytlin 1987), and the effective action is given by

115 $$\begin{aligned} S = \frac{1}{16\pi }\int d^4 x\sqrt{- g}\left[ R-\frac{1}{2}g^{\mu \nu }\partial _\mu \varPhi \partial _\nu \varPhi + \alpha e^{-\beta \varPhi }R_{GB}^2\right] + S_m(\varPsi _m; g_{\mu \nu }),\qquad \end{aligned}$$

where $\varPhi$ is the dilaton field, $\gamma$ is a coupling constant, $\alpha$ is a positive coefficient and $R_{GB}^2=R_{\mu \nu \rho \sigma }R^{\mu \nu \rho \sigma }-4R_{\mu \nu }R^{\mu \nu }+R^2$ is the Gauss–Bonet term. The equations of motion for this theory are given by (see, e.g., Kleihaus et al. 2016)

116 $$\begin{aligned} \Box \varPhi= & {} \alpha \gamma e^{-\beta \varPhi }R_{GB}^2 \end{aligned}$$

117 $$\begin{aligned} G_{\mu \nu }= & {} 8\pi T_{\mu \nu }+\frac{1}{2}\left[ \nabla _\mu \varPhi \nabla _\nu \varPhi -\frac{1}{2}\nabla _\rho \varPhi \nabla ^\rho \varPhi \right] \nonumber \\&-\,\alpha e^{\beta \varPhi }\left[ H_{\mu \nu }+4(\beta ^2\nabla ^\rho \varPhi \nabla ^\sigma \varPhi -\beta \nabla ^\rho \nabla ^\sigma \varPhi )P_{\mu \rho \nu \sigma }\right] , \end{aligned}$$

where

118 $$\begin{aligned} H_{\mu \nu }= & {} 2\left[ RR_{\mu \nu }-2R_{\mu \rho }R^{\rho }{}_{\nu }-2R_{\mu \rho \nu \sigma }R^{\rho \sigma }+R_{\mu \rho \sigma \lambda }R_{\nu }{}^{\rho \sigma \lambda }\right] \nonumber \\&-\,\frac{1}{2}g_{\mu \nu }R^2_{GB}, \end{aligned}$$

119 $$\begin{aligned} P_{\mu \nu \rho \sigma }= & {} R_{\mu \nu \rho \sigma }+2g_{\mu [\sigma } R_{\rho ]\nu }+2g_{\nu [\rho } R_{\sigma ]\mu }+Rg_{\mu [\rho }g_{\sigma ]\nu }, \end{aligned}$$

are second-order partial differential equations because of the particular form of the Gauss–Bonet term. In this theory black hole solutions exist only for up to a maximum value of $|\alpha |$ (Kanti et al. 1996), hence rotating neutron star solutions are interesting to find only in this regime. Pani et al. (2011) build models of slowly rotating compact stars in this theory and find that only the product $\alpha \beta$ matters for the structure of compact stars in EdGB theory, whereas the larger the value of this product the smaller the maximum neutron star mass that can be supported in this theory. They also find that stellar solutions do not exist for arbitrarily large values of $\alpha \beta$ (this was already known about the existence of black hole solutions, Kanti et al. 1996, in this theory). As a result, the maximum observed mass could be used to place constraints on $\alpha \beta$.

Kleihaus et al. (2016) develop a code for building rapidly rotating neutron stars in EdGB theory. The authors consider two different equations of state, FPS and DI-II (Diaz Alonso and Ibanez Cabanell 1985). They confirm the results of Pani et al. (2011) and in addition find that rotation enhances the effects of deviations from GR (see Fig. 7). Furthermore, the authors find that the quadrupole moment depends on the value of the EdGB coupling constant and that the dependence is enhanced for larger value of the angular velocity. Finally, Kleihaus et al. discover that the GR $I{-}Q$ relation extends to EdGB theory with weak dependence on the value of the coupling parameter $\alpha$ when the NS dimensionless spin is 0.4. Therefore, EdGB theory provides yet another example where the $I{-}Q$ relations cannot be utilized to constrain deviations from GR.

### Differentially rotating neutron stars

The non-uniformity of rotation in the early stages of the life of a compact object (or right after the merger of a binary system) opens another dimension in the allowed parameter space of equilibrium models. The simplest description appropriate for neutron stars is the 1-parameter law (45), introduced in Komatsu et al. (1989a, b); Eriguchi et al. (1994). Relativistic models of differentially rotating stars were constructed numerically by Baumgarte et al. (2000), where it was pointed out that these configurations can support more mass than uniformly rotating stars. The authors coined the term “hypermassive” neutron stars for these compact objects whose mass exceeds the supramassive limit.

**Fig. 8:**
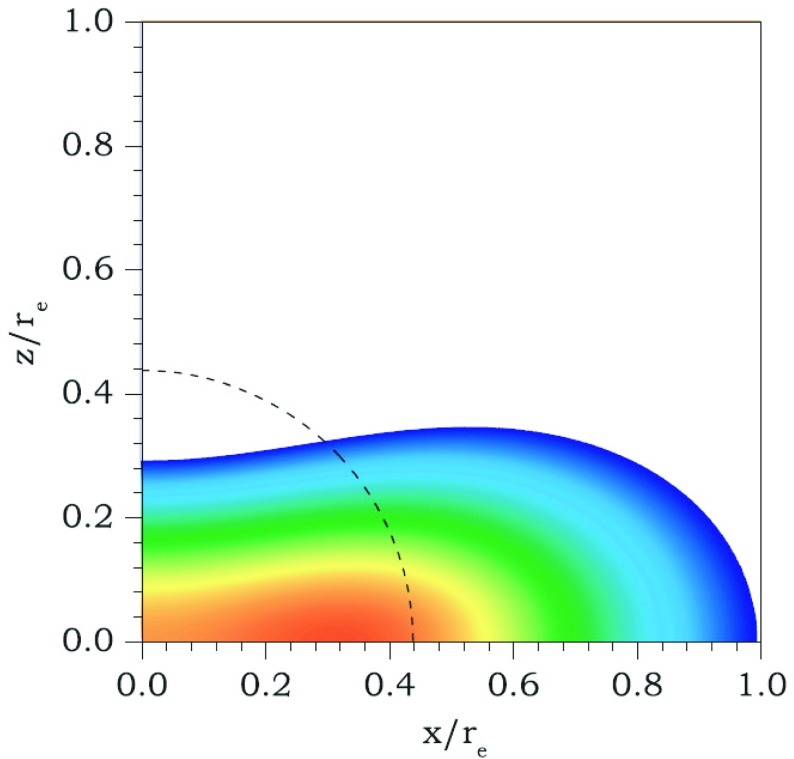
Density stratification for model A11 of Table 4, displaying an off-center density maximum. In comparison, the shape of the nonrotating star of same rest mass is shown, scaled by the equatorial radius of the rotating model (dashed line). (Image reproduced with permission from Stergioulas et al. 2004, copyright by RAS)

Examples of equilibrium sequences of differentially rotating polytropic models, using the above rotation law, were constructed by Stergioulas et al. (2004). Table 4 shows the detailed properties of a fixed rest mass sequence (A), in which the central density decreases as the star rotates more rapidly and of a sequence of fixed central density (B), in which the mass increases significantly with increasing rotation. $\varOmega _c$ and $\varOmega _e$ are the values of the angular velocity at the center and at the equator, respectively while $r_p$ and $r_e$ is the coordinate radius at the pole and at the equator (other quantities shown are defined as in Table 1). While most models along these sequences are quasi-spherical (meaning that the maximum density appears at the center), the fastest rotating members are quasi-toroidal, with an off-center maximum density. An example is shown in Fig. 8.

**Table 4:** Properties of two sequences of differentially rotating equilibrium models

Model	$\varepsilon _c$	*M*	*R*	$r_e$	$r_p/r_e$	$\varOmega _c$	$\varOmega _e$	*T* / |*W*|
	$(\times 10^{-3})$					$(\times 10^{-2})$	$(\times 10^{-2})$	
A0	1.444	1.400	9.59	8.13	1.0	0.0	0.0	0.0
A1	1.300	1.405	10.01	8.54	0.930	2.019	0.759	0.018
A2	1.187	1.408	10.40	8.92	0.875	2.580	0.977	0.033
A3	1.074	1.410	10.84	9.35	0.820	2.944	1.125	0.049
A4	0.961	1.413	11.37	9.87	0.762	3.192	1.232	0.066
A5	0.848	1.418	12.01	10.49	0.703	3.340	1.303	0.086
A6	0.735	1.422	12.78	11.25	0.643	3.383	1.336	0.107
A7	0.622	1.427	13.75	12.21	0.579	3.339	1.337	0.131
A8	0.509	1.433	15.01	13.45	0.513	3.197	1.300	0.158
A9	0.396	1.439	16.70	15.13	0.444	2.953	1.223	0.189
A10	0.283	1.447	19.03	17.44	0.370	2.604	1.101	0.223
A11	0.170	1.456	21.92	20.30	0.294	2.184	0.944	0.260
B0	1.444	1.400	9.59	8.13	1.0	0.0	0.0	0.0
B1	1.444	1.437	9.75	8.24	0.950	1.801	0.666	0.013
B2	1.444	1.478	9.92	8.36	0.900	2.574	0.944	0.026
B3	1.444	1.525	10.11	8.49	0.849	3.189	1.160	0.040
B4	1.444	1.578	10.31	8.63	0.800	3.728	1.342	0.055
B5	1.444	1.640	10.53	8.77	0.750	4.227	1.504	0.071
B6	1.444	1.713	10.76	8.91	0.700	4.707	1.651	0.087
B7	1.444	1.798	11.01	9.05	0.650	5.185	1.789	0.105
B8	1.444	1.899	11.26	9.17	0.600	5.683	1.921	0.124
B9	1.444	2.020	11.50	9.26	0.550	6.232	2.052	0.144
B10	1.444	2.167	11.71	9.27	0.500	6.889	2.192	0.165
B11	1.444	2.341	11.80	9.13	0.450	7.770	2.357	0.187
B12	1.444	2.532	11.64	8.72	0.400	9.118	2.584	0.207

Sequence A is a sequence of fixed rest mass with $M_0=1.506\,M_{\odot }$ and sequence B is a sequence of fixed central rest mass density $\rho _c=1.28 \times 10^{-3}$ with $A/r_e=1$. All models are relativistic polytropes with $N=1$, $K=100$ and rotation law parameter $A/r_e=1$. The definitions of the various quantities are given in the main text. All quantities are in dimensionless units with $c=G=M_{\odot }=1$. (Table adapted from Stergioulas et al. 2004)

**Fig. 9:**
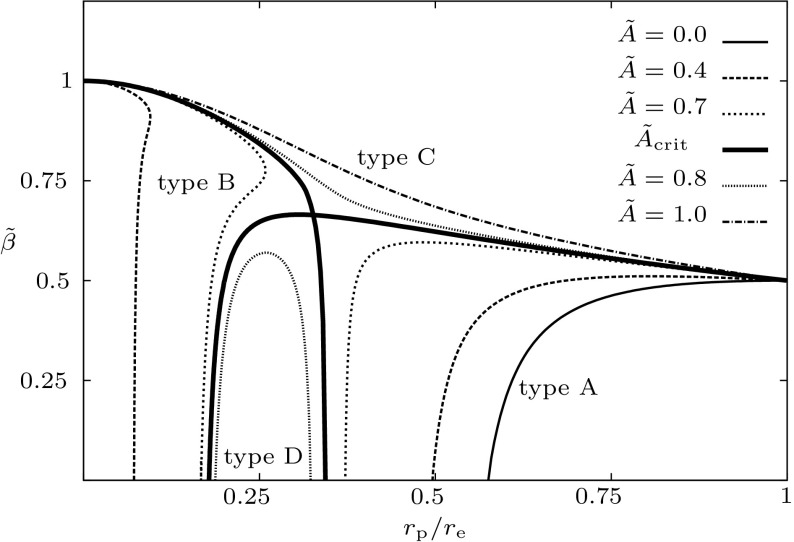
Different types of sequences of differentially rotating equilibrium models (see text for a detailed description). Here $\tilde{A} = \hat{A}^{-1}$. (Image reproduced with permission from Ansorg et al. 2009, copyright by the authors)

Ansorg et al. (2009) found 4 different types of differentially rotating models (which they label as type A, B, C and D) for the same 1-parameter law (45) which exists in parts of the allowed parameter space. For a sufficiently weak degree of differential rotation, sequences with increasing rotation terminate at the mass-shedding limit, but for moderate and strong rates of differential rotation equilibrium sequences can exhibit a continuous transition to a regime of toroidal fluid bodies. Figure 9 displays sequences of $N=1$ polytropes with various values of the parameter $\tilde{A}=\hat{A}^{-1}=r_e/A$ and a fixed central density. In the vertical axis, the parameter $\tilde{\beta }$ is related to the shape of the surface of the star and ranges between 0 (when rotation is limited by mass shedding at the equator) and 1 (when the radius on the polar axis becomes 0, indicating the transition to a toroidal configuration). As the axis ratio $r_p/r_e$ is varied, type A sequences start at a nonrotating model and terminate at the mass-shedding limit. Type B sequences have no nonrotating member, but connect models at the mass-shedding limit to toroidal configurations. Type C sequences connect the nonrotating limit to toroidal configurations, while type D sequences connect models at the mass-shedding limit to other models at the mass-shedding limit. It will be interesting to study the stability properties of models of these different types. One should keep in mind that these different types arise for the simple 1-parameter law (45) and a more complicated picture may arise for multi-parameter rotation laws. More recently, Studzińska et al. (2016) thoroughly explored the parameter space for the rotation law (45) and determined how the maximum mass depends on the stiffness, on the degree of differential rotation and on the maximum density, taking into account all types of solutions that were shown to exist in Ansorg et al. (2009).

A well know fact about differentially rotating neutron stars is that they can support more mass than the supramassive limit—the maximum mass when allowing for maximal uniform rotation. Neutron stars with mass larger than the supramassive limit are known as hypermassive neutron stars. Equilibrium sequences of differentially rotating models with polytropic equations of state, and using the same differential rotation law have been constructed by Lyford et al. (2003). There, the focus was on the effects of differential rotation on the maximum mass configuration. The authors find that differential rotation can support about 50% more mass than the TOV limit mass, as opposed to uniform rotation that typically increases the TOV limit by about 20%. In a subsequent paper, Morrison et al. (2004) extended this result to realistic equations of state. However, recent calculations by Gondek-Rosinska et al. (2017) focussing on $n=1$ polytropes, discover that the maximum mass depends not only on the degree of differential rotation, but also on the type of solution identified in Ansorg et al. (2009), i.e., A, B, C or D. The authors find that different classes have different maximum mass limits and even for moderate degrees of differential rotation $\hat{A}^{-1} \sim 1$, the maximum rest-mass configuration can be significantly higher than 2.0 times the TOV limit. Although, masses greater than two times the TOV limit can never be achieved in hypermassive neutron stars formed following a binary neutron star merger, it would be interesting to investigated the dynamical stability of the maximum mass configurations constructed in Gondek-Rosinska et al. (2017).

### Proto-neutron stars

Following the gravitational collapse of a massive stellar core, a proto-neutron star (PNS) is born with an initially large temperature of order 50 MeV and a correspondingly large radius of up to 100 km. If the PNS is slowly rotating, one can study its evolution assuming spherical symmetry (see, for example Burrows and Lattimer 1986; Burrows et al. 1995; Bombaci et al. 1995; Keil and Janka 1995; Keil et al. 1996; Prakash et al. 1997; Pons et al. 1999, 2000; Prakash et al. 2001; Strobel et al. 1999). Up to a time of about 100 ms after core bounce, the PNS is lepton rich and consists of an unshocked core at densities $n>0.1 \,\mathrm {fm}^{-3}$, with entropy per baryon $s\sim 1$, surrounded by a transition region and a low-density but high-entropy, shocked envelope with $s \sim$ 4–10, which extends to large radii. The lepton number is roughly $Y_l\sim 0.4$ and neutrinos in the core and in the transition region are trapped (the PNS is opaque to neutrinos), while at densities less than $n\sim 6\times 10^{-4}\,\mathrm {fm}^{-3}$ the outer envelope becomes transparent to neutrinos. Within about 0.5 s, the outer envelope cools and contracts with the entropy per baryon becoming roughly $s\sim 2$ throughout the star (the lepton number in the outer envelope drops to $Y_l\sim 0.3$). Further cooling results in a fully deleptonized, hot neutron star at several tens of seconds after core bounce, with a roughly constant entropy per baryon of $s\sim$ 1–2. After several minutes, when the neutron star has cooled to $T<1\,\mathrm {MeV}$, the thermal effects are negligibly small in the bulk of the star and a zero-temperature EOS can be used to describe its main properties.

The structure of hot PNSs is described by finite-temperature EOSs, such as those presented in Lattimer and Swesty (1991); Sugahara and Toki (1994); Toki et al. (1995); Lalazissis et al. (1997); Strobel et al. (1999b); Pons et al. (1999); Shen et al. (1998); Hempel and Schaffner-Bielich (2010); Typel et al. (2010); Fattoyev et al. (2010); Shen et al. (2011b, 2011a); Hempel et al. (2012); Steiner et al. (2013) (see also Oertel et al. 2017 for a review). These candidate EOSs differ in several respects (for example in the thermal pressure at high densities). The sample of cold EOSs that has been extended, so far, is quite limited and does not correspond to the wide range of possibilities allowed by current observational constraints. Therefore, PNS models that have been constructed only cover a small region of the allowed parameter space. Understanding the detailed evolution of a PNS is significant, as the star could undergo transformations that could be associated with direct or indirect observational evidence, such as the delayed collapse of a hypermassive PNS (see Brown and Bethe 1994; Baumgarte et al. 1996, 2000).

If the PNS is born rapidly rotating, its evolution will sensitively depend on the rotation rate and other factors, such as the development of the magnetorotational instability (MRI). Some partial understanding has emerged by studying quasi-equilibrium sequences of rotating models (Hashimoto et al. 1994; Strobel et al. 1999; Sumiyoshi et al. 1999; Villain et al. 2004). Exact equilibria can be found in the case that the model is considered to be barotropic, where all thermodynamical quantities (energy density, pressure, entropy, temperature) depend only on the baryon number density. Special cases, such as homentropic or isothermal stars have also been considered. In Villain et al. (2004) a barotropic EOS was constructed by rescaling temperature, entropy and lepton number profiles that were obtained from detailed, one-dimensional simulations of PNS evolutions, while the rotational properties of the models were taken from two-dimensional core-collapse simulations.

The main conclusion from the studies of sequences of quasi-equilibrium models is that PNSs that are born with moderate rotation, will contract and spin up during the cooling phase (see e.g., Goussard et al. 1998; Strobel et al. 1999). This could lead to a PNS rotating with large enough rotation rate for secular or dynamical instabilities to become interesting. It is not clear, however, whether the quasi-stationary approximation is valid when the stars reach the mass-shedding limit, as, upon further thermal contraction, the outer envelope could actually be shed from the star, resulting in an equatorial stellar wind. It should be noted here that a small amount of differential rotation significantly affects the mass-shedding limit, allowing more massive stars to exist than uniform rotation allows.

Studies of PNSs are being extended to include additional effects, such as entropy and lepton-driven convective instabilities and hydromagnetic instabilities (Epstein 1979; Livio et al. 1980; Burrows and Lattimer 1986; Burrows and Fryxell 1992; Miralles et al. 2000, 2002, 2004; Dessart et al. 2006; Lasky et al. 2012), meridional flows (Eriguchi and Müller 1991), local and mean-field magnetic dynamos (Thompson and Duncan 1993; Xu and Busse 2001; Bonanno et al. 2003; Reinhardt and Geppert 2005; Naso et al. 2008), magnetic braking and viscous damping of differential rotation (Shapiro 2000; Liu and Shapiro 2004; Duez et al. 2004; Thompson et al. 2005; Duez et al. 2006b), and the MRI and Tayler instabilities (Akiyama et al. 2003; Kotake et al. 2004; Thompson et al. 2005; Ardeljan et al. 2005; Masada et al. 2006; Shibata et al. 2006; Cerdá-Durán et al. 2007; Masada et al. 2007; Stephens et al. 2007; Bisnovatyi-Kogan and Moiseenko 2008; Spruit 2008; Kiuchi et al. 2008; Obergaulinger et al. 2009; Siegel et al. 2013; Guilet et al. 2016). These effects will be important for the evolution of both PNSs formed after core collapse, as well as for hypermassive or supramassive neutron stars possibly formed after a binary neutron star merger.

## Rotating relativistic stars in LMXBs

### Particle orbits and kHz quasi-periodic oscillations

X-ray observations of accreting sources in LMXBs have revealed a rich phenomenology that is waiting to be interpreted correctly and could lead to significant advances in our understanding of compact objects (see Lamb et al. 1998; van der Klis 2000; Psaltis 2001). The most important feature of these sources is the observation (in most cases) of twin kHz quasi-periodic oscillations (QPOs) (see van der Klis 2006; Abramowicz and Fragile 2013 for reviews on QPOs). The high frequency of these variabilities and their quasi-periodic nature are evidence that they are produced in high-velocity flows near the surface of the compact star. To date, there exist a large number of different theoretical models that attempt to explain the origin of these oscillations. No consensus has been reached, yet, but once a credible explanation is found, it will lead to important constraints on the properties of the compact object that is the source of the gravitational field in which the kHz oscillations take place. The compact stars in LMXBs are spun up by accretion, so that many of them may be rotating rapidly; therefore, the correct inclusion of rotational effects in the theoretical models for kHz QPOs is important. Under simplifying assumptions for the angular momentum and mass evolution during accretion, one can use accurate rapidly rotating relativistic models to follow the possible evolutionary tracks of compact stars in LMXBs (Cook et al. 1994c; Zdunik et al. 2002).

In most theoretical models, one or both kHz QPO frequencies are associated with the orbital motion of inhomogeneities or blobs in a thin accretion disk. In the actual calculations, the frequencies are computed in the approximation of an orbiting test particle, neglecting pressure terms. For most equations of state, stars that are massive enough possess an ISCO, and the orbital frequency at the ISCO has been proposed to be one of the two observed frequencies. To first order in the rotation rate, the orbital frequency at the prograde ISCO is given by (see Kluźniak et al. 1990)

120 $$\begin{aligned} f_{\mathrm {ISCO}} \simeq 2210 \, (1+0.75\chi ) \left( \frac{1\,M_{\odot }}{M} \right) \,\mathrm {Hz}, \end{aligned}$$

where $\chi :=J/M^2$. At larger rotation rates, higher order contributions of $\chi$ as well as contributions from the quadrupole moment *Q* become important and an approximate expression has been derived by Shibata and Sasaki (1998), which, when written as above and truncated to the lowest order contribution of *Q* and to $\mathcal{O}(\chi ^2)$, becomes

121 $$\begin{aligned} f_{\mathrm {ISCO}} \simeq 2210 \, (1+0.75\chi +0.78\chi ^2-0.23Q_2) \left( \frac{1\,M_{\odot }}{M} \right) \,\mathrm {Hz}, \end{aligned}$$

where $$Q_2=-Q/M^3$$.

Notice that, while rotation increases the orbital frequency at the ISCO, the quadrupole moment has the opposite effect, which can become important for rapidly rotating models. Numerical evaluations of $f_{\mathrm {ISCO}}$ for rapidly rotating stars have been used in Miller et al. (1998) to arrive at constraints on the properties of the accreting compact object.

In other models, orbits of particles that are eccentric and slightly tilted with respect to the equatorial plane are involved (the relativistic precession model). For eccentric orbits, the periastron advances with a frequency $\nu _{\mathrm {pa}}$ that is the difference between the Keplerian frequency of azimuthal motion $\nu _{\mathrm {K}}$ and the radial epicyclic frequency $\nu _{\mathrm {r}}$. On the other hand, particles in slightly tilted orbits fail to return to the initial displacement $\psi$ from the equatorial plane, after a full revolution around the star. This introduces a nodal precession frequency $\nu _{\mathrm {pa}}$, which is the difference between $\nu _{\mathrm {K}}$ and the frequency of the motion out of the orbital plane (meridional frequency) $\nu _{\psi }$. Explicit expressions for the above frequencies, in the gravitational field of a rapidly rotating neutron star, have been derived recently by Marković (2000), while in Marković and Lamb (2000) highly eccentric orbits are considered. Morsink and Stella (1999) compute the nodal precession frequency for a wide range of neutron star masses and equations of state and (in a post-Newtonian analysis) separate the precession caused by the Lense–Thirring (frame-dragging) effect from the precession caused by the quadrupole moment of the star. The nodal and periastron precession of inclined orbits have also been studied using an approximate analytic solution for the exterior gravitational field of rapidly rotating stars (Sibgatullin 2002). These precession frequencies are relativistic effects and have been used in several models to explain the kHz QPO frequencies (Stella et al. 1999; Psaltis and Norman 2000; Abramowicz and Kluźniak 2001; Kluźniak and Abramowicz 2002; Amsterdamski et al. 2002).

It is worth mentioning that it has recently been found that an ISCO also exists in Newtonian gravity, for models of rapidly rotating low-mass strange stars. The instability in the circular orbits is produced by the large oblateness of the star (Kluźniak et al. 2001; Zdunik and Gourgoulhon 2001; Amsterdamski et al. 2002) (see also Török et al. 2014 for a more recent study). Epicyclic frequencies for Maclaurin spheroids in Newtonian gravity have also been computed by Kluźniak and Rosińska (2013).

Epicyclic frequencies for rapidly rotating strange stars have been computed by Gondek-Rosińska et al. (2014) adopting the MIT bag model for the equation of state of quark matter. They find that the orbits around rapidly rotating strange quark stars are mostly affected by the stellar oblateness, rather than by the effects of general relativity.

For reviews on applications of current QPO models and what one can learn about the properties of NSs see Bhattacharyya (2010) , Török et al. (2010), Pappas (2012), Miller and Lamb (2016) and Özel and Freire (2016).

*Going further*:   Observations of some LMXBs finding that the difference in the frequencies of the peak QPOs is equal to half the spin frequency of the star raise some questions regarding the validity of the popular beat-frequency model (Miller et al. 1998) (but see Lamb and Miller 2003). Motivated by this tension, another model for QPOs is suggested by Li and Narayan (2004) in which it is argued that a strong magnetic field may truncate the inner parts of the disk and at the interface between the accretion disk and the magnetosphere surrounding the accreting star that gas becomes Rayleigh–Taylor and, possible also, Kelvin–Helmholtz unstable, leading to nonaxisymmetric structures which result in the high-frequency QPOs that can explain observations. For other studies considering the impact of magnetic fields see also Kluźniak and Rappaport (2007), Kulkarni and Romanova (2008), Tsang and Lai (2009), Lovelace et al. (2009), Bakala et al. (2010), Bakala et al. (2012), Fu and Lai (2012), Romanova et al. (2012), Long et al. (2012) and references therein. Another model that can explain the observations where the difference in the frequencies of the twin peaks is equal to half the spin frequency of the star is the so-called non-linear resonance model (Kluźniak et al. 2004) (see also Lee et al. 2004; Horák et al. 2009; Urbanec et al. 2010). For a recent work investigating the compatibility of realistic neutron star equations of state with several QPO modes see Török (2016). Constants of motion in stationary, axisymmetric spacetimes have been investigated recently in Markakis (2014).

### Angular momentum conservation during burst oscillations

Some sources in LMXBs show signatures of type I X-ray bursts, which are thermonuclear bursts on the surface of the compact star (Lewin et al. 1995). Such bursts show nearly-coherent oscillations in the range 270–620 Hz (see van der Klis 2000; Strohmayer 2001; Strohmayer and Bildsten 2006; Watts 2012 for reviews). One interpretation of the burst oscillations is that they are the result of rotational modulation of surface asymmetries during the burst. In such a case, the oscillation frequency should be nearly equal to the spin frequency of the star. This model currently has difficulties in explaining some observed properties, such as the oscillations seen in the tail of the burst, the frequency increase during the burst, and the need for two anti-podal hot spots in some sources that ignite at the same time. Alternative models also exist (see, e.g., Psaltis 2001).

Changes in the oscillation frequency by a few Hz during bursts have been associated with expansion and contraction of the burning shell. Cumming et al. (2002) compute the expected spin changes in general relativity taking into account rapid rotation. Assuming that the angular momentum per unit mass is conserved, the change in angular velocity with radius is given by

122 $$\begin{aligned} \frac{d\ln \varOmega }{d\ln r} = - 2 \left[ \left( 1-\frac{v^2}{2}-\frac{R}{2} \frac{\partial \nu }{\partial r} \right) \left( 1-\frac{\omega }{\varOmega }\right) - \frac{R}{2\varOmega }\frac{\partial \omega }{\partial r}\right] , \end{aligned}$$

where *R* is the equatorial radius of the star and all quantities are evaluated at the equator. The slow rotation limit of the above result was derived previously by Abramowicz et al. (2001). The fractional change in angular velocity can then be estimated as

123 $$\begin{aligned} \frac{\varDelta \varOmega }{\varOmega } = \frac{d\ln \varOmega }{d\ln r}\left( \frac{\varDelta r}{R}\right) , \end{aligned}$$

where $\varDelta r$ is the coordinate expansion of the burning shell, a quantity that depends on the shell’s composition. Cumming et al. find that in the expansion phase the expected spin down is a factor of two to three times smaller than observed values, if the atmosphere rotates rigidly. More detailed modeling is needed to fully explain the origin and properties of burst oscillations (see Watts 2012 for a recent review on theoretical models of thermonuclear bursts).

A very interesting topic is modeling the expected X-ray spectrum of an accretion disk in the gravitational field of a rapidly rotating neutron star or of the “hot spot” on its surface as it could lead to observational constraints on the source of the gravitational field. See, e.g., Thampan and Datta (1998), Sibgatullin and Sunyaev (1998), Sibgatullin and Sunyaev (2000), Bhattacharyya (2002), Bhattacharyya (2001), where work initiated by Kluźniak and Wilson (1991) in the slow rotation limit is extended to rapidly rotating relativistic stars.

Following an earlier work which uses approximate spacetimes (Cadeau et al. 2005), light curves from ray-tracing on spacetimes corresponding to realistic models of rapidly rotating neutron stars (generated with the RNS code) are obtained by Cadeau et al. (2007) assuming that the X-ray photons arise from a hot spot on the NS. There it was shown that the dominant effect due to rotation comes from the stellar oblateness, and that approximating a rapidly rotating star as a sphere results in large errors if one is trying to fit for the radius and mass. However, for cases with stellar spin frequencies $<\,\sim 300\,\mathrm {Hz}$ rapidly rotating spacetime models are not necessary and only the stellar oblateness has to be taken into consideration. As a result, Morsink et al. (2007) develop the Oblate Schwarzschild (OS) model in which photons emerge from a hot spot in the NS oblate surface, and they reach the observer following the geodesics of a corresponding Schwarzschild spacetime, while doppler effects due to rotation are taken into consideration as in the standard model of Miller and Lamb (1998). Morsink et al. demonstrate that the OS model suffices to describe the effects due to the NS rotation. An approximate analytic model for pulse profiles taking into account gravitational light bending, doppler effect, anisotropic emission and time delays is presented by Poutanen and Beloborodov (2006). Another simple model adopting the Hartle–Thorne approximation for generating pulse profiles from rotating neutron stars is developed by Psaltis and Özel (2014). For related studies see also Lee and Strohmayer (2005), Bauböck et al. (2012), Ck et al. (2013), Miller and Lamb (2015) and references therein.

*Going further*: A number of theoretical works whose aim to model atomic lines in NS atmospheres in order to infer the NS properties from the atomic line redshift see, e.g., (Özel and Psaltis 2003; Bildsten et al. 2003; Chang et al. 2005, 2006; Bhattacharyya et al. 2006; Bauböck et al. 2013; Özel 2013; Heinke 2013; Bauböck et al. 2015).

## Oscillations and stability

The study of oscillations of relativistic stars is motivated by the prospect of detecting such oscillations in electromagnetic or gravitational wave signals. In the same way that helioseismology is providing us with information about the interior of the Sun, the observational identification of oscillation frequencies of relativistic stars could constrain the high-density equation of state (Andersson and Kokkotas 1996). The oscillations could be excited after a core collapse or during the final stages of a neutron star binary merger. Rapidly rotating relativistic stars can become unstable to the emission of gravitational waves.

When the displacement due to the oscillations of an equilibrium star are small compared to its radius, it will suffice to approximate them as linear perturbations. Such perturbations can be described in two equivalent ways. In the Lagrangian approach, one studies the changes in a given fluid element as it oscillates about its equilibrium position. In the Eulerian approach, one studies the change in fluid variables at a fixed point in space. Both approaches have their strengths and weaknesses.

In the Newtonian limit, the Lagrangian approach has been used to develop variational principles (Lynden-Bell and Ostriker 1967; Friedman and Schutz 1978), but the Eulerian approach proved to be more suitable for numerical computations of mode frequencies and eigenfunctions (Ipser and Managan 1985; Managan 1985; Ipser and Lindblom 1990, 1991b, a). Clement (1981) used the Lagrangian approach to obtain axisymmetric normal modes of rotating stars, while nonaxisymmetric solutions were obtained in the Lagrangian approach by Imamura et al. (1985) and in the Eulerian approach by Managan (1985) and Ipser and Lindblom (1990). While a lot has been learned from Newtonian studies, in the following we will focus on the relativistic treatment of oscillations of rotating stars.

### Quasi-normal modes of oscillation

A general linear perturbation of the energy density in a static and spherically symmetric relativistic star can be written as a sum of quasi-normal modes that are characterized by the indices (*l*, *m*) of the spherical harmonic functions $Y_l^m$ and have angular and time dependence of the form

124 $$\begin{aligned} \delta \varepsilon \sim f(r) P_l^m(\cos \theta ) e^{i(m\phi +\omega _{\mathrm {i}} t)}, \end{aligned}$$

where $\delta$ indicates the Eulerian perturbation of a quantity, $\omega _{\mathrm {i}}$ is the angular frequency of the mode as measured by a distant inertial observer, *f*(*r*) represents the radial dependence of the perturbation, and $P_l^m(\cos \theta )$ are the associated Legendre polynomials. Normal modes of nonrotating stars are degenerate in *m* and it suffices to study the axisymmetric $(m=0)$ case.

The Eulerian perturbation in the fluid 4-velocity $\delta u^a$ can be expressed in terms of vector harmonics, while the metric perturbation $\delta g_{ab}$ can be expressed in terms of spherical, vector, and tensor harmonics. These are either of “polar” or “axial” parity. Here, parity is defined to be the change in sign under a combination of reflection in the equatorial plane and rotation by $\pi$. A polar perturbation has parity $(-\,1)^l$, while an axial perturbation has parity $(-\,1)^{l+1}$. Because of the spherical background, the polar and axial perturbations of a nonrotating star are completely decoupled.

A normal mode solution satisfies the perturbed gravitational field equation,

125 $$\begin{aligned} \delta (G^{ab}-8 \pi T^{ab})=0, \end{aligned}$$

and the perturbation of the conservation of the stress-energy tensor,

126 $$\begin{aligned} \delta (\nabla _aT^{ab})=0, \end{aligned}$$

with suitable boundary conditions at the center of the star and at infinity. The latter equation is decomposed into an equation for the perturbation in the energy density $\delta \varepsilon$ and into equations for the three spatial components of the perturbation in the 4-velocity $\delta u^a$. As linear perturbations have a gauge freedom, at most six components of the perturbed field equation (125) need to be considered.

For a given pair (*l*, *m*), a solution exists for any value of the frequency $\omega _{\mathrm {i}}$, consisting of a mixture of ingoing and outgoing wave parts. Outgoing quasi-normal modes are defined by the discrete set of eigenfrequencies for which there are no incoming waves at infinity. These are the modes that will be excited in various astrophysical situations.

The main modes of pulsation that are known to exist in relativistic stars have been classified as follows ($f_0$ and $\tau _0$ are typical frequencies and damping times of the most important modes in the nonrotating limit):

1. *Polar fluid modes* are slowly damped modes analogous to the Newtonian fluid pulsations:
*f*(undamental)-modes: surface modes due to the interface between the star and its surroundings ($f_0 \sim 2 \,\mathrm {kHz}$, $\tau _0<1 \,\mathrm {s}$),
*p*(ressure)-modes: nearly radial ($f_0 > 4 \,\mathrm {kHz}$, $\tau _0 > 1 \,\mathrm {s}$),
*g*(ravity)-modes: nearly tangential, degenerate at zero frequency in nonrotating isentropic stars; they have nonzero frequencies in stars that are non-isentropic or that have a composition gradient or a first order phase transition ($f_0<500 \,\mathrm {Hz}$, $\tau _0 > 5 \,\mathrm {s}$).
2. *Axial and hybrid fluid modes*:
*inertial* modes: degenerate at zero frequency in nonrotating stars. In a rotating star, some inertial modes are generically unstable to the CFS instability; they have frequencies from zero to kHz and growth times inversely proportional to a high power of the star’s angular velocity. Hybrid inertial modes have both axial and polar parts even in the limit of no rotation.
*r*(otation)-modes: a special case of inertial modes that reduce to the classical axial *r*-modes in the Newtonian limit. Generically unstable to the CFS instability with growth times as short as a few seconds at high rotation rates.
3. *Polar and axial spacetime modes*:
*w*(ave)-modes: Analogous to the quasi-normal modes of a black hole (very weak coupling to the fluid). High frequency, strongly damped modes ($f_0>6 \,\mathrm {kHz}$, $\tau _0 \sim 0.1 \,\mathrm {ms}$).

For a more detailed description of various types of oscillation modes, see Kokkotas (1997b, 1997a), McDermott et al. (1988), Carroll et al. (1986) and Kokkotas and Schmidt (1999).

### Effect of rotation on quasi-normal modes

In a continuous sequence of rotating stars that includes a nonrotating member, a quasi-normal mode of index *l* is defined as the mode which, in the nonrotating limit, reduces to the quasi-normal mode of the same index *l*. Rotation has several effects on the modes of a corresponding nonrotating star:

1. The degeneracy in the index *m* is removed and a nonrotating mode of index *l* is split into $2l+1$ different (*l*, *m*) modes.
2. Prograde ($m<0$) modes are now different from retrograde ($m>0$) modes.
3. A rotating “polar” *l*-mode consists of a sum of purely polar and purely axial terms (Stergioulas 1996), e.g., for $l=m$,
  127 $$\begin{aligned} P_l^{\mathrm {rot}} \sim \sum _{l'=0}^\infty (P_{l+2l'} +A_{l+2l' \pm 1}), \end{aligned}$$
  that is, rotation couples a polar *l*-term to an axial $l \pm 1$ term (the coupling to the $l+1$ term is, however, strongly favoured over the coupling to the $l-1$ term, Chandrasekhar and Ferrari 1991). Similarly, for a rotating “axial” mode with $l=m$,
  128 $$\begin{aligned} A_l^{\mathrm {rot}} \sim \sum _{l'=0}^\infty (A_{l+2l'} +P_{l+2l' \pm 1}). \end{aligned}$$
4. Frequencies and damping times are shifted. In general, frequencies (in the inertial frame) of prograde modes increase, while those of retrograde modes decrease with increasing rate of rotation.
5. In rapidly rotating stars, *apparent intersections* between higher order modes of different *l* can occur. In such cases, the shape of the eigenfunction is used in the mode classification.

In rotating stars, quasi-normal modes of oscillation have been studied only in the slow rotation limit, in the post-Newtonian, and in the Cowling approximations. The solution of the fully relativistic perturbation equations for a rapidly rotating star is still a very challenging task and only recently have they been solved for zero-frequency (neutral) modes (Stergioulas 1996; Stergioulas and Friedman 1998). First frequencies of quasi-radial modes have now been obtained through 3D numerical time evolutions of the nonlinear equations (Font et al. 2002).

*Going further*:   The equations that describe oscillations of the solid crust of a rapidly rotating relativistic star are derived by Priou (1992). The effects of superfluid hydrodynamics on the oscillations of neutron stars have been investigated by several authors, see, e.g., Lindblom and Mendell (1994); Comer et al. (1999), Andersson and Comer (2001), Andersson et al. (2002), Andersson et al. (2004), Prix et al. (2004), Sidery et al. (2008), Samuelsson and Andersson (2009), Passamonti et al. (2009a), Andersson et al. (2011), Passamonti and Andersson (2012), Passamonti et al. (2016) and references therein.

### Axisymmetric perturbations

#### Secular and dynamical axisymmetric instability

Along a sequence of nonrotating relativistic stars with increasing central energy density, there is always a model for which the mass becomes maximum. The maximum-mass turning point marks the onset of an instability in the fundamental radial pulsation mode of the star.

Applying the turning point theorem provided by Sorkin (1982), Friedman et al. (1988) provide a sufficient condition for a secular axisymmetric instability of rotating stars, when the mass becomes maximum along a sequence of constant angular momentum. An equivalent criterion (implied in Friedman et al. 1988) is provided by Cook et al. (1992): The secular axisymmetric instability sets in when the angular momentum becomes minimum along a sequence of constant rest mass. The instability first develops on a secular timescale that is set by the time required for viscosity to redistribute the star’s angular momentum. This timescale is long compared to the dynamical timescale and comparable to the spin-up time following a pulsar glitch. Eventually, the star encounters the onset of dynamical instability and collapses to a black hole (see Shibata et al. 2000a for recent numerical simulations). Thus, the onset of the secular instability to axisymmetric perturbations separates stable neutron stars from neutron stars that will undergo collapse to a black hole. More recently, Takami et al. (2011) investigated the dynamical stability of rotating stars, computing numerically the neutral point of the fundamental, quasi-radial *F*-mode frequency, which signals the onset of the dynamical stability. In their simulations, they found that the *F*-mode frequency can go through zero before a star reaches turning point. Prabhu et al. (2016) investigate the axisymmetric stability of rotating relativistic stars through a variational principle and show that the sign of the canonical energy gives a necessary and sufficient condition for dynamical instability. In addition, they determine a lower bound for exponential growth.

Goussard et al. (1997) extend the stability criterion to hot proto-neutron stars with nonzero total entropy. In this case, the loss of stability is marked by the configuration with minimum angular momentum along a sequence of both constant rest mass and total entropy. In the nonrotating limit, Gondek et al. (1997) compute frequencies and eigenfunctions of radial pulsations of hot proto-neutron stars and verify that the secular instability sets in at the maximum mass turning point, as is the case for cold neutron stars.

**Fig. 10:**
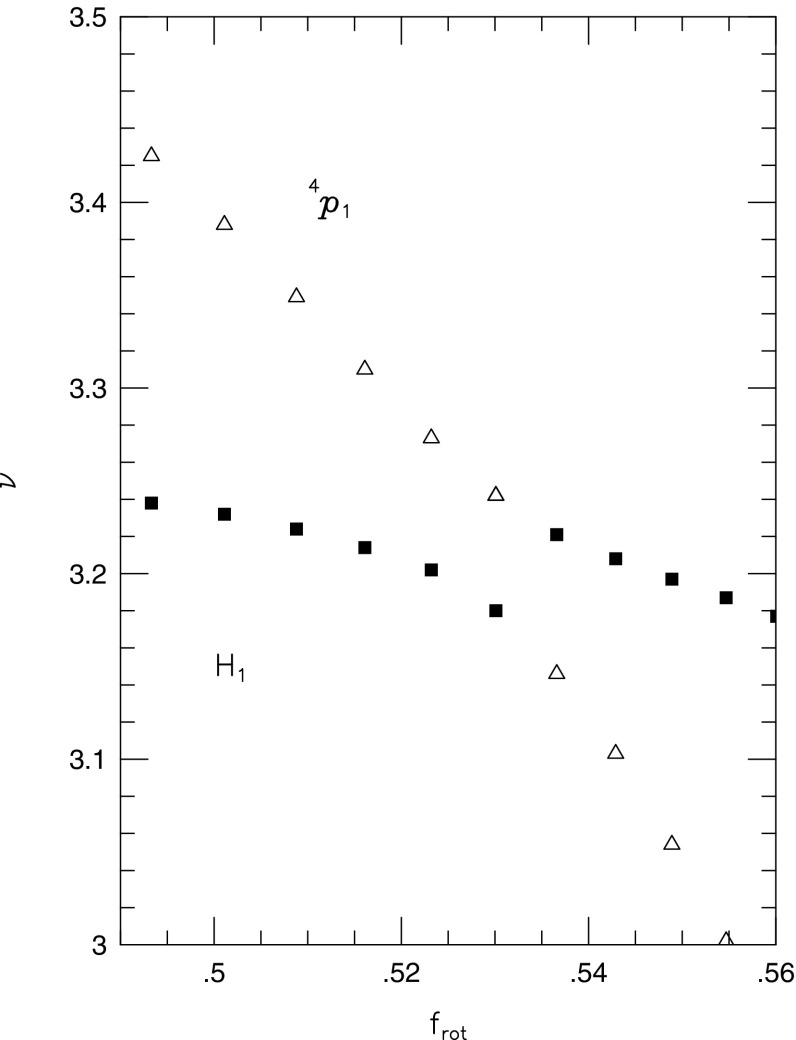
Apparent intersection (due to avoided crossing) of the axisymmetric first quasi-radial overtone ($H_1$) and the first overtone of the $l=4$ *p*-mode (in the Cowling approximation). Frequencies are normalized by $\sqrt{\rho _{\mathrm {c}}/4\pi }$, where $\rho _{\mathrm {c}}$ is the central energy density of the star. The rotational frequency $f_{\mathrm {rot}}$ at the mass-shedding limit is 0.597 (in the above units). Along continuous sequences of computed frequencies, mode eigenfunctions are exchanged at the avoided crossing. Defining quasi-normal mode sequences by the shape of their eigenfunction, the $H_1$ sequence (filled boxes) appears to intersect with the ${}^4p_1$ sequence (triangle), but each sequence shows a discontinuity, when the region of apparent intersection is well resolved. In the notation ${}^l\mathrm{mode}_n$, the superscript indicates the *l* of the perturbation, while the subscript indicates the harmonic overtone. (Image reproduced with permission from Yoshida and Eriguchi 2001, copyright by RAS)

#### Axisymmetric pulsation modes

Axisymmetric ($m=0$) pulsations in rotating relativistic stars could be excited in a number of different astrophysical scenarios, such as during core collapse, in star quakes induced by the secular spin-down of a pulsar or during a large phase transition, or in the merger of two relativistic stars in a binary system, among others. Due to rotational couplings, the eigenfunction of any axisymmetric mode will involve a sum of various spherical harmonics $Y_l^0$, so that even the quasi-radial modes (with lowest order $l=0$ contribution) would, in principle, radiate gravitational waves.

Quasi-radial modes in rotating relativistic stars have been studied by Hartle and Friedman (1975) and by Datta et al. (1998) in the slow rotation approximation. Yoshida and Eriguchi (2001) study quasi-radial modes of rapidly rotating stars in the relativistic Cowling approximation and find that apparent intersections between quasi-radial and other axisymmetric modes can appear near the mass-shedding limit (see Fig. 10). These apparent intersections are due to *avoided crossings* between mode sequences, which are also known to occur for axisymmetric modes of rotating Newtonian stars. Along a continuous sequence of computed mode frequencies an avoided crossing occurs when another sequence is encountered. In the region of the avoided crossing, the eigenfunctions of the two modes become of mixed character. Away from the avoided crossing and along the continuous sequences of computed mode frequencies, the eigenfunctions are exchanged. However, each “quasi-normal mode” is characterized by the shape of its eigenfunction and thus, the sequences of computed frequencies that belong to particular quasi-normal modes are discontinuous at avoided crossings (see Fig. 10 for more details). The discontinuities can be found in numerical calculations, when quasi-normal mode sequences are well resolved in the region of avoided crossings. Otherwise, quasi-normal mode sequences will appear as intersecting.

**Fig. 11:**
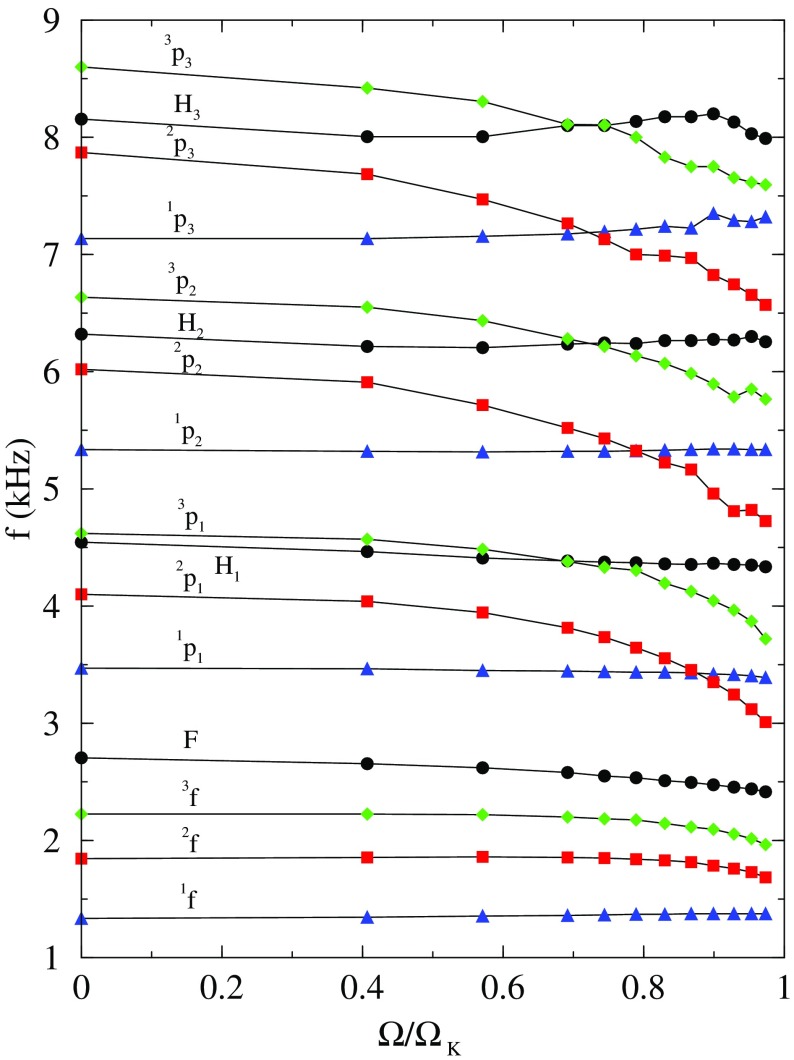
Frequencies of several axisymmetric modes along a sequence of rapidly rotating relativistic polytropes of $N=1.0$, in the Cowling approximation. On the horizontal axis, the angular velocity of each model is scaled to the angular velocity of the model at the mass-shedding limit. Lower order modes are weakly affected by rapid rotation, while higher order modes show apparent mode intersections. (Image reproduced with permission from Font et al. 2001, copyright by RAS)

Several axisymmetric modes have recently been computed for rapidly rotating relativistic stars in the Cowling approximation, using time evolutions of the nonlinear hydrodynamical equations (Font et al. 2001) (see Font et al. 2000 for a description of the 2D numerical evolution scheme). As in Yoshida and Eriguchi (2001), Font et al. (2001) find that apparent mode intersections are common for various higher order axisymmetric modes (see Fig. 11). Axisymmetric inertial modes also appear in the numerical evolutions.

The first fully relativistic frequencies of quasi-radial modes for rapidly rotating stars (without assuming the Cowling approximation) have been obtained recently, again through nonlinear time evolutions (Font et al. 2002) (see Sect. 5.2).

*Going further*:   The stabilization, by an external gravitational field, of a relativistic star that is marginally stable to axisymmetric perturbations is discussed in Thorne (1998).

### Nonaxisymmetric perturbations

#### Nonrotating limit

Thorne, Campolattaro, and Price, in a series of papers (Thorne and Campolattaro 1967; Price and Thorne 1969; Thorne 1969), initiated the computation of nonradial modes by formulating the problem in the Regge–Wheeler (RW) gauge (Regge and Wheeler 1957) and numerically computing nonradial modes for a number of neutron star models. A variational method for obtaining eigenfrequencies and eigenfunctions has been constructed by Detweiler and Ipser (1973). Lindblom and Detweiler (1983) explicitly reduced the system of equations to four first order ordinary differential equations and obtained more accurate eigenfrequencies and damping times for a larger set of neutron star models. They later realized that their system of equations is sometimes singular inside the star and obtained an improved set of equations, which is free of this singularity (Detweiler and Lindblom 1985).

Chandrasekhar and Ferrari (1991) expressed the nonradial pulsation problem in terms of a fifth order system in a diagonal gauge, which is formally independent of fluid variables. Thus, they reformulate the problem in a way analogous to the scattering of gravitational waves off a black hole. Ipser and Price (1991) show that in the RW gauge, nonradial pulsations can be described by a system of two second order differential equations, which can also be independent of fluid variables. In addition, they find that the diagonal gauge of Chandrasekhar and Ferrari has a remaining gauge freedom which, when removed, also leads to a fourth order system of equations (Price and Ipser 1991).

In order to locate purely outgoing wave modes, one has to be able to distinguish the outgoing wave part from the ingoing wave part at infinity. This is typically achieved using analytic approximations of the solution at infinity.

*W*-modes pose a more challenging numerical problem because they are strongly damped and the techniques used for *f*- and *p*-modes fail to distinguish the outgoing wave part. The first accurate numerical solutions were obtained by Kokkotas and Schutz (1992), followed by Leins et al. (1993). Andersson et al. (1995) successfully combine a redefinition of variables with a complex-coordinate integration method, obtaining highly accurate complex frequencies for *w*-modes. In this method, the ingoing and outgoing solutions are separated by numerically calculating their analytic continuations to a place in the complex-coordinate plane, where they have comparable amplitudes. Since this approach is purely numerical, it could prove to be suitable for the computation of quasi-normal modes in rotating stars, where analytic solutions at infinity are not available.

The non-availability of asymptotic solutions at infinity in the case of rotating stars is one of the major difficulties for computing outgoing modes in rapidly rotating relativistic stars. A method that may help to overcome this problem, at least to an acceptable approximation, has been found by Lindblom et al. (1997). The authors obtain approximate near-zone boundary conditions for the outgoing modes that replace the outgoing wave condition at infinity and that enable one to compute the eigenfrequencies with very satisfactory accuracy. First, the pulsation equations of polar modes in the Regge–Wheeler gauge are reformulated as a set of two second order radial equations for two potentials—one corresponding to fluid perturbations and the other to the perturbations of the spacetime. The equation for the spacetime perturbation reduces to a scalar wave equation at infinity and to Laplace’s equation for zero-frequency solutions. From these, an approximate boundary condition for outgoing modes is constructed and imposed in the near zone of the star (in fact, on its surface) instead of at infinity. For polytropic models, the near-zone boundary condition yields *f*-mode eigenfrequencies with real parts accurate to 0.01–0.1% and imaginary parts with accuracy at the 10–20% level, for the most relativistic stars. If the near zone boundary condition can be applied to the oscillations of rapidly rotating stars, the resulting frequencies and damping times should have comparable accuracy.

#### Slow rotation approximation

The slow rotation approximation is useful for obtaining a first estimate of the effect of rotation on the pulsations of relativistic stars. To lowest order in rotation, a polar *l*-mode of an initially nonrotating star couples to an axial $l \pm 1$ mode in the presence of rotation. Conversely, an axial *l*-mode couples to a polar $l \pm 1$ mode as was first discussed by Chandrasekhar and Ferrari (1991).

The equations of nonaxisymmetric perturbations in the slow rotation limit are derived in a diagonal gauge by Chandrasekhar and Ferrari (1991), and in the Regge–Wheeler gauge by Kojima (1992, 1993b), where the complex frequencies $\sigma = \sigma _R + i \sigma _I$ for the $l=m$ modes of various polytropes are computed. For counterrotating modes, both $\sigma _R$ and $\sigma _I$ decrease, tending to zero, as the rotation rate increases (when $\sigma$ passes through zero, the star becomes unstable to the CFS instability). Extrapolating $\sigma _R$ and $\sigma _I$ to higher rotation rates, Kojima finds a large discrepancy between the points where $\sigma _R$ and $\sigma _I$ go through zero. This shows that the slow rotation formalism cannot accurately determine the onset of the CFS instability of polar modes in rapidly rotating neutron stars.

In Kojima (1993a), it is shown that, for slowly rotating stars, the coupling between polar and axial modes affects the frequency of *f*- and *p*-modes only to second order in rotation, so that, in the slow rotation approximation, to $\mathcal{O}( \varOmega )$, the coupling can be neglected when computing frequencies. This result was already known from the original work of Hartle et al. (1972), where it was noted that a reversal of the direction of rotation cannot change the shape of the mode or its frequency.

The linear perturbation equations in the slow rotation approximation have been derived in a new gauge by Ruoff et al. (2002). Using the Arnowitt–Deser–Misner (ADM) formalism (Arnowitt et al. 2008), a first order hyperbolic evolution system is obtained, which is suitable for numerical integration without further manipulations (as was required in the Regge–Wheeler gauge). In this gauge (which is related to a gauge introduced for nonrotating stars in Battiston et al. 1971), the symmetry between the polar and axial equations becomes directly apparent.

The case of relativistic inertial modes is different, as these modes have both axial and polar parts at order $\mathcal{O}(\varOmega )$, and the presence of continuous bands in the spectrum (at this order in the rotation rate) has led to a series of detailed investigations of the properties of these modes (see Kokkotas and Ruoff 2003 for a review). Ruoff et al. (2003) finally show that the inclusion of both polar and axial parts in the computation of relativistic *r*-modes, at order $\mathcal{O}(\varOmega )$, allows for discrete modes to be computed, in agreement with post-Newtonian (Lockitch et al. 2001) and nonlinear, rapid-rotation (Stergioulas and Font 2001) calculations.

#### Post-Newtonian approximation

A step toward the solution of the perturbation equations in full general relativity has been taken by Cutler (1991), Cutler and Lindblom (1992), Lindblom (1995), who obtain frequencies for the $l=m$ *f*-modes in rotating stars in the first post-Newtonian (1-PN) approximation. The perturbation equations are derived in the post-Newtonian formalism (see Blanchet 2003), i.e., the equations are separated into equations of consistent order in 1 / *c*.

Cutler and Lindblom show that in this scheme, the perturbation of the 1-PN correction of the four-velocity of the fluid can be obtained analytically in terms of other variables; this is similar to the perturbation in the three-velocity in the Newtonian Ipser–Managan scheme. The perturbation in the 1-PN corrections are obtained by solving an eigenvalue problem, which consists of three second order equations, with the 1-PN correction to the eigenfrequency of a mode $\varDelta \omega$ as the eigenvalue.

Cutler and Lindblom obtain a formula that yields $\varDelta \omega$ if one knows the 1-PN stationary solution and the solution to the Newtonian perturbation equations. Thus, the frequency of a mode in the 1-PN approximation can be obtained without actually solving the 1-PN perturbation equations numerically. The 1-PN code was checked in the nonrotating limit and it was found to reproduce the exact general relativistic frequencies for stars with $M/R=0.2$, obeying an $N=1$ polytropic EOS, with an accuracy of 3–8%.

Along a sequence of rotating stars, the frequency of a mode is commonly described by the ratio of the frequency of the mode in the comoving frame to the frequency of the mode in the nonrotating limit. For an $N=1$ polytrope and for $M/R=0.2$, this frequency ratio is reduced by as much as 12% (for the fundamental $l=m$ modes) in the 1-PN approximation compared to its Newtonian counterpart—keeping the gravitational mass fixed—which is representative of the effect that general relativity has on the frequency of quasi-normal modes in rotating stars.

#### Cowling approximation

In several situations, the frequency of pulsations in relativistic stars can be estimated even if one completely neglects the perturbation in the gravitational field, i.e., if one sets $\delta g_{ab}=0$ in the perturbation equations (McDermott et al. 1983). In this approximation, the pulsations are described only by the perturbation in the fluid variables, and the scheme works quite well for *f*, *p*, and *r*-modes (Lindblom and Splinter 1990). A different version of the Cowling approximation, in which $\delta g_{tr}$ is kept nonzero in the perturbation equations, has been suggested to be more suitable for *g*-modes (Finn 1988), since these modes could have large fluid velocities, even though the variation in the gravitational field is weak.

Yoshida and Kojima (1997) examine the accuracy of the relativistic Cowling approximation in slowly rotating stars. The first order correction to the frequency of a mode depends only on the eigenfrequency and eigenfunctions of the mode in the absence of rotation and on the angular velocity of the star. The eigenfrequencies of *f*, $p_1$, and $p_2$ modes for slowly rotating stars with *M* / *R* between 0.05 and 0.2 are computed (assuming polytropic EOSs with $N=1$ and $N=1.5$) and compared to their counterparts in the slow rotation approximation.

For the $l=2$ *f*-mode, the relative error in the eigenfrequency because of the Cowling approximation is 30% for weakly relativistic stars ($M/R=0.05$) and about 15% for stars with $M/R=0.2$; the error decreases for higher *l*-modes. For the $p_1$ and $p_2$ modes the relative error is similar in magnitude but it is smaller for less relativistic stars. Also, for *p*-modes, the Cowling approximation becomes more accurate for increasing radial mode number.

**Fig. 12:**
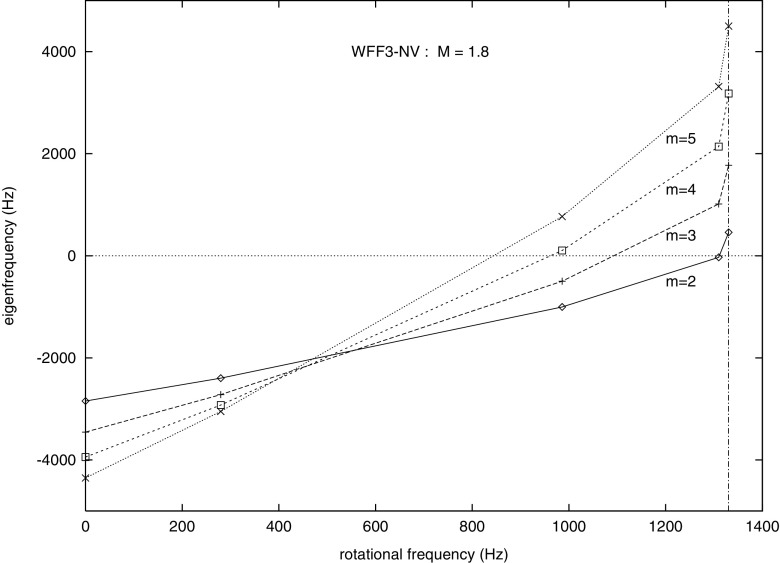
Eigenfrequencies (in the Cowling approximation) of *f*-modes along a $M = 1.8\,M_{\odot }$ sequence of models, constructed with the WFF3-NV EOS. The vertical line corresponds to the frequency of rotation of the model at the mass-shedding limit of the sequence. (Image reproduced with permission from Yoshida and Eriguchi 1999, copyright by AAS)

As an application, Yoshida and Eriguchi (1997, 1999) use the Cowling approximation to estimate the onset of the *f*-mode CFS instability in rapidly rotating relativistic stars and to compute frequencies of *f*-modes for several realistic equations of state (see Fig. 12).

The effects of rotation on the frequencies of the quasi-normal modes in the case of a proto-neutron star are studied by Ferrari et al. (2004), where the growth time of unstable g modes is estimated based on a post-Newtonian formula.

Perturbations in Newtonian axisymmetric background configurations but accounting for the superfluid hydrodynamics are studied by Passamonti et al. (2009a). Passamonti et al. (2009b) study *g*-modes for stratified rapidly rotating neutrons stars also in a Newtonian framework. In a follow-up work, Gaertig and Kokkotas (2009) extend the latter work in general relativity finding good qualitative agreement with the Newtonian results.

Yoshida et al. (2005) adopt the Cowling approximation to study *r*-mode oscillations of rapidly and rigidly rotating, barotropic, relativistic stars. Their formulation and method is the general relativistic extension of the Yoshida and Eriguchi method (Yoshida and Eriguchi 1997), which amounts to the solution of a second-order, time-indepedent partial differential equation for the eigenvalue problem. Using the method they obtain the frequencies of the *r*-mode oscillations as a function of *T* / |*W*|, and find that the normalized oscillation frequencies $\sigma /\varOmega$ (where $\varOmega$ is the stellar rotation frequency) scale almost linearly with *T* / |*W*| and decrease as *T* / |*W*| increases.

Gaertig and Kokkotas (2008, 2011) adopt the Cowling approximation to study $m=\pm 2$ nonaxisymmetric oscillations and instabilities of rapidly rotating general relativistic, polytropic stars using a time-dependent approach for the first time. They introduce a formulation for the linearized equations of motion for a perfect fluid appropriate for a rapidly rotating star in a comoving frame using surface fitted coordinates. The equations of state they adopt have $(\varGamma ,K)=(2,100)$ (labeled as the EOS BU), $(\varGamma ,K)=(2.46,0.00936)$ (labeled as the EOS A), and $(\varGamma ,K)=(2.34,0.0195)$ (labeled as the EOS II). The values for *K* are in geometrized units with $M_\odot =1$. From the BU EOS they adopt a neutron star model with $M=1.4\,M_{\odot }$ and circumferential radius $R=14.15$ km. For the A (II) EOS they consider a model with $M=1.61\,M_{\odot }$ ($M=1.91\,M_{\odot }$) and $R=9.51$ km ($R=11.68$ km).

For $l=2$ polar perturbations, Gaertig and Kokkotas find the anticipated splitting of counter-rotating $m=2$ and corotating $m=-2$ f modes as the star is set to rotate at higher rates. They find that even for rapidly rotating stars the higher the compactness of the star the higher the *f*-mode frequency. They also discover an equation-of-state independent fit for the *f*-mode frequencies ($\sigma _0$) in the corotating frame as a function of the rotation frequency as follows

129 $$\begin{aligned} \frac{\sigma }{\sigma _0} = 1.0 + C_{lm}^{(1)}\left( \frac{\varOmega }{\varOmega _K}\right) + C_{lm}^{(2)}\left( \frac{\varOmega }{\varOmega _K}\right) ^2, \end{aligned}$$

where $\sigma _0$ is the *f*-mode frequency for a nonrotating star and $\varOmega _K$ the mass-shedding limit rotation frequency. From the numerical calculations they find $C_{2-2}^{(1)} = -0.27$, $C_{2-2}^{(2)} = -0.34$ and $C_{22}^{(1)} = 0.47$, $C_{22}^{(2)} = -0.51$. To transform these frequencies to the stationary frame one must use $\sigma _\mathrm{stat} = \sigma _\mathrm{corot} -m\varOmega$. Through this equation the authors compute the stationary frame frequencies and are able to determine the critical rotation rate at which $\sigma _\mathrm{stat}=0$ for $m=2$. For rotation rates higher than the critical one the *f*-mode is retrograde in the corotating frame, and prograde in the stationary frame, hence the mode becomes unstable to the Chandrasekhar–Friedman–Schutz instability (Chandrasekhar 1970; Friedman and Schutz 1978) (see next section). The authors are able to also study nonaxisymmetric axial $l=2,m=2$ perturbations, i.e., *r*-modes, finding results that are in excellent agreement with earlier results from nonlinear general-relativistic simulations by Stergioulas and Font (2001).

In follow up work, Krüger et al. (2010) adopt the Cowling approximation generalizing the approach of Gaertig and Kokkotas (2008) to investigate nonaxisymmetric oscillations of rapidly and differentially rotating relativistic stars. Adopting polytropic equations of state, the authors find that for nonaxisymmetric *f*-modes the higher the degree of differential rotation the neutral point is reached at a lower value of *T* / |*W*|, hence differential rotation favors the development of the Chandrasekhar–Friedman–Schutz instability. Krüger et al. also study *r*-mode oscillations for which they find a discrete spectrum only, in contrast to some earlier studies (Kojima 1998; Passamonti et al. 2008) that found evidence for a continuum spectrum (see also discussion in the next section).

**Fig. 13:**
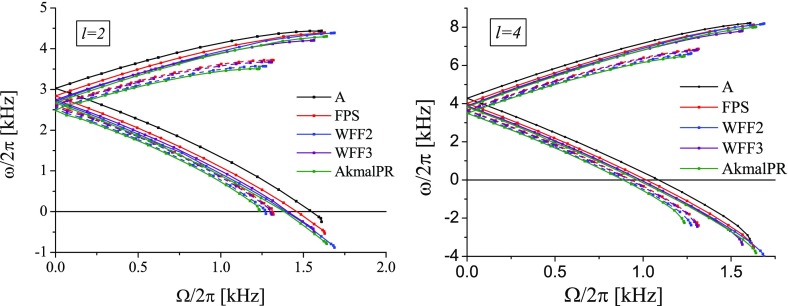
*f*-mode frequencies in the inertial frame as a function of the stellar rotation angular frequency for different EOSs labeled as A, FPS, WFF2, WFF3 and AkmalPR (see Doneva et al. 2013a for more details). Left: $l = | m | = 2$. Right: $l = | m | = 4$. (Image reproduced with permission from Doneva et al. 2013a, copyright by APS)

A substantial step forward in the field of gravitational wave asteroseismology is taken by the work of Doneva et al. (2013a) who extend the work by Gaertig and Kokkotas (2008) in several different ways: (a) they consider realistic equations of state, (b) they treat higher modes up to $l=m=4$, (c) they address the problem of inferring the properties of a neutron star from observations of observed frequencies and damping timescales using *f*-modes $l>2$. The authors use the rns code to build the equilibrium rotating configurations adopting five realistic EOSs, constructing two constant-central-density rotational sequences up to the mass-shedding limit for most of them. For each EOS, the first sequence starts with a non-spinning star with mass $1.4\,M_{\odot }$, and the second with a non-spinning star near the TOV limit. The characteristic mode splitting of the *f*-modes for different values of *m* as the stars are spun-up can be seen in Fig. 13 with the upper branch being the stable one and the lower branch being (potentially) unstable.

When normalizing the oscillation frequencies in the corotating frame as in Eq. (129), they find that Eq. (129) is still a good approximation with interpolation parameters for the unstable modes given by

- $$l=m=2$$: $$C_{22}^{(1)} = 0.402$$ and $$C_{22}^{(2)} = -0.406$$, with $$\frac{\sigma _{0,l=2}}{2\pi }[\mathrm {kHz}]=1.562+1.151\left( \frac{\bar{M}_0}{\bar{R}_0^3}\right) ^{1/2}$$.
- $$l=m=3$$: $$C_{33}^{(1)} = 0.373$$ and $$C_{33}^{(2)} = -0.485$$, with $$\frac{\sigma _{0,l=3}}{2\pi }[\mathrm {kHz}]=1.764+1.577\left( \frac{\bar{M}_0}{\bar{R}_0^3}\right) ^{1/2}$$.
- $$l=m=4$$: $$C_{44}^{(1)} = 0.360$$ and $$C_{44}^{(2)} = -0.543$$, with $$\frac{\sigma _{0,l=4}}{2\pi }[\mathrm {kHz}]=1.958+1.898\left( \frac{\bar{M}_0}{\bar{R}_0^3}\right) ^{1/2}$$.

In the expressions for $\sigma _{0,l}$ above the mass and radius are normalized as $\bar{M}_0= M_0/1.4\,M_{\odot }$ and $\bar{R}_0= R_0/10\,\mathrm {km}$, and stand for the masses and radii of the nonspining configurations, respectively. Interestingly, Doneva et al. find a universal fitting relation for all stable-mode ($l=-m=2$, $l=-m=3$ and $l=-m=4$) oscillation frequencies, where the coefficients in Eq. (129) are given by $C_{lm}^{(1)} = -0.235$ and $C_{lm}^{(2)} = -0.358$. The Kepler limit $\varOmega _K$ in Eq. (129) is well described (within 2% accuracy) by $(1/2\pi )\varOmega _K[\mathrm {kHz}]= 1.716(\bar{M}_0/\bar{R}_0^3)^{1/2}-0.189$. Finally, the authors find that the masses and radii of the rotating configurations are well-described as functions of the masses and radii of the non-spinning counterparts and the angular frequency by the following expressions

130 $$\begin{aligned} \frac{M}{M_0}=0.991+9.36\times 10^{-3}e^{3.28\frac{\varOmega }{\varOmega _K}} \end{aligned}$$

and

131 $$\begin{aligned} \frac{R}{R_0}=0.997+2.77\times 10^{-3}e^{4.74\frac{\varOmega }{\varOmega _K}} \end{aligned}$$

Doneva et al. (2013a) also provide approximate relations for the damping (growth) timescale of the stable (unstable) modes. These are relations are approximate because the authors adopt the Cowling approximation and as a result they can only estimate the gravitational wave damping timescale. The expressions are given in the form

132 $$\begin{aligned} \left( \frac{\tau _l}{\tau _0}\right) ^{1/2l}=\sum _{n=0}^3 c_{ln}\left( \frac{\sigma }{\sigma _0}\right) ^n, \ \ l=2,3,4 \end{aligned}$$

which the authors argue will remain valid even if the Cowling approximation is lifted because these involve properly rescaled quantities. The authors also provide fits for non-spinning limit $\tau _0$ for the different l modes which scale as $\tau _0^{-1} = (1/\bar{R}_0)(\bar{M}_0/\bar{R}_0)^{l+1}(\bar{c}_{l0}+\bar{c}_{l1}(\bar{M}_0/\bar{R}_0)$. With these approximately EOS-independent expressions for the *f*-mode frequencies and damping timescales, one can obtain the mass and the radius of a rotating neutron star following a determination of the nonrotating-limit parameters. However, as the authors point out, not all combinations of frequencies and damping times are capable of providing information about the neutron star mass and radius. For example, measuring two frequencies alone can provide information for $\varOmega$ and $M/R^3$, but not of *M* and *R* separately. Therefore, measuring the damping timescale of at least one *f*-mode is necessary to break the degeneracy and estimate *M* and *R* separately. But, this is going to be a rather challenging task because of the long integration times required in noisy detector data.

*Going further*: A new approach for performing asteroseismology for neutron stars was introduced by Doneva and Kokkotas (2015). The *f*-mode oscillation frequencies in modified gravity theories have recently been addressed by Staykov et al. (2015). The authors adopt the Cowling approximation and treat $R^2$ gravity as a first case. The authors derive the $R^2$-gravity asteroseismology relations which they find they are approximately EOS-independent as in the case of general relativity. By varying the $R^2$-gravity coupling constant within the range allowed by current observations, the authors estimate that the $R^2$-gravity asteroseismology relations deviate from those in general relativity by up to 10%. This implies that it will be difficult to further constrain $R^2$-gravity via gravitational wave asteroseismology. A study of *f*-modes of rapidly rotating stars in a scalar–tensor theory of gravity are studied for the first time by Yazadjiev et al. (2017).

### Nonaxisymmetric instabilities

#### Introduction

Rotating cold neutron stars, detected as pulsars, have a remarkably stable rotation period. But, at birth or during accretion, rapidly rotating neutron stars can be subject to various nonaxisymmetric instabilities, which will affect the evolution of their rotation rate.

If a proto-neutron star has a sufficiently high rotation rate (so that, e.g., $T/W > 0.27$ in the case of Maclaurin spheroids), it will be subject to a dynamical instability driven by hydrodynamics and gravity, typically referred to as the dynamical bar-mode instability. Through the $l=2$ mode, the instability will deform the star into a bar shape. This highly nonaxisymmetric configuration will emit strong gravitational waves with frequencies in the kHz regime. The development of the instability and the resulting waveform have been computed numerically in the context of Newtonian gravity by Houser et al. (1994) and in full general relativity by Shibata et al. (2000a) (see Sect. 5.1.3).

At lower rotation rates, the star can become unstable to secular nonaxisymmetric instabilities, driven by gravitational radiation or viscosity. Gravitational radiation drives a nonaxisymmetric instability when a mode that is retrograde in a frame corotating with the star appears as prograde to a distant inertial observer, via the Chandrasekhar–Friedman–Schutz (CFS) mechanism (Chandrasekhar 1970; Friedman and Schutz 1978): A mode that is retrograde in the corotating frame has negative angular momentum, because the perturbed star has less angular momentum than the unperturbed one. If, for a distant observer, the mode is prograde, it removes positive angular momentum from the star, and thus the angular momentum of the mode becomes increasingly negative.

The instability evolves on a secular timescale, during which the star loses angular momentum via the emitted gravitational waves. When the star rotates more slowly than a critical value, the mode becomes stable and the instability proceeds on the longer timescale of the next unstable mode, unless it is suppressed by viscosity.

Neglecting viscosity, the CFS instability is generic in rotating stars for both polar and axial modes. For polar modes, the instability occurs only above some critical angular velocity, where the frequency of the mode goes through zero in the inertial frame. The critical angular velocity is smaller for increasing mode number *l*. Thus, there will always be a high enough mode number *l* for which a slowly rotating star will be unstable. Many of the hybrid inertial modes (and in particular the relativistic *r*-mode) are generically unstable in all rotating stars, since the mode has zero frequency in the inertial frame when the star is nonrotating (Andersson 1998; Friedman and Morsink 1998).

The shear and bulk viscosity of neutron star matter is able to suppress the growth of the CFS instability except when the star passes through a certain temperature window. In Newtonian gravity, it appears that the polar mode CFS instability can occur only in nascent neutron stars that rotate close to the mass-shedding limit (Ipser and Lindblom 1991b, a, 1992; Yoshida and Eriguchi 1995; Lindblom and Mendell 1995), but the computation of neutral *f*-modes in full relativity (Stergioulas 1996; Stergioulas and Friedman 1998) shows that relativity enhances the instability, allowing it to occur in stars with smaller rotation rates than previously thought.

*Going further*:   A numerical method for the analysis of the ergosphere instability in relativistic stars, which could be extended to nonaxisymmetric instabilities of fluid modes, is presented by Yoshida and Eriguchi (1996).

#### CFS instability of polar modes

The existence of the CFS instability in rotating stars was first demonstrated by Chandrasekhar (1970) in the case of the $l=2$ mode in uniformly rotating, uniform density Maclaurin spheroids. Friedman and Schutz (1978) show that this instability also appears in compressible stars and that all rotating, self-gravitating perfect fluid configurations are generically unstable to the emission of gravitational waves. In addition, they find that a nonaxisymmetric mode becomes unstable when its frequency vanishes in the inertial frame. Thus, zero-frequency outgoing modes in rotating stars are neutral (marginally stable).

In the Newtonian limit, neutral modes have been determined for several polytropic EOSs (Imamura et al. 1985; Managan 1985; Ipser and Lindblom 1990; Yoshida and Eriguchi 1995). The instability first sets in through $l=m$ modes. Modes with larger *l* become unstable at lower rotation rates, but viscosity limits the interesting ones to $l \le 5$. For an $N=1$ polytrope, the critical values of *T* / *W* for the $l=3,4$, and 5 modes are 0.079, 0.058, and 0.045, respectively, and these values become smaller for softer polytropes. The $l=m=2$ “bar” mode has a critical *T* / *W* ratio of 0.14 that is almost independent of the polytropic index. Since soft EOSs cannot produce models with high *T* / *W* values, the bar mode instability appears only for stiff Newtonian polytropes of $N \le 0.808$ (James 1964; Skinner and Lindblom 1996). In addition, the viscosity-driven bar mode appears at the same critical *T* / *W* ratio as the bar mode driven by gravitational radiation (Ipser and Managan 1985) (we will see later that this is no longer true in general relativity).

The post-Newtonian computation of neutral modes by Cutler and Lindblom (1992), Lindblom (1995) has shown that general relativity tends to strengthen the CFS instability. Compared to their Newtonian counterparts, critical angular velocity ratios $\varOmega _{\mathrm {c}}/\varOmega _0$ (where $\varOmega _0=(3M_0/4R_0^3)^{1/2}$, and $M_0$, $R_0$ are the mass and radius of the nonrotating star in the sequence) are lowered by as much as 10% for stars obeying the $N=1$ polytropic EOS (for which the instability occurs only for $l=m \ge 3$ modes in the post-Newtonian approximation).

**Fig. 14:**
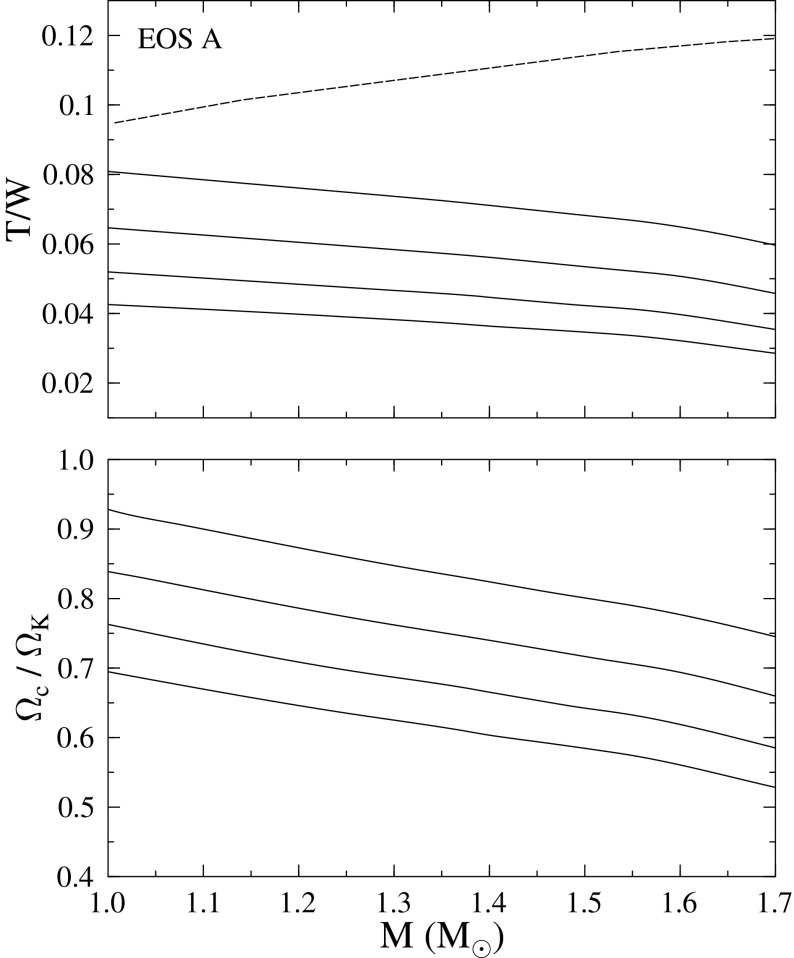
The $l=m$ neutral *f*-mode sequences for EOS A. Shown are the ratio of rotational to gravitational energy *T* / *W* (upper panel) and the ratio of the critical angular velocity $\varOmega _{\mathrm {c}}$ to the angular velocity at the mass-shedding limit for uniform rotation (lower panel) as a function of gravitational mass. The solid curves are the neutral mode sequences for $l=m=2, 3, 4$, and 5 (from top to bottom), while the dashed curve in the upper panel corresponds to the mass-shedding limit for uniform rotation. The $l=m=2$ *f*-mode becomes CFS-unstable even at 85% of the mass-shedding limit, for $1.4\,M_{\odot }$ models constructed with this EOS. (Image reproduced with permission from Morsink et al. 1999, copyright by AAS)

In full general relativity, neutral modes have been determined for polytropic EOSs of $N \ge 1.0$ by Stergioulas (1996), Stergioulas and Friedman (1998), using a new numerical scheme. The scheme completes the Eulerian formalism developed by Ipser and Lindblom (1992) in the Cowling approximation (where $\delta g_{ab}$ was neglected), by finding an appropriate gauge in which the time independent perturbation equations can be solved numerically for $\delta g_{ab}$. The computation of neutral modes for polytropes of $N=1.0$, 1.5, and 2.0 shows that relativity significantly strengthens the instability. For the $N=1.0$ polytrope, the critical angular velocity ratio $\varOmega _{\mathrm {c}} / \varOmega _{\mathrm {K}}$, where $\varOmega _{\mathrm {K}}$ is the angular velocity at the mass-shedding limit at same central energy density, is reduced by as much as 15% for the most relativistic configuration (see Fig. 14). A surprising result (which was not found in computations that used the post-Newtonian approximation) is that the $l=m=2$ bar mode is unstable even for relativistic polytropes of index $N=1.0$. The classical Newtonian result for the onset of the bar mode instability ($N_{\mathrm {crit}} <0.808$) is replaced by

133 $$\begin{aligned} N_{\mathrm {crit}} < 1.3 \end{aligned}$$

in general relativity. For relativistic stars, it is evident that the onset of the gravitational-radiation-driven bar mode does not coincide with the onset of the viscosity-driven bar mode, which occurs at larger *T* / *W* (Bonazzola et al. 1998). The computation of the onset of the CFS instability in the relativistic Cowling approximation by Yoshida and Eriguchi (1997) agrees qualitatively with the conclusions in Stergioulas (1996), Stergioulas and Friedman (1998).

Morsink et al. (1999) extend the method presented in Stergioulas and Friedman (1998) to a wide range of realistic equations of state (which usually have a stiff high density region, corresponding to polytropes of index $N=0.5$–0.7) and find that the $l=m=2$ bar mode becomes unstable for stars with gravitational mass as low as 1.0–$1.2\,M_{\odot }$. For $1.4\,M_{\odot }$ neutron stars, the mode becomes unstable at 80–95% of the maximum allowed rotation rate. For a wide range of equations of state, the $l=m=2$ *f*-mode becomes unstable at a ratio of rotational to gravitational energies $T/W \sim 0.08$ for $1.4\,M_{\odot }$ stars and $T/W \sim 0.06$ for maximum mass stars. This is to be contrasted with the Newtonian value of $T/W \sim 0.14$. The empirical formula

134 $$\begin{aligned} \left( T/W \right) _2 = 0.115\,-\,0.048 \frac{M}{M_{\mathrm {max}}^{\mathrm {sph}}}, \end{aligned}$$

where $M_{\mathrm {max}}^{\mathrm {sph}}$ is the maximum mass for a spherical star allowed by a given equation of state, gives the critical value of *T* / *W* for the bar *f*-mode instability, with an accuracy of 4–6%, for a wide range of realistic EOSs.

**Fig. 15:**
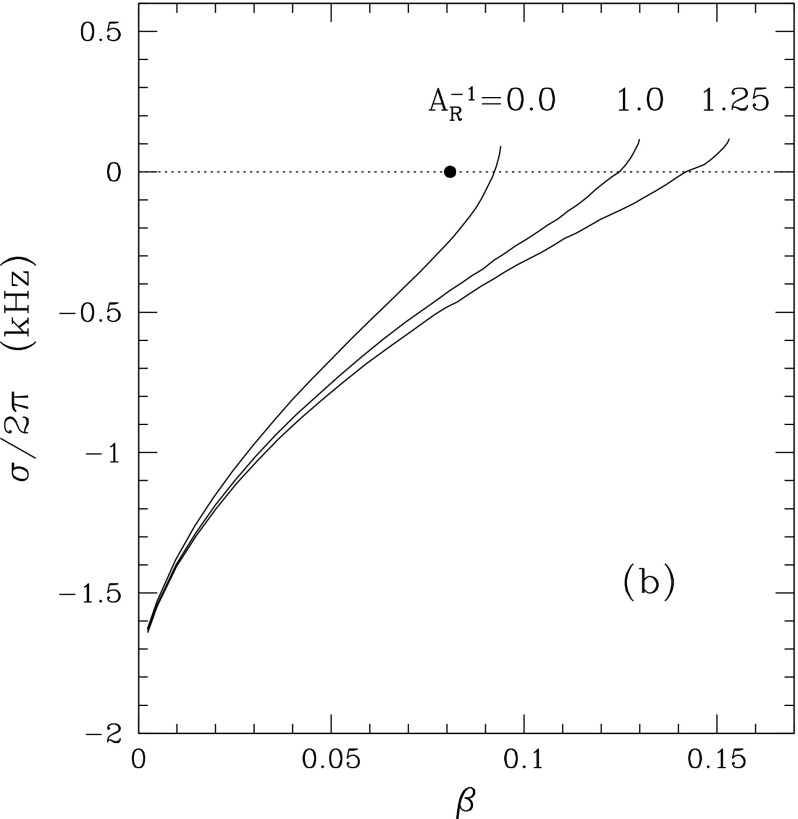
Eigenfrequencies (in the Cowling approximation) of the $m=2$ mode as a function of the parameter $\beta =T/|W|$ for three different sequences of differentially rotating neutron stars (the $A_{\mathrm {r}}^{-1}=0.0$ line corresponding to uniform rotation). The filled circle indicates the neutral stability point of a uniformly rotating star computed in full general relativity (Stergioulas and Friedman 1998). Differential rotation shifts the neutral point to higher rotation rates. (Image reproduced with permission from Yoshida et al. 2002, copyright by AAS)

In newly-born neutron stars the CFS instability could develop while the background equilibrium star is still differentially rotating. In that case, the critical value of *T* / *W*, required for the instability in the *f*-mode to set in, is larger than the corresponding value in the case of uniform rotation (Yoshida et al. 2002) (Fig. 15). The mass-shedding limit for differentially rotating stars also appears at considerably larger *T* / *W* than the mass-shedding limit for uniform rotation. Thus, Yoshida et al. (2002) suggest that differential rotation favours the instability, since the ratio $(T/W)_{\mathrm {critical}}/(T/W)_{\mathrm {shedding}}$ decreases with increasing degree of differential rotation.

**Fig. 16:**
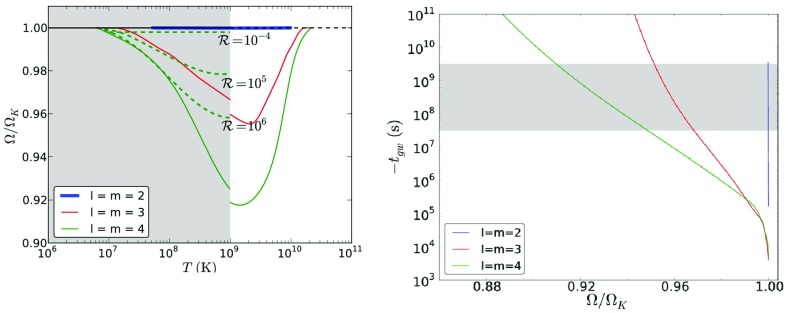
Left: The *f*-mode instability window neutron star rotation angular frequency vs temperature. Here $\varOmega _K\simeq 6868\,\mathrm {rad/s}$ is the angular frequency at the mass-shedding limit for a $n=0.73$, and mass $M=1.48\,M_{\odot }$ model with radius $R=10.47\,\mathrm {km}$ (in the TOV limit). The shaded area shows the region of superfluidity, and the dashed curves represent different values for the mutual friction drag parameter $\mathcal {R}$ (shown only for the $m = 4$ mode). Right: The *f*-mode instability growth time as a function of $\varOmega$ for the same model as in the left panel. The shaded area show the range of cooling timescales from an initial temperature $T = 5 \times 10^{10}\,\mathrm {K}$ to a final $T = 5{-}9 \times 10^8\,\mathrm {K}$. (Image reproduced with permission from Gaertig et al. 2011, copyright by APS)

Gaertig et al. (2011) perform a calculation of the CFS *f*-mode instability in rapidly rotating, relativistic neutron stars employing the Cowling approximation while treating dissipation through shear and bulk viscosity as well as superfluid mutual friction (Lindblom and Mendell 2000) of the nuclear matter. They adopt a polytropic equation of state and construct, self-consistent equilibria with the RNS code. The focus of the study is primarily on the $l=4,m=4$ mode, which is the dominant one because it has the largest instability window. For the full dissipative system, Gaertig et al. compute the *f*-mode instability growth time and instability window, i.e., the curve $\varOmega (T)$—the angular velocity at which the growth time of the instability equals the dissipation timescale due to viscous effects as a function of the temperature *T*. For the analysis they consider one background solution which is a star with polytropic index $n=0.73$, mass $M=1.48\,M_{\odot }$ and radius $R=10.47$ km (in the TOV limit). The main results are shown in Fig. 16 from which it becomes clear that the instability window for the $m=4$ mode is the widest with $\varOmega \gtrsim 0.92 \varOmega _K$, where $\varOmega _K$ is the mass-shedding limit angular velocity. The minimum *f*-mode instability growth time found is on the order of $10^{3}\,\mathrm {s}$. The authors conclude that the *f*-mode instability is more likely to be excited in nascent neutron stars, spinning with $\varOmega \gtrsim 0.9\varOmega _K$ and having a temperature $T \lesssim 2\times 10^{10}\,\mathrm {K}$.

Passamonti et al. (2013) adopt linear perturbation theory and the Cowling approximation to study the evolution of the *f*-mode instability in rapidly rotating, polytropic relativistic neutron stars, while treating thermal effects, magnetic fields, and the impact of unstable *r*-modes. The authors focus on two polytropic stars with indices $n=1$ and $n=0.62$, and masses $1.4\,M_{\odot }$ and $1.98\,M_{\odot }$, respectively. They report that magnetic fields affect the evolution and the gravitational waves generated during the instability, if the strength is larger than $10^{12}\,\mathrm {G}$. An unstable *r*-mode dominates over the *f*-mode when the *r*-mode reaches saturation but these conclusions are limited by the unknown *r*-mode amplitudes at saturation. Finally, the authors find that the thermal evolution suggests that heat generated by shear viscosity during the saturation phase balances exactly cooling by neutrinos, and prevents mutual friction from ever becoming important.

Doneva et al. (2013a) adopt the Cowling approximation to study the *f*-mode instability in rapidly rotating stars with realistic equations of state focusing on a constant mass sequence with $M=2.0\,M_{\odot }$. The authors confirm the earlier result that $l=m=4$ modes have a larger instability window (Gaertig et al. 2011). In addition, the authors report that realistic EOSs have a larger instability window than polytropic EOSs, thus, favouring the *f*-mode CFS instability.

#### CFS instability of axial modes

In nonrotating stars, axial fluid modes are degenerate at zero frequency, but in rotating stars they have nonzero frequency and are called *r*-modes in the Newtonian limit (Papaloizou and Pringle 1978; Saio 1982). To order $\mathcal{O}(\varOmega )$, their frequency in the inertial frame is

135 $$\begin{aligned} \omega _{\mathrm {i}} = -m\varOmega \Bigl ( 1-\frac{2}{l(l+1)} \Bigr ), \end{aligned}$$

while the radial eigenfunction of the perturbation in the velocity can be determined at order $\varOmega ^2$ (Kojima 1998). According to Eq. (135), *r*-modes with $m>0$ are prograde ($\omega _{\mathrm {i}}<0$) with respect to a distant observer but retrograde ($\omega _{\mathrm {r}} = \omega _{\mathrm {i}}+m\varOmega >0$) in the comoving frame for all values of the angular velocity. Thus, *r*-modes in relativistic stars are generically unstable to the emission of gravitational waves via the CFS instability, as was first discovered by Andersson (1998) for the case of slowly rotating, relativistic stars. This result was proved rigorously by Friedman and Morsink (1998), who showed that the canonical energy of the modes is negative.

Two independent computations in the Newtonian Cowling approximation (Lindblom et al. 1998; Andersson et al. 1999) showed that the usual shear and bulk viscosity assumed to exist for neutron star matter is not able to damp the *r*-mode instability, even in slowly rotating stars. In a temperature window of $10^5 \,\mathrm {K}< T < 10^{10} \,\mathrm {K}$, the growth time of the $l=m=2$ mode becomes shorter than the shear or bulk viscosity damping time at a critical rotation rate that is roughly one tenth the maximum allowed angular velocity of uniformly rotating stars. Gravitational radiation is dominated by the mass quadrupole term. These results suggested that a rapidly rotating proto-neutron star will spin down to Crab-like rotation rates within one year of its birth, because of the *r*-mode instability. Due to uncertainties in the actual viscous damping times and because of other dissipative mechanisms, this scenario is also consistent with somewhat higher initial spins, such as the suggested initial spin period of several milliseconds for the X-ray pulsar in the supernova remnant N157B (Marshall et al. 1998). Millisecond pulsars with periods less than a few milliseconds can then only form after the accretion-induced spin-up of old pulsars and not in the accretion-induced collapse of a white dwarf.

**Fig. 17:**
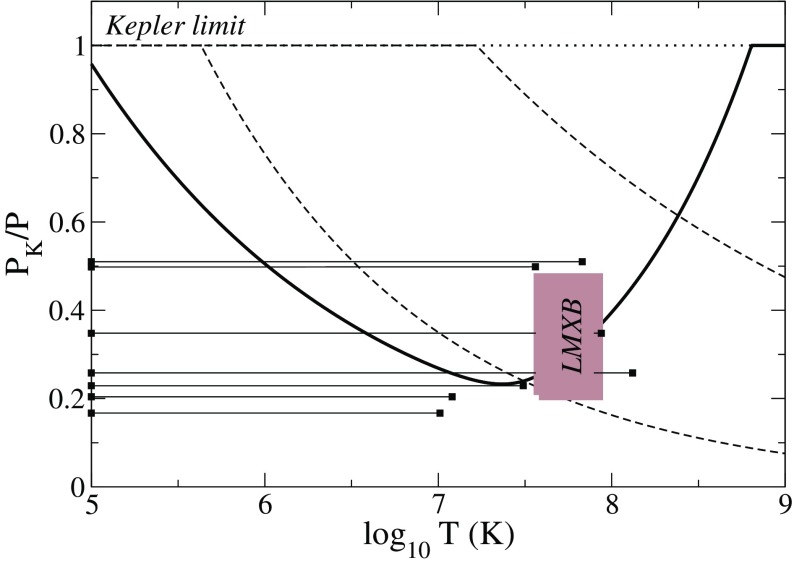
The *r*-mode instability window for a strange star of $M=1.4\,M_{\odot }$ and *R* = 10 km (solid line). Dashed curves show the corresponding instability windows for normal npe fluid and neutron stars with a crust. The instability window is compared to (i) the inferred spin-periods for accreting stars in LMBXs [shaded box], and (ii) the fastest known millisecond pulsars (for which observational upper limits on the temperature are available) [horizontal lines]. (Image reproduced with permission from Andersson et al. 2002, copyright by RAS)

The precise limit on the angular velocity of newly-born neutron stars will depend on several factors, such as the strength of the bulk viscosity, the cooling process, superfluidity, the presence of hyperons, and the influence of a solid crust. In the uniform density approximation, the *r*-mode instability can be studied analytically to $\mathcal{O}(\varOmega ^2)$ in the angular velocity of the star (Kokkotas and Stergioulas 1999). A study on the issue of detectability of gravitational waves from the *r*-mode instability was presented in Owen et al. (1998) (see Sect. 4.5.5), while Andersson et al. (1999) and Bildsten (1998) proposed that the *r*-mode instability is limiting the spin of millisecond pulsars spun-up in LMXBs and it could even set the minimum observed spin period of $\sim 1.5\,\mathrm {ms}$ (see Andersson et al. 2000). This scenario is also compatible with observational data, if one considers strange stars instead of neutron stars (Andersson et al. 2002) (see Fig. 17).

**Fig. 18:**
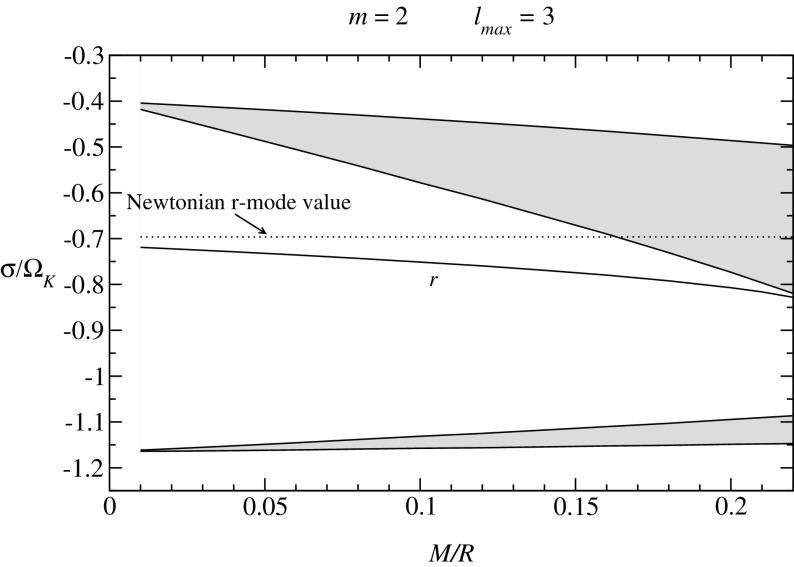
Relativistic *r*-mode frequencies for a range of the compactness ratio *M*/*R*. The coupling of polar and axial terms, even in the order $\mathcal{O}(\varOmega )$ slow rotation approximation has a dramatic impact on the continuous frequency bands (shaded areas), allowing the *r*-mode to exist even in highly compact stars. The Newtonian value of the *r*-mode frequency is plotted as a dashed-dotted line. (Image reproduced with permission from Ruoff et al. 2003, copyright by RAS)

Since the discovery of the *r*-mode instability, a large number of authors have studied the development of the instability and its astrophysical consequences in more detail. Unlike in the case of the *f*-mode instability, many different aspects and interactions have been considered. This intense focus on the detailed physics has been very fruitful and we now have a much more complete understanding of the various physical processes that are associated with pulsations in rapidly rotating relativistic stars. The latest understanding of the *r*-mode instability is that it may not be a very promising gravitational wave source (as originally thought), but the important astrophysical consequences, such as the limits of the spin of young and of recycled neutron stars are still considered plausible. The most crucial factors affecting the instability are magnetic fields (Spruit 1999; Rezzolla et al. 2000, 2001a, b), possible hyperon bulk viscosity (Jones 2001; Lindblom and Owen 2002; Haensel et al. 2002) and nonlinear saturation (Stergioulas and Font 2001; Lindblom et al. 2001, 2002; Arras et al. 2003). The question of the possible existence of a continuous spectrum has also been discussed by several authors, but the most recent analysis suggests that higher order rotational effects still allow for discrete *r*-modes in relativistic stars (Yoshida and Lee 2002; Ruoff et al. 2003) (see Fig. 18).

Haskell and Andersson (2010) study the effects of superfluid hyperon bulk viscosity on the *r*-mode instability window using a multifluid formalism. They find that although the extra bulk viscosity does not alter the instability window qualitatively, it could become substantial and even suppress the *r*-mode instability altogether in a range of temperatures and neutron star radii. However, hyperons are predicted only by certain equations of state and the relativistic mean field theory is not universally accepted. Thus, our ignorance of the true equation of state still leaves a lot of room for the *r*-mode instability to be considered a viable mechanism for the generation of detectable gravitational radiation.

In a subsequent paper, Andersson et al. (2013) study the superfluid *r*-mode instability which arises in rotating stars in which there is “differential” rotation between the crust and the underlying superfluid, and which was first discovered by Glampedakis and Andersson (2009) as a new mechanism for explaining the unpinning of vortices in pulsar glitches. In Andersson et al. (2013), the analysis goes beyond the strong-drag limit adopted in Glampedakis and Andersson (2009) and it is shown that there exist dynamically unstable modes (growth time comparable to the stellar rotation period), and that the *r*-modes undergo a secular instability.

**Fig. 19:**
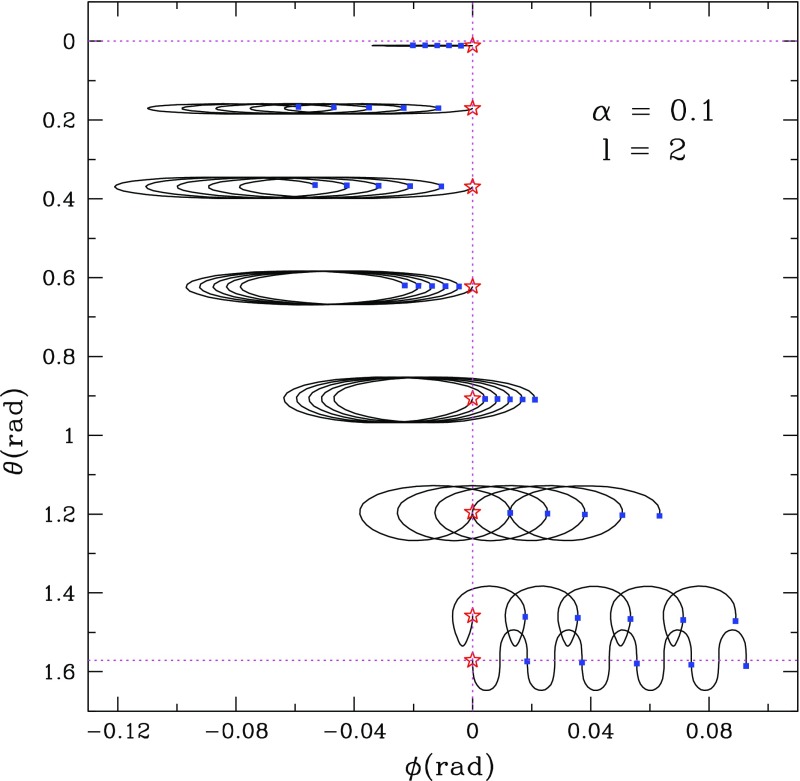
Projected trajectories of several fiducial fluid elements (as seen in the corotating frame) for an $l=m=2$ Newtonian *r*-mode. All of the fluid elements are initially positioned on the $\phi _0=0$ meridian at different latitudes (indicated with stars). Blue dots indicate the position of the fluid elements after each full oscillation period. The *r*-mode induces a kinematical, differential drift. (Image reproduced with permission from Rezzolla et al. 2001a, copyright by APS)

Magnetic fields can affect the *r*-mode instability, as the *r*-mode velocity field creates differential rotation, which is both kinematical and due to gravitational radiation reaction (see Fig. 19). Under differential rotation, an initially weak poloidal magnetic field is wound-up, creating a strong toroidal field, which causes the *r*-mode amplitude to saturate. On the other hand a more recent study of the effects of a dipole magnetic field on *r*-modes of slowly rotating, relativistic neutron stars by Chirenti and Skákala (2013) reports that magnetic fields affect the *r*-mode oscillation frequencies and the *r*-mode instability growth time very little even for strengths as large as $B \sim 10^{15}\,\mathrm {G}$.

**Fig. 20:**
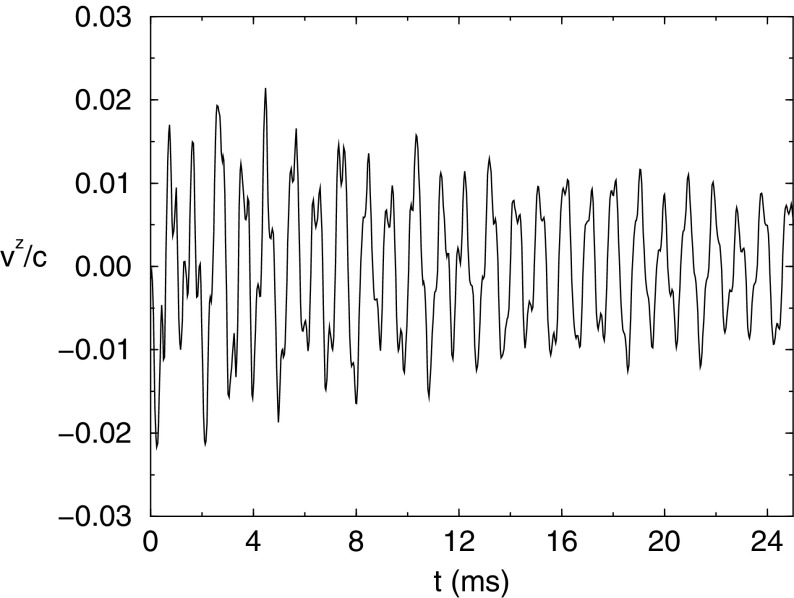
Evolution of the axial velocity in the equatorial plane for a relativistic *r*-mode in a rapidly rotating $N=1.0$ polytrope (in the Cowling approximation). Since the initial data used to excite the mode are not exact, the evolution is a superposition of (mainly) the $l=m=2$ *r*-mode and several inertial modes. The amplitude of the oscillation decreases due to numerical (finite-differencing) viscosity of the code. A beating between the $l=m=2$ *r*-mode and another inertial mode can also be seen. (Image reproduced with permission from Stergioulas and Font 2001, copyright by APS)

The detection of gravitational waves from *r*-modes depends crucially on the nonlinear saturation amplitude. A first study by Stergioulas and Font (2001) suggests that *r*-modes can exist at large amplitudes of order unity for dozens of rotational periods in rapidly rotating relativistic stars (Fig. 20). The study used 3D relativistic hydrodynamical evolutions in the Cowling approximation. This result was confirmed by Newtonian 3D simulations of nonlinear *r*-modes by Lindblom and Owen (2002), Lindblom et al. (2001). Lindblom et al. went further, using an accelerated radiation reaction force to artificially grow the *r*-mode amplitude on a hydrodynamical (instead of the secular) timescale. At the end of the simulations, the *r*-mode grew so large that large shock waves appeared on the surface of the star, while the amplitude of the mode subsequently collapsed. Lindblom et al. suggested that shock heating may be the mechanism that saturates the *r*-modes at a dimensionless amplitude of $\alpha \sim 3$.

Other studies of nonlinear couplings between the *r*-mode and higher order inertial modes (Arras et al. 2003) and new 3D nonlinear Newtonian simulations (Gressman et al. 2002) seem to suggest a different picture. The *r*-mode could be saturated due to mode couplings or due to a hydrodynamical instability at amplitudes much smaller than the amplitude at which shock waves appeared in the simulations by Lindblom et al. Such a low amplitude, on the other hand, modifies the properties of the *r*-mode instability as a gravitational wave source, but is not necessarily bad news for gravitational wave detection, as a lower spin-down rate also implies a higher event rate for the *r*-mode instability in LMXBs in our own Galaxy (Andersson et al. 2002; Heyl 2002). The 3D simulations need to achieve significantly higher resolutions before definite conclusions can be reached, while the Arras et al. work could be extended to rapidly rotating relativistic stars (in which case the mode frequencies and eigenfunctions could change significantly, compared to the slowly rotating Newtonian case, which could affect the nonlinear coupling coefficients). Spectral methods can be used for achieving high accuracy in mode calculations; first results have been obtained by Villain and Bonazzola (2002) for inertial modes of slowly rotating stars in the relativistic Cowling approximation.

More recently, Bondarescu et al. (2009) perform a study of the non-linear development of the *r*-mode instability, including three-mode couplings, neutrino cooling and viscous heating effects on rotating stars near the mass-shedding limit. In their most optimistic scenarios, the authors conclude that gravitational waves from the r-mode instability in young, rapidly spinning neutron stars may be detectable by advanced LIGO out to 1 Mpc for years, and perhaps decades, after formation. In follow up work, Bondarescu and Wasserman (2013) include interactions of the $\ell =m=2$ *r*-mode with “pairs of daugther modes” close to resonance. They find that if dissipation occurs at the crust–core boundary layer, the *r*-mode saturation amplitude is too large for the star to be spun up by accretion to even 300 Hz because of angular momentum loss to gravitational radiation. Spin up to higher frequencies seems to require that the core–crust transition occur over a lengthscale much longer than 1 cm.

The idea of utilizing X-ray and UV observations of low-mass X-ray binaries to constrain the physics of the *r*-mode instability is discussed by Haskell et al. (2012).

For a more extensive coverage of the numerous articles on the *r*-mode instability that appeared in recent years, the reader is referred to several review and recent articles (Andersson and Kokkotas 2001; Friedman and Lockitch 2002; Lindblom 2001; Kokkotas and Ruoff 2003; Andersson 2002; Alford et al. 2015; Kokkotas and Schwenzer 2016; Jasiulek and Chirenti 2016).

*Going further*:   If rotating stars with very high compactness exist, then *w*-modes can also become unstable, as was found by Kokkotas et al. (2004). The possible astrophysical implications are still under investigation.

#### Effect of viscosity on the CFS instability

In the previous sections, we have discussed the growth of the CFS instability driven by gravitational radiation in an otherwise non-dissipative star. The effect of neutron star matter viscosity on the dynamical evolution of nonaxisymmetric perturbations can be considered separately, when the timescale of the viscosity is much longer than the oscillation timescale. If $\tau _{\mathrm {gr}}$ is the computed growth rate of the instability in the absence of viscosity, and $\tau _{\mathrm {s}}$, $\tau _{\mathrm {b}}$ are the timescales of shear and bulk viscosity, then the total timescale of the perturbation is

136 $$\begin{aligned} \frac{1}{\tau } = \frac{1}{\tau _{\mathrm {gr}}} + \frac{1}{\tau _{\mathrm {s}}} + \frac{1}{\tau _{\mathrm {b}}}. \end{aligned}$$

Since $\tau _{\mathrm {gr}} < 0$ and $\tau _{\mathrm {b}}$, $\tau _{\mathrm {s}}>0$, a mode will grow only if $\tau _{\mathrm {gr}}$ is shorter than the viscous timescales, so that $1/\tau <0$.

In normal neutron star matter, shear viscosity is dominated by neutron–neutron scattering with a temperature dependence of $T^{-2}$ (Flowers and Itoh 1976), and computations in the Newtonian limit and post-Newtonian approximation show that the CFS instability is suppressed for $T <10^6$–$10^7 \,\mathrm {K}$ (Ipser and Lindblom 1991b, a; Yoshida and Eriguchi 1995; Lindblom 1995). If neutrons become a superfluid below a transition temperature $T_{\mathrm {s}}$, then mutual friction, which is caused by the scattering of electrons off the cores of neutron vortices could significantly suppress the *f*-mode instability for $T<T_{\mathrm {s}}$ (Lindblom and Mendell 1995), but the *r*-mode instability remains unaffected (Lindblom and Mendell 2000). The superfluid transition temperature depends on the theoretical model for superfluidity and lies in the range $10^8$–$6 \times 10^9 \,\mathrm {K}$ (Page 1994).

In a pulsating fluid that undergoes compression and expansion, the weak interaction requires a relatively long time to re-establish equilibrium. This creates a phase lag between density and pressure perturbations, which results in a large bulk viscosity (Sawyer 1989). The bulk viscosity due to this effect can suppress the CFS instability only for temperatures for which matter has become transparent to neutrinos (Lai and Shapiro 1995; Bonazzola et al. 1996). It has been proposed that for $T>5 \times 10^9 \,\mathrm {K}$, matter will be opaque to neutrinos and the neutrino phase space could be blocked (Lai and Shapiro 1995; see also Bonazzola et al. 1996). In this case, bulk viscosity will be too weak to suppress the instability, but a more detailed study is needed.

In the neutrino transparent regime, the effect of bulk viscosity on the instability depends crucially on the proton fraction $x_{\mathrm {p}}$. If $x_{\mathrm {p}}$ is lower than a critical value ($\sim 1/9$), only modified URCA processes are allowed. In this case bulk viscosity limits, but does not completely suppress, the instability (Ipser and Lindblom 1991b, a; Yoshida and Eriguchi 1995). For most modern EOSs, however, the proton fraction is larger than $\sim 1/9$ at sufficiently high densities (Lattimer et al. 1991), allowing direct URCA processes to take place. In this case, depending on the EOS and the central density of the star, the bulk viscosity could almost completely suppress the CFS instability in the neutrino transparent regime (Zdunik 1996). At high temperatures, $T>5 \times 10^9 \,\mathrm {K}$, even if the star is opaque to neutrinos, the direct URCA cooling timescale to $T \sim 5 \times 10^9 \,\mathrm {K}$ could be shorter than the growth timescale of the CFS instability.

Bildsten and Ushomirsky (2000) considered the dissipation of *r*-modes due to the presence of a viscous boundary layer between the oscillating fluid and the crust and found it to be several orders of magnitude higher than the dissipation due to shear in the neutron star interior, if the crust is rigid. Subsequently, Lindblom et al. (2000) included the effects of the Coriolis force in more realistic neutron-star models, finding that an *r*-mode amplitude value of $\sim 5\times 10^{-3}$ for maximally rotating stars would result in sufficient heating at the crust-core boundary layer for the crust to melt. These initial computations used a rigid crust that did not participate in the *r*-mode oscillation, but the magnitude of the effect strongly depends on a slippage parameter $\mathcal S$ that measures the fractional difference in velocity of the normal fluid between the crust and the core and the fractional pinning of vortices in the crust (Levin and Ushomirsky 2001; Glampedakis and Andersson 2006a, b). The dependence of the crust-core slippage on the spin frequency is complicated, and is very sensitive to the physical thickness of the crust.

*Going further*:   For more recent work on viscous damping of *r*-modes see Alford et al. (2012) (and references therein) who treat non-linear viscous effects in the large-amplitude regime, as well as consider hadronic stars, strange quark stars, and hybrid stars. See also Kolomeitsev and Voskresensky (2015) (and references therein) for a microphysical computation of the shear and bulk viscosities from various processes and applications to viscous damping of *r*-modes. For recent work on the *r*-mode instability window and applications on pulsar recycling see Gusakov et al. (2014, 2016), and references therein.

#### Gravitational radiation from CFS instability

Conservation of angular momentum and the inferred initial period (assuming magnetic braking) of a few milliseconds for the X-ray pulsar in the supernova remnant N157B (Marshall et al. 1998) suggests that a fraction of neutron stars may be born with very large rotational energies. The *f*-mode bar CFS instability thus appears as a promising source for the planned gravitational wave detectors (Lai and Shapiro 1995). It could also play a role in the rotational evolution of merged binary neutron stars, if the post-merger angular momentum exceeds the maximum allowed to form a Kerr black hole (Baumgarte and Shapiro 1998b) or if differential rotation temporarily stabilizes the merged object.

Lai and Shapiro (1995) have studied the development of the *f*-mode instability using Newtonian ellipsoidal models (Lai et al. 1993, 1994). They consider the case when a rapidly rotating neutron star is created in a core collapse. After a brief dynamical phase, the proto-neutron star becomes secularly unstable. The instability deforms the star into a nonaxisymmetric configuration via the $l=2$ bar mode. Since the star loses angular momentum via the emission of gravitational waves, it spins down until it becomes secularly stable. The frequency of the waves sweeps downward from a few hundred Hz to zero, passing through LIGO’s ideal sensitivity band. A rough estimate of the wave amplitude shows that, at $\sim 100\,\mathrm {Hz}$, the gravitational waves from the CFS instability could be detected out to the distance of 140 Mpc by the advanced LIGO detector. This result is very promising, especially since for relativistic stars the instability will be stronger than the Newtonian estimate (Stergioulas and Friedman 1998). More recent work by Passamonti et al. (2013) suggests that the gravitational wave signal generated during the *f*-mode instability, could potentially be detectable by Advanced LIGO/Virgo from a source located in the Virgo cluster, as long as the star was massive enough.

Pnigouras and Kokkotas (2015, 2016) develop a formalism to study the saturation of the *f*-mode instability as a result of nonlinear coupling of modes. They find that parent (unstable) modes couple resonantly to daughter modes which drain energy from the parent modes leading to saturation of the instability. These results can be applicable to neutron stars formed in core collapse and following neutron star mergers. Doneva et al. (2015a) report that gravitational waves generated by the *f*-mode instability in supramassive neutron stars (that could form following binary neutron star mergers) could be detectable by advanced LIGO at 20 Mpc (where, however, the event rate is very low, so that a more sensitive instrument is needed for realistic detection rates). The stochastic gravitational wave background due to the *f*-mode instability in neutron stars is estimated by in Surace et al. (2016). They find that for the $l=m=2$ *f*-mode $\varOmega _\mathrm{GW} \sim 10^{-9}$ which could be detectable through cross correlating data from pairs of grounds based detectors.

**Fig. 21:**
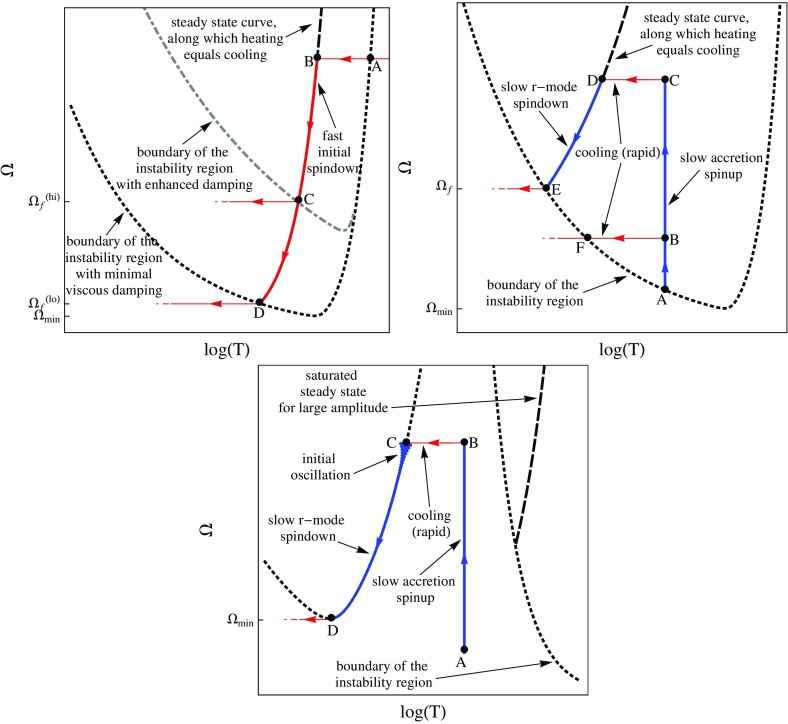
Different scenarios in which the r-mode instability can generate gravitational waves in the angular velocity ($\varOmega$) - temperature (*T*) plane. Top left panel: Spindown of nascent sources; Top right panel: Pulsar recycling in LMXBs and spindown of millisecond pulsars; Lower panel: Recycling and spindown in sources with increased damping. Each time the evolution goes through or lies at the boundary of the instability region (region within the dotted lines) gravitational wave emission is switched on. (Image reproduced with permission from Kokkotas and Schwenzer 2016, copyright by SIF/Springer)

Whether *r*-modes should also be considered a promising gravitational wave source depends crucially on their nonlinear saturation amplitude (see Sect. 4.5.3). Nevertheless, the issues of detectability and interpretation of gravitational waves generated by the *r*-mode instability is discussed by Owen (2010), and the effects of realistic equations of state and the potential for gravitational waves from the *r*-mode instability to constrain the nuclear equation of state are studied by Idrisy et al. (2015). Applications of the different *r*-mode instability scenarios (see Fig. 21) in gravitational-wave astronomy were recently presented by Kokkotas and Schwenzer (2016).

*Going further*:   The possible ways for neutron stars to emit gravitational waves and their detectability are reviewed in Bonazzola and Gourgoulhon (1996, 1997), Giazotto et al. (1997), Flanagan (1998), Thorne (1995), Schutz (1999) and Cutler and Thorne (2002).

#### Viscosity-driven instability

A different type of nonaxisymmetric instability in rotating stars is the instability driven by viscosity, which breaks the circulation of the fluid (Roberts and Stewartson 1963; James 1964). The instability is suppressed by gravitational radiation, so it cannot act in the temperature window in which the CFS instability is active. The instability sets in when the frequency of an $l=-m$ mode goes through zero in the rotating frame. In contrast to the CFS instability, the viscosity-driven instability is not generic in rotating stars. The $m=2$ mode becomes unstable at a high rotation rate for very stiff stars, and higher *m*-modes become unstable at larger rotation rates.

In Newtonian polytropes, the instability occurs only for stiff polytropes of index $N<0.808$ (James 1964; Skinner and Lindblom 1996). For relativistic models, the situation for the instability becomes worse, since relativistic effects tend to suppress the viscosity-driven instability (while the CFS instability becomes stronger). According to recent results by Bonazzola et al. (1998), for the most relativistic stars, the viscosity-driven bar mode can become unstable only if $N<0.55$. For $1.4\,M_{\odot }$ stars, the instability is present for $N<0.67$.

These results are based on an approximate computation of the instability in which one perturbs an axisymmetric and stationary configuration, and studies its evolution by constructing a series of triaxial quasi-equilibrium configurations. During the evolution only the dominant nonaxisymmetric terms are taken into account. The method presented in Bonazzola et al. (1998) is an improvement (taking into account nonaxisymmetric terms of higher order) of an earlier method by the same authors (Bonazzola et al. 1996). Although the method is approximate, its results indicate that the viscosity-driven instability is likely to be absent in most relativistic stars, unless the EOS turns out to be unexpectedly stiff.

An investigation by Shapiro and Zane (1996) of the viscosity-driven bar mode instability, using incompressible, uniformly rotating triaxial ellipsoids in the post-Newtonian approximation, finds that the relativistic effects increase the critical *T* / *W* ratio for the onset of the instability significantly. More recently, new post-Newtonian (Di Girolamo and Vietri 2002) and fully relativistic calculations for uniform density stars (Gondek-Rosińska and Gourgoulhon 2002) show that the viscosity-driven instability is not as strongly suppressed by relativistic effects as suggested in Shapiro and Zane (1996). The most promising case for the onset of the viscosity-driven instability (in terms of the critical rotation rate) would be rapidly rotating strange stars (Gondek-Rosińska et al. 2003), but the instability can only appear if its growth rate is larger than the damping rate due to the emission of gravitational radiation—a corresponding detailed comparison is still missing.

The non-linear evolution of the bar mode instability has been studied via post-Newtonian hydrodynamic simulations by Ou et al. (2004), and Shibata and Karino (2004). Ou et al. find that the instability goes through a “Dedekind-like” configuration (Chandrasekhar 1969) before becoming unstable due to a hydrodynamical shearing instability. Shibata and Karino find that the end state of the instability is an ellipsoidal star of ellipticity $e\gtrsim 0.7$.

#### One-arm (spiral) instability

A remarkable feature about highly differentially rotating neutron stars is that they can also become unstable to a dynamical one-arm ($m=1$) “spiral” instability.

The one-arm instability in differentially rotating stars was discovered in Newtonian hydrodynamic simulations with soft polytropic equations of state and a high degree of differential rotation by Centrella et al. (2001). The instability growth occurs on a dynamical (rotational period) timescale and saturates within a few tens of rotational periods. During the development of the instability a perturbation displaces the stellar core from the center of mass resulting in the core orbiting around the center of mass at roughly constant angular frequency. The $m=1$ deformation leads to a time-changing quadrupole moment which results in the emission of gravitational waves which may be detectable. This, in part, motivates the study of this instability and the conditions under which it develops.

Shortly after the discovery of the $m=1$ instability, Saijo et al. (2003) confirmed its existence with further Newtonian hydrodynamic simulations and suggested that a toroidal configuration may be necessary to trigger the one-arm instability but not sufficient. Guided by observations reported by Watts et al. (2005) that the low-*T* / |*W*| dynamical (bar-mode) instability (discovered by Shibata et al. 2002, 2003 for highly differentially rotating stars, see also Saijo and Yoshida 2006; Cerdá-Durán et al. 2007; Passamonti and Andersson 2015) develops near the corotation radius, i.e., the radius where the angular frequency of the unstable mode matches the local angular velocity of the fluid, Saijo and Yoshida (2006) argue that the one-arm spiral instability is also excited near the corotation radius. Hydrodynamic simulations of differentially rotating stars by Ou and Tohline (2006) in Newtonian gravity, and by Corvino et al. (2010) in general relativity seem to confirm this picture, although Ou and Tohline also point to the significance of the existence of a minimum of the vortensity within the star. These studies seem to point to a type of resonant excitation of the unstable mode. Ou and Tohline (2006) further find that the one-arm spiral instability can develop even for stiff equations of state ($\varGamma =2$), as well as for non-toroidal configurations, as long as the radial vortensity profile exhibits a local minimum. A recent simplified Newtonian perturbative analysis by Saijo and Yoshida (2016) (see also Yoshida and Saijo 2017) solving an eigenvalue problem on the equatorial plane of a star with $j=\mathrm const.$ differential rotation law, suggests that when a corotation radius is present *f*-modes become unstable giving rise to the class of “low-*T* / |*W*|”, shearing instabilities. In Yoshida and Saijo (2017), the role of the corrotation radius is further explored and the authors suggest that low-*T* / |*W*| instabilities may arise due because of “over-reflection” of sound waves between the stellar surface and the corotation band. More recently, Muhlberger et al. (2014), find that $m=1$ modes were excited in general-relativistic magnetohydrodynamic simulations of the low-*T* / |*W*| instability in isolated neutron stars (see also Fu and Lai 2011). Despite multiple studies of the $m=1$ instability, a clear interpretation of how and under what conditions the instability arises is still absent.

## Dynamical simulations of rotating stars in numerical relativity

In the framework of the $3+1$ split of the Einstein equations (Smarr and York 1978), the spacetime metric obtains the Arnowitt–Deser–Misner (ADM) form (Arnowitt et al. 2008)

137 $$\begin{aligned} ds^2=-(\alpha ^2-\beta _i\beta ^i) \, dt^2 + 2\beta _i \, dx^i \, dt+\gamma _{ij} \, dx^i \, dx^j, \end{aligned}$$

where $\alpha$ is the lapse function, $\beta ^i$ is the shift three-vector, and $\gamma _{ij}$ is the spatial three-metric, with $i=1\ldots 3$. Casting the spacetime metric of a stationary, axisymmetric rotating star [see Eq. (5)] in the ADM form, the metric has the following properties:

- The metric function $\omega$ describing the dragging of inertial frames by rotation is related to the shift vector through $\beta ^\phi =-\omega$. This shift vector satisfies the *minimal distortion shift* condition.
- The metric satisfies the *maximal slicing* condition, while the lapse function is related to the metric function $\nu$ in (5) through $\alpha =e^\nu$.
- The quasi-isotropic coordinates are suitable for numerical evolution, while the radial-gauge coordinates (Bardeen and Piran 1983) are not suitable for nonspherical sources (see Bonazzola et al. 1993 for details).
- The ZAMOs are the Eulerian observers, whose worldlines are normal to the $t = \,\mathrm {const.}$ hypersurfaces.
- Uniformly rotating stars have $\varOmega = \,\mathrm {const.}$ in the *coordinate frame*. This can be shown by requiring a vanishing rate of shear.
- Normal modes of pulsation are discrete in the coordinate frame and their frequencies can be obtained by Fourier transforms (with respect to coordinate time *t*) of evolved variables at a fixed coordinate location (Font et al. 2000).

Crucial ingredients for the successful long-term and accurate evolution of rotating stars in numerical relativity are the Baumgarte–Shapiro–Shibata–Nakamura (BSSN) (see Nakamura et al. 1987; Shibata and Nakamura 1995; Baumgarte and Shapiro 1998a; Alcubierre et al. 2000) or Generalized-Harmonic (Pretorius 2005; Lindblom et al. 2006) formulations for the spacetime evolution, and high-order, finite volume (magneto)hydrodynamical schemes that have been shown to preserve the sharp features at the surface of the star (see, e.g., Font et al. 2000; Stergioulas and Font 2001; Font et al. 2002; Shibata 2003a; Baiotti et al. 2005a; Duez et al. 2005; Anderson et al. 2006; Giacomazzo and Rezzolla 2007; Yamamoto et al. 2008; East et al. 2012; Löffler et al. 2012).

**Fig. 22:**
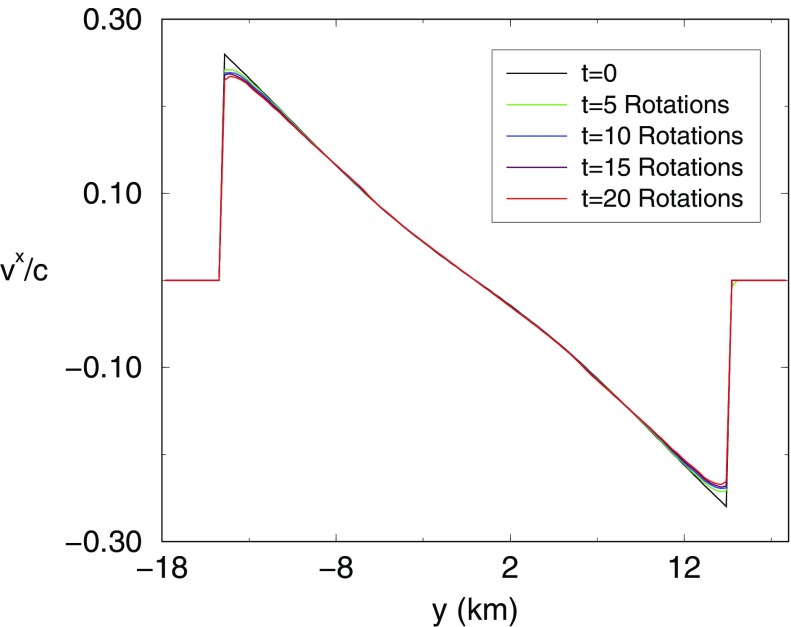
Time evolution of the rotational velocity profile for a stationary, rapidly rotating relativistic star (in the Cowling approximation), using the 3rd order PPM scheme and a $116^3$ grid. The initial rotational profile is preserved to a high degree of accuracy, even after 20 rotational periods. (Image reproduced with permission from Stergioulas and Font 2001, copyright by APS)

### Numerical evolution of equilibrium models

#### Stable equilibrium

Preserving the equilibrium of a stable rotating neutron star has now become a standard test for numerical relativity codes. The long-term stable evolution of rotating relativistic stars in 3D simulations has become possible through the use of High-Resolution Shock-Capturing (HRSC) methods (see Font 2008 for a review). Stergioulas and Font (2001) evolve rotating relativistic stars near the mass-shedding limit for dozens of rotational periods (evolving only the equations of hydrodynamics) (see Fig. 22), while accurately preserving the rotational profile, using the 3rd order PPM reconstruction (Colella and Woodward 1984). This method was shown to be superior to other, commonly used methods, in 2D evolutions of rotating relativistic stars (Font et al. 2000).

Fully coupled hydrodynamical and spacetime evolutions in 3D have been obtained by Shibata (1999a) and by Font et al. (2002). In Shibata (1999a), the evolution of approximate (conformally flat) initial data is presented for about two rotational periods, and in Font et al. (2002), the simulations extend to several full rotational periods, using numerically exact initial data and a monotonized central difference (MC) slope limiter (van Leer 1977). The MC slope limiter is somewhat less accurate in preserving the rotational profile of equilibrium stars than the 3rd order PPM method, but, on the other hand, it is easier to implement in a numerical code.

Other evolutions of uniformly and differentially rotating stars in 3D, using different gauges and coordinate systems, are presented in Duez et al. (2003), while 2D evolutions are presented in Shibata (2003a). In Duez et al. (2005), the axisymmetric dynamical evolution of a rapidly, uniformly rotating neutron star for 10 rotation periods shows that the PPM reconstruction preservers the maximum value of the rest-mass density better than either the MC or the Convex Essentially Non-oscillatory (CENO) (Liu and Osher 1998) reconstruction method. It is reported that PPM achieves similar performance also for full 3D evolution of the same rotating neutron star models. The initial data for these simulations are equilibrium numerical solutions of the Einstein equations generated using the Cook et al. (1996) code. Evolutions of stable, uniformly rotating neutron stars are also performed in Liebling et al. (2010) with initial data generated in Bocquet et al. (1995).

#### Instability to collapse

*Hydrodynamic Simulations:* Shibata et al. (2000b) study the stability of supramassive neutron stars rotating at the mass-shedding limit, for a $\varGamma =2$ polytropic EOS. Their 3D simulations in full general relativity show that stars on the mass-shedding sequence, with central energy density somewhat larger than that of the maximum mass model, are dynamically unstable to collapse. Thus, the dynamical instability of rotating neutron stars to axisymmetric perturbations is close to the corresponding secular instability. The initial data for these simulations are approximate, conformally flat axisymmetric solutions, but their properties are not very different from exact axisymmetric solutions even near the mass-shedding limit (Cook et al. 1996). It should be noted that the approximate minimal distortion (AMD) shift condition does not prove useful in the numerical evolution, once a horizon forms. Instead, modified shift conditions are used in Shibata et al. (2000b). In the above simulations, no massive disk around the black hole is formed, because the equatorial radius of the initial model is inside the radius which becomes the ISCO of the final black hole, a result also confirmed in Baiotti et al. (2005a) via 3D hydrodynamic evolution in full general relativity, using HRSC methods and excision technique to follow the evolution past the black hole formation.

To study the effects of the stiffness of the equation of state, Shibata (2003b) performs axisymmetric hydrodynamic simulations in full GR of polytropic supramassive neutron stars with polytropic index between 2 / 3 and 2. The initial data are marginally stable and to induce gravitational collapse, Shibata initially reduced the pressure uniformly by 0.5% subsequently solving the Hamiltonian and momentum constraints. Independently of the polytropic index he finds the final state to be a Kerr black hole, and the disk mass to be $<10^{-3}$ of the initial stellar mass.

Adopting the BSSN formulation, Duez et al. (2004) perform evolutions of rapidly (differentially) rotating, hypermassive, $n=1$ polytropic neutron stars with strong shear viscosity in full general relativity both in axisymmetry and in 3D, as a means for predicting the outcome after the loss of differential rotation. Like magnetic fields, shear viscosity redistributes angular momentum, braking the differential rotation until the star is eventually uniformly rotating. During this process the outer layers of the star gain angular momentum and the star expands. The loss of the differential rotation support may lead to collapse to a black hole. The initial data used are self-consistent general relativistic equilibria generated with the Cook et al. (1994b, 1994a) code. It is found that without rapid cooling, if the hypermassive NS is sufficiently massive (38% more massive than the $n=1$ supramassive-limit mass) the star collapses and forms a black hole after about 28 rotational periods surrounded by a massive disk. The evolution is continued through black hole formation by using excision methods. Hypermassive neutron stars whose mass is only 10% larger than the supramassive limit, do not promptly collapse to a black hole due to the additional support provided by thermal pressure which is generated through viscous heating. However, rapid cooling (e.g., due to neutrinos) can remove the excess heat and eventually these hypermassive neutron star models collapse to a black hole, too. In all cases where a black hole forms, a massive disk surrounds the black hole with rest-mass 10–20% of the initial stellar rest mass.

A different aspect of the neutron star collapse to a black hole is investigated in Giacomazzo et al. (2011), where the focus is on cosmic censorship. They performed hydrodynamic simulations in full general relativity of differentially rotating, polytropic neutron star models generated by the RNS code (Stergioulas 1999). The evolutions are performed using the BSSN formulation, and the Whisky MHD code (Giacomazzo and Rezzolla 2007). They consider 5 values for the polytropic index (0.5, 0.75, 1.0, 1.25, 1.5), and initial models that are both sub-Kerr $J/M^2<1$ and supra-Kerr $J/M^2>1$ to answer the following two questions: (1) Do dynamically unstable stellar models exist with $J/M^2 > 1$? (2) If a stable stellar model with $J/M^2 > 1$ is artificially induced to collapse to a black hole, does it violate cosmic censorship? The answer to question (1) is that finding supra-Kerr models which are dynamically unstable to gravitational collapse will be difficult as at least a parameter survey for different polytropes and different strengths of (one-parameter) differential rotation did not produce such models. The answer to (2) is that a supra-Kerr model can be induced to collapse only if a severe pressure depletion is performed. However, even in this case, prompt formation of a rotating black hole does not take place. This result does not exclude the possibility that a naked singularity can be produced by the collapse of a supra-Kerr, differentially rotating star. However, the authors argue that a generic supra-Kerr progenitor does not form a naked singularity, thereby indicating that cosmic censorship still holds in the collapse of differentially rotating neutron stars.

**Fig. 23:**
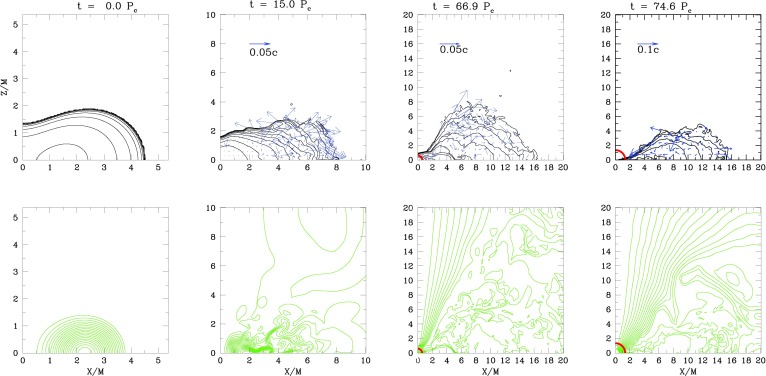
Magnetohydrodynamic evolution in full GR of a hypermassive, $n=1$ polytropic neutron star which is initially $70\%$ more massive than the $n=1$ supramassive-limit mass. Upper row: rest-mass density contours corresponding to $\rho /\rho _\mathrm{max,0}= 10^{-0.3 i-0.09} (i=0{-}12)$ and velocity arrows. Lower row: magnetic field lines are drawn for $A_{\phi } = A_{\phi ,\min } + (A_{\phi ,\max } - A_{\phi ,\min }) i/20 (i=1{-}19)$, where $A_{\phi ,\max }$ and $A_{\phi ,\mathrm min}$ are the maximum and minimum value of $A_{\phi }$ (the toroidal component of the vector potential) respectively at the given time. The thick solid (red) curves denote the black hole apparent horizon. (Image reproduced with permission from Duez et al. (2006a), copyright by APS)

*Magnetohydrodynamic Simulations:* Duez et al. (2006a) perform axisymmetric ideal magnetohydrodynamic (MHD) evolutions in full GR of a $n=1$ polytropic, equilibrium model of hypermassive neutron star which is initially seeded with dynamically unimportant purely poloidal magnetic fields (plasma $\beta$ parameter $\sim 10^3$), but sufficiently strong so that the fastest growing mode of the MRI can be resolved. The mass of the neutron star model is 70% larger than the corresponding TOV limit. The spacetime evolution is performed using the BSSN formulation and a non-staggered, flux-CT constrained transport method is employed to enforce the $\mathbf{\nabla \cdot B}=0$ constraint to machine precision (see Duez et al. 2005 and references therein). They find that magnetic winding and the MRI amplify the magnetic field and magnetically brake the differential rotation through redistribution of angular momentum. Following 74.6 rotational periods of evolution, the star eventually collapses to form a black hole surrounded by a turbulent, magnetized, hot accretion torus with large scale collimated magnetic fields (see Fig. 23). Due to the chosen gauge conditions the evolution could not be continued sufficiently long after the black hole formation to observe collimated outflows, but the remnant system provides a promising engine for a short-hard Gamma-ray Burst (sGRB). In a companion paper, Shibata et al. (2006) perform axisymmetric magnetohydrodynamic simulations in full GR of the magnetorotational, catastrophic collapse of initially piecewise polytropic hypermassive neutron star models seeded with weak, purely poloidal magnetic fields, and find that the remnant torus has a temperature $\ge 10^{12}\,\mathrm {K}$ and can hence lead to copious ($\nu \bar{\nu }$) thermal radiation. In follow up work (Duez et al. 2006b), the authors use both polytropic and piecewise polytropic differentially rotating neutron star models and find that catastrophic collapse to a BH and a plausible sGRB engine forms only for sufficiently massive hypermassive neutron stars (as low as 14% more massive than the supramassive limit mass). The end state of initially differentially rotating neutron stars whose mass is smaller than the supramassive limit mass is a uniformly rotating neutron star core, surrounded by a differentially rotating torus-like envelope. The remnant black hole-tori systems are evolved in Stephens et al. (2008) using a combination of black hole excision and the Cowling approximation, finding that these systems launch mildly relativistic outflows (Lorentz factors $\sim 1.2$-1.5), but that a stiff equation of state is likely to suppress these outflows. We note that GW searches triggered by sGRBs have tremendous potential to constrain sGRB progenitors such as collapsing hypermassive neutron stars that are formed following binary neutron star mergers (Abbott et al. 2016c).

Magnetohydrodynamic evolutions of a magnetized and uniformly rotating, unstable neutron star in full general relativity and 3 spatial dimensions are performed in Liebling et al. (2010). Instead of the BSSN formulation, the generalized harmonic formulation with excision is adopted. The $\mathbf{\nabla \cdot B}=0$ constraint is controlled by means of a hyperbolic divergence cleaning method (see Anderson et al. 2006 and references therein). A $\varGamma$-law ($\varGamma =2$) equation of state is used and the initial neutron star models are self-consistent, $n=1$ polytropic, uniformly rotating, magnetized (polar magnetic field strength $10^{16}\,\mathrm {G}$), equilibrium, self-consistent solutions of the Einstein equations generated with the Magstar code (Bocquet et al. 1995). Without any initial perturbation the unstable star collapses and forms a black hole. In agreement with earlier studies, the calculations demonstrate no evidence of a significant remnant disk. However, evidence for critical phenomena is found when the initial star is perturbed: a slight increase of the initial pressure leads to collapse and black hole formation, however if the initial perturbation increases the pressure above a threshold value, the star expands and oscillates around a new, potentially stable solution.

*Gravitational radiation from rotating neutron star collapse:* Baiotti et al. (2005b) study gravitational wave emission from rotating collapse of a neutron star. They perform 3D hydrodynamic simulations in full general relativity using the Whisky hydrodynamics code (Baiotti et al. 2005a). Singularity excision is used to follow the black hole formation. The equilibrium initial data correspond to a uniformly rotating neutron star near its mass-shedding limit (of dimensionless spin parameter $J/M^2=0.54$), and are solutions to the Einstein equations. The matter is modeled as an $n=1$ polytrope, and the collapse is triggered by a $2\%$ pressure depletion. The constraints are re-solved after the pressure reduction to start the evolution with valid, constraint-satisfying initial data. A $\varGamma$-law equation of state is used for the evolution to allow for shock heating. The gravitational waves are extracted on spheres of large radius using the gauge invariant Moncrief method (Moncrief 1974). The authors find that a characteristic amplitude of the gravitational wave burst is $h_c = 5.77\times 10^{-22}(M/M_\odot )(r/10\,\mathrm {kpc})^{-1}$ at a characteristic frequency $f_c = 931\,\mathrm {Hz}$. The total energy emitted in gravitational waves is found to be $E/M = 1.45\times 10^{-6}$, and they report that these waves could be detectable by ground based gravitational wave laser interferometers, but only for nearby sources. In a follow-up paper, Baiotti and Rezzolla (2006) use the Whisky hydrodynamics code to perform simulations of collapsing, slowly and rapidly, uniformly rotating neutron stars. The rapidly rotating model is the same as the one in Baiotti et al. (2005b), while the slowly rotating model has a dimensionless spin parameter ($J/M^2=0.21$). For these simulations they adopted the puncture gauge conditions (Campanelli et al. 2006; Baker et al. 2006), instead of singularity excision, allowing them to continue the integration of the Einstein equations for much longer times and even study the black hole ring-down phase. With the complete gravitational wave train the authors report that the energy lost to gravitational wave emission becomes $E/M = 3.7\times 10^{-6}$ ($E/M = 3.3\times 10^{-6}M$) for the rapidly (slowly) rotating progenitor. The role of the puncture gauge conditions in collapse simulations has been investigated in Thierfelder et al. (2011), Dietrich and Bernuzzi (2015).

**Fig. 24:**
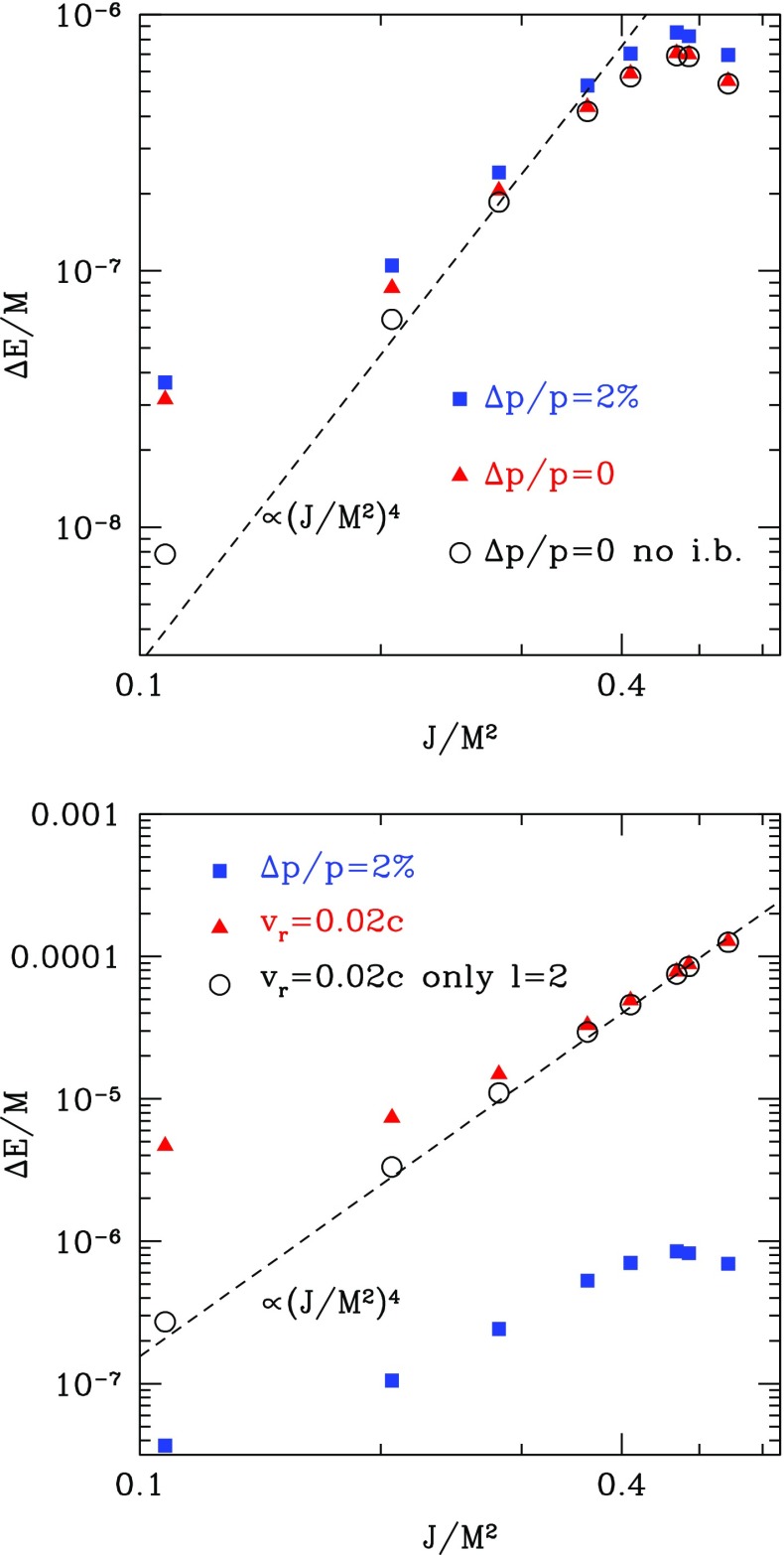
Energy carried off by gravitational waves during the collapse of uniformly rotating neutron stars for different values of dimensionless spin parameter $J/M^2$ and initial perturbations. Left panel: Filled squares and triangles denote models with a 2% pressure depletion and unperturbed models, respectively. Open triangles, refer to the same models as the filled ones, but exclude the initial (potentially spurious) burst in the waveforms. Right panel: Filled triangles denote models with an inward radial velocity perturbation of magnitude 0.02*c*, and the open circles the same models but considering only the $l =2$ contribution to the emitted energy; for comparison the pressure-depleted models are plotted (filled squares). In both plots the dashed lines indicate a scaling $\sim (J/M^2)^4$. (Image reproduced with permission from Baiotti et al. 2007, copyright by IOP)

To study the effects of rotation and different perturbations on the gravitational waves arising from the collapse of uniformly rotating neutron stars, Baiotti et al. (2007) perform hydrodynamic simulations in full GR using the Whisky code and similar evolution techniques as in Baiotti and Rezzolla (2006). For initial data they consider 9 equilibrium neutron star models along a sequence of dynamically unstable stars, all modeled as $n=1$ polytropes, whose dimensionless spin parameter ranges from 0 to 0.54, and their ratio of kinetic to gravitational binding energy ranges from 0 to 7.67. For all cases the collapse is induced either by a 2% pressure depletion or the addition of an inward, radial velocity perturbation of magnitude 0.02*c*, but this time the constraints are not resolved leading to a small initial violation of the constraints. The gravitational waves were extracted on a sphere of radius $50\,M$, and the results of their study are summarized in Fig. 24, where the total energy carried off by gravitational waves *E* is plotted vs $J/M^2$ for various perturbations. It is clear that over the range of dimensionless spin parameters, rotation influences the gravitational wave amplitude by about 2 orders of magnitude. When excluding the initial (potentially spurious) burst of gravitational waves, *E* scales roughly as $(J/M^2)^4$ and the models with velocity perturbations emit gravitational waves more strongly (total energy emitted is about 2 orders of magnitude larger than $2\%$ pressure perturbations).

**Fig. 25:**
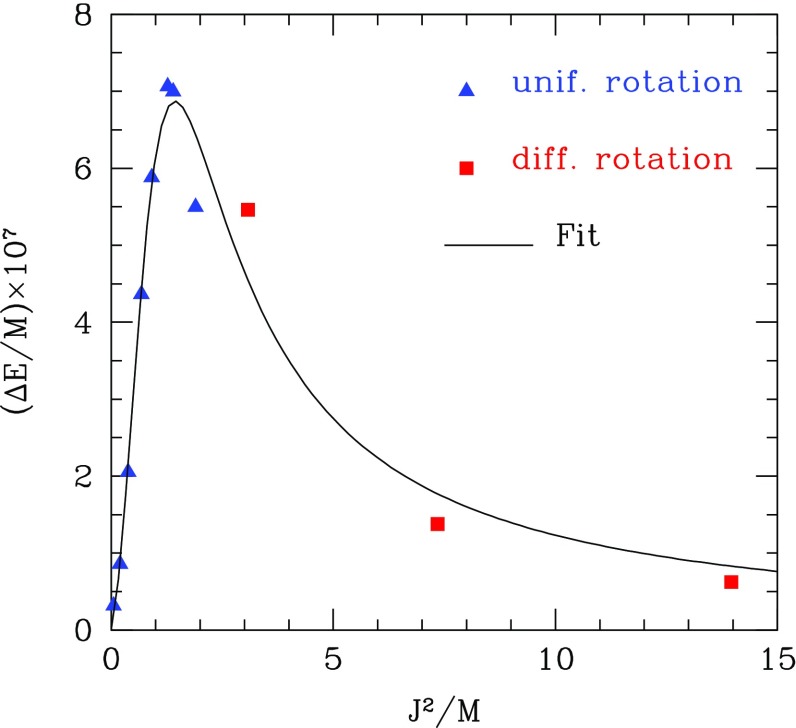
Energy carried off by gravitational waves, normalized to the total initial mass *M*, as a function of $J^2/M$ for collapsing, rapidly rotating neutron stars initially modeled as $n=1$ polytropes. Triangles represent uniformly rotating models, whereas the squares refer to the differentially rotating models discussed here. The solid line is the best fit (see Eq. (138)). (Image reproduced with permission from Giacomazzo et al. 2011, copyright by APS)

In Giacomazzo et al. (2011), in addition to addressing cosmic censorship, they also extended these results by considering the gravitational wave emission arising from differentially rotating, collapsing neutron stars with initially $J/M^2 > 0.54$. They consider the energy lost in gravitational waves as a function of $J^2/M$ (which is proportional to the initial quadrupole moment). They find that as $J^2/M$ increases past $J^2/M \approx 1$, *E* decreases (see Fig. 25) and suggest the following fitting formula for the energy carried off by gravitational waves

138 $$\begin{aligned} \frac{E}{M}=\frac{(J^2/M)^{n_1}}{a_1(J^2/M)^{n_2}+a_2}, \end{aligned}$$

where $n_1=1.43\pm 0.74$, $n_2=2.63\pm 0.53$, $a_1=(5.17\pm 4.37)\times 10^{5}$, $a_2=(1.11\pm 0.57)\times 10^{5}$. The authors also analyze the signal-to-noise ratio for these sources assuming a fiducial distance of 10 kpc, finding that ratios of order 50 are possible for advanced LIGO and VIRGO, around 1700 for the Einstein telescope, thus these objects could be detectable by third-generation, ground-based laser interferometers at distances of $\sim 1\,\mathrm {Mpc}$.

Collapse of the NS can potentially occur not only to a black hole but also to a hybrid quark star, i.e., a compact star with a deconfined quark matter core and outer layers made of neutrons. The collapse can proceed through a first-order phase transition in the core. Following up on the Lin et al. (2006) study, this scenario (known as phase-transition-induced collapse) is studied in Abdikamalov et al. (2009) via axisymmetric, general relativistic hydrodynamic simulations adopting the conformal flatness approximation using the CoCoNut code (Dimmelmeier et al. 2005). The initial neutron star models are both nonrotating and rotating, $\varGamma =2$ polytropes. The evolution adopts an approximate, phenomenological hybrid equation of state: a $\varGamma$-law ($\varGamma =2$) is adopted for hadronic matter—“hm” for short—(rest-mass density ($\rho _0$) such that $\rho _0 < \rho _\mathrm{hm}=6.97\times 10^{14}\,\mathrm {g\ cm}^{-3}$), the EOS of the MIT bag model for massless and non-interacting quarks at zero temperature is adopted for the quark matter—“qm” for short—($\rho _0 > \rho _\mathrm{qm}=9\rho _\mathrm{nuc}$, where $\rho _\mathrm{nuc}=2.7\times 10^{14}\,\mathrm {g\ cm}^{-3}$ is the nuclear saturation density), and a linear combination of the two for densities $\rho _\mathrm{hm} \le \rho _0 \le \rho _\mathrm{qm}$. The gravitational waves emitted by the collapsing NS are computed using the quadrupole formula integrated in time as in Dimmelmeier et al. (2002a). Abdikamalov et al. find that the emitted gravitational-wave spectrum is dominated by the fundamental quasi-radial and quadrupolar pulsation modes, but that the strain amplitudes are much smaller than suggested previously by Newtonian simulations (Lin et al. 2006). Therefore, it will be challenging to detect gravitational waves from phase-transition-induced collapse.

The gravitational waveforms from collapse have also been studied through a new code adopting multi-patch methods in Reisswig et al. (2013a), by Dietrich and Bernuzzi (2015), and through simulations of the accretion-induced collapse of neutron stars by Giacomazzo and Perna (2012).

#### Dynamical bar-mode instability

**Fig. 26:**
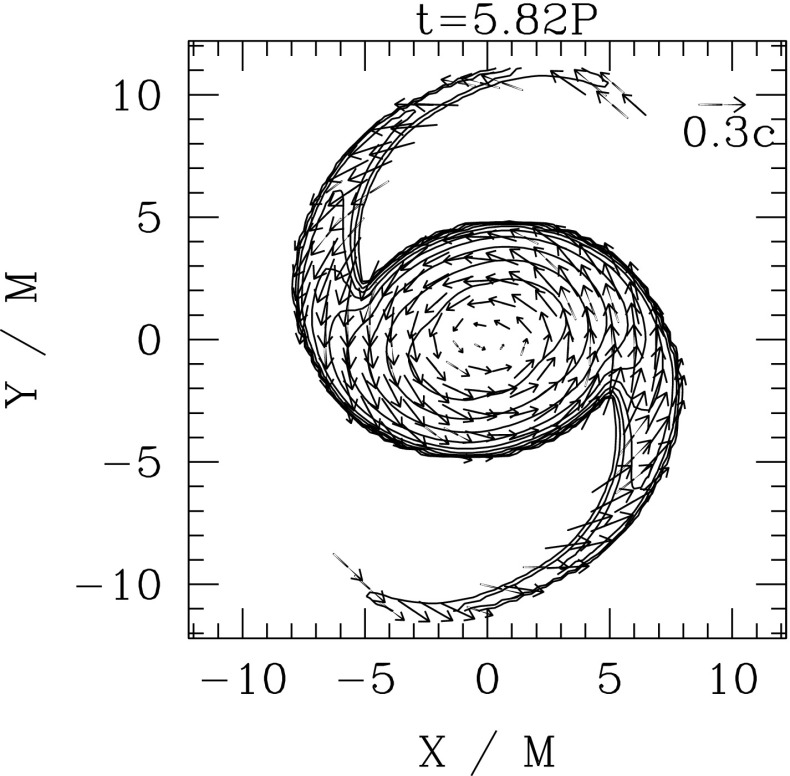
Density contours and velocity flow for a neutron star model that has developed spiral arms, due to the dynamical bar-mode instability. The computation was done in full General Relativity. (Image reproduced with permission from Shibata et al. 2000a, copyright by AAS)

Shibata et al. (2000a) study the dynamical bar-mode instability in differentially rotating neutron stars, in fully relativistic 3D simulations. They find that stars become unstable when rotating faster than a critical value of $\beta \equiv T/|W| \sim 0.24 {\,-\,} 0.25$. This is only somewhat smaller than the Newtonian value of $\beta \sim 0.27$. Models with rotation only somewhat above critical become differentially rotating ellipsoids, while models with $\beta$ much larger than critical also form spiral arms, leading to mass ejection, see for example Fig. 26. In any case, the differentially rotating ellipsoids formed during the bar-mode instability have $\beta >0.2$, indicating that they will be secularly unstable to bar-mode formation (driven by gravitational radiation or viscosity). The decrease of the critical value of $\beta$ for dynamical bar formation due to relativistic effects has been confirmed by post-Newtonian simulations (Saijo et al. 2001).

Shibata and Sekiguchi (2005b) study nonaxisymmetric dynamical instabilities in the context of (differentially) rotating stellar core collapse. The initial data corresponding to rotating, $\varGamma =4/3$ polytropic models with maximum rest-mass density $10^{10}\,\mathrm {g/cm}^3$, various degrees of differential rotation and values for the rotational parameter $\beta =T/|W|$ ranging from 0.00232 to 0.0263. The adopted differential rotation law is given by

139 $$\begin{aligned} u^tu_\phi = \varpi ^2 (\varOmega _a-\varOmega ), \end{aligned}$$

where $\varOmega =u^\phi /u^t$, $\varOmega _a$ is the angular velocity at the location of the rotation axis, and $\varpi _d$ is a constant.

For the evolution a hybrid $\varGamma$-law equation of state is adopted that has a cold part and a thermal part. The cold part has polytropic exponent $\varGamma _1$ for densities less than the nuclear density and $\varGamma _2$ otherwise. Most of the models in this study are evolved using $\varGamma _1=4/3$ and $\varGamma _2=2$, but other values are considered, too. The thermal part $\varGamma _\mathrm{th}=\varGamma _1$. The early stage of the collapse is followed by an axisymmetric hydrodynamic code in full GR and when the core becomes sufficiently compact—the minimum value of the lapse function becomes 0.8–0.85—they add a bar-mode density perturbation and after resolving the Hamiltonian and momentum constraints assuming conformal flatness and maximal slicing the evolution is followed using a 3D code. They find that a dynamical bar more instability can occur in newly formed neutron stars following the collapse of a stellar core, and that $\beta$ can be amplified even beyond the value critical value $\sim 0.27$ when: (i) the initial stellar model is highly differentially rotating $\varpi _d/R_e \lesssim 0.1$, where $R_e$ is the equatorial radius of the star; (ii) the initial value of the rotational parameter is in the range $0.01 \lesssim \beta _\mathrm{init} \lesssim 0.02$; (iii) the initial star is massive enough to become sufficiently compact so that it is rapidly spinning, but less massive than the critical mass value that leads to catastrophic collapse to a black hole. They find that the maximum $\beta$ value reached by a proto-neutron star is 0.36 for a stiff equation of state with $\varGamma _2=2.75$.

Baiotti et al. (2007) use the Whisky code to perform 3D hydrodynamic studies of the dynamical bar mode instability in full GR. The initial data used correspond to equilibrium, differentially rotating, polytropic neutron star models with $\varGamma =2$, $K=100$ and have a constant rest mass of $M_0\approx 1.51\,M_{\odot }$ and various initial $\beta$ parameters. In contrast to earlier studies that added an $m=2$ perturbation to trigger the bar mode instability, in this work the instability is triggered primarily by truncation error and additional simulations are performed to investigate the effects of initial $m=1$ and $m=2$ perturbations. The evolutions adopt of $\varGamma$-law equation of state. They find that: (i) An initial $m=1$ or $m=2$ mode perturbation affects the lifetime of the bar, but not the growth timescale of the instability, unless the initial $\beta$ is near the threshold value for instability; (ii) For models with $\beta \sim \beta _{c}$ imposing $\pi$ symmetry can radically change the dynamics and extend the lifetime of the bar. However, this does not hold for models with initial $\beta \gg \beta _c$, in which case even symmetries cannot produce long-lived bar; (iii) The bar lifetime depends strongly on the ratio $\beta /\beta _c$ and is generally of the order of the dynamical timescale ranging from $\sim 6$ to $\sim 24\,\mathrm {ms}$ (for comparison the initial equatorial period considered range from 2 to $3.9\,\mathrm {ms}$); (iv) Nonlinear mode-coupling takes place during the instability development, i.e, an $m=2$ mode also excites an $m=1$ mode and vice versa. This mixing can severely limit the bar lifetime and even suppress the bar.

**Fig. 27:**
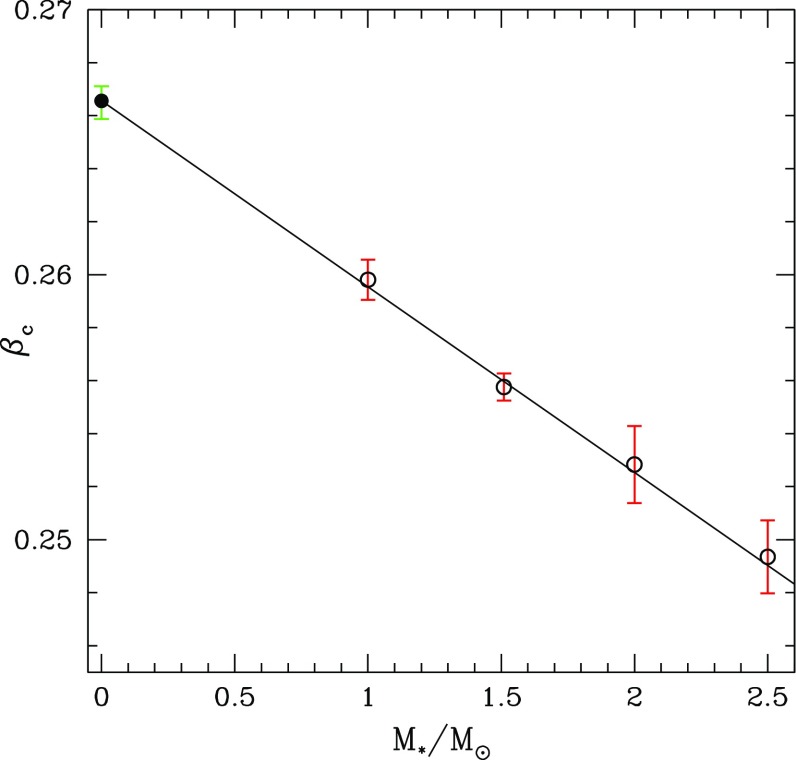
The open circles represent the extrapolated $\beta _c$ as a function of the stellar rest-mass (for the masses considered the compactness also increases along the positive horizontal axis). The filled circle represents the Newtonian limit for $\beta _c$. The plot corresponds to $\varGamma =2$ polytropes. (Image reproduced with permission from Manca et al. 2007, copyright by IOP)

Manca et al. (2007) use the same methods as in Baiotti et al. (2007) to analyze the effects of initial stellar compactness on $\beta _c$ for the onset of the dynamical bar-mode instability through hydrodynamic simulations in full GR. They evolve four sequences of models of constant baryonic mass ($1.0\,M_{\odot }$, $1.51\,M_{\odot }$, $2.0\,M_{\odot }$, and $2.5\,M_{\odot }$,) for a total of 59, $\varGamma =2$-polytropic stellar models. Using an extrapolation technique for these models they estimate $\beta _c$ for each constant-mass sequence, finding that the higher the initial compactness the smaller $\beta _c$ becomes. Their results are summarized in Fig. 27. In addition to the dependence of $\beta _c$ on the compactness, it is also found that for stars with sufficiently large mass (rest-mass greater than $2.0\,M_{\odot }$) and compactness (greater than $\sim 0.1$), the fastest growing mode corresponds to $m=3$, and that for all 59 models the nonaxisymmetric instability occurs on a dynamical timescale with the $m=1$ mode being dominant toward the final stages of the instability.

Recently, De Pietri et al. (2014) and Löffler et al. (2015) use the Einstein Toolkit (Löffler et al. 2012) to perform a study very similar to the one in Manca et al. (2007) but changing the polytropic exponent to $\varGamma =2.25,2.5,2.75,3.0$ and considering five constant-mass sequences of differentially rotating equilibrium neutron stars with masses $0.5\,M_{\odot }$, $1.0\,M_{\odot }$, $1.5\,M_{\odot }$, $2.0\,M_{\odot }$, and $2.5\,M_{\odot }$. Using a similar extrapolation method as in Manca et al. (2007) they find that the threshold value for $\beta$ is reduced by $\sim 5\%$ when compared to the $\varGamma =2$ case, concluding that a stiffer, realistic equation of state is expected to have smaller $\beta _c$ for the onset of the dynamical bar-mode instability.

Franci et al. (2013) adopt the Whisky code to perform magnetohydrodynamic simulations in full GR in order to study the effects of magnetic fields on the development of the bar-mode instability. The initial $\varGamma =2$ polytropic, differentially rotating, equilibrium stars are seeded with an initially purely poloidal magnetic field confined in the neutron star interior. The magnitude of the magnetic field at the center of the star is chosen in the range $10^{14}$–$10^{16}\,\mathrm {G}$. Their magnetohydrodynamic calculations show that strong initial magnetic fields, $B\gtrsim 10^{16}\,\mathrm {G}$, can suppress the instability completely, while smaller magnetic fields have negligible impact on the instability.

We note here that some preliminary studies in full GR of the low-*T* / |*W*|, bar-mode ($m=2$) instability have been carried out in Cerdá-Durán et al. (2007), Corvino et al. (2010) and De Pietri et al. (2014) via hydrodynamic simulations and in Muhlberger et al. (2014) via magnetohydrodynamic simulations, where it was shown that magnetic fields can suppress the development of the instability, but only for a narrow range of the magnetic field strength.

### Pulsations of rotating stars

**Fig. 28:**
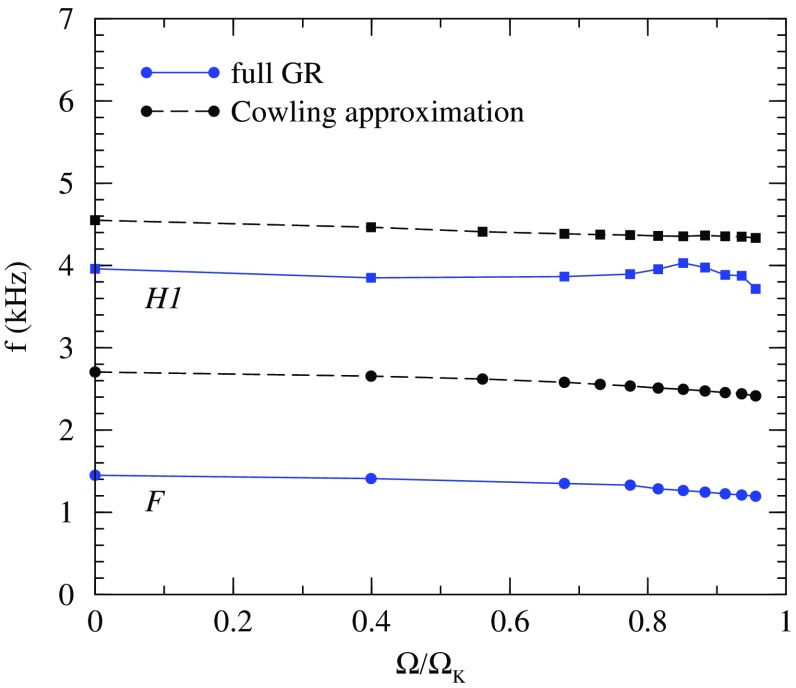
The first fully relativistic, quasi-radial pulsation frequencies for a sequence of rapidly rotating $N=1$ polytropes, up to the mass-shedding limit (with fixed central density along the sequence). The frequencies of the fundamental mode *F* (filled circles) and of the first overtone $H_1$ (filled squares) are obtained through *coupled* hydrodynamical and spacetime evolutions (blue solid lines). The corresponding frequencies obtained from computations in the relativistic Cowling approximation (fixed spacetime) (Font et al. 2001) are shown as black dashed lines. (Image reproduced with permission from Font et al. 2002, copyright by APS)

Pulsations of rotating relativistic stars are traditionally studied (when possible) as a time independent, linear eigenvalue problem, but recent advances in numerical relativity also allow the study of such pulsations via numerical time evolutions. Quasi-radial mode frequencies of rapidly rotating stars in full general relativity have been obtained in Font et al. (2002), something that has not been achieved yet with linear perturbation theory. The fundamental quasi-radial mode in full general relativity has a similar rotational dependence as in the relativistic Cowling approximation, and an empirical relation between the full GR computation and the Cowling approximation can be constructed (Fig. 28). For higher order modes, apparent intersections of mode sequences near the mass-shedding limit do not allow for such empirical relations to be constructed.

For a comparison study of linear and non-linear evolution methods, in the case of nonrotating polytropic models, as well as for a comparison of different gravitational wave extraction techniques see, e.g., Baiotti et al. (2009)

In the relativistic Cowling approximation, 2D time evolutions have yielded frequencies for the $l=0$ to $l=3$ axisymmetric modes of rapidly rotating relativistic polytropes with $N=1.0$ (Font et al. 2001). The higher order overtones of these modes show characteristic apparent crossings near mass-shedding (as was observed for the quasi-radial modes in Yoshida and Eriguchi 2001). For a recent code aimed at computing oscillation frequencies adopting the conformal flatness approximation see Yoshida (2012), who finds good agreement between *f* and *p*-mode frequencies when compared to the full theory in the case of slowly rotating stars.

Numerical relativity has also enabled the first study of nonlinear *r*-modes in rapidly rotating relativistic stars (in the Cowling approximation) by Stergioulas and Font (2001). For several dozen dynamical timescales, the study shows that nonlinear *r*-modes with amplitudes of order unity can exist in a star rotating near mass-shedding. However, on longer timescales, nonlinear effects may limit the *r*-mode amplitude to smaller values (see Sect. 4.5.3).

In another study, Siebel et al. (2002) perform spherically symmetric simulations in full GR of the Einstein–Klein–Gordon-perfect fluid system to study the interaction of a scalar field with a spherical neutron star using a characteristic approach to solve the dynamical equations. The initial data correspond to a TOV, $\varGamma =2$ polytropic neutron star for the fluid and metric variables, and a Gaussian pulse is chosen for the initial scalar field. For a small amplitude scalar field pulse, radial oscillations are excited on the NS, and a Fourier analysis shows that the oscillation frequencies corresponding to the fundamental, first and second overtone modes computed through the full non-linear evolution are very close to those of a linearized analysis, but generally smaller by $\lesssim 1$–$2\%$.

The gravitational waveforms from oscillating spherical and rigidly rotating (near the mass shedding limit), $\varGamma =2$ polytropic neutron stars have been computed by Shibata and Sekiguchi (2004). The equilibrium polytropic stars were perturbed using a velocity perturbation with magnitude 0.1*c* at the neutron star surface and evolved using a fully general relativistic hydrodynamics code in axisymmetry. Gauge invariant methods and a quadrupole formula were used to compute the gravitational wave signature and it is found that the wave phase and modulation of the amplitude can be computed accurately using a quadrupole formula but not the amplitude. It is also found that for both spherical and rotating stars the gravitational wave frequency is associated with the fundamental $l=2$ mode, and that for rotating stars another frequency in the gravitational wave signal is detected, which is likely associated with the quasiradial oscillation $p_1$ mode.

Stergioulas et al. (2004) perform 2D hydrodynamic simulations of differentially rotating neutron stars to study non-linear pulsations in the Cowling approximation. It is found that for $\varGamma =2$ polytropic stars near the mass shedding limit, shocks forming near the stellar surface damp the oscillations and this mechanism may set a small saturation amplitude for modes that are unstable to the emission of gravitational waves.

**Fig. 29:**
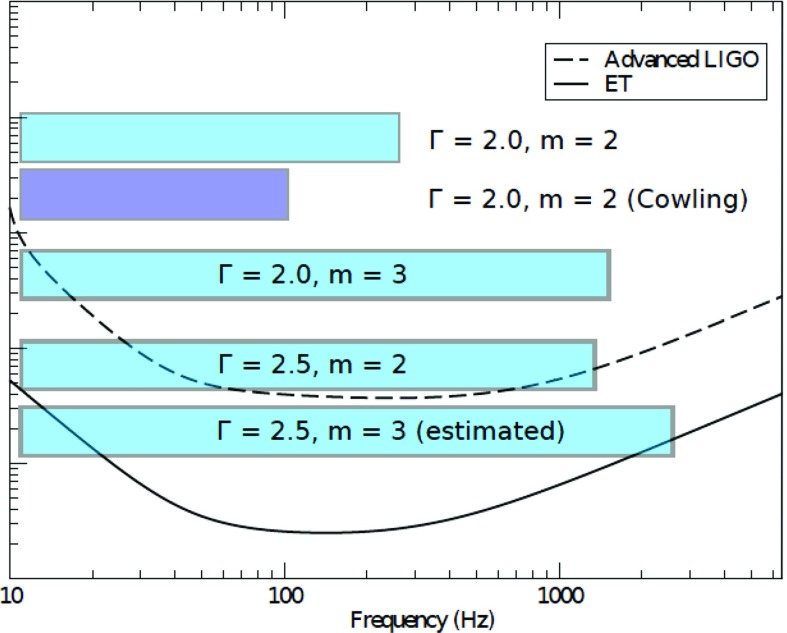
Frequency band of the CFS instability based on $m = 2$ and $m = 3$ f -mode oscillations for two sequences of rigidly rotating polytropic neutron stars with $\varGamma =2$ and $\varGamma =2.5$. Each band is limited on the left by the neutral point and on the right by the frequency of the counter-rotating mode in the most rapidly rotating model. The sensitivity noise curves of aLIGO and the future Einstein Telescope are also plotted. (Image reproduced with permission from Zink et al. 2010, copyright by APS)

Zink et al. (2010) study the location of the neutral-points for the $l=|m| = 2$ and $l=|m| = 3$ *f*-mode oscillations of uniformly rotating polytropes using 2D, hydrodynamic simulations both in the Cowling approximation and in full general relativity. These frequencies are important because rapidly rotating neutron stars can become unstable to the gravitational-wave-driven CFS instability. A polytropic equation of state is adopted both for the construction of initial data (generated with the RNS code) and for the evolution. To assess the effects of the stiffness of the equation of state, two sequences of models are considered: one with $\varGamma =2$ and one with $\varGamma =2.5$. The rotational parameter $\beta$ ranges from 0 to 0.08 for the $\varGamma =2$ sequence, and from 0 to 0.12 for the $\varGamma =2.5$ sequence. All models are chosen to have an initial central rest-mass density close to that of the maximum mass TOV star with the same equation of state, i.e., $\rho _c=2.6823\times 10^{-3} (100/K)$ ($\rho _c=5.0\times 10^{-3} (1000/K)^{3/2}$) for $\varGamma =2$ ($\varGamma =2.5$). To excite the modes the authors add a small-amplitude, $l=|m|$ density perturbation and evolve the initial data. The authors find that the Cowling approximation in some cases underestimates the lower limit of the *f*-mode CFS instability window by 10% and the upper limit by 60%. The authors conclude that general relativity enhances the detectability of a CFS-unstable neutron star substantially and derive limits on the observable gravitational-wave frequency band available to the instability, finding. These results are summarized in Fig. 29.

Kastaun et al. (2010) perform axisymmetric and 3D general relativistic hydrodynamic simulation in the Cowling approximation using the PIZZA code (Kastaun 2006) to qualitatively understand what mechanisms set the saturation amplitude of the *f*-mode instability. Using the RNS code they build initial data corresponding to uniformly rotating neutron star models with various masses and degree of rotation and polytropic indices $n=2,1,0.6849$. For one of the models studied (MA65) the $l=m=2$ *f*-mode is excited by the CFS instability in full GR, and hence is a candidate for detectable gravitational waves. To excite high amplitude oscillations, they linearly scale the eigenfunctions of specific energy and 3-velocity, and add them to the background solution. The primary focus is on the $l=|m|=2$ and $l=2,\ m=0$ modes. It is found that the saturation amplitude of high-amplitude axisymmetric *f*-mode oscillations is rapidly determined by shock formation near the surface of the star. It is also found that stiffer EOSs allow higher amplitudes before shocks begin to dissipate the *f*-modes, and rotation affects the damping of axisymmetric modes only weakly, until the Kepler limit is reached, at which point damping occurs via mass shedding. For nonaxisymmetric *f*-mode oscillations, the saturation amplitude is not determined by shocks, but is primarily determined by damping due to wave breaking and non-linear mode coupling.

In follow up work, Kastaun (2011) performs relativistic hydrodynamical simulations in the Cowling approximation to study non-linear, *r*-mode oscillations using the PIZZA code. The initial data correspond to two rigidly rotating, $\varGamma =2$ polytropic models of neutron stars with rest-masses $1.6194\,M_{\odot }$ and $1.7555\,M_{\odot }$, and ratio of polar to equatorial radius 0.85 and 0.7, respectively. The initial data are seeded with an $l=m=2$ perturbation based on the eigenvectors from linearized studies of *r*-mode oscillations, and then scaled to larger amplitudes such that the energy in the *r*-mode divided by the stellar binding energy is of order $10^{-3}$. The initial data are then evolved adopting a polytropic equation of state for the hydrodynamics. It is found that the frequencies of the *r*-modes in the inertial frame agree to better than 0.1% with those found by linearized studies in Krüger et al. (2010). As found in earlier studies, Kastaun finds that differential rotation develops during the evolution of the *r*-mode with high initial amplitude. Related with the onset of differential rotation is also the decay of the *r*-modes, which is why it is hypothesized that the saturation of the differential rotation should occur when the *r*-mode decay due to differential rotation is balanced by the differential rotation due to the presence of the *r*-mode. Kastaun also argues that for the models under study the *r*-mode decay is not due to shock formation near the stellar surface. Finally, it is pointed out that while mode-mode coupling is probably not the main cause of the *r*-mode decay for these models, as found for other models in Gressman et al. (2002), Lin and Suen (2006) by Newtonian simulations, mode-mode coupling cannot be ruled out as a possible cause for the *r*-mode decay.

Note that the perturbative work by Chugunov (2015) finds that, for the stable *r*-mode, differential rotation is pure gauge, reflecting only the differential rotation in the initial conditions. For the unstable *r*-mode, Friedman et al. (2016) find that the 2nd-order differential rotation is unique.

It should be noted that the work of Kastaun should be considered preliminary, because the presence of magnetic fields could brake the differential rotation (Rezzolla et al. 2000, 2001a, b) and also give rise to a turbulent environment. For example, for large enough amplitudes Rezzolla et al. (2000) argue that the magnetic fields could grow large enough to completely suppress the *r*-mode instability. Hence, a magnetohydronamic study is required to fully understand this feedback mechanism. In a recent work, Friedman et al. (2017) explore how much differential rotation, that is induced by the *r*-mode instability can boost the magnetic fields. The authors argue that magnetic-field amplification is restricted in strength by the saturation amplitude of the unstable mode, and if the saturation amplitude is weak enough $\lesssim 10^{-4}$, then the magnetic field cannot grow to levels that suppress or modify that *r*-mode in neutron stars with “normal” and type II superconducting interiors.

### Rotating core collapse

#### Collapse to a rotating black hole

Black hole formation in relativistic core collapse was first studied in axisymmetry by Nakamura (1981, 1983), using the $(2+1)+1$ formalism (Maeda et al. 1980). The outcome of the simulation depends on the rotational parameter

140 $$\begin{aligned} q \equiv J/M^2. \end{aligned}$$

A rotating black hole is formed only if $q<1$, indicating that cosmic censorship holds. Stark and Piran (1985), Piran and Stark (1986) use the $3+1$ formalism and the radial gauge of Bardeen–Piran (Bardeen and Piran 1983) to study black hole formation and gravitational wave emission in axisymmetry. In this gauge, two metric functions used in determining $g_{\theta \theta }$ and $g_{\phi \phi }$ can be chosen such that at large radii they asymptotically approach $h_+$ and $h_\times$ (the even and odd transverse traceless amplitudes of the gravitational waves, with 1 / *r* fall-off at large radii; note that $h_+$ defined in Stark and Piran 1985 has the opposite sign as that commonly used, e.g., in Thorne 1983). In this way, the gravitational waveform is obtained at large radii directly in the numerical evolution. It is also easy to compute the gravitational energy emitted, as a simple integral over a sphere far from the source: $\varDelta E \sim r^2\int dt(h_{+,r}^2+h_{\times ,r}^2)$. Using polar slicing, black hole formation appears as a region of exponentially small lapse, when $q<\mathcal{O}(1)$. The initial data consists of a nonrotating, pressure deficient TOV solution, to which angular momentum is added by hand. The obtained waveform is nearly independent of the details of the collapse: It consists of a broad initial peak (since the star adjusts its initial spherical shape to a flattened shape, more consistent with the prescribed angular momentum), the main emission (during the formation of the black hole), and an oscillatory tail, corresponding to oscillations of the formed black hole spacetime. The energy of the emitted gravitational waves during the axisymmetric core collapse is found not to exceed $7\times 10^{-4}\,M_{\odot } c^2$ (to which the broad initial peak has a negligible contribution). The emitted energy scales as $q^4$, while the energy in the even mode exceeds that in the odd mode by at least an order of magnitude. The qualitative morphology of the gravitational wave signal from collapse has been studied through perturbative approaches by Seidel and Moore (1987), Seidel et al. (1988), and Seidel (1990).

Shibata (2000) carried out axisymmetric simulations of rotating stellar collapse in full general relativity, using a Cartesian grid, in which axisymmetry is imposed by suitable boundary conditions. The details of the formalism (numerical evolution scheme and gauge) are given in Shibata (1999b). It is found that rapid rotation can prevent prompt black hole formation. When $q=\mathcal{O}(1)$, a prompt collapse to a black hole is prevented even for a rest mass that is 70–80% larger than the maximum allowed mass of spherical stars, and this depends weakly on the rotational profile of the initial configuration. The final configuration is supported against collapse by the induced differential rotation. In these axisymmetric simulations, shock formation for $q<0.5$ does not result in a significant heating of the core; shocks are formed at a spheroidal shell around the high density core. In contrast, when the initial configuration is rapidly rotating ($q=\mathcal{O}(1)$), shocks are formed in a highly nonspherical manner near high density regions, and the resultant shock heating contributes in preventing prompt collapse to a black hole. A qualitative analysis in Shibata (2000) suggests that a disk can form around a black hole during core collapse, provided the progenitor is nearly rigidly rotating and $q=\mathcal{O}(1)$ for a stiff progenitor EOS. On the other hand, $q \ll 1$ still allows for a disk formation if the progenitor EOS is soft. At present, it is not clear how much the above conclusions depend on the restriction to axisymmetry or on other assumptions—3-dimensional simulations of the core collapse of such initially axisymmetric configurations have still to be performed.

Shibata and Shapiro perform axisymmetric, hydrodynamic simulations in full general relativity to follow the collapse of a rigidly rotating, supermassive star (SMS) to a supermassive black hole (SMBH) in Shibata and Shapiro (2002). The initial, equilibrium $\varGamma =4/3$ polytropic, SMS of arbitrary mass *M* rotates at the mass-shedding limit, is marginally unstable to collapse, and has $T/|W| \simeq 0.009$ and $J/M^2\simeq 0.97$. The collapse is induced via a $1\%$ pressure depletion and proceeds homologously early on, until eventually an apparent horizon forms at the center. Shibata and Shapiro estimate that the final black hole will contain $\sim 90\%$ of the total mass of the system and have a spin parameter $J/M^2 \sim 0.75$, with the remaining gas forming a disk around the black hole. In follow up work, Liu et al. (2007) study the magnetorotational collapse of supermassive stars in axisymmetry and find that following black hole formation the magnetic-field lines partially collimate along the hole’s spin axis speculating that these systems may be able to launch jets.

In a more recent studies in full general relativity, Shibata et al. (2016) and Sun et al. (2017) study the gravitational wave emission from such collapsing, rotating supermassive stars. Both studies find that for stellar masses $M\sim 10^6\,M_{\odot }$, the gravitational waves peak in the LISA band and could be detectable out to cosmological redshift $z\sim 3$. Sun, Paschalidis, Ruiz and Shapiro also point out that for supermassive stars with mass $M\sim 10^4$, the gravitational waves from collapse could be detectable by DECIGO/BBO out to redshift $z\sim 11$. This scenario is very interesting because future space-based gravitational wave observatories have the potential to probe whether supermassive stars exist and can form seed black holes that later on could grow through accretion to form the supermassive black holes we observe at the centers of quasars as early as $z=7$.

**Fig. 30:**
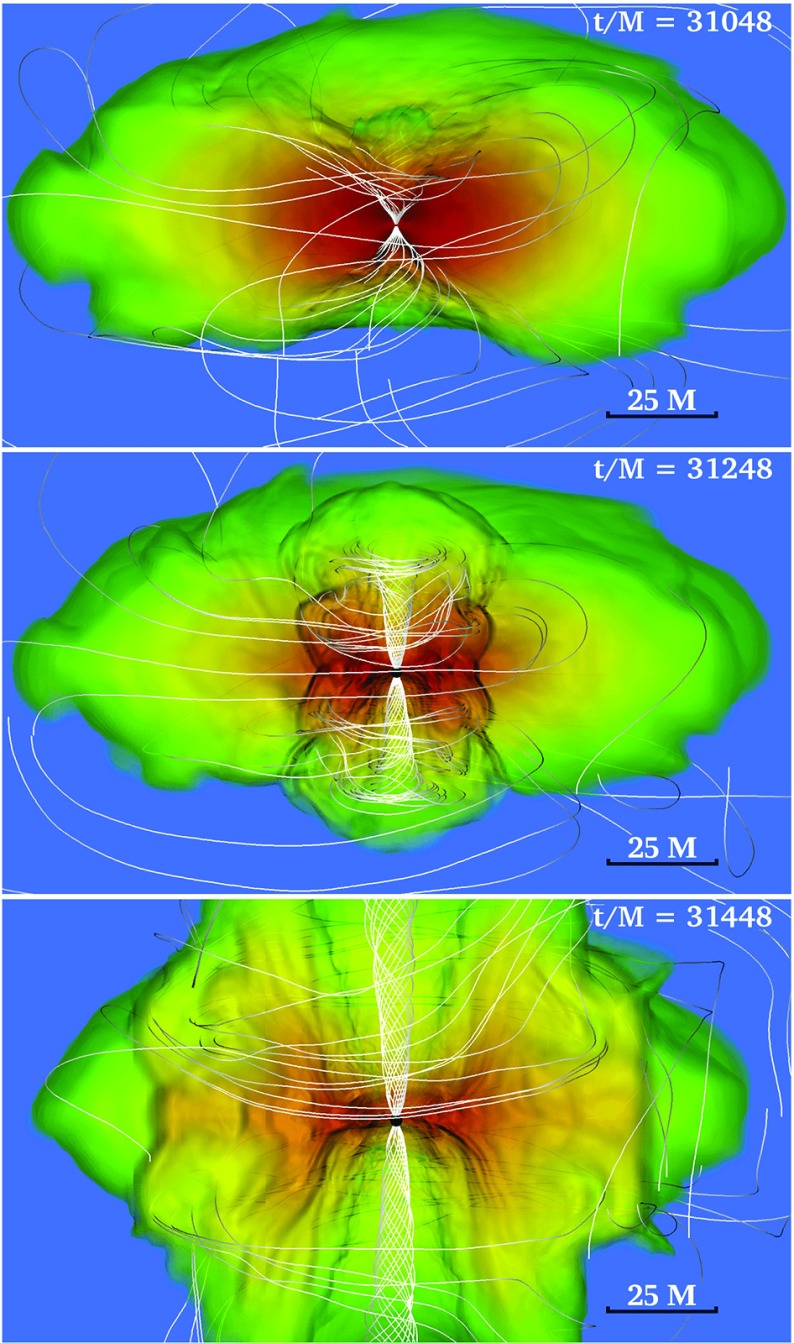
Cut of 3D rest-mass density profile (colored volume rendering) of a magnetized rotating, collapsing supermassive star with magnetic-field lines indicated by white curves. The top panel corresponds to the time near BH formation, the middle panel is during the development of the incipient jet, and the bottom panel shows the fully developed incipient jet. (Image reproduced with permission from Sun et al. 2017, copyright by APS)

Sun et al. (2017) also investigate the effects of magnetic fields and find that shortly after black hole formation the black hole - accretion disk engine that forms can launch jets (see Fig. 30) with characteristic jet luminosity $L_\mathrm{jet} \sim 10^{51}\,\mathrm {erg\ s}^{-1}$ which could be observable as a very long gamma-ray burst by current satellites such as Swift. Thus, collapsing supermassive stars could be multimessenger sources.

In a recent work, Uchida et al. (2017) perform axisymmetric calculations in full general relativity to investigate the effects of nuclear burning in the collapse of supermassive stars, finding that the collapse proceeds nearly unaffected. In addition, they find that if a supermassive star core is sufficiently rapidly rotating about 1% of the initial rest-mass becomes unbound with characteristic velocity and kinetic energy 0.2*c* and $10^{54-56}$ erg.

A 3D dimensional hydrodynamics code capable of following the collapse of a massive relativistic star in full general relativity is presented in Font et al. (2002). A different numerical code for axisymmetric gravitational collapse in the $(2+1)+1$ formalism is described in Choptuik et al. (2003).

**Fig. 31:**
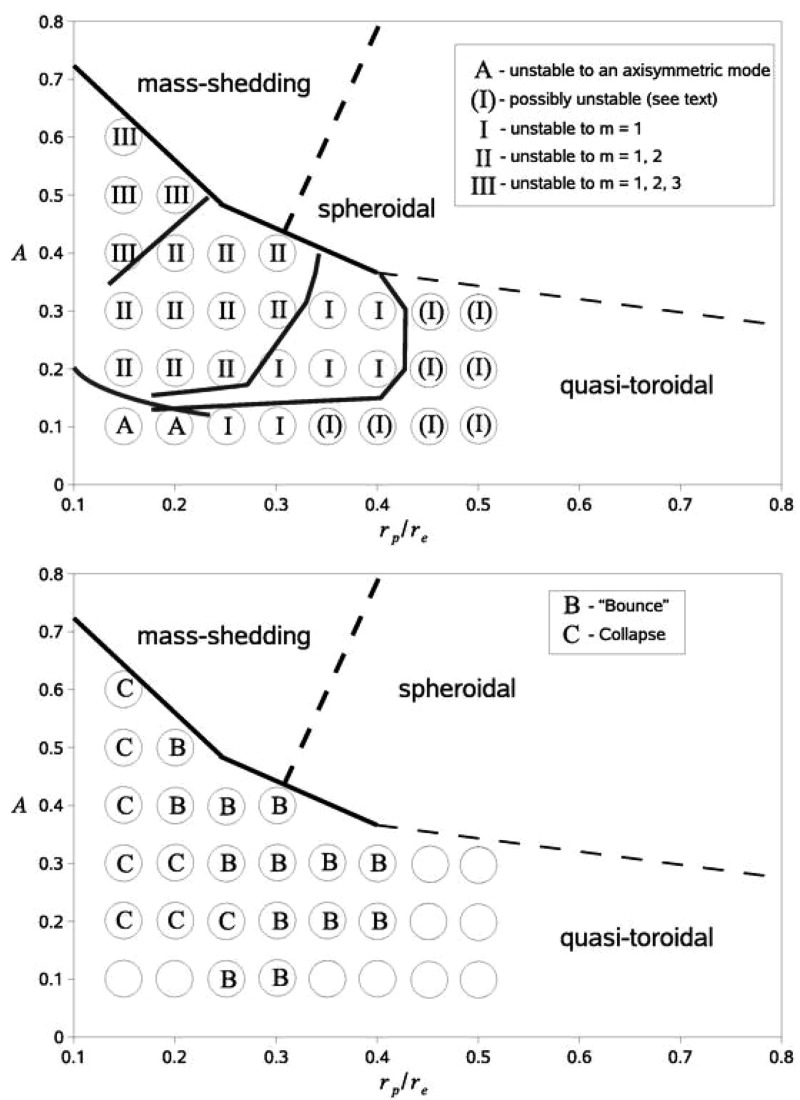
Top panel: Stability of quasi-toroidal models with $$\rho _c =10^{-7}$$ on the *A*–$$r_p/r_e$$ plane where *A* is a constant controlling the degree of differential rotation. (We point out that, here, *A* does not coincide with *A* in Eq. (45), but it equals the *A* appearing in that equation divided by the equatorial coordinate radius $$r_e$$). A Latin number denotes the highest azimuthal order more that becomes unstable, i.e., I implies that only $$m = 1$$ is unstable, II implies $$m = 1, 2$$ are unstable, and III implies $$ m = 1, 2, 3$$ are unstable. Models denoted by (I) are either secularly unstable—growth times $$\tau > t_\mathrm{dyn}$$ (where $$t_\mathrm{dyn}$$ is the dynamical timescale), or stable (see Zink et al. 2007). Models denoted by A exhibit an axisymmetric instability. The line in the lower left indicates the location of the sequence $$J/M^2 = 1$$, and the three lines inside the quasi-toroidal region indicate the locations of sequences with $$T/|W| = 0.14$$ (right), $$T/|W| = 0.18$$ (middle) and $$T/|W| = 0.26$$ (left). Lower panel: Remnants of the models from left panel, which are unstable with respect to nonaxisymmetric modes. The nonlinear behaviour has been analyzed by observing the evolution of the function minimum value of the lapse $$\alpha _{\min }$$ Models which show a minimum in this function are marked by B for bounce, while models exhibiting an exponential collapse of the lapse are marked by C for collapse. (Image reproduced with permission from Zink et al. 2007, copyright by APS)

Zink et al. (2006) perform hydrodynamic simulations of supermassive stars in full general relativity to study for the first time the off-center formation of a black hole through fragmentation of a general relativistic polytrope. They adopt the Cactus code and the Whisky module for the spacetime and hydrodynamics, in conjunction with a $\varGamma$ law equation of state. The initial data correspond to $n=3$ equilibrium polytrope that is differentially rotating with rotation law (45) $u^tu_\phi =A^2(\varOmega _c-\varOmega )$, where *A* is a constant that regulates the degree of differential rotation, $\varOmega _c$ is the angular velocity at the center of the star, and $\varOmega$ the angular velocity at a given (cylindrical) radius. The particular initial model they consider is generated using the RNS code, and has $A=r_e/3$, where $r_e$ is the equatorial coordinate radius of the star, a central density $\rho _c = 3.38\times 10^{-6}$ (in geometrized, polytropic units where $G=1=c=K$ and *K* is the polytropic constant), the ratio of the polar $r_p$ to equatorial radius is $r_p/r_3=0.24$, and $T/|W|=0.227$. After the initial data are generated small nonaxisymmetric density perturbations are added of the form

141 $$\begin{aligned} \rho \rightarrow \rho \left( 1+\frac{1}{\lambda r_e}\sum _{m=1}^{4}\lambda _mBr\sin (m\phi )\right) , \end{aligned}$$

where $\lambda _m=0,1$ and $\lambda =\sum _{m}\lambda _m$, and the collapse is induced by a $0.1\%$ pressure depletion. After the initial perturbation the constraints are not solved again because the amplitude *B* is chosen sufficiently small that the truncation-error induced constraint violation dominates. They find that the star is unstable to $m = 1$ and $m = 2$, and these modes grow from the linear to the nonlinear regime with time, and eventually, depending on the initial perturbation, lead to the formation of one or more off-center fragments. Using an adaptive-mesh refinement type of method, they follow the behavior in the case where one off-center fragment forms, and find that it collapses to form a black hole. Based on these results the authors argue that the fragmentation could turn a massive star into a binary black hole with a massive accretion disk around it. In a follow-up paper, Zink et al. (2007) use the same codes to study many more cases including different values for compactness, equation of state stiffness, and different axes ratios (corresponding to even low *T* / |*W*| models). It is found that: (1) the growth time of the $m = 1$ and $m = 2$ modes increases with lower $r_p/r_e$, (2) the $m = 1$ and $m = 2$ modes are stabilized with increasing $\varGamma$—stiffness of the equation of state—and *T* / |*W*| decreasing from 0.227 to 0.159, (3) the instability growth time is approximately similar for stars of different compactness (with *T* / |*W*| approximately constant), but the outcome of the fragmentation can differ drastically—the fragments of more compact stars $M/R \gtrsim 0.044$ seem to collapse and form black holes, but stars with low compactness $M/R \lesssim 0.022$ seem to prevent black hole formation. The results summarizing whether the instability develops and whether the fragments collapse to a black hole or not are presented in Fig. 31. Zink et al. also conclude that along a sequence of increasing *T* / |*W*| and restricted to a few dynamical timescales, the $m = 1$ perturbation is dominant before higher-order modes become unstable, suggesting the (off-center) formation of a single black hole with a massive accretion disk. However, the authors note that these results do not exclude the possibility that on a longer timescale a higher-order mode will be activated before the $m=1$ mode becomes unstable so that multiple black holes could form. The authors also find that in the cases where two fragments form and collapse, a runaway instability takes over, leading eventually to a central collapse.

Montero et al. (2012) perform axisymmetric, HRSC hydrodynamic simulations in full general relativity (adopting the BSSN formulation) to study the collapse and explosion of rotating supermassive stars while accounting for thermonuclear effects. Their simulations adopt an equation of state that accounts for the gas pressure, and the pressure associated with radiation and electron-positron pairs. In addition, they include the effects of thermonuclear energy released by hydrogen and helium burning and neutrino cooling through thermal processes. The initial models are $n=3$ polytropic, rigidly rotating equilibrium configurations constructed with the LORENE library. They find that nonrotating supermassive stars with a mass of $\sim 5 \times 10^5\,M_{\odot }$ and an initial metallicity less than $Z_{CNO} \sim 0.007$ collapse to a black hole, while the threshold metallicity is reduced to $Z_{CNO} \sim 0.001$ for uniformly rotating supermassive stars. The critical initial metallicity is increased for $10^6\,M_{\odot }$ stars. It is noted that, for some models, collapse to a black hole does not occur unless the effects of $e^{\pm }$ pairs are accounted for, which render the star unstable to gravitational collapse by reducing the effective adiabatic index. For the stars that collapse the evolution is continued past black hole formation, and the computed peak neutrino and antineutrino luminosities for all flavors is $L \sim 10^{55}\,\mathrm {erg/s}$.

**Fig. 32:**
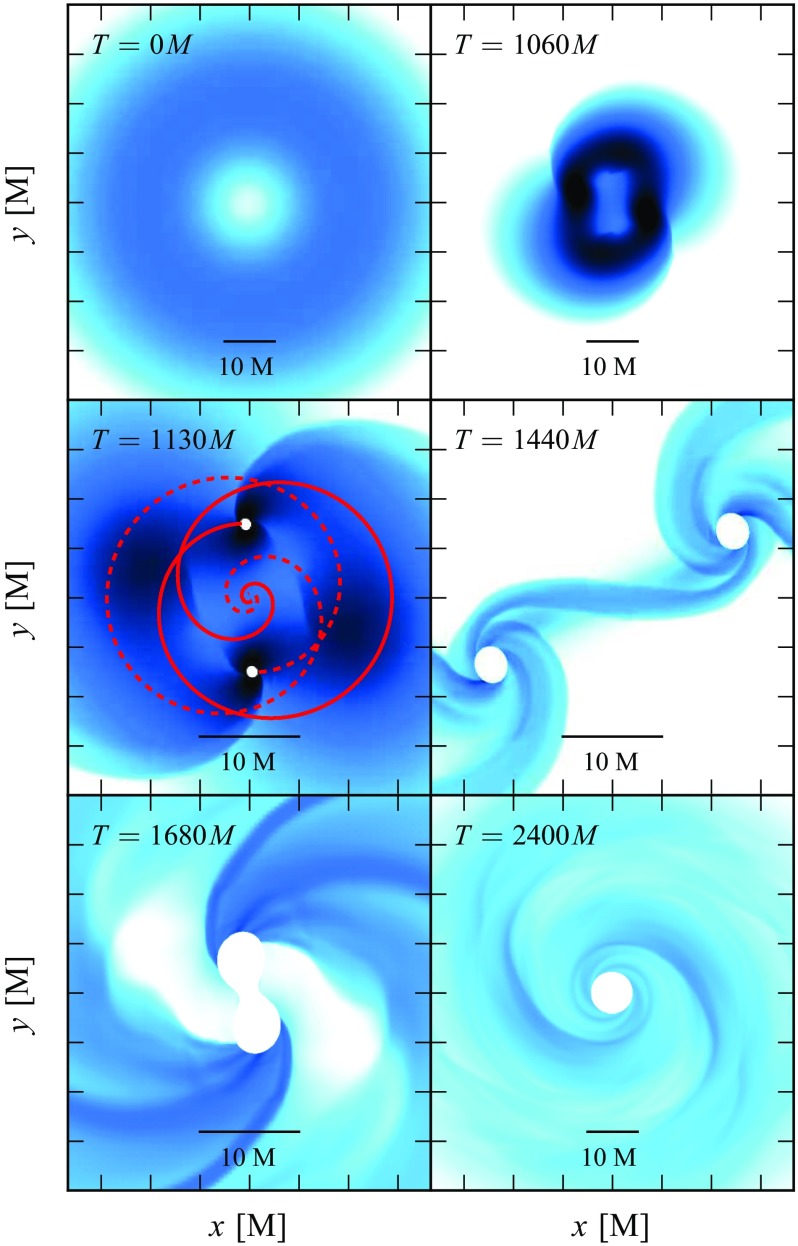
Equatorial density contours at select times of a fragmenting, collapsing supermassive star, forming a supermassive black hole binary. Dark colors indicate high density, light colors indicate low density. The logarithmic density colormap ranges from $10^{-7}M^{-2}$ (white) to $10^{-3}M^{-2}$ (black). In the bottom two panels, the colormap is rescaled to the range $[10^{-8}M^{-2}, 10^{-4}M^{-2}]$. The upper two and the bottom right panels show physical dimensions of $\pm \,40M$, while the remaining panels show physical dimensions of $\pm \,20M$. The white disks roughly indicate the black hole apparent horizons. (Image reproduced with permission from Reisswig et al. 2013b, copyright by APS)

Reisswig et al. (2013b) inspired by the results of Montero et al. (2012) revisit the fragmentation instabilities in differentially rotating supermassive stars studied in Zink et al. (2006, 2007) who, in turn, extended the configurations studied by Saijo (2004). However, instead of a $\varGamma =4/3$, they adopt a slightly softer $\varGamma =1.33$ equation of state and consider nonaxisymmetric perturbations of the form $\rho \rightarrow \rho (1 + A_m r \sin (m\phi ))$, on initially equilibrium $n=3$ polytropes with rotational parameter $J/M^2=1.0643$ which are generated with the RNS code. The hydrodynamic evolutions in full general relativity are performed using the Einstein Toolkit. For the case where only an $m=2$ perturbation is considered, the authors find that the initial star gives rise to two fragments which subsequently collapse and form a bound supermassive black hole binary, which in turn inspirals and merges in the gaseous environment of the star (see Fig. 32). The authors compute the associated gravitational wave signature and conclude that if $m = 2$, fragmentation and formation of a supermassive black hole binary occurs in supermassive stellar collapse, the coalescence of the binary will result in a unique gravitational wave signal that can be detected at redshifts $z \gtrsim 10$ with DECIGO and the Big Bang Observer, if the supermassive star’s mass is in the range $10^4$–$10^6\,M_{\odot }$.

Ott et al. (2011) use the Einstein Toolkit (Löffler et al. 2012) to perform 3D hydrodynamic simulations of rotating core collapse in full general relativity to study gravitational wave emission from collapsar model for long gamma-ray bursts. The initial data correspond to the inner $\sim 5700\,\mathrm {km}$ profile of the realistic 75-$M_\odot$, $10^{-4}$-solar metallicity model u75 of Woosley et al. (2002), which corresponds to the inner $\sim 4.5\,M_{\odot }$ of the star and imposing different rotational profiles. A hybrid, piecewise polytropic, $\varGamma$-law equation of state is adopted for the evolution (as in e.g., Shibata 2003a), such that $\varGamma _1= 1.31$ at subnuclear densities, and $\varGamma _2=2.4$ at supernuclear densities, and $\varGamma _\mathrm{th}=4/3$. Octant symmetry is imposed throughout the evolution and crude neutrino cooling is accounted for. Gravitational waves are extracted using Cauchy-characteristic matching as described in Reisswig et al. (2011). The initial data are evolved through collapse, core bounce, proto-neutron star (PNS) formation through collapse of the PNS to a BH. Gravitational waves are computed for all rotational profiles and it is found that the peak amplitude at bounce is approximately proportional to the model spin, and following bounce the signal is dominated by quadrupole motion due to turbulence behind the post-bounce shock. Following PNS collapse, a second burst in the waveform appears which corresponds to the BH formation, which subsequently rings down. As expected, characteristic gravitational wave frequencies are 1–3 kHz, and it is estimated that for such an event taking place 10 kpc away the signal-to-noise ratio for aLIGO will be $\sim 50$.

The collapsar scenario is also studied in DeBrye et al. (2013) through hydrodynamic simulations in general relativity adopting the conformal flatness approximation and performed using the CoCoNuT code. More detailed microphysics and a neutrino leakage scheme is implemented to account for deleptonization and neutrino cooling, and the initial stellar mass, metallicity, and rotational profile of the stellar progenitor are varied to determine their influence on the outcome. It is shown that shown that sufficiently fast rotating cores collapse due to the fall-back of matter surrounding the compact remnant and due to neutrino cooling, eventually forming spinning BHs.

### Formation of rotating neutron stars

Rotating neutron stars can be formed following the collapse of a massive star to a neutron star. Moreover, rapidly differentially rotating neutron stars are a natural outcome of the merger of a binary neutron star system which is not sufficiently massive for the merger remnant to promptly collapse to a black hole.

#### Stellar collapse to a rotating neutron star

First attempts to study the formation of rotating neutron stars in axisymmetric collapse were initiated by Evans (1984, 1986). Dimmelmeier et al. (2001), Dimmelmeier (2001) have performed general relativistic simulations of neutron star formation in rotating collapse. In the numerical scheme, HRSC methods are employed for the hydrodynamical evolution, while for the spacetime evolution the *conformal flatness approximation* (Wilson and Mathews 1995) is used. Surprisingly, the gravitational waves obtained during the neutron star formation in rotating core collapse are weaker in general relativity than in Newtonian simulations. The reason for this result is that relativistic rotating cores bounce at larger central densities than in the Newtonian limit (for the same initial conditions). The gravitational waves are computed from the time derivatives of the quadrupole moment, which involves the volume integration of $\rho r^4$. As the density profile of the formed neutron star is more centrally condensed than in the Newtonian case, the corresponding gravitational waves turn out to be weaker. Details of the numerical methods and of the gravitational wave extraction used in the above studies can be found in Dimmelmeier et al. (2002a, b). In addition, the rotational core collapse to proto-neutron star simulations performed in Dimmelmeier et al. (2002b) suggest that types of rotational supernova core collapse and gravitational waveforms identified in earlier Newtonian simulations (Zwerger and Müller 1997) (regular collapse, multiple bounce collapse, and rapid collapse) are also present in conformal gravity.

Fully relativistic axisymmetric simulations with coupled hydrodynamical and spacetime evolution in the light-cone approach, have been obtained by Siebel et al. (2002, 2003). One of the advantages of the light-cone approach is that gravitational waves can be extracted accurately at null infinity, without spurious contamination by boundary conditions. The code by Siebel et al. combines the light-cone approach for the spacetime evolution with HRSC methods for the hydrodynamical evolution. In Siebel et al. (2003) it is found that gravitational waves are extracted more accurately using the Bondi news function than by a quadrupole formula on the null cone.

Shibata (2003a) presents an axisymmetric hydrodynamics code based on HRSC methods and considers rotating stellar core collapse of a realistic, uniformly rotating, equilibrium star near the mass shedding limit with central density $\sim 10^{10}\mathrm { g\ cm}^{-3}$, and $\varGamma =4/3$ yielding a mass $M=1.491\,M_{\odot }$ and radius $R=1910\,\mathrm {km}$. The evolution adopts a $\varGamma$-law-type equation of state consisting of cold component and a thermal component (allowing for shock heating). The cold part is piecewise polytropic with exponents $\varGamma _1$ (for rest-mass densities $\rho _0\le \rho _{nuc}=2\times 10^{14}\,\mathrm {g\ cm}^{-3}$) and $\varGamma _2$ for ($\rho _0 > \rho _{nuc}$). The collapse is triggered by a small reduction of $\varGamma _1$ from the 4 / 3 value, i.e., $\varGamma _1 = 1.325$. Due to the absence of centrifugal force, after the shock formation, shock fronts of *prolate* shape spread outward. As the collapse proceeds, the central density monotonically increases until it exceeds $\rho _\mathrm{nuc}$. When the central density is $\sim 3.5\rho _{nuc}$, the collapse is halted and a proto neutron star is formed, which demonstrates approximate quasi-periodic oscillations. In a follow-up paper, Shibata and Sekiguchi (2004) perform hydrodynamic simulations in full GR of neutron star formation from stellar collapse adopting similar methods as in Shibata (2003a) and adopting the same parametric equation of state as in Dimmelmeier et al. (2002b), but focusing on the gravitational wave signatures of such events. As in Dimmelmeier et al. (2002b) gravitational waves are computed based on a quadrupole formula and it is found that waveforms computed based on their fully general relativistic simulations are only qualitatively in good agreement with the ones in Dimmelmeier et al. (2002b) which were computed based on the conformal flatness approximation. Quantitatively, the quadrupole formula used in the conformal flatness calculations (Dimmelmeier et al. 2002b), yields different results and Shibata and Sekiguchi suggest the use of a quadrupole formula which is calibrated based on fully general relativistic calculations.

Cerdá-Durán et al. (2005) introduce a new formalism based on the conformal flatness approximation that extends the original formulation (Wilson and Mathews 1995) by adding to the conformally flat 3-metric, second-order post-Newtonian terms that lead to deviations from isotropy. This new approximation is termed by the authors the CFC+ formulation and a numerical implementation is described. After testing the code using oscillating stars, the authors find that the resulting oscillation frequencies using the CFC+ formalism are practically the same as those using the original conformal flatness formalism. The authors conclude that even for stars near the mass-shedding limit the CFC+ formalism accounts for corrections at the level of 1%. The first application of the code is axisymmetric rotational core collapse to a proto-neutron star. It is shown that the gravitational waves extracted using the quadrupole formula are not substantially different between CFC+ and the original conformal flatness approach.

Obergaulinger et al. (2006) perform axisymmetric, magnetohydrodynamic simulations of magnetorotational core collapse accounting for relativistic effects with a modified TOV potential. The initial data are Newtonian, equilibrium (differentially) rotating polytropes which are seeded with a dynamically weak, dipolar magnetic field (central field strength of $10^{10}-10^{13}\,\mathrm {G}$). The evolution adopts HRSC schemes for the magnetohydrodynamic equations and the constrained transport method of Evans and Hawley (1988) for the $\mathbf{\nabla \cdot B}=0$ constraint, and a hybrid $\varGamma$-law equation of state as in Dimmelmeier et al. (2002b). The main effects of magnetic fields are to trigger the MRI, and brake the differential rotation of the initial star. It is found that both of these effects operate in their simulations and that the saturated magnetic field reaches magnitudes of order $10^{16}$. However, it is not reported whether a proto-magnetar forms in these simulations. It is generally found that only stronger initial magnetic fields can affect the gravitational wave signatures significantly, and that the gravitational waves should be detectable by aLIGO, if the source is about 10 kpc away.

The gravitational-wave signal from rotating core collapse has been investigated via hydrodynamic evolutions in 2 and 3 spatial dimensions, both in Newtonian gravity (Müller and Hillebrandt 1981; Müller 1982; Mönchmeyer et al. 1991; Zwerger and Müller 1997; Kotake et al. 2003; Ott et al. 2004) and in general relativity (Dimmelmeier et al. 2002a, b, 2008; Shibata and Sekiguchi 2004; Obergaulinger et al. 2006; Ott et al. 2007) and four different types of gravitational wave signals have been identified so far (see also Ott 2009 for a comprehensive review on core collapse supernovae):

I. In this type the stellar core bounces due to the stiffening of the equation of state at nuclear densities and subsequently rings down into equilibrium. The gravitational wave train possesses one large peak corresponding to core bounce, and then undergoes a damped ring-down phase.
II. In this type the stellar core bounce is driven by centrifugal forces occurring at sub-nuclear densities and unlike type I the post bounce phase consists of multiple bounces that are gradually damped. As a result the gravitational waveform is characterized by multiple distinct peaks corresponding to each bounce.
III. In this type the stellar core undergoes rapid collapse following bounce. The gravitational waveforms are low-amplitude and possess a subdominant peak.
IV. For magnetized progenitors with $B\gtrsim 10^{12}\,\mathrm {G}$, the magnetic fields can affect the bounce dynamics. The gravitational wave signature, which has been referred to as magnetic-type, initially resembles the multiple-bounce signal. However, after the first shock launching the gravitational wave signal shows high-amplitude oscillations whose frequencies increase as the collapse proceeds.

More detailed studies in Ott et al. (2007), Dimmelmeier et al. (2008) that account for both general relativistic and microphysics effects suggest that the generic core-collapse gravitation signal is of type I. Some work on understanding the different oscillation modes of proto-neutron stars was performed by Fuller et al. (2015), where the effects of relativity were largely ignored.

Recently, magnetorotational core collapse has been studied by Mösta et al. (2014) via ideal magnetohydrodynamic simulations in full GR that accounts for microphysics by adopting a finite-temperature nuclear equation of state and a neutrino leakage scheme. The simulations are performed using the Einstein Toolkit and the focus is on outflows and magnetic instabilities, rather than the gravitational wave signal. Fundamental differences are reported between axisymmetric and full 3D simulations in which a kink develops breaking the axisymmetry of the expanding lobes.

In a more recent work, Andresen et al. (2017) perform 3D multi-group neutrino hydrodynamic simulations of core-collapse supernovae focusing on the gravitational wave signatures generated during the first few hundreds of milliseconds from the post-bounce phase. Approximate general relativistic effects are accounted for by use of a pseudorelativistic effective potential. The authors find that gravitational waves from models dominated by the standing-accretion-shock instability (SASI) are clearly distinct from models that are convection-dominated. The main difference arises in the low-frequency band around 100–200 Hz. The authors also find that the gravitational wave strain above 250 Hz in 3D is considerably lower than in 2D simulations. The authors’ results suggest that second-generation detectors will be able to detect only very nearby events, but that third-generation detectors could distinguish SASI- and convection-dominated models at distances of 10 kpc.

#### Binary neutron star mergers

Binary neutron stars have been simulated using numerical relativity techniques for over a decade. There are recent technical reviews of the topic focusing primarily on the history of relevant studies, numerical techniques and the final fate of the merger remnant, see e.g., Duez (2010) and Faber and Rasio (2012), Baiotti and Rezzolla (2017), Paschalidis (2017). Here, we focus on the remnant NS properties highlighting the most recent results related to the remnant hypermassive neutron star (HMNS) oscillations, and how these can help to constrain the nuclear equation of state.

Observational determination of masses in the known binary neutron star (NSNS) systems (Chamel et al. 2013; Miller and Miller 2015; Özel and Freire 2016; Miller and Lamb 2016; Oertel et al. 2017) indicates that a likely range of the total binary mass (sum of individual TOV masses) is $2.4\,M_{\odot }< M_\mathrm{tot} < 3.0\,M_{\odot }$ with a peak around $2.7\,M_{\odot }$. Since observations (Demorest et al. 2010; Antoniadis et al. 2013) require a TOV limit mass of $\gtrsim 2.0\,M_{\odot }$, a likely outcome of a NSNS merger is a long-lived ($\gtrsim 10\,\mathrm {ms}$) HMNS (see, e.g., Hotokezaka et al. 2011; Bauswein et al. 2012).

The rotational profile of hypermassive neutron stars formed following binary neutron star mergers have been studied by a number of authors (Shibata and Uryū 2000; Shibata and Taniguchi 2006; Baiotti et al. 2008; Anderson et al. 2008b; Liu et al. 2008; Bernuzzi et al. 2012; Kastaun and Galeazzi 2015; De Pietri et al. 2016; Kastaun et al. 2016). The common outcome in these studies is that the actual differential rotation profile does not seem to match the *j*-constant rotation law that is usually adopted in models of isolated differentially rotating neutron stars. Instead, the post-merger remnants almost universally exhibit a rotation profile that is approximately uniform in the core that smoothly turns into quasi-Keplerian in the outer layers. An extended study in Hanauske et al. (2017) argues that this profile seems to also be EOS-independent.

**Fig. 33:**
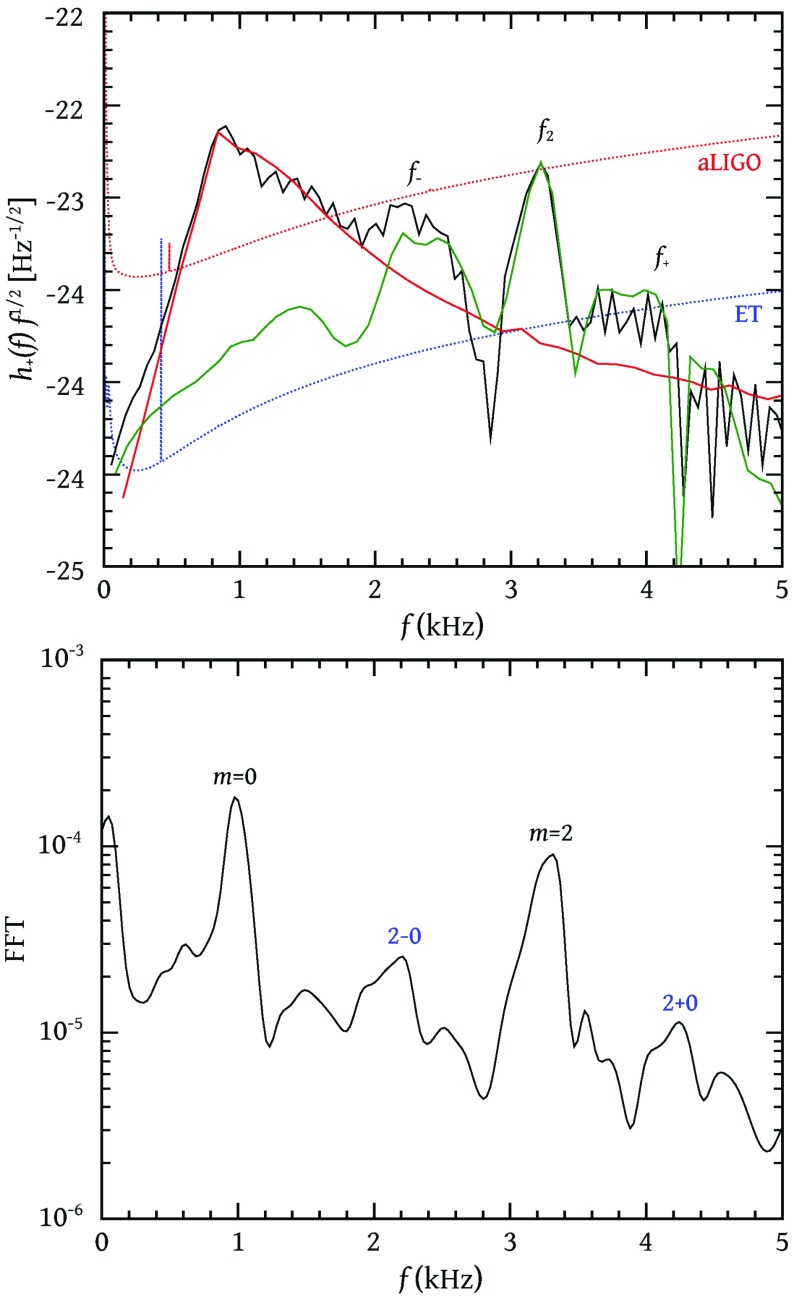
*Top panel*: Total (black), pre-merger (red) and post-merger (green) scaled power spectral density, compared to the aLIGO and ET unity SNR sensitivity curves for a HMNS formed in the merger of an equal mass NSNS system using the Lattimer–Swesty (LS). Each neutron star has a mass of $1.35\,M_{\odot }$ and the distance to the source is set at a nominal value of 100 Mpc. *Lower panel*: Corresponding FFT of the evolution of pressure in the equatorial plane, where discrete oscillation frequencies and their quasi-linear combinations can be seen. (Image reproduced with permission from Stergioulas et al. 2011, copyright by the authors)

The gravitational-wave spectrum in the post-merger phase comprises several distinct peaks that could be used for characterizing the hypermassive compact object (see, e.g., Zhuge et al. 1994; Oechslin et al. 2002; Shibata and Uryū 2002; Shibata et al. 2005; Shibata and Taniguchi 2006; Kiuchi et al. 2009; Dietrich et al. 2017). That several post-merger GW peaks do in fact originate from specific oscillation modes of the remnants was established in Stergioulas et al. (2011), by extracting eigenfunctions in the equatorial plane for the dominant oscillation frequencies. Gravitational wave spectra were split into pre- and post-merger parts and it was shown that several peaks in the post-merger GW spectrum have discrete counterparts in the evolution of the fluid that correspond to specific normal modes of oscillation. The dominant peak was identified as being the co-rotating $m=2$ *f*-mode (denoted as $f_2$ or $f_\mathrm{peak}$), while additional frequencies ($f_{-}$ and $f_{+}$ ) were shown to originate from the quasi-linear combination between $f_2$ and the quasi-radial oscillation frequency $f_0$. The quasi-radial frequencies satisfy $f_- =f_2 - f_0$ and $f_+ =f_2 + f_0$, forming an equidistant triplet with $f_2$ (see Fig. 33). Since the amplitude of $f_+$ is much smaller than other frequency peaks, the quasi-linear combination frequency $f_-$ is the more important one from the observational point of view (after $f_2$) and it has been renamed to $f_{2-0}$ in subsequent studies, in order to emphasize its origin. Since $f_{2-0}$ is a quasi-linear feature, its amplitude quickly decays (it is the product of the amplitudes of $f_2$ and $f_0$).

A more extensive parameter search by Bauswein and Stergioulas (2015) revealed that apart from the $f_{2-0}$ quasi-linear peak, a fully nonlinear peak (denoted as $f_\mathrm{spiral}$) exists in most cases, originating from the transient appearance of a spiral deformation with two antipodal bulges at the time of merging. Investigating a large number of EOSs and different masses, Bauswein and Stergioulas (2015) found that the post-merger phase can be broadly classified as belonging to one of three different types:

1. *Type* I (soft EOS/high mass): $f_{2-0}$ is the strongest secondary peak.
2. *Type* II (intermediate EOS/intermediate mass): $f_{2-0}$ and $f_\mathrm{spiral}$ have roughly comparable amplitudes.
3. *Type* III (stiff EOS/low mass): $f_\mathrm{spiral}$ is the strongest secondary peak.

**Fig. 34:**
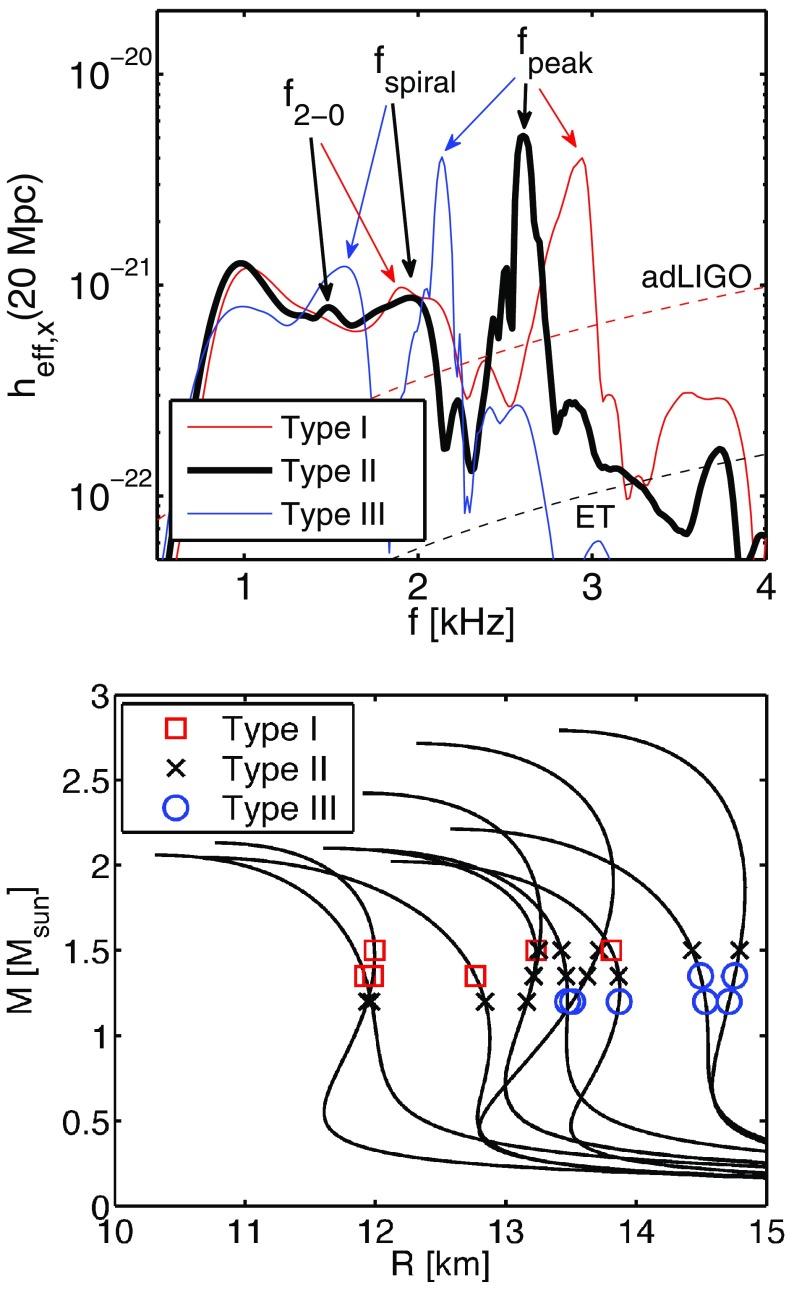
*Top panel*: GW spectra of 1.35–$1.35\,M_{\odot }$ mergers with a soft (red), intermediate (black) and stiff EOS (blue), at a reference distance of 20 Mpc. *Lower panel*: Different types of merger dynamics are indicated for several EOSs and different masses (in each case, half of the sum of individual pre-merger masses is shown). (Image reproduced with permission from Bauswein and Stergioulas 2015, copyright by APS)

For a broad sample of EOSs and for initial masses of $2.4\,M_{\odot }\le M_\mathrm{tot} \le 3.0\,M_{\odot }$, the frequency of $f_\mathrm{spiral}$ is in the range $f_\mathrm{peak} - 0.5\,\mathrm {kHz}< f_\mathrm{spiral}< f_\mathrm{peak} - 0.9\,\mathrm {kHz}$, while $f_{2-0}$ is in the range $f_\mathrm{peak} - 0.9\,\mathrm {kHz}< f_\mathrm{2-0}< f_\mathrm{peak} - 1.3\,\mathrm {kHz}$. The fact that the two ranges do not overlap can be used in search strategies and in identifying the type of the merger dynamics. Fig. 34 (left panel) displays GW spectra for three representative cases (corresponding to the three types described above), while the right panel shows the dependence of the different types on the initial mass ($M_\mathrm{tot}/2$ is shown) in a mass vs. radius plot for nonrotating models.

**Fig. 35:**
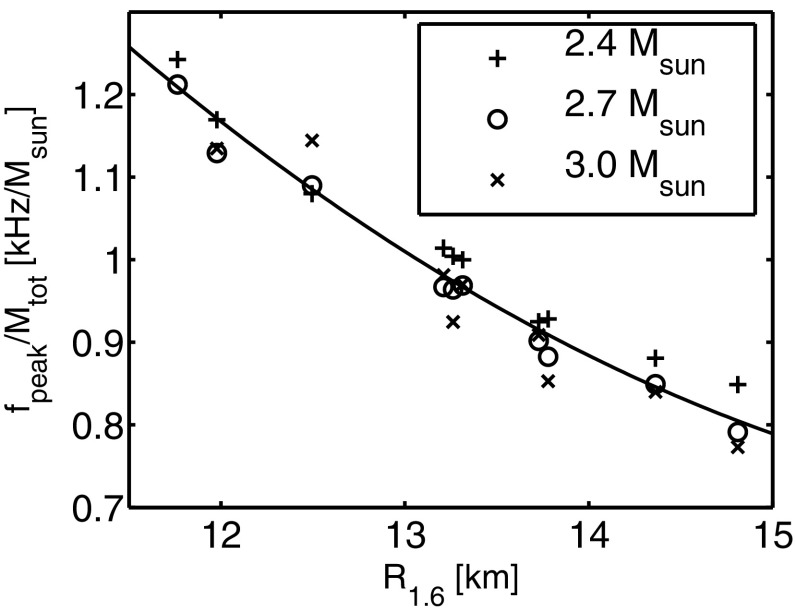
Peak frequency $f_\mathrm{peak}$ scaled by the total mass $M_\mathrm{tot}$ versus the radius of a nonrotating NS of mass $1.6\,M_{\odot }$ for different EOSs. The symbols correspond to different values of the total mass. The solid line shows the quadratic fit in Eq. (143), which can be used to determine $R_{1.6}$ with a maximum uncertainty of a few percent for a given $f_\mathrm{peak}$ measurement in a system where the total mass $M_\mathrm{tot}$ is determined from the inspiral gravitational waveform. (Image reproduced with permission from Bauswein et al. 2016, copyright by SIF/Springer)

Bauswein and Janka (2012) found that the peak frequency $f_\mathrm{peak}$ is directly related to the radius of nonrotating neutron stars through an EOS-independent empirical relation, which can be used to observationally determine neutron star radii with high accuracy, when the total mass of the system is known. Because the remnants for mergers in the $2.4\,M_{\odot }\le M_\mathrm{tot} \le 3.0\,M_{\odot }$ have a central density comparable to that of a $\sim 1.6\,M_{\odot }$ nonrotating neutron star, the uncertainty in the above empirical relation is reduced when it is cast in terms of the radius $R_{1.6}$ of a $\sim 1.6\,M_{\odot }$ nonrotating star (Bauswein et al. 2012). In Bauswein and Janka (2012) representative examples of three initial binary setups (focusing on the $1.35+1.35\,M_{\odot }$ case) were discussed. Relations for different binary masses and mass ratios (using also a larger set of EOSs) were discussed and presented in Bauswein et al. (2012). For specific binary masses such an empirical relation can have an uncertainty of only a few percent. In the case of a $1.35+1.35\,M_{\odot }$ merger, the relation yielding the radius of a $\sim 1.6\,M_{\odot }$ nonrotating star is (Bauswein et al. 2014)

142 $$\begin{aligned} R_{1.6}=1.099\cdot f_\mathrm{peak}^2-8.574\cdot f_\mathrm{peak}+28.07. \end{aligned}$$

The $f_\mathrm{peak }$ versus radius relation can be scaled by the total mass, to become a universal relation, which is (to high accuracy) quadratic in the radius:

143 $$\begin{aligned} f_{\mathrm {peak}}[\mathrm {kHz}]/M_\mathrm{tot}[M_\odot ]= 0.0157\cdot R_{1.6}^2-0.5495\cdot R_{1.6} +5.5030, \end{aligned}$$

see Bauswein et al. (2016) and Fig. 35. This relation depends only weakly on the mass ratio. Similar relations can easily be constructed for the radius of nonrotating stars at lower or higher masses than $1.6\,M_{\odot }$, but then the accuracy of radius determinations deteriorates for $1.35\,M_{\odot }+1.35\,M_{\odot }$ mergers. For other total binary masses, other TOV radii are obtained with minimal uncertainty (Bauswein et al. 2012).

A single event in the most likely range of $2.4\,M_{\odot }\le M_\mathrm{tot} \le 3.0\,M_{\odot }$ will thus suffice to significantly constrain the EOS in the density range that corresponds to a TOV mass of $1.6 \,M_{\odot }$. At significantly higher densities (close to $M_{\max }>2\,M_{\odot }$), it is unlikely that direct constraints can be obtained. On the one hand, the expected merger rate may diminish above $M_\mathrm{tot} > 3.0\,M_{\odot }$, since all known double neutron star systems have masses smaller than this (notice that measuring neutron star radii from inspiral waveforms is similarly restricted to low masses). On the other hand, even in the rare case of a merger with an unusually high total mass it is quite possible that the remnant will promptly collapse to a black hole, before the radius can be measured through the detection of post-merger gravitational waves. However, Bauswein et al. (2014) devised a method to extrapolate the mass and radius of the maximum-mass TOV model from at least two well-separated low-mass $f_\mathrm{peak}$ measurements. The method is based on the observation that for a given EOS $f_\mathrm{peak}$ is almost a linear function of $M_\mathrm{tot}$, while the slope of this relation can be used to determine empirically the threshold mass of binary systems to black hole collapse, $M_\mathrm{thres}$. From an empirical relation between $M_\mathrm{thres}$ and the maximum TOV mass $M_{\max }$, found in Bauswein et al. (2013), one thus arrives at a determination of $M_{\max }$ with an uncertainty of order $0.1\,M_{\odot }$. Furthermore, an empirical relation between the peak frequency $f_\mathrm{peak}^\mathrm{thres}$ for a binary system with mass equal to the threshold mass $M_\mathrm{thres}$ and the radius $R_{\max }$ of the maximum-mass TOV model then permits a determination of $R_{\max }$ with an uncertainty of order 5%. Similar considerations allow the determination of the central density of the maximum-mass TOV star, $\rho _{c,{\max }}$ with an uncertainty of order 10%.

**Fig. 36:**
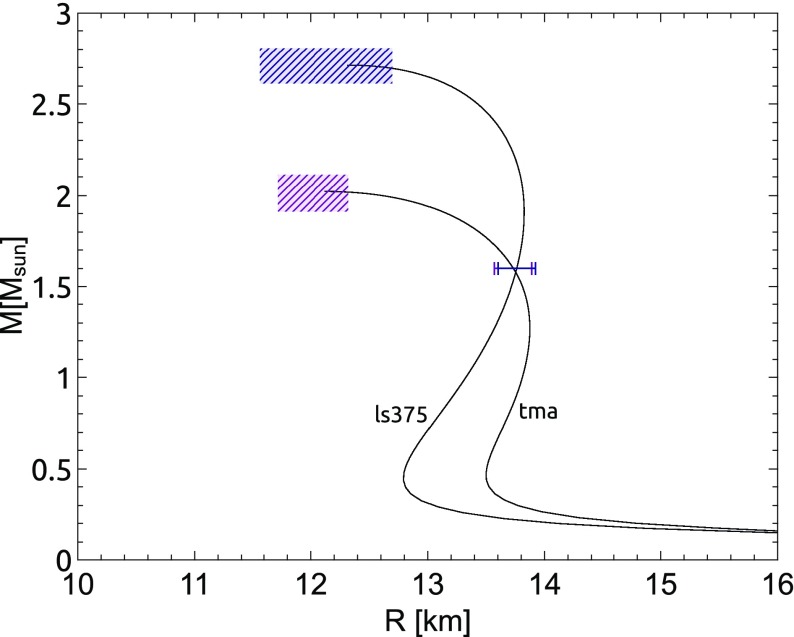
Mass-radius relations for two EOSs with similar stellar properties in the intermediate mass range around $1.6\,M_{\odot }$ where the two mass-radius relations cross. Bars at $1.6\,M_{\odot }$ indicate the maximum deviation of the estimated radius inferred from a single GW detection of a low-mass binary NS merger. Using the extrapolation procedure described in Bauswein et al. (2014) the two EOSs can clearly be distinguished. Boxes illustrate the maximum deviation of the estimated properties of the maximum-mass configuration. (Image reproduced with permission from Bauswein et al. 2014, copyright by APS)

Two representative cases of the determination of the radius and mass $(R_{\max },M_{\max }$) of the maximum-mass TOV model are shown in Fig. 36. These two EOSs cross at about $1.6\,M_{\odot }$, so that they cannot be distinguished by a single low-mass merger event. However, extrapolating two well-separated low-mass $f_\mathrm{peak}$ measurements (using the procedure described in Bauswein et al. 2014) allows for a clear distinction of the EOS.

Lehner et al. (2016) study hypermassive neutron stars formed in equal and unequal-mass NSNS mergers with realistic, hot nuclear equations of state while employing an approximate neutrino cooling scheme. The authors find agreement with earlier findings in Bauswein and Stergioulas (2015), as well as that for a given total mass, the mass ratio has only a small effect on $f_\mathrm{peak}$ (Bauswein et al. 2012). They also discuss an interesting empirical relation between $f_\mathrm{peak}$ and the GW frequency at contact. A different correlation between the post-merger oscillation frequency and the tidal coupling constant $\kappa _2^T$ has been discussed by Bernuzzi et al. (2015). The authors report that their proposed correlation exhibits small scatter with the binary total mass, mass-ratio, EOS, and thermal effects. However, only total masses in the range of 2.5–$2.7\,M_{\odot }$ where considered and thermal effects where not based on realistic finite temperature EOSs.

The above results suggest that a postmerger gravitational wave detection can potentially determine neutron star radii to high accuracy and thus constrain the EOS. The model can be further refined by taking into account additional effects. For example, although preliminary MHD studies suggest that realistic magnetic fields do not have a significant direct impact on the $f_\mathrm{peak}$ frequency (see, e.g., Endrizzi et al. 2016; Kawamura et al. 2016), the timescale on which MRI could modify the background requires further studies.

Takami et al. (2014) perform binary neutron star merger simulations for different EOSs (which are fitted by piecewise polytropes) using the Whisky code, and suggest a universal (EOS and mass-independent) empirical relation between a secondary peak in the GW spectrum and the compactness *M* / *R* of the progenitor neutron stars, although their analysis was for a restricted set of EOSs and for varying mass ranges, without distinguishing between $f_{2-0}$ and $f_\mathrm{spiral}$. In Rezzolla and Takami (2016), a somewhat more extensive set of models is considered. While their GW spectra appear to be broadly consistent with the unified picture presented in Bauswein and Stergioulas (2015), a different interpretation of the secondary peaks (not consistent with Bauswein and Stergioulas 2015) is presented.

Maione et al. (2017) also perform a large number of binary neutron star merger simulations (using the Einstein Toolkit, Löffler et al. 2012) surveying the effects of total mass, the EOS stiffness and the mass ratio. They test their results against the two competing interpretations of the sub-dominant frequencies in the post-merger spectrum that have been presented in Bauswein and Stergioulas (2015) and Takami et al. (2014). The authors conclude that they agree with Bauswein and Stergioulas (2015) (which includes two different mechanisms for producing *mass-dependent* sub-dominant frequencies, $f_{2-0}$ and $f_\mathrm{spiral}$) in that at least two different mechanisms should be considered for the interpretation of these sub-dominant frequencies. In several models, Maione et al. were able to confirm another prediction of the unified model of Bauswein and Stergioulas (2015), that the presence of $f_\mathrm{spiral}$ would leave an observable imprint also in the maximum density evolution, as a modulation with frequency $f_\mathrm{peak}-f_\mathrm{spiral}$, due to the relative instantaneous orientation of the external spiral structure with respect to the internal double core structure.

Shibata and Kiuchi (2017) perform viscous hydrodynamic simulations of binary neutron star mergers in full GR and argue that for large values of the shear viscosity any post-merger oscillations could be damped within 5 ms. Nevertheless, the values of the shear viscosity the authors adopted may be large compared to realistic values anticipated due to magnetic fields. More recently, Alford et al. (2017) explored various dissipative mechanisms that may operate in a binary neutron star merger remnant and argue that bulk viscosity may be sufficiently strong to dampen any post-merger oscillations. A careful investigation of the effects of bulk viscosity in future relativistic calculations of mergers will show whether bulk viscosisty plays an important role. For another review summarizing binary neutron star post-merger oscillation properties see Baiotti and Rezzolla (2017). Properties of the post-merger remnants have also been investigated in Kaplan et al. (2014), Kastaun and Galeazzi (2015), Kastaun et al. (2016).

The detectability of such post-merger oscillations has recently also been considered. Clark et al. (2016) developed a post-merger GW detection template, based on the method of principal component analysis (PCA) and evaluated the prospects for detectability when using present and planned gravitational wave interferometers. Adopting a signal-to-noise (*SNR*) ratio detection threshold of 5, an optimally oriented source and the galactic merger rate of Abadie et al. (2010) they calculated that post-merger oscillations would not be detectable by advanced LIGO at design sensitivity, but could become detectable out to $\sim 110$–$180\,\mathrm {Mpc}$ (depending on the EOS) with the proposed LIGO Voyager upgrade (LV) (LIGO Scientific Collaboration 2015), out to $\sim 200$–$340\,\mathrm {Mpc}$ with the planned third-generation Einstein Telescope (ET) (Amaro-Seoane et al. 2009) and out to $\sim 330$–$530\,\mathrm {Mpc}$ with the planned Cosmic Explorer (CE) (Abbott et al. 2017) detector. These results translate to EOS-dependent detection rates of $\sim 0.2$–$0.9\,\mathrm {year}^{-1}$ for LV, $\sim 1$–$6\,\mathrm {year}^{-1}$ for ET and $\sim 5$–$23\,\mathrm {year}^{-1}$ for CE.

Yang et al. (2017) focus on the detectability of the dominant component of the $l=2,m=2$ mode, exploring different EOSs and considering the ET and CE detectors. They adopt the same SNR detection threshold of 5 as Clark et al. (2016). Instead of the galactic merger rate of Abadie et al. (2010) adopted by Clark et al.  they adopt the galactic merger rate of Belczynski et al. (2016), Dominik et al. (2015), Mink and Belczynski (2015), which is consistent with the latest pulsar beaming corrections and improved modeling of PSR J0737-3039B (Kim et al. 2015), and roughly 10 times lower than the one adopted by Clark et al. In addition, Yang et al. consider sources that are not optimally oriented, and instead have random sky orientations. Moreover, they adopt the Gaussian mass-distribution for binary neutron stars of Özel and Freire (2016) and reject models with total mass larger than the threshold mass for prompt collapse. Under the above assumptions, Yang et al. perform 100 Monte-Carlo (MC) realizations assuming a 1-year of observations, and (for the EOSs treated) they find it is not likely that a detection of the dominant component of the post-merger $l=2,m=2$ mode from individual sources will be made. However, this conclusion depends on the EOS. For example, for the TM1 EOS the rate of individual detections exceeds $1\,\mathrm {year}^{-1}$ only in 15% of the MC realizations, but rises to $\sim 60\%$ of MC realizations for the Shen EOS.

To increase the prospects for detection, Yang et al. (2017) develop a method that takes advantage of the empirical relation between the peak frequency (when scaled by the total binary mass) and the radius of a nonrotating neutron star of mass $1.6\,M_{\odot }$ (as given in Bauswein et al. 2016; see Eq. (143)) to coherently stack an ensemble of post-merger signals. The authors find that after coherently stacking the post-merger oscillations from different sources, the percentage of MC realizations with stacked SNR above the detection threshold rises considerably to 91% for the TM1 EOS and CE. In agreement with Clark et al. (2016), Yang et al. find that systematic errors (e.g., scatter in the $f_\mathrm{peak}$-EOS universal relationship) at this time dominate the statistical errors in inferring the NS radius and hence constrain the EOS. Another work exploring stacking of post-merger signals was recently presented by Bose et al. (2017) (but without considering the detection probability). As pointed in Yang et al. (2017) the results from all of these studies should be considered preliminary, because they depend on the mass distribution and the merger rates of binary neutron stars as well as imprecise models of the post-merger gravitational waveforms which should all improve with time.

#### One-arm instability

Apart from the dynamical bar-mode ($m=2$), highly differentially rotating stars can also become unstable to a dynamical one-arm ($m=1$) “spiral” instability. Such, highly differentially rotating neutron stars can form either during core-collapse or binary neutron star mergers. Hence, one might expect that the one-arm instability could arise in these dynamical scenarios. Indeed, the instability has been found to operate in the differentially rotating neutron star cores formed in general relativistic hydrodynamic core-collapse simulations by Ott et al. (2005, 2007) and Kuroda et al. (2014). Shibata and Sekiguchi (2005b) also report the emergence of $m=1$ modes in core-collapse simulations, but the $m=1$ perturbations are not reported to grow significantly. However, until recently the $m=1$ instability has never been found to operate in hypermassive neutron stars formed in a binary neutron star merger. Nevertheless, we note that Anderson et al. (2008a) performed magnetized neutron star mergers in full general relativity and reported the emergence of $m=1$ modes following merger, which were attributed to magnetic Tayler instabilities (Tayler 1973a, b; Markey and Tayler 1973). In addition, $m=1$ density modes in hypermassive neutron stars formed following binary neutron star mergers were reported by Bernuzzi et al. (2014), where they were explained to arise due to mode couplings.

**Fig. 37:**
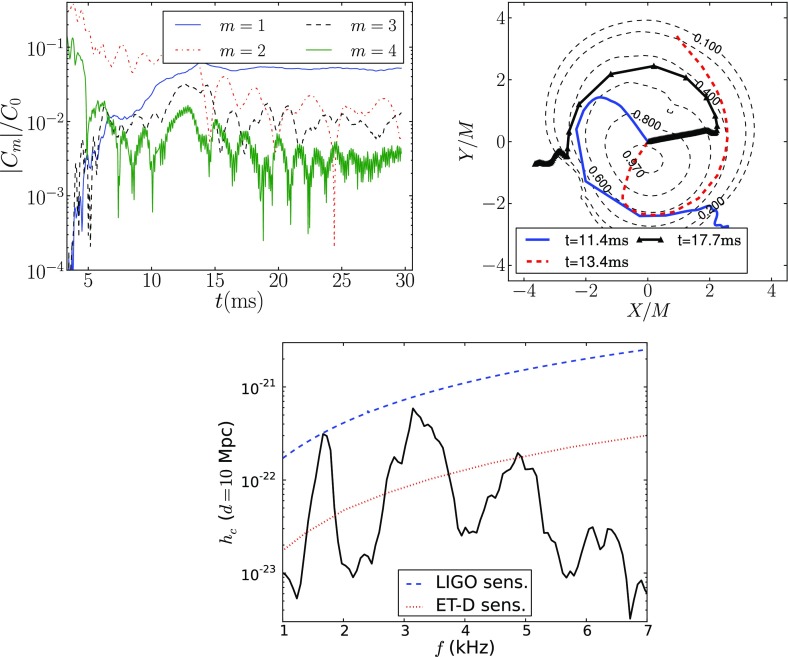
Top left panel: Magnitude of $C_m$ normalized to $C_0$ as a function of coordinate time. Top right panel: Thick lines indicate the phase of the mode $C_1$ as a function of cylindrical radius $\varpi$ at select times. Dashed thin lines are contours of the rest-mass density normalized to its maximum value at $t=13.4$ ms. The numbers inlined are the values of the level surfaces. The small contour at $X/M\approx Y/M \approx 1$ has a value of 0.6 and is at the cite of a strong vortex. Lower panel: gravitational wave characteristic strain (solid black curve) vs gravitational wave frequency for the last $\sim 15\,\mathrm {ms}$ (after the $m=1$ instability has saturated) seen by an observer located edge-on at $r=10$ Mpc. The first peak on the left corresponds to the $m=1$ mode. Notice that the frequencies of the peaks satisfy $f_m\simeq m f_1, \quad m=1,\ldots 4$, with $f_1 \simeq 1.7$ kHz. Dashed (blue) curve: the aLIGO sensitivity curve. Dotted red curve: the proposed Einstein Telescope (ET-D) sensitivity curve (Hild et al. 2011) (Image reproduced with permission from Paschalidis et al. 2015, copyright by APS)

But, recent hydrodynamic simulations in full general relativity adopting a piecewise polytropic equation of state of moderate stiffness by Paschalidis et al. (2015), report the development of the one-arm instability in the highly differentially rotating hypermassive neutron star remnant for the first time. The emergence of the instability in the merger remnants of eccentric binary neutron star mergers was subsequently studied in East et al. (2016b) with a larger survey of hydrodynamic simulations in full general relativity. Paschalidis et al. and East et al. argued that the trigger of the instability is the post-merger vortex dynamics during the merger of the two stars. The growth time of the instability in the cases studied was $\sim 1$–$2\,\mathrm {ms}$ and the instability saturates within $\sim 10$–$20\,\mathrm {ms}$ from merger. The $m=1$ instability is most easily observed in an azimuthal density decomposition through the quantities

144 $$\begin{aligned} C_m(\varpi ,z) = \frac{1}{2\pi }\int _0^{2\pi }\rho _0 u^0\sqrt{-g}e^{im\phi } \, d\phi \end{aligned}$$

and

145 $$\begin{aligned} C_m = \frac{1}{2\pi }\int _0^{2\pi }\rho _0 u^0\sqrt{-g}e^{im\phi } \, d^3x, \end{aligned}$$

where *g* is the determinant of the metric, $\rho _0$ the rest-mass density, $u^\mu$ the fluid 4-velocity, and $\phi$ an azimuthal angle defined in a center-of-mass frame. Here, $\varpi$ is the cylindrical radius from the center of mass. These quantities are shown in the left and middle panels of Fig. 37 for a binary neutron star merger remnant that underwent the one-arm instability in Paschalidis et al. (2015), where it is clear (left panel) that $\sim 10\,\mathrm {ms}$ following merger the $m=1$ density azimuthal mode amplitude is larger than all other non-zero *m* modes, signaling the saturation and dominance of the instability. Notice the almost constant amplitude of the $m=1$ mode throughout the evolution, which acts as a quasistationary source of gravitational waves. The middle panel also plots the rest-mass density contours and the phase of the $C_1$ mode in the center of mass and on the equatorial plane at select times. Notice how the high-density hypermassive neutron star core is displaced from the center of mass—a signature of the $m=1$ instability. Observe also the spiral pattern of the phase of the $m=1$ mode as it becomes sheared toward the surface of the remnant (although it is unclear at the moment whether the spiralling of the phase of the mode has any physical significance).

Paschalidis et al. also investigated whether previous criteria for the development of the instability hold in these cases, too. They find that there exists a corotation radius within the star prior to the development of the instability. This extends earlier criteria for the development of shear instabilities from isolated cold stars to hot hypermassive neutron stars formed by binary neutron star mergers.

Both Paschalidis et al. (2015) and East et al. (2016b) demonstrated that the instability is imprinted on the gravitational waves generated during the post-merger phase. In particular, the m $=$ 1 instability gives rise to an $l=2,m=1$ mode of gravitational waves that is quasi-periodic and almost constant in amplitude, and with the gravitational wave fundamental frequencies being consistent with the dominant rotational frequencies of azimuthal density modes. Moreover, the $l=2,m=1$ gravitational wave signature occurs at roughly half the frequency of the $l=2,m=2$ mode (higher modes have frequencies $f_m\simeq m f_1$—another signature of the one-arm instability) and hence lies in a regime where the LIGO detector is more sensitive (see right panel of Fig. 37). If the $m=1$ mode persists during the hypermassive neutron star lifetime $t_\mathrm{HMNS} \sim O(1)\,\mathrm {s}$ (Paschalidis et al. 2015; East et al. 2016b), the peak power at the $m=1$ mode frequency can be amplified by a factor $\sqrt{t_\mathrm{HMNS}/(15\,\mathrm {ms})}\sim O(10)$. Thus, for long-lived (1–2 s) hypermassive neutron stars for which the one-arm instability persists Paschalidis et al. and East et al. predicted that the GWs could be detectable by aLIGO at $\sim 10$ Mpc and by the Einstein Telescope at $\sim 100\,\mathrm {Mpc}$, and speculated that the GWs from the instability may help to constrain the EOS of the matter above nuclear saturation density.

While these simulations had high eccentricity at merger, Paschalidis et al. and East et al. found that the instability arises for cases where the total angular momentum at merger $J/M^2 \sim 0.9$–1.0, where *J* is the ADM angular momentum and *M* the ADM mass. Since this part of the parameter space is also relevant for quasicircular mergers, Paschalidis et al. and East et al. predicted that the $m=1$ instability should arise in quasicircular binary neutron star mergers, too. These predictions were subsequently confirmed by Radice et al. (2016) and Lehner et al. (2016) who performed hydrodynamic simulations in full GR for equal-mass, and equal- and unequal-mass binary neutron star mergers, respectively and confirmed that the one-arm instability develops in quasicircular mergers. Radice et al. employed piecewise polytropic equations of state, while Lehner et al. adopted realistic equations of state. The Radice et al. study concluded that aLIGO is unlikely to detect the $l=2,m=1$ modes arising from the one-arm instability, but, as Lehner et al. pointed out, it is worth searching for such quasimonochromatic signatures, because the gravitational wave signal from the inspiral will reduce the signal-to-noise ratio required for detection of the post-merger gravitational wave signal. Both studies found that for hypermassive neutron stars that undergo delayed collapse to black hole $\gtrsim 20\,\mathrm {ms}$ following merger, the instability develops for all equations of state they considered. Finally, Lehner et al. (2016) confirm the hypothesis of Paschalidis et al. (2015), East et al. (2016b) that the frequencies of the peaks in the gravitational wave power spectrum should correlate with the nuclear equation of state. The same conclusion was also reached in the more recent relativistic studies of East et al. (2016a), where it was shown that softer equations of state result in higher frequency $l=2,m=1$ gravitational wave modes. East et al. (2016a) find that the one-arm instability can be triggerred almost independently of the background configuration that forms following merger, i.e., independently of whether the hypermassive neutron star is toroidal, ellipsoidal or a double core configuration. They also estimate that typical signal-to-noise ratios of the $l=2,m=1$ GW modes generated by the one-arm instability would be $\sim 3$ for aLIGO at 10 Mpc and $\sim 3$ at 100 Mpc for the Einstein Telescope. These signal-to-noise estimates are more optimistic that those presented in Radice et al. (2016), but less optimistic than the ones in Lehner et al. (2016). Thus, gravitational waves from the $m=1$ instability could potentially be used to probe the nuclear equation of state, although more work is needed to solidify this idea.

### Evolution of magnetized, rotating neutron stars

Recent advances in the field of numerical relativity that combine HRSC methods with the BSSN or the Generalized harmonic formulation as well as approaches that control the no-magnetic-monopole constraint $\mathbf{\nabla }\cdot \mathbf{B} = 0$ (see, e.g., Etienne et al. 2010, 2012 for a summary of such methods), have allowed the evolution of magnetohydrodynamic models of neutron stars that enabled the study of magnetic effects such as magnetic instabilities as well as magnetically driven outflows and magnetospheric phenomena.

#### Magnetic instabilities

Axisymmetric, magnetohydrodynamic simulations of neutron stars endowed with purely toroidal magnetic fields are performed in full GR by Kiuchi et al. (2008). Both rotating and nonrotating, magnetized, equilibrium $\varGamma =2$ polytropes are evolved and the stability of such configurations is investigated varying the compaction, profile and strength of magnetic fields and degree of rotation. The equilibrium initial data are constructed as described in Kiuchi and Yoshida (2008), and the toroidal magnetic field is set such that

146 $$\begin{aligned} b_\phi =B_0 u^t(\rho h\alpha ^2\gamma _{\phi \phi })^k, \end{aligned}$$

where $B_0$ and *k* are constants that determine the *B*-field strength and profile, $u^\mu$ is the fluid four-velocity, *h* the relativistic enthalpy, $\alpha$ the lapse function and $\gamma _{ij}$ is the 3-metric. Note that Eq. (146) assumes geometrized polytropic units. As is clear from Eq. (146) the magnetic field is confined in the NS interior. For the evolution, the GR magnetohydrodynamics code described in Shibata and Sekiguchi (2005a) is adopted in conjunction with a $\varGamma$-law equation of state. It is found that for $k=1$ the stars are stable, but for $k\ge 2$ a dynamical instability sets in, which occurs on an Alfvén timescale until a new state is reached which is dynamically stable against axisymmetric perturbations. It is also found that rotation tends to stabilize the stars, and overall these results are in agreement with earlier studies by Tayler (1973a), Acheson (1978), and Pitts and Tayler (1985). In a follow-up study, Kiuchi et al. (2011) use similar methods to study the stability of toroidal magnetic fields in nonrotating and rapidly, rigidly rotating, $\varGamma =2$ polytropes in full general relativity and $3+1$ dimensions, focusing on the $k=1$ case and nonaxisymmetric perturbations. Very strong initial magnetic fields of $10^{16}$–$10^{17}\,\mathrm {G}$ are chosen, which may not be realistic but are useful to accelerate the development of instabilities. It is found that the Parker (Parker 1966, 1967) and/or Tayler instabilities operate in both nonrotating and rotating stars triggering long-term turbulence. In contrast to the axisymmetric simulations, it is found that the magnetic fields never reach a dynamically stable state after the onset of turbulence. It is concluded that unlike linearized studies even rotation cannot stabilize the $k=1$ case against nonaxisymmetric perturbations.

**Fig. 38:**
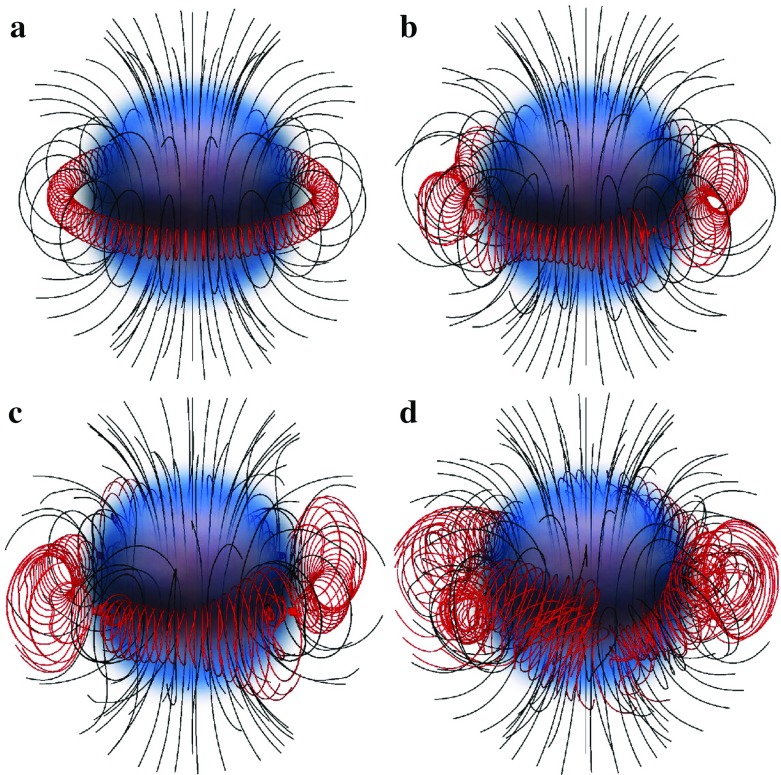
Time evolution of a magnetized, rotating neutron star model with central magnetic field $10^{17}\,\mathrm {G}$, initially rotating at 100 Hz with a polar to equatorial radius ratio of 0.99. Lines represent the magnetic field lines and the volume rendering the rest-mas density. The red field lines are seeded on the equatorial plane close to the neutral line, and the black field lines are seeded on the equatorial plane interior to the neutral line. The volume rendering is an contour surface at 37% of the central rest-mass density. The plots correspond to times **a** $t = 0\,\mathrm {ms}$, **b** $t = 17\,\mathrm {ms}$, **c** $t = 27\,\mathrm {ms}$ and **d** $t = 42\,\mathrm {ms}$. (Image reproduced with permission from Lasky et al. 2012)

Lasky et al. (2011, 2012) study the stability of initially purely poloidal magnetic fields threading nonrotating and uniformly rotating, polytropic, equilibrium neutron star models which are generated with the magstar module of the LORENE libraries. The studies vary several quantities: (i) the degree of rotation, (ii) the strength of the magnetic field, (iii) the stiffness of the equation of state, while the mass of the star is fixed at $1.31\,M_{\odot }$. For the evolutions, the THOR and HORIZON ideal magnetohydrodynamic codes are used (Zink et al. 2010; Zink 2011) keeping the spacetime fixed. Consistent with perturbation studies (Markey and Tayler 1973; Wright 1973), it is found that on an Alfvén timescale a magnetohydrodynamic instability develops (“kink” instability) leading to violent re-arrangement of the magnetic fields. Such a re-arrangement may be the engine behind magnetar flares. The simulations demonstrate that the re-arrangement leads to *f*-mode oscillations, but that gravitational waves from *f*-modes are not likely to be detected by current or near-future gravitational wave observatories. On the other hand, gravitational waves from Alfvén waves propagating inside the neutron star are more promising candidates. It is found that rotation separates the timescales of different instabilities, varicose vs kink instability, but both modes are always present regardless of the degree of rotation. The end-state magnetic field geometries derived from the simulations are nonaxisymmetric, with approximately 65% of the magnetic energy in the poloidal field and the authors conclude that these resemble twisted torus configurations, i.e., the toroidal magnetic field component is confined within the closed poloidal field lines. The development of the instability and the final magnetic field configuration in one of their rotating star cases is shown in Fig. 38.

Lasky and Melatos (2013) study tilted torus magnetic fields, which are defined as a superposition of a poloidal component extending from the stellar interior to its exterior, with symmetry axis tilted with respect to the spin axis, and an interior toroidal component, with symmetry axis aligned with the spin axis. Using the HORIZON code they perform a general relativistic magnetohydrodynamics evolution of a magnetized, differentially rotating, polytropic neutron star model (prepared with the RNS code), to argue that such tilted torus magnetic fields arise naturally. The significance of the result is that tilted torus magnetic fields, if they are of magnetar strength, lead to triaxial deformations on the star, and hence the star becomes a quasi-periodic emitter of gravitational waves. The authors argue that these configurations have a distinguishable gravitational wave signature and could be discerned from other magnetic field configurations, if detected by gravitational wave observatories.

Ciolfi et al. (2011), Ciolfi and Rezzolla (2012) also study the stability of initially purely poloidal magnetic fields threading nonrotating $\varGamma =2$ polytropic equilibrium neutron star models which are generated with the magstar module of the LORENE libraries. The mass of the neutron star is chosen to be $1.4\,M_{\odot }$ and the strength of the dipole magnetic field at the pole in the range $1-9.5\times 10^{16}\,\mathrm {G}$. The magnetic field extends from the NS interior to its exterior and to handle the exterior magnetic field the authors add a non-gauge invariant “resistive” term to the right-hand-side of the evolution equation for the vector potential of the form $\eta \nabla ^2 \mathbf{A}$, where $\eta$ is a constant. The evolutions are performed with the Whisky code and the spacetime is held fixed (Cowling approximation). To shorten the time for the development of the instability, a small, $m=2$ perturbation is added to the initial $\theta$ component of the fluid velocity. In agreement with perturbation theory (Markey and Tayler 1973; Wright 1973), it is found that the instability is triggered and accompanied by the production of toroidal magnetic field. As in Lasky et al. (2011, 2012), the instability occurs on an Alfvén timescale and saturates when the strength of the toroidal field is comparable to that of the poloidal one. Major rearrangements of the magnetic field take place that could lead to electromagnetic emission, and excite *f*-mode stellar oscillations. The evolutions settle to a solution that is stable on a dynamical/Alfvén timescale and the authors argue that a stable neutron star magnetic field configuration should comprise both toroidal and poloidal components.

#### Magnetically driven outflows

Shibata et al. (2011) and Kiuchi et al. (2012) perform axisymmetric and 3D, magnetohydrodynamic simulations of a differentially rotating star in full general relativity adopting the BSSN formulation. Their 3D evolutions are performed using a fixed-mesh-refinement hierarchy with the Balsara divergence-free interpolation scheme (Balsara 2001, 2009) coupled to the flux-CT constrained transport method for the magnetic field to remain divergence-free to machine precision even across refinement levels. They adopt a piecewise polytropic equation of state for initial equilibrium rotating neutron star model, and a hybrid $\varGamma$-law equation of state which consists of a cold part and a thermal part. The ADM mass of the differentially rotating neutron star ($2.02\,M_{\odot }$) is slightly larger than the TOV limit with the adopted equation of state ($2.01\,M_{\odot }$) but smaller than the corresponding supramassive limit ($2.27\,M_{\odot }$). They seed the star with a weak purely poloidal, dipolar magnetic field such that the maximum B-field strength (as measured in a frame comoving with the fluid) is $4.2\times 10^{13}$ or $1.7\times 10^{14}\,\mathrm {G}$, and such that the magnetic dipole moment is aligned with the angular momentum of the star. It is found that after the evolution begins strong outflows are launched with the Poynting ($L_B$) and matter ejection ($L_M$) luminosities scaling as

147 $$\begin{aligned}&L_B \sim 10^{47}\left( \frac{B_0}{10^{13}\,\mathrm {G}}\right) ^2\left( \frac{R_e}{10^{6}\,\mathrm {cm}}\right) ^3\left( \frac{\varOmega }{10^{4}\,\mathrm {rad/s}}\right) \,\mathrm {erg/s}, \end{aligned}$$

148 $$\begin{aligned}&L_M \sim 10^{48}\left( \frac{B_0}{10^{13}\,\mathrm {G}}\right) ^2\left( \frac{R_e}{10^{6}\,\mathrm {cm}}\right) ^3\left( \frac{\varOmega }{10^{4}\,\mathrm {rad/s}}\right) \,\mathrm {erg/s}. \end{aligned}$$

**Fig. 39:**
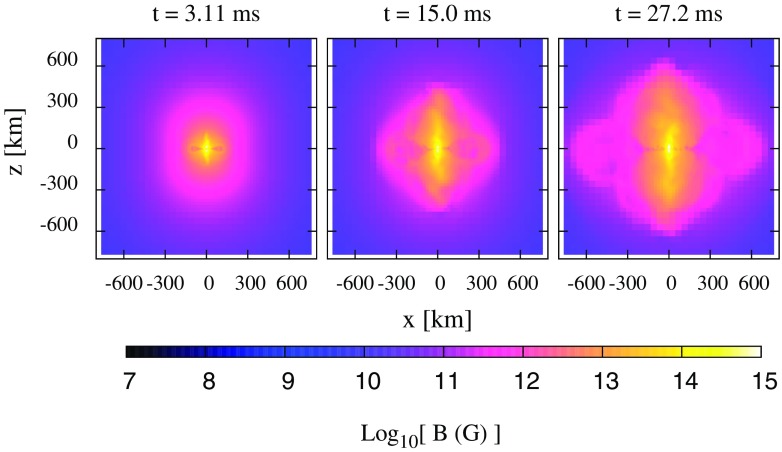
Snapshots, at select times, of the magnetic field strength on a the x − z (meridional) plane for the case with magnetic field strength $1.7\times 10^{14}\,\mathrm {G}$ evolved in full 3D dimensions. Image reproduced with permission from Kiuchi et al. 2012, copyright by APS)

These results hold both in axisymmetry and in 3 spatial dimensions. While the authors report that in 3 dimensions a kink instability (Goedbloed and Poedts 2004) develops, they find that the instability does not affect the outgoing luminosities because it saturates at a small amplitude. However, it does affect the geometry of the outflow (see Fig. 39) leading to nonaxisymmetric features. As noted by the authors these outflows could shine electromagnetically, but it is not very likely that the signals will be detectable.

Motivated by these earlier results, Siegel et al. (2014) perform 3D ideal magnetohydrodynamics simulations of a differentially rotating $\varGamma =2$ polytropic, hypermassive neutron star (with a mass of $2.43\,M_{\odot }$) which is initially seeded with either a dipolar magnetic field or a random magnetic field. The evolutions are performed using the Whisky code and adopting a vector potential formulation for maintaining the $\mathbf{\nabla }\cdot \mathbf{B}=0$ constraint, coupled to the generalized Lorenz gauge (Farris et al. 2012). As in Shibata et al. (2011) and Kiuchi et al. (2012) the authors also find outflows soon after the evolutions start. In the cases where a dipole magnetic field is initially seeded in the star, a collimated outflow along the stellar rotation axis is also launched in addition to a magnetized wind, whereas in the random magnetic field case, the outflow is more in a form of a wind and isotropic. The typical Poynting luminosity associated with these outflows is found to be

149 $$\begin{aligned} L_B \sim 10^{48}\left( \frac{B_0}{10^{14}\,\mathrm {G}}\right) ^2\left( \frac{R_e}{10^{6}\,\mathrm {cm}}\right) ^3\left( \frac{P}{10^{-4}\,\mathrm {rad/s}}\right) ^{-1}\,\mathrm {erg/s}, \end{aligned}$$

where *P* is the rotation period at the location of the spin axis.

#### Magnetospheric studies

Lehner et al. (2012) develop a novel scheme for matching the equations of ideal general relativistic magnetohydrodynamic stellar interiors to Maxwell’s equations for force-free electrodynamic or vacuum exteriors. The spacetime is dynamical and evolved using the generalized harmonic formulation in conjunction with black hole excision. Validating their method and code using the force-free aligned rotator test (see, e.g., Goldreich and Julian 1969; Contopoulos et al. 1999; Spitkovsky 2006; Contopoulos and Spitkovsky 2006; Komissarov 2006; McKinney 2006), and Michel monopole solution (Michel 1973), they study the electromagnetic emission arising from both nonrotating and rotating, collapsing, polytropic neutron star models. The initial data are generated using the magstar module of the LORENE libraries, and correspond to self-consistent rotating $\varGamma =2$ polytropes in unstable equilibrium, threaded by dipole magnetic fields. It is found that in the nonrotating stellar collapse approximately 1% (10%) of the stored energy in the initial magnetosphere is radiated away during the collapse in the force-free (electrovacuum) cases. The average outgoing Poynting luminosity for a nonrotating collapsing neutron star with a force-free exterior scales as

150 $$\begin{aligned} L_\mathrm{EM}\approx 10^{48} \bigg (\frac{B_\mathrm{pole}}{10^{15}\,\mathrm {G}}\bigg )^2\,\mathrm {erg/s}, \end{aligned}$$

and has a predominantly dipolar distribution. Here, $B_\mathrm{pole}$ is the strength of the initial magnetic field at the stellar pole. On the other hand, for rotating stellar collapse approximately 20% of the stored energy in the initial magnetosphere is radiated away during the collapse both in the force-free and electrovacuum cases. The average outgoing Poynting luminosity for rotating collapsing neutron stars with a force-free exterior scales as

151 $$\begin{aligned} L_\mathrm{EM}\approx 1.3\times 10^{48} \bigg (\frac{B_\mathrm{pole}}{10^{15}\,\mathrm {G}}\bigg )^2\,\mathrm {erg/s}, \end{aligned}$$

and has a predominantly quadrupolar distribution.

The electromagnetic emission from nonrotating collapsing neutron stars is also studied in Dionysopoulou et al. (2013) using a general relativistic resistive magnetohydrodynamic scheme in full general relativity assuming electrovacuum for the stellar exterior. It is found that up to 5% of the initial energy in the magnetosphere is radiated away and following the black hole formation the evolution of the magnetic field follows an exponential decay, with complex frequency matching the quasinormal mode ringing of a Schwarzschild black hole (Kokkotas and Schmidt 1999) to within a few percent. This result seems to be in disagreement with the calculations of Baumgarte and Shapiro (2003) who studied Oppenheimer–Snyder collapse of ideal magnetohydrodynamic matter matched onto an exterior electrovacuum and recovered the power-law decay anticipated from Price’s theorem (Price 1972).

The aforementioned studies focused on dynamical spacetime magnetospheric effects. However, special relativistic studies of stationary pulsar magnetospheres were performed well before these general relativistic studies. In particular, many flat-spacetime works attempted to compute the pulsar spin-down due to dipole emission in the limit of force-free electrodynamics. The first successful numerical solution of the pulsar equation was presented by Contopoulos et al. (1999), which was later followed by numerous studies of aligned and oblique rotators (see, e.g., Spitkovsky 2006; Contopoulos and Spitkovsky 2006; Komissarov 2006; McKinney 2006; Gruzinov 2007, 2008; Kalapotharakos and Contopoulos 2009; Tchekhovskoy and Spitkovsky 2013; Kalapotharakos et al. 2012a, b; Gruzinov 2013; Uzdensky and Spitkovsky 2013; Contopoulos et al. 2014 and references therein) that studied global features of the magnetosphere. These studies did not include the magnetized NS interior, and modeled the effects of rotation through a boundary condition on the spherical stellar surface, which is modelled as a perfect conductor. Simulations of pulsar magnetospheres in flat spacetime have produced important results, such as a proof of existence of a stationary force-free magnetospheric configuration, the calculation of the spin-down luminosity of force-free aligned and oblique rotators, and the evolution of the obliquity angle (Philippov et al. 2014), all in flat spacetime. There have also been some analytic efforts to understand the emission from an accelerated isolated pulsar in flat spacetime (see, e.g., Brennan and Gralla 2014; Gralla and Jacobson 2014).

Recently, a general relativistic resistive magnetohydrodynamics scheme in full general relativity was introduced by Palenzuela (2013). The scheme is presented and tested using the force-free aligned rotator solution. Using a rotating relativistic $\varGamma =2$ polytrope endowed with a dipole magnetic field, Palenzuela reports that the outgoing Poynting luminosity—the spin-down luminosity—differs by 20% from its flat spacetime value and several potential sources for this difference are listed, including resistive, general relativistic effects and the way the flat spacetime formula is applied to a general relativistic case.

**Fig. 40:**
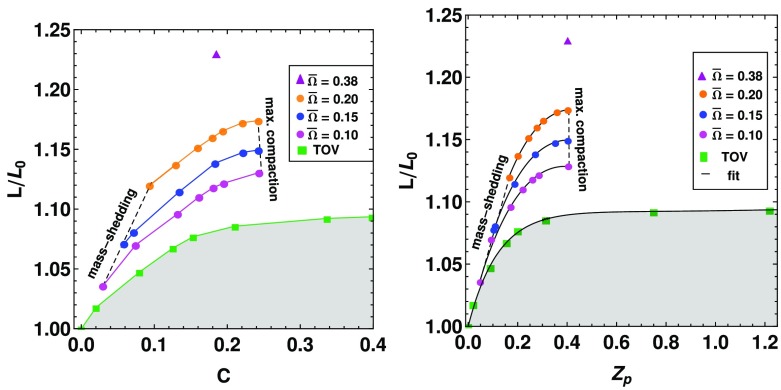
Pulsar spin-down luminosity *L* normalized by $L_0 = 1.02\mu ^2 \varOmega ^4$, the flat-spacetime result. Left panel: $L/L_0$ versus stellar compactness $C=M/R_e$, where $R_e$ is the equatorial radius. Right panel: *L* / *L*0 vs polar redshift $Z_p$. The parameter space for rotating stars is contained between the left dashed line (the mass-shedding limit) and the right dashed line (maximum compactness). The top point (triangle) represents the value for the supramassive neutron star limit for $n = 1$. For these rapidly rotating stars, the lower shaded zone is the area of the parameter space that cannot be reached, unless flat spacetime is assumed. In the constant angular velocity sequences $\bar{\varOmega }=\varOmega \cdot K^{n/2}$ is a dimensionless angular velocity, with *K* standing for the polytropic constant. (Image reproduced with permission from Ruiz et al. 2014, copyright by APS)

More recently, Ruiz et al. (2014) study the pulsar spin-down luminosity via time-dependent simulations in general relativity. The evolutions are performed using the technique developed by Paschalidis et al. (2013), Paschalidis and Shapiro (2013) for matching general relativistic ideal magnetohydrodynamics to its force-free limit. Equilibrium rotating, polytropic neutron star models of different compactnesses are considered using 3 constant-angular velocity sequences ranging from the mass-shedding limit to the maximum compactness configuration. The initial data are prepared using the Cook et al. code (Cook et al. 1992, 1994b, a). Both slowly rotating and rapidly rotating stars are considered. The stars are endowed with a general relativistic magnetic dipole (Wasserman and Shapiro 1983) and the electromagnetic fields are evolved keeping fixed both the spacetime and the fluid (a valid assumption for weak magnetic fields). The structure of the final, steady-state magnetosphere reached for all evolved stars resembles the structure of the magnetosphere found in flat spacetime studies. However, it is found that general relativity gives rise to a modest enhancement of the spin-down luminosity when compared to its flat spacetime value: the maximum enhancement found for $n=1$ polytropes is 23%, and for a rapidly rotating $n=0.5$ polytrope an even greater enhancement of 35% is found. The spin-down luminosity for all cases studied is shown in Fig. 40. This enhancement in the spin-down luminosity due to general relativistic effects has been confirmed in more recent simulations by Philippov et al. (2015) and Pétri (2016) who used approximate metrics for the spacetime around a rotating neutron star. However, semi-analytic work presented by Gralla et al. (2016) suggests that the general relativistic corrections in the slow-rotation limit should disappear (a result also mentioned in Philippov et al. 2015).

## Footnotes

1 Following the standard convention, we report numerical values of $\epsilon _c$ as $\epsilon _c/c^2$.

2 Notice that, although this particular EOS does not satisfy the current observational constraint of a $2\,M_{\odot }$ maximum-mass nonrotating model, the qualitative features of all sequences of models discussed here are generic for practically all EOSs.
